# A Novel Defined Pyroptosis-Related Gene Signature for Predicting the Prognosis of Endometrial Cancer

**DOI:** 10.1155/2022/7570494

**Published:** 2022-12-16

**Authors:** Shuguang Liu, Chao Zeng, Huaisheng Lv, Yan Zhang, Hong Xiong, Hongping Tang

**Affiliations:** ^1^Department of Pathology, The Eighth Affiliated Hospital, Sun Yat-sen University, Shenzhen, Guangdong Province, China; ^2^Department of Pathology, Affiliated Shenzhen Maternity & Child Healthcare Hospital, Southern Medical University, Shenzhen, Guangdong Province, China; ^3^Department of Pathology, The First Affiliated Hospital of Guangdong University of Pharmacy, Guangzhou, Guangdong Province, China; ^4^Department of Pathology, Shenzhen Longhua District Maternity & Child Healthcare Hospital, Shenzhen, Guangdong Province, China; ^5^Department of Statistics, University of California, Los Angeles, California, USA

## Abstract

Endometrial carcinoma (EC) is the second major female genital malignancy. Genetic signatures may be an improved choice to predict the prognosis of EC patients. The relationship between pyroptosis and tumours has attracted much attention in recent years. Here, we constructed a new pyroptosis-related gene (PRG) signature for predicting the prognosis of EC. In this study, gene data and clinical information of EC patients were obtained from The Cancer Genome Atlas (TCGA). Following the identification of PRGs correlated with EC prognosis, we further investigate the bioinformatics functions of these PRGs by univariate Cox regression analysis and Gene Ontology (GO) and Kyoto Encyclopedia of Genes and Genomes (KEGG) enrichment analyses. Then, we used the least absolute contraction and selection operator (LASSO) regression and multiple Cox regression analysis to construct a new PRG signature that contains seven PRGs (NFKB1, EEF2K, CTSV, MDM2, GZMB, PANX1, and PTEN) and performed the Kaplan-Meier (K-M) analysis, receiver operating characteristic curve (ROC) analysis, and principal component analysis (PCA) to evaluate the prognostic value of our novel PRG signature. Finally, we assessed the correlations between pyroptosis and immune cells/checkpoints through the CIBERSORT tool and single-sample gene set enrichment analysis (ssGSEA). The result suggested that our signature was powerful in predicting EC prognosis and may play a part in assessing response to immunotherapy in EC patients. In conclusion, our study established a novel PRG signature for EC, which can be used as an effective prognostic marker in clinical practice in the future.

## 1. Introduction

Endometrial cancer (EC) severely threatens women's health, ranking second in female genital malignancies. The incidence rate of endometrial cancer has been increasing significantly in recent years. According to the statistics, there were 417,000 new cases and 97,000 deaths in 2020 worldwide [1]. Although the therapeutic effect of EC is relatively good, a large number of patients still suffer from tumour recurrence, and approximately 18% of patients eventually die [2, 3]. Molecular classification and emerging targeted therapies can provide a basis for selecting clinical treatment strategies in EC [4, 5]. Therefore, exploring biomarkers to predict the prognosis is very important for the clinical diagnosis and treatment of EC.

Pyroptosis is a novel characterized form of programmed cell death (PCD) discovered after apoptosis and necrosis. Pyroptosis was first found in macrophages after pathogen infection and then confirmed to play a critical role in both inflammatory and immune defenses [6–9]. Recently, the role of pyroptosis in tumourigenesis, development, and treatment of tumours has attracted more and more attention. It was indicated that pyroptosis could promote inflammatory cell death of cancer and inhibit the proliferation and migration of tumour cells, thus affecting the progress and prognosis of tumour [10, 11]. For example, the expression of pyroptotic inflammasome GSDMD is decreased in gastric cancer (GC) cells, which promotes the proliferation of GC cells [10]. Recent studies have shown that PRG signatures can be used as effective prognostic markers in ovarian cancer [12], lung cancer [13], bladder cancer [14], and squamous cell cancer in the head and neck [15]. However, the prognostic value of PRGs in EC has not yet been elucidated.

In this study, we performed a systematic study to construct a prognostic multigene signature with PRGs and validated it in the TCGA cohort. This novel seven-PRG signature provides ideas for predicting the prognosis of EC and shares intriguing insights for more in-depth explorative research upon the mechanism of tumour immunity. Also, it may provide a basis for developing targeted anticancer therapy of EC.

## 2. Materials and Methods

### 2.1. Datasets and Preprocessing

The profiling gene data and corresponding clinical data of patients from 35 normal and 515 tumour tissues were obtained from TCGA (https://tcga-data.nci.nih.gov/tcga/) on November 12, 2021. Only samples from the primary tumour and RNA-seq data from fresh frozen samples were chosen in this study, and criteria were excluded as follows: data from FFPE tissue samples, duplicate RNA-seq data in the same patient sample, and cases without survival information. Furthermore, to develop a prognostic model, EC samples from TCGA database (after removing samples with patient's survival time < 30 days) were randomly divided into a training set (*n* = 387) and a testing set (*n* = 128), and the ratio was set at 3 : 1.

### 2.2. Identification of PRGs in EC

A total of 133 PRGs were extracted from the GeneCards database (https://www.genecards.org/) to identify differentially expressed genes (DEGs, listed in Supplementary Table S1). The raw read counts were normalized, and DEGs related to pyroptosis were identified using the R package “DEseq2” [16]. The protein-protein interaction (PPI) network for the DEGs mentioned above was constructed by search tool for the retrieval of interacting genes (STRING, https://string-db.org/) [17].

### 2.3. Functional Enrichment Analysis

The Kyoto Encyclopedia of Genes and Genomes (KEGG, http://www.kegg.jp/) pathway enrichment analyses and Gene Ontology (GO, http://www.geneontology.org) annotation were used to explore the biological functions of DEGs associated with pyroptosis through “org.Hs.eg.db,” “clusterProfiler,” “ggplot2,” and “enrichplot” packages in R. Adjusted *p* value < 0.05 was established as the statistical significance threshold.

### 2.4. Construction of Prognostic PRG Signature

Univariate Cox regression analysis was performed first to assess the correlation between PRGs and the survival status of EC patients in the training set. Then, to reduce the dimensionality of the candidate genes according to the best penalty factor (*λ*), the LASSO regression analysis was applied. After multivariate Cox regression analysis was performed for these selected DEGs and their coefficients, a risk signature was constructed using the Akaike information criterion for the stepwise backward/forward model. The risk score (RS) for each patient was calculated using the risk formula: RS = ∑*Ni* (expression of signature gene *i*^∗^ coefficient *βi*).

### 2.5. Evaluation and Validation

According to the median risk score of our PRG signature, EC patients were divided into low-risk and high-risk groups. Survival curves were generated through the Kaplan-Meier method for the survival difference. Receiver operation characteristic (ROC) curves and area under the curve (AUC) values corresponding to 1, 2, and 3 years were used to evaluate the predictive efficiency of the signature. Principal component analysis (PCA) was adopted via the “prcomp” function in the “stats” R package. Correlation analysis was performed to verify the connections between the clinicopathological characteristics of the patients and the RS. The independent prediction ability was demonstrated by univariate and multivariate Cox regression analyses. In addition, stratified analysis was used to examine the precision of prognostic prediction based on different clinicopathological groups.

### 2.6. Construction and Evaluation by Nomogram

Based on the clinicopathological features and the RS, a nomogram model was then established to predict 1-, 2-, and 3-year mortality with the rms package of R. The calibration curve with the foreign package is used to determine the predictive performance of the nomogram.

### 2.7. Tumour Immunity Analysis

The CIBERSORT algorithm and the Spearman correlation were used to assess different distributions of 22 types of tumour-infiltrating immune cells (TIICs) with the variation of the risk score. The correlation between the signature-based risk score and the expression of the immune checkpoint genes (programmed cell death protein 1 (PD1), PD ligand 1 (PDL1), and cytotoxic T-lymphocyte-associated protein 4 (CTLA4)) was assessed by Pearson's test.

### 2.8. Statistical Analysis

All statistical analyses were performed with the R (version 4.1.2, http://www.r-project.org) and R Bioconductor packages. The Kaplan-Meier curve with a log-rank test from the survival package and Cox proportional risk regression models were used for survival analysis. ROC analysis was used to detect the sensitivity and specificity of the gene signature risk score to predict survival. The AUC values can be used as an index of prognostic precision. In all analyses, *p* value < 0.05 stands for a statistically significant difference.

## 3. Results

### 3.1. Defining the Expression of PRGs in EC

**Figure 1:**
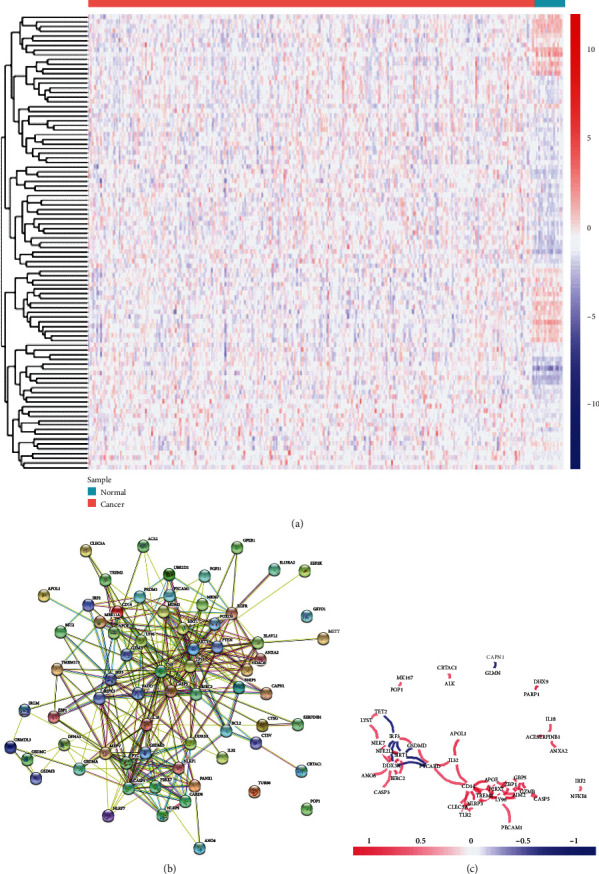
Expression and interaction of the PRGs in EC. (a) Heatmap (blue: low expression level; red: high expression level) of the PRGs in EC (green) and normal endometrial tissues (red). (b) Interactions of the PRGs shown by the PPI network (score = 0.9). (c) Correlation network containing PRGs with core > 0.5 (red: positive correlations; blue: negative correlations).

We first explored the expression of 133 PRG expressions from 35 normal and 515 tumour tissues in TCGA, using the DESeq2 package in R. A total of 97 PRGs were identified (all *p* < 0.05), including 64 upregulated genes and 33 downregulated genes. Details of these PRGs were listed in Table S2. A heatmap of these genes is visualized in Figure 1(a). A PPI analysis with the minimum required interaction score of 0.9 (the highest confidence) was constructed to explore the interactions of the PRGs, which revealed that TP53, NLRP3, CASP8, NFKB1, AKT1, BIRC2, TNF, CASP3, JUN, and PYCARD (all node degree > 10) were the core genes (Figure 1(b)). The correlation network containing PRGs with core > 0.5 is shown in Figure 1(c).

### 3.2. Functional Enrichment of the PRGs in EC

**Figure 2:**
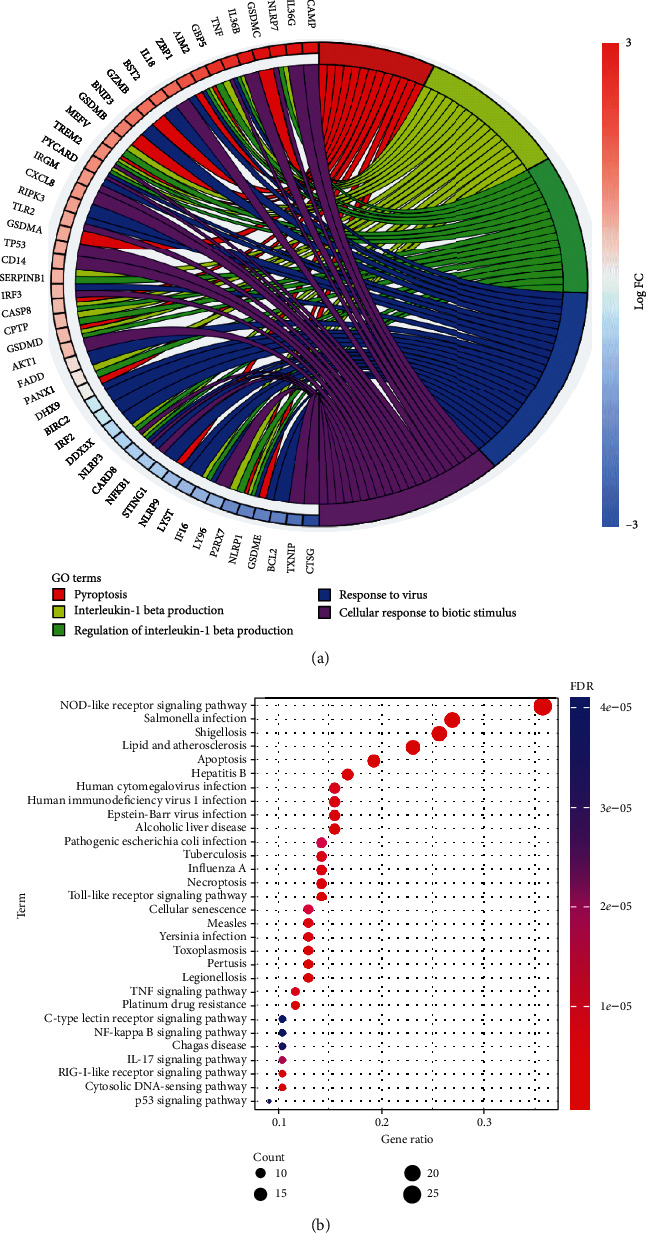
Enrichment analyses of PRGs in EC. (a) GO term analyses indicated the mainly biological functions involved in PRGs in EC. (b) Bubble graph of KEGG pathway analyses suggested the mainly biological functions involved in PRGs in EC.

Function annotation analyses of the 97 PRGs were performed. GO enrichment (listed in Supplementary Table S3) suggested that these genes were mainly involved in “pyroptosis,” “interleukin-1 beta production,” “regulation of interleukin-1 beta production,” “response to virus,” and “cellular response to biotic stimulus” (Figure 2(a)). KEGG pathway enrichment analysis (listed in Supplementary Table S4) suggested that these PRGs were mainly involved in “NOD-like receptor signaling pathway,” “*Salmonella* infection,” “shigellosis,” “lipid and atherosclerosis,” and “apoptosis” (Figure 2(b)).

### 3.3. Identification and Validation of the Prognostic PRG Signature

**Figure 3:**
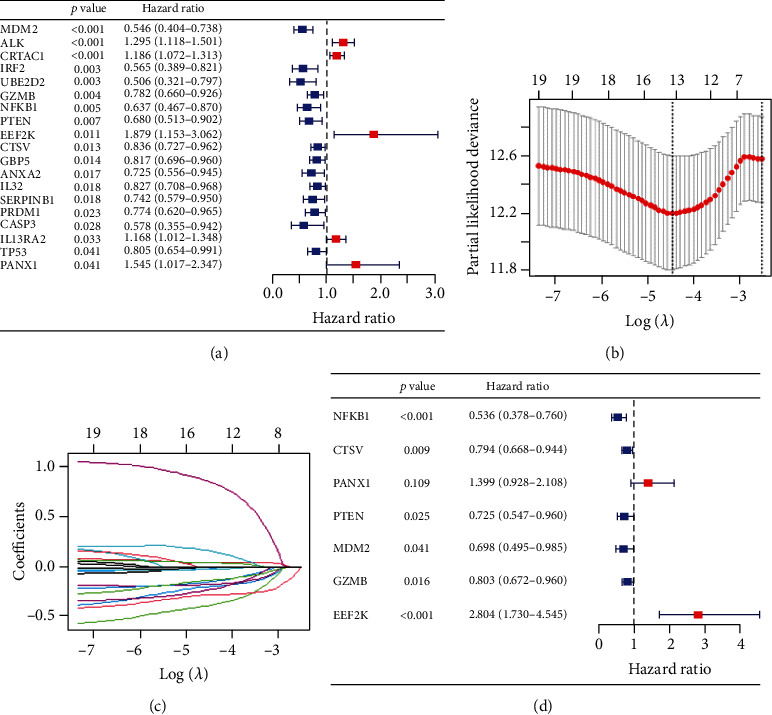
Construction of the risk signature in the training set. (a) Univariate Cox regression of OS for 19 PRGs with *p* < 0.05. (b) LASSO coefficient distribution of 19 PRGs. (c) LASSO model was adjusted based on the minimum criteria (regularization parameter *λ*). (d) Multivariate Cox regression analysis revealed 7 PRGs as independent prognostic factors for EC patients.

Univariate Cox regression analysis was first performed to identify the prognostic-associated PRGs. Nineteen genes (MDM2, ALK, CRTAC1, IRF2, UBE2D2, GZMB, NFKB1, PTEN, EEF2K, CTSV, GBP5, ANXA2, IL32, SERPINB1, PRDM1, CASP3, IL13RA2, TP53, and PANX1) that meet the criteria *p* < 0.5 are obtained for further analysis (Figure 3(a)). Then, by performing a least absolute shrinkage and selection operator (LASSO) (Figure 3(b)), fourteen PRGs were sought out according to the optimal *λ* value (Figure 3(c)). Finally, multivariate Cox regression analysis revealed that seven PRGs (NFKB1, CTSV, PANX1, PTEN, MDM2, GZMB, and EEF2K) were independent prognostic factors for patients with EC (Figure 3(d)).

As calculated in the following, the risk score = (−0.62342 × expression value of NFKB1) + (−0.23063 × expression value of CTSV) + (0.335488 × expression value of PANX1) + (−0.3216 × expression value of PTEN) + (−0.35904 × expression value of MDM2) + (−0.21909 × expression value of GZMB) + (1.030988 × expression value of EEF2K).

**Figure 4:**
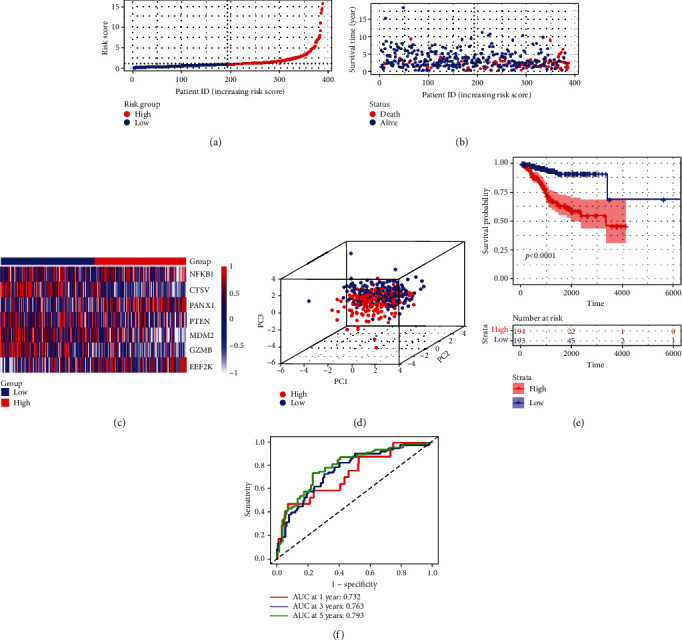
Construction of the risk signature in the training cohort. (a) Risk score map of patients in the high- and low-risk groups. (b) Survival status map of patients in the death (red spheres) and alive (blue spheres) groups. (c) Heatmap of gene expression in the high- and low-risk groups. (d) PCA plot showing different clustering between EC (red spheres) and normal endometrial tissues (blue spheres). (e) The Kaplan-Meier survival curves of patients in the high- and low-risk groups. (f) ROC curve evaluating the predictive performance of the risk signature.

Based on the median risk score, 387 EC patients in the training set were divided into low-risk (*n* = 193) and high-risk groups (*n* = 194). The distribution patterns of risk scores and survival status are illustrated in Figures 4(a) and 4(b). A heatmap exhibited seven-gene expression between the high- and low-risk groups (Figure 4(c)). PCA showed that patients with different risks were satisfactorily divided into two clusters (Figure 4(d)). The Kaplan-Meier curve showed that patients in the high-risk group had more deaths and shorter survival times than those in the low-risk group (Figure 4(e)). The areas under the ROC curve for predicting 1-, 3-, and 5-year survival rates were 0.732, 0.763, and 0.793, respectively (Figure 4(f)).

**Figure 5:**
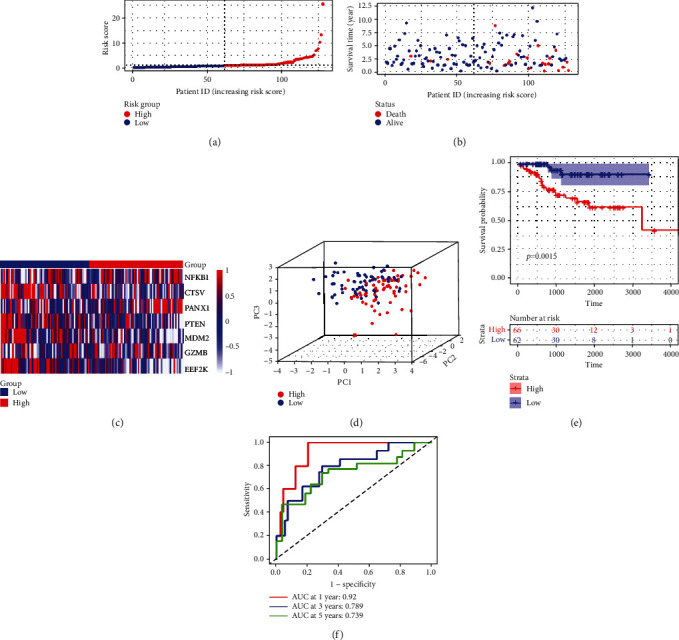
Construction of the risk signature in the test cohort. (a) Risk score map of patients in the high- and low-risk groups. (b) Survival status map of patients in the death (red spheres) and alive (blue spheres) groups. (c) Heatmap of gene expression in the high- and low-risk groups. (d) PCA plot showing different clustering between EC (red spheres) and normal endometrial tissues (blue spheres). (e) The Kaplan-Meier survival curves of patients in the high- and low-risk groups. (f) ROC curve evaluating the predictive performance of the risk signature.

**Figure 6:**
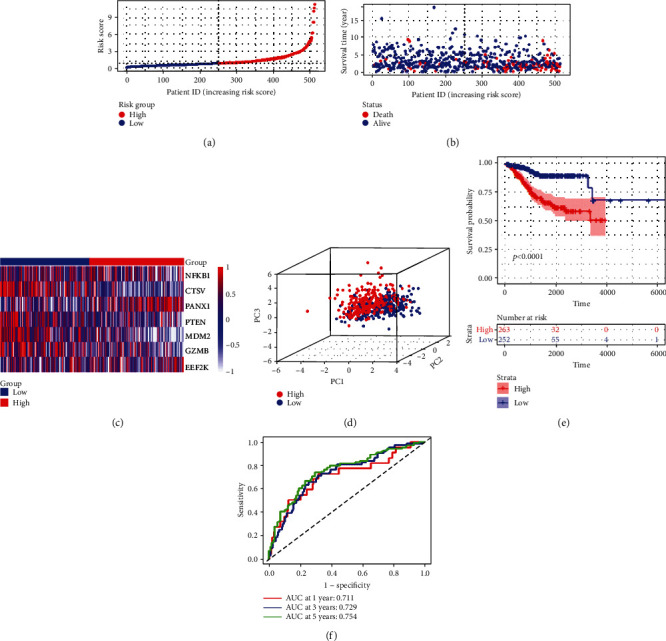
Construction of the risk signature in the entire cohort. (a) Risk score map of patients in the high- and low-risk groups. (b) Survival status map of patients in the death (red spheres) and alive (blue spheres) groups. (c) Heatmap of gene expression in the high- and low-risk groups. (d) PCA plot showing different clustering between EC (red spheres) and normal endometrial tissues (blue spheres). (e) The Kaplan-Meier survival curves of patients in the high- and low-risk groups. (f) ROC curve evaluating the predictive performance of the risk signature.

To validate the efficiency of the signature, based on the same median risk score of the training set, 128 patients in the testing set were divided into low-risk (*n* = 62) and high-risk groups (*n* = 66). The distribution patterns of risk scores and survival status are illustrated in Figures 5(a) and 5(b). A heatmap exhibited seven-gene expression between the high- and low-risk groups (Figure 5(c)). PCA showed that patients with different risks were satisfactorily divided into two clusters (Figure 5(d)). The Kaplan-Meier curve showed that patients in the high-risk group had more deaths and shorter survival times (Figure 5(e)). The AUCs for predicting 1-, 3-, and 5-year survival rates were 0.92, 0.789, and 0.739, respectively (Figure 5(f)). Meanwhile, we performed the same analyses in the entire set. The distribution patterns of risk scores and survival status are illustrated in Figures 6(a) and 6(b), and a heatmap exhibiting seven-gene expression between the high- and low-risk groups is shown in Figure 6(c). PCA showed that patients with different risks were satisfactorily divided into two clusters (Figure 6(d)). The Kaplan-Meier curve showed that patients in the high-risk group had more deaths and shorter survival times (Figure 6(e)). The AUCs for predicting 1-, 3-, and 5-year survival rates were 0.711, 0.729, and 0.754, respectively. (Figure 6(f)).

### 3.4. Correlation of the PRG Prognostic Signature with Clinical Features

**Figure 7:**
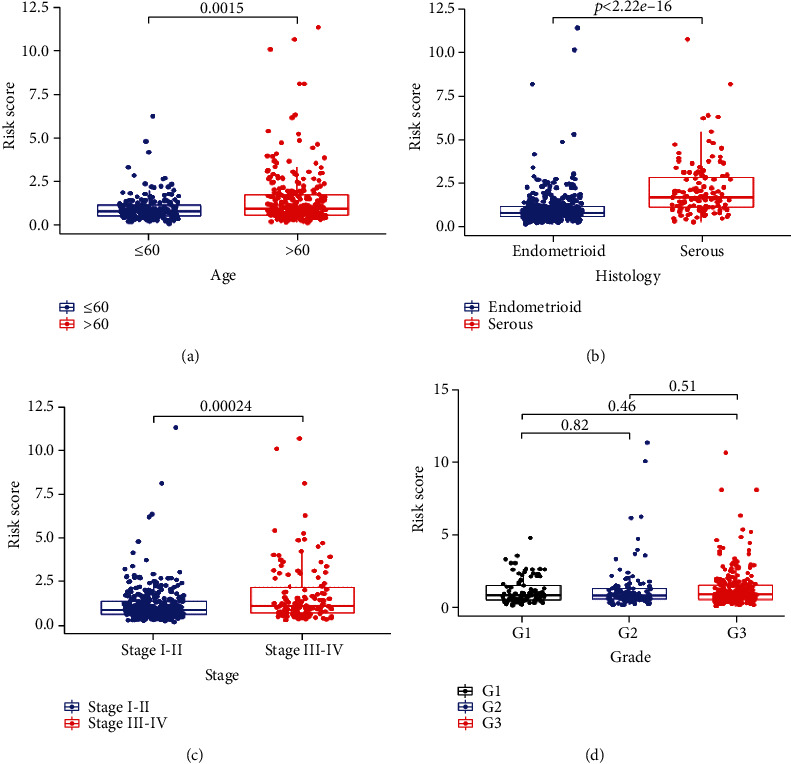
The PRG signature was associated with clinicopathological features of EC. The correlation between the signature risk scores and (a) age (≤60 and >60), (b) histology (endometrioid and serous), (c) stage (stages I-II and stages III-IV), and (d) grade (grade 1, grade 2, and grade 3).

The correlation analysis showed significant relationship between the risk scores and clinicopathological features such as age (Figure 7(a), *p* = 0.0015), histology (Figure 7(b), *p* < 2.22*e* − 16), and stage (Figure 7(c), *p* = 0.00024), while there was no significant relationship between the risk scores and grade (Figure 7(d), all *p* > 0.05). These results suggested that our PRGs may be associated with EC progress.

**Figure 8:**
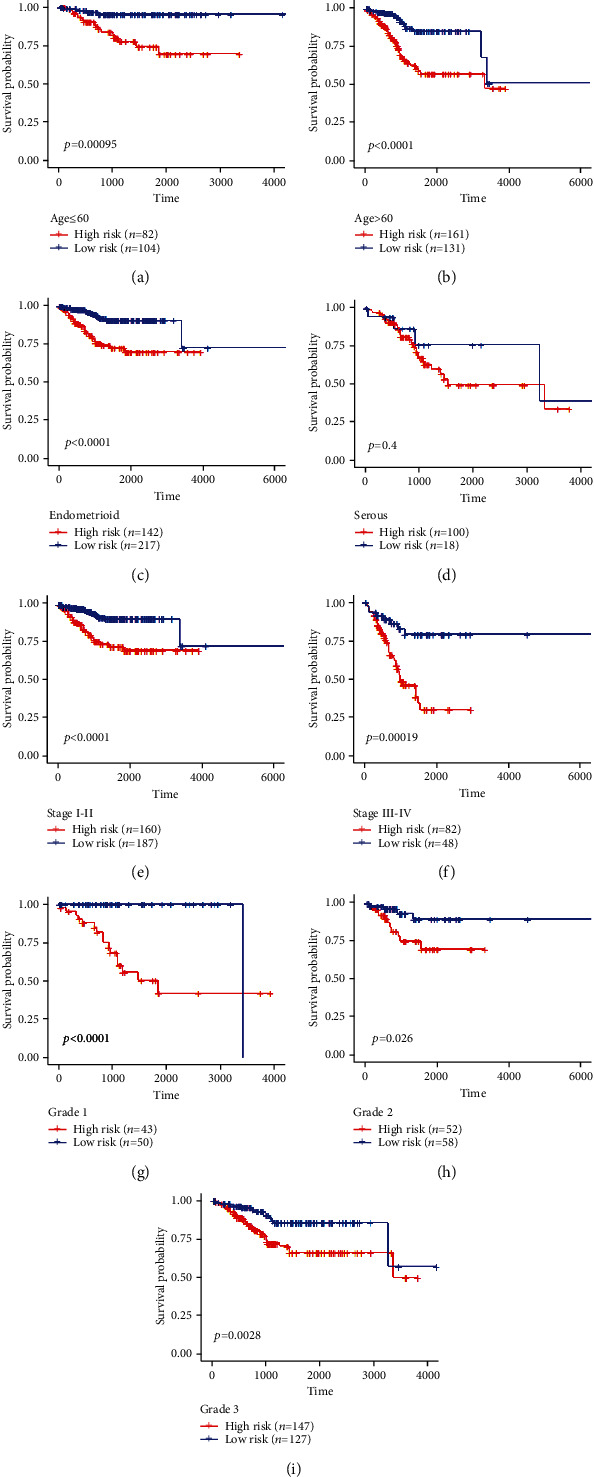
Survival curve showed the OS rates of the high- and low-risk groups stratified into different clinicopathological subgroups: (a) age ≤ 60, (b) age > 60, (c) endometrioid, (d) serous, (e) stages I-II, (f) stages III-IV, (g) grade 1, (h) grade 2, and (i) grade 3.

The stratified analysis showed that high risk was an independent index of poorer survival outcome in comparison to low risk based on different subgroups of clinicopathological features, such as age ≤ 60 (*p* = 0.00095, Figure 8(a)) and >60 (*p* < 0.0001, Figure 8(b)), endometrioid (*p* < 0.0001, Figure 8(c)) and serous (*p* = 0.4, Figure 8(d)), stages I-II (*p* = 0.0076, Figure 8(e)) and stages III-IV (*p* = 0.00019, Figure 8(f)), grade 1 (*p* < 0.0001, Figure 8(g)), grade 2 (*p* = 0.026, Figure 8(h)), and grade 3 (*p* = 0.0028, Figure 8(i)). The result suggested that our PRG signature was powerful to predict the prognosis in different subgroups of EC patients.

### 3.5. Independent Predictive Value of PRG Risk Signature

**Figure 9:**
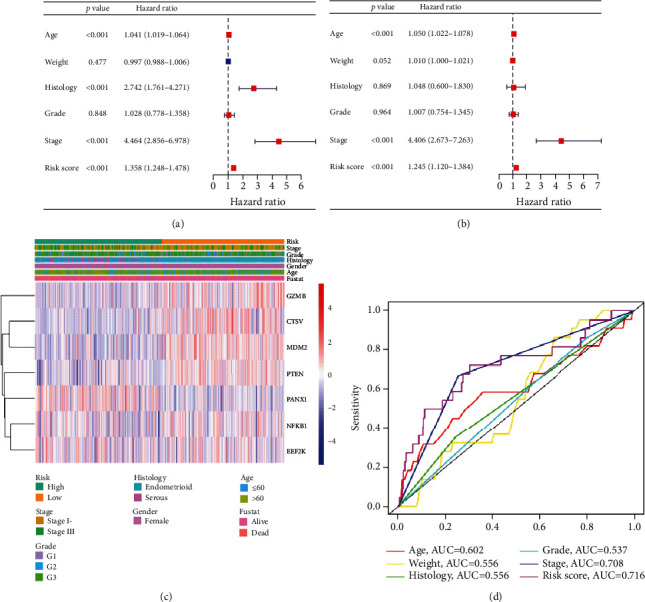
Relationship between the risk scores and clinicopathological factors in EC. (a) Forest plot of univariate analysis for the entire TCGA cohort. (b) Forest plot of multivariate analysis for the entire TCGA cohort. (c) Heatmap showed the distribution of clinicopathological factors and seven PRGs between the high- and low-risk groups. (d) The AUC of the ROC curve showed the prognostic accuracy of age, weight, histology, grade, stage, and risk scores.

Univariate and multivariate analyses were performed to verify the PRG risk signature as an independent prognostic factor in EC patients. Univariate analysis (listed in Supplementary Table S5) revealed that PRG risk score, as well as age, histology, and stage, was significantly correlated with the patient's survival time (all *p* < 0.001, Figure 9(a)). Multivariate analysis (listed in Supplementary Table S6) demonstrated that PRG risk score, age, and stage predict the prognosis independently in patients with EC (all *p* < 0.001, Figure 9(b)). A heatmap for the connection between the predictive gene signature and clinicopathological features is shown in Figure 9(c). ROC curve analysis showed that the AUC value of the risk score was 0.716, which was significantly higher than that of patients' age (AUC = 0.602) and patients' weight (AUC = 0.556), histology (AUC = 0.556), grade (AUC = 0.537), and stage (AUC = 0.708) (Figure 9(d)). These results suggest that this PRG signature was an effectively independent predictor of the prognosis of EC patients.

### 3.6. Nomogram Building and Validation

**Figure 10:**
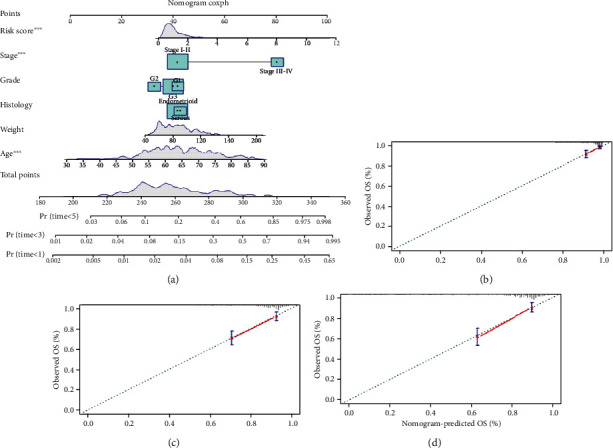
Nomogram for predicting the 1-, 3-, and 5-year survival probability of patients with EC. (a) Age, weight, histology, grade, stage, and risk score fitted into the nomogram. Calibration curves of (b) 1 year, (c) 3 years, and (d) 5 years for the nomogram.

To further estimate EC patients' survival probability, a comprehensive prognostic nomogram was built based on six parameters, including age, weight, histology, grade, stage, and risk score (Figure 10(a)). The calibration plots showed excellent consistency between the nomogram predictions and actual observations in terms of the 1-year (Figure 10(b)), 3-year (Figure 10(c)), and 5-year (Figure 10(d)) survival rates in the entire TCGA cohort (listed in Supplementary Table S7). It was also confirmed the efficiency of prognosis prediction of our PRG risk signature.

### 3.7. Immunological Activity between Different Risk Subgroups

**Figure 11:**
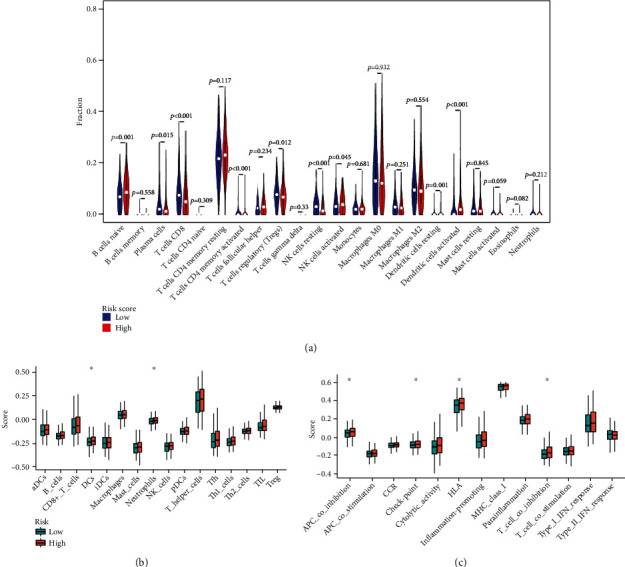
TIICs of EC patients. (a) Violin plot showed the different proportions of TIICs in the high- and low-risk group. Comparison of the ssGSEA scores for immune cells (b) and immune pathways (c) between the high- and low-risk group (^∗^*p* < 0.05).

CIBERSORT was implemented to evaluate the association between the risk score and the 22 types of tumour-infiltrating immune cells (TIICs) in EC. We observed significantly higher proportions of B cell naïve, T cell CD4 memory resting, NK cells activated, and dendritic cells activated and lower proportions of plasma cells, CD8+ T cells, T cell regulatory (Tregs), NK cell resting, and dendritic cell resting in the high-risk group (Figure 11(a)). Then, the enrichment scores of 16 types of immune cells and the activity enrichment analysis of 13 immune-related pathways were compared between TCGA dataset using ssGSEA.  Figure 11(b) shows that there are significantly higher proportions of immune cells, such as DCs and neutrophils in the high-risk group. Moreover, four immune pathways, such as APC_co_inhibition, checkpoint, HLA, and T_cell_coinhibition, were also significantly higher in the high-risk group (Figure 11(c)). The results indicated that the infiltration of these immune cells and pathways might significantly influence the prognosis of EC patients.

**Figure 12:**
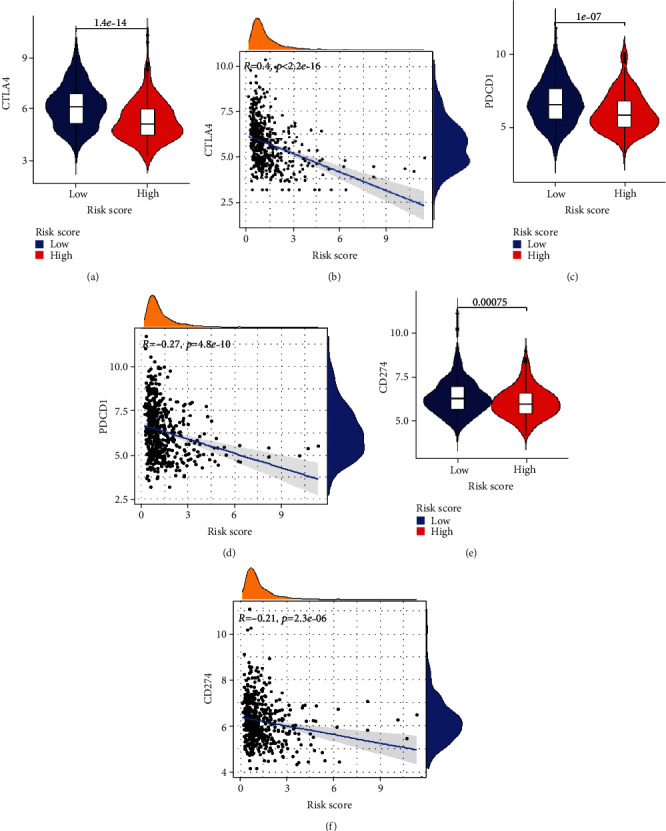
The association between PRG signature and immune checkpoint genes. (a) Expression of CTLA4 in low and high risk. (b) Correlation between risk score and CTLA4. (c) Expression of PD1 in low and high risk. (b) Correlation between risk score and PD1. (e) Expression of PDL1 in low and high risk. (f) Correlation between risk score and PDL1.

The expression levels of immune checkpoint genes and correlation analysis between the high- and low-risk groups showed significant decrease of CTLA4, PD1, and PDL1 in the high-risk group: the expression levels of CTLA4 (*p* = 1.4*e* − 14, Figure 12(a)) and the correlation between risk score and CTLA4 (cor = −0.4, *p* < 2.2*e* − 16, Figure 12(b)), the expression levels of PD1 (*p* = 1.4*e* − 14, Figure 12(c)) and the correlation between risk score and PD1 (cor = −0.27, *p* < 4.8e − 10, Figure 12(d)), and the expression levels of PDL1 (*p* = 0.00075, Figure 12(e)) and the correlation between risk score and PDL1 (cor = −0.21, *p* = 2.3*e* − 06, Figure 12(f)). These data indicated that the PRG signature might play a part in assessing response to immunotherapy in EC patients.

## 4. Discussion

EC is one of the most common female genital malignancies and threatens women's lives worldwide [18]. Because of the lack of effective screening methods, there is still a large part of EC that is difficult to diagnose at the early stage [19]. The current clinicopathological features, such as age, tumour size, and tumour histological type, are not sufficient to accurately predict the outcome of patients with EC. Finding new molecular mechanisms is very necessary for the clinical diagnosis and treatment of EC.

As a novel immunogenic form of inflammatory programmed cell death pathway [20], pyroptosis can participate in local inflammation and attract immune cell infiltration, which provides a good opportunity to alleviate the immunosuppression of the tumour microenvironment [21]. It has been proven that pyroptosis may play various roles in tumours and PRGs have the effect of inhibiting tumour growth and tumour immunity [10]. Ye et al. [12] showed a novel signature of seven PRGs (AIM2, PJVK, PLCG1, GSDMA, ELANE, CASP3, and CASP6) for predicting the prognosis of ovarian cancer (OC). Zhang et al. [22] identified a new signature featuring seven PRGs (BAK1, CHMP4B, NOD2, NLRP6, GSDMC, PLCG1, and SCAF11) in predicting the prognosis of hepatocellular carcinoma (HCC) patients. Chen et al. [14] constructed eight-PRG signature (AIM2, BAK1, GZMA, GZMB, IRF1, NOD2, TNF, and TP63) to predict the survival in bladder cancer (BC) patients. Qian et al. [15] performed a novel PRG signature (GSDME, IL1B, NLRP1, and NLRP6) for prognostic prediction of head and neck squamous cell carcinoma. In EC, Chen et al. [23] recently analyzed 33 PRGs between tumour samples and normal samples and obtained six pyroptosis-related prognostic DEGs (GPX4, GSDMD, GSDME, IL6, NOD2, and PYCARD).

In this study, using bioinformatics, we compared the expression of 133 currently known PRGs between EC and normal endometrial tissues in TCGA dataset and identified 19 DEGs associated with pyroptosis in EC. In order to further assess the prognostic value of these PRGs, we constructed 7-gene signature (NFKB1, CTSV, PANX1, PTEN, MDM2, GZMB, and EEF2K) via Cox univariate analysis and LASSO Cox regression analysis and then subsequently performed GO and KEGG to validate our PRG risk signature.

The nuclear factor-*κ*B (NF-*κ*B) signaling pathway plays an important role in inflammation and immune response [24]. As a subunit of NF-*κ*B, NFKB1 has been shown to be a pathway-specific suppressor of inflammation, aging, and tumours such as hematological malignancies [25]. Upregulation of NFKB1 can promote the invasiveness of breast cancer cells in vitro [26]. Cathepsin V (CTSV) is a member of the cathepsin family, which is highly expressed in activated macrophages and is involved in inflammatory diseases like myasthenia gravis [27, 28]. Studies showed that high expression of CTSV was associated with poor prognosis of breast cancer [29], and ectopic expression of CTSV increased the number of migrated and invaded colorectal cancer cells in vivo [30].

Pannexin 1 (PANX1) is a critical ATP-releasing channel and widely participates in the regulation of the tumour immune microenvironment by infiltrating multiple immune cells, such as cancer-associated fibroblast cells, macrophage, lymphocytes, and neutrophil cells [31]. High expression of PANX1 was significantly related to the poor outcome of multiple cancers, especially in pancreatic adenocarcinoma (PAAD) [32]. In addition, PANX1 overexpression is associated with the EMT transformation of breast cancer cells and poorer clinical outcomes in breast cancer patients [33]. As a tumour suppressor gene, phosphatase and tensin homolog (PTEN) protein plays an important role in PI3K/AKT pathway [34]. The inactivation of PTEN may be considered a key factor for early endometrial tumourigenesis [35]. Similar to our study, Kanamori et al. found that PTEN was a significant prognostic indicator of EC [36]. As a critical negative regulator of the tumour suppressor p53, mouse double minute 2 (MDM2) can bind mutant and wild-type p53 protein and plays a key role in controlling its function [37]. MDM2 is abnormally upregulated in several types of tumours, especially those of mesenchymal origin [38]. Ambros et al. first reported that there was a selective correlation of expression between MDM2 and p53 expression in EC [39]. Buchynska et al. further proved that high expression of p53 with low expression of MDM2 might be the characteristic features of low-differentiated EC [40]. In our study, as a prognostic PRG, the expression level of MDM2 in EC is also lower than in normal endometrial tissues. Granzyme B (GZMB) is a component of cytolytic granules within cytotoxic T lymphocytes (CTLs) and natural killer (NK) cells, which are involved in several pathologies, including the formation of the tumour microenvironment [41]. GZMB expression was associated with worse clinical outcomes in non-small-cell lung cancer [42] and with early signs of metastasis in colorectal cancer [43]. Eukaryotic elongation factor 2 kinase (EEF2K) is a new member of atypical protein kinase and acts as a negative regulator of cell growth [44]. It was shown that EEF2K is activated and overexpressed in many cancers. EEF2K can positively help to improve breast cancer cells' survival ability under nutrient deprivation or insufficient growth factor [45]. EEF2K is also a clinical indicator of metastasis and prognosis of stomach adenocarcinoma and could serve as a potential therapeutic target [46].

In our study, this PRG prognostic signature's association with EC progression can be preferably illustrated through the results of correlation analysis and stratified analysis, and this finding exerts satisfactory predicting power in terms of the survival period of EC patients. The development of univariate and multivariate analyses facilitates our verification, which suggests that PRG's risk can serve as an independent prognostic factor in EC patients.

Furthermore, in-depth exploring the mechanisms of this genes signature, CIBERSORT functional analysis of immune cell subsets revealed significantly higher proportions of B cells naïve, T cell CD4 memory resting, NK cells activated, and dendritic cells activated and lower proportions of plasma cells, CD8+ T cells, Tregs, NK cell resting, and dendritic cell resting in the high-risk group of EC. Then, the enrichment score analyses showed that there were significantly higher proportions of immune cells, such as DCs and neutrophils, and four immune pathways, such as APC_co_inhibition, checkpoint, HLA, and T_cell_coinhibition, were also observed to be significantly higher in the high-risk group (Figure 10(c)). These results indicated that reduced levels of antitumour immunity might lead to a poor prognosis. Additionally, our study showed a significant decrease in immune checkpoint genes of CTLA4, PD1, and PDL1 in the high-risk group. Current studies have shown that immunotherapy, especially immune checkpoint inhibitors (ICIS), may produce lasting therapeutic effects. The combined application of induced blepharoptosis and ICIS can enhance anticancer activity [47]. The PD-1 pathway is a critical pathway of immunosuppression in the tumour microenvironment. The generation of endogenous antitumour immunity to inhibit cancer development can be ideally achieved through the inhibition of PD-1 and PD-L1 [48]. Further research on the pyroptosis-related immune mechanism will be beneficial to guide clinical diagnosis and treatment of EC.

In summary, more and more studies had shown that PRGs are closely related to tumour prognosis, but as a complex gene network, there may be differences in key genes related to prognosis in different types of tumours and even in different research groups of the same type of tumours. Here, we developed a novel PRG signature using different platforms and validated this signature model. Our study suggested that using this seven-PRG signature is able to predict prognosis in patients with EC. It may provide new insight into the molecular mechanism and a classification tool in the clinical practice of EC.

## Data Availability

The datasets and code generated and analyzed in this study are available from the corresponding author on request.

## Supplementary Materials

Supplementary 1 Table S1: 133 pyroptosis-related genes retrieved from the GeneCards.

Supplementary 2 Table S2: 97 pyroptosis-related DEGs in EC.

Supplementary 3 Table S3: GO enrichment analysis.

Supplementary 4 Table S4: KEGG pathway analysis.

Supplementary 5 Table S5: univariate Cox regression analysis.

Supplementary 6 Table S6: multivariate Cox regression analysis.

Supplementary 7 Table S7: clinicopathological features as well as risk score.

## References

[1] Sung H., Ferlay J., Siegel R. L. (2021). Global cancer statistics 2020: GLOBOCAN estimates of incidence and mortality worldwide for 36 cancers in 185 countries. *CA: A Cancer Journal for Clinicians*.

[2] Trojano G., Olivieri C., Tinelli R., Damiani G. R., Pellegrino A., Cicinelli E. (2019). Conservative treatment in early stage endometrial cancer: a review. *Acta Bio-Medica*.

[3] Del Carmen M. G., Boruta D. M., Schorge J. O. (2011). Recurrent endometrial cancer. *Clinical Obstetrics and Gynecology*.

[4] Urick M. E., Bell D. W. (2019). Clinical actionability of molecular targets in endometrial cancer. *Nature Reviews Cancer*.

[5] Yen T. T., Wang T. L., Fader A. N., Shih I. M., Gaillard S. (2020). Molecular classification and emerging targeted therapy in endometrial cancer. *International Journal of Gynecological Pathology*.

[6] Wellington M., Koselny K., Sutterwala F. S., Krysan D. J. (2014). Candida albicans triggers NLRP3-mediated pyroptosis in macrophages. *Eukaryotic Cell*.

[7] Jorgensen I., Miao E. A. (2015). Pyroptotic cell death defends against intracellular pathogens. *Immunological Reviews*.

[8] Taabazuing C. Y., Okondo M. C., Bachovchin D. A. (2017). Pyroptosis and apoptosis pathways engage in bidirectional crosstalk in monocytes and macrophages. *Cell Chemical Biology*.

[9] Erkes D. A., Cai W., Sanchez I. M. (2020). Mutant BRAF and MEK inhibitors regulate the tumor immune microenvironment via pyroptosis. *Cancer Discovery*.

[10] Fang Y., Tian S., Pan Y. (2020). Pyroptosis: a new frontier in cancer. *Biomedicine & Pharmacotherapy*.

[11] Tan Y. F., Wang M., Chen Z. Y., Wang L., Liu X. H. (2020). Inhibition of BRD4 prevents proliferation and epithelial-mesenchymal transition in renal cell carcinoma via NLRP3 inflammasome-induced pyroptosis. *Cell Death & Disease*.

[12] Ye Y., Dai Q., Qi H. (2021). A novel defined pyroptosis-related gene signature for predicting the prognosis of ovarian cancer. *Cell Death Discovery*.

[13] Gao J., Qiu X., Xi G. (2018). Downregulation of GSDMD attenuates tumor proliferation via the intrinsic mitochondrial apoptotic pathway and inhibition of EGFR/Akt signaling and predicts a good prognosis in non-small cell lung cancer. *Oncology Reports*.

[14] Chen W., Zhang W., Zhou T., Cai J., Yu Z., Wu Z. (2021). A newly defined pyroptosis-related gene signature for the prognosis of bladder cancer. *International Journal of General Medicine*.

[15] Qian X., Tang J., Chu Y. (2021). A novel pyroptosis-related gene signature for prognostic prediction of head and neck squamous cell carcinoma. *International Journal of General Medicine*.

[16] Love M. I., Huber W., Anders S. (2014). Moderated estimation of fold change and dispersion for RNA-seq data with DESeq2. *Genome Biology*.

[17] Szklarczyk D., Gable A. L., Lyon D. (2019). STRING v11: protein-protein association networks with increased coverage, supporting functional discovery in genome-wide experimental datasets. *Nucleic Acids Research*.

[18] McAlpine J. N., Temkin S. M., Mackay H. J. (2016). Endometrial cancer: not your grandmother’s cancer. *Cancer*.

[19] Bendifallah S., Ballester M., Darai E. (2017). Endometrial cancer: predictive models and clinical impact. *Bulletin du Cancer*.

[20] Man S. M., Karki R., Kanneganti T. D. (2017). Molecular mechanisms and functions of pyroptosis, inflammatory caspases and inflammasomes in infectious diseases. *Immunological Reviews*.

[21] Wang M., Jiang S., Zhang Y., Li P., Wang K. (2019). The multifaceted roles of pyroptotic cell death pathways in cancer. *Cancers*.

[22] Zhang S., Li X., Zhang X., Zhang S., Tang C., Kuang W. (2021). The pyroptosis-related gene signature predicts the prognosis of hepatocellular carcinoma. *Frontiers in Molecular Biosciences*.

[23] Chen Y., Liao Y., Du Q. (2022). Roles of pyroptosis-related gene signature in prediction of endometrial cancer outcomes. *Frontiers in Medicine*.

[24] Li Q., Verma I. M. (2002). NF-*κ*B regulation in the immune system. *Nature Reviews Immunology*.

[25] Cartwright T., Perkins N. D., Wilson C. L. (2016). NFKB1: a suppressor of inflammation, ageing and cancer. *The FEBS Journal*.

[26] Kim G. C., Kwon H. K., Lee C. G. (2018). Upregulation of Ets1 expression by NFATc2 and NFKB1/RELA promotes breast cancer cell invasiveness. *Oncogene*.

[27] Yasuda Y., Li Z., Greenbaum D., Bogyo M., Weber E., Brömme D. (2004). Cathepsin V, a novel and potent elastolytic activity expressed in activated macrophages. *The Journal of Biological Chemistry*.

[28] Tolosa E., Li W., Yasuda Y. (2003). Cathepsin V is involved in the degradation of invariant chain in human thymus and is overexpressed in myasthenia gravis. *The Journal of Clinical Investigation*.

[29] Sereesongsaeng N., McDowell S. H., Burrows J. F., Scott C. J., Burden R. E. (2020). Cathepsin V suppresses GATA3 protein expression in luminal A breast cancer. *Breast Cancer Research*.

[30] Wang C. H., Wang L. K., Wu C. C. (2020). Cathepsin V mediates the tazarotene-induced gene 1-induced reduction in invasion in colorectal cancer cells. *Cell Biochemistry and Biophysics*.

[31] Penuela S., Harland L., Simek J., Laird D. W. (2014). Pannexin channels and their links to human disease. *The Biochemical Journal*.

[32] Bao L., Sun K., Zhang X. (2021). PANX1 is a potential prognostic biomarker associated with immune infiltration in pancreatic adenocarcinoma- a pan-cancer analysis. *Channels*.

[33] Jalaleddine N., El-Hajjar L., Dakik H. (2019). Pannexin 1 is associated with enhanced epithelial-to-mesenchymal transition in human patient breast cancer tissues and in breast cancer cell lines. *Cancers*.

[34] Yuan T. L., Cantley L. C. (2008). PI3K pathway alterations in cancer: variations on a theme. *Oncogene*.

[35] Konopka B., Paszko Z., Janiec-Jankowska A., Goluda M. (2002). Assessment of the quality and frequency of mutations occurrence in *PTEN* gene in endometrial carcinomas and hyperplasias. *Cancer Letters*.

[36] Kanamori Y., Kigawa J., Itamochi H. (2002). PTEN expression is associated with prognosis for patients with advanced endometrial carcinoma undergoing postoperative chemotherapy. *International Journal of Cancer*.

[37] Oliner J. D., Saiki A. Y., Caenepeel S. (2016). The role of MDM2 amplification and overexpression in tumorigenesis. *Cold Spring Harbor Perspectives in Medicine*.

[38] Mendoza M., Mandani G., Momand J. (2014). The MDM2 gene family. *Biomolecular Concepts*.

[39] Ambros R. A., Sheehan C. E., Kallakury B. V. (1996). MDM2 and p 53 protein expression in the histologic subtypes of endometrial carcinoma. *Modern Pathology*.

[40] Buchynska L. G., Nesina I. P., Kashuba E. V. (2007). Different trends of p 53, MDM2 and p 14 ARF expression patterns in endometrial adenocarcinomas versus hyperplasia. *Experimental Oncology*.

[41] Konishi M., Erdem S. S., Weissleder R., Lichtman A. H., McCarthy J. R., Libby P. (2015). Imaging granzyme B activity assesses immune-mediated myocarditis. *Circulation Research*.

[42] Hurkmans D. P., Basak E. A., Schepers N. (2020). Granzyme B is correlated with clinical outcome after PD-1 blockade in patients with stage IV non-small-cell lung cancer. *Journal for Immunotherapy of Cancer*.

[43] Salama P., Phillips M., Platell C., Iacopetta B. (2011). Low expression of granzyme B in colorectal cancer is associated with signs of early metastastic invasion. *Histopathology*.

[44] Kenney J. W., Moore C. E., Wang X., Proud C. G. (2014). Eukaryotic elongation factor 2 kinase, an unusual enzyme with multiple roles. *Advances in Biological Regulation*.

[45] Leprivier G., Rotblat B., Khan D., Jan E., Sorensen P. H. (2015). Stress-mediated translational control in cancer cells. *Biochimica et Biophysica Acta (BBA) - Gene Regulatory Mechanisms*.

[46] Jiang M., Qi L., Jin K. (2021). eEF2K as a novel metastatic and prognostic biomarker in gastric cancer patients. *Pathology-Research and Practice*.

[47] Wang Q., Wang Y., Ding J. (2020). A bioorthogonal system reveals antitumour immune function of pyroptosis. *Nature*.

[48] Garg A. D., Agostinis P. (2017). Cell death and immunity in cancer: from danger signals to mimicry of pathogen defense responses. *Immunological Reviews*.

