# Supplementary material for: A Novel Defined Pyroptosis-Related Gene Signature for Predicting the Prognosis of Endometrial Cancer
Source: Dis Markers. 2022 Dec 16;2022:7570494. doi: 10.1155/2022/7570494 (PMC9806687; doi:10.1155/2022/7570494)
Supplement: Supplementary 1 — Table S1: 133 pyroptosis-related genes retrieved from the GeneCards. [file 7570494.f1.docx]

Table S1. 133 pyroptosis-related genes retrieved from the GeneCards

| No. | Symbol | Description | Category 删除 |
| --- | --- | --- | --- |
| 1 | GSDMD | Gasdermin D | Protein Coding |
| 2 | GSDME | Gasdermin E | Protein Coding |
| 3 | NLRP3 | NLR Family Pyrin Domain Containing 3 | Protein Coding |
| 4 | CASP1 | Caspase 1 | Protein Coding |
| 5 | GSDMB | Gasdermin B | Protein Coding |
| 6 | CASP4 | Caspase 4 | Protein Coding |
| 7 | GSDMC | Gasdermin C | Protein Coding |
| 8 | GSDMA | Gasdermin A | Protein Coding |
| 9 | GZMB | Granzyme B | Protein Coding |
| 10 | NLRP1 | NLR Family Pyrin Domain Containing 1 | Protein Coding |
| 11 | GZMA | Granzyme A | Protein Coding |
| 12 | IL1B | Interleukin 1 Beta | Protein Coding |
| 13 | NLRC4 | NLR Family CARD Domain Containing 4 | Protein Coding |
| 14 | CASP5 | Caspase 5 | Protein Coding |
| 15 | CASP8 | Caspase 8 | Protein Coding |
| 16 | ZBP1 | Z-DNA Binding Protein 1 | Protein Coding |
| 17 | PYCARD | PYD And CARD Domain Containing | Protein Coding |
| 18 | AIM2 | Absent In Melanoma 2 | Protein Coding |
| 19 | NAIP | NLR Family Apoptosis Inhibitory Protein | Protein Coding |
| 20 | DHX9 | DExH-Box Helicase 9 | Protein Coding |
| 21 | NLRP9 | NLR Family Pyrin Domain Containing 9 | Protein Coding |
| 22 | APIP | APAF1 Interacting Protein | Protein Coding |
| 23 | HMGB1 | High Mobility Group Box 1 | Protein Coding |
| 24 | CASP3 | Caspase 3 | Protein Coding |
| 25 | FOXO3 | Forkhead Box O3 | Protein Coding |
| 26 | CASP6 | Caspase 6 | Protein Coding |
| 27 | IL18 | Interleukin 18 | Protein Coding |
| 28 | TXNIP | Thioredoxin Interacting Protein | Protein Coding |
| 29 | DDX3X | DEAD-Box Helicase 3 X-Linked | Protein Coding |
| 30 | GJA1 | Gap Junction Protein Alpha 1 | Protein Coding |
| 31 | GBP1 | Guanylate Binding Protein 1 | Protein Coding |
| 32 | CPTP | Ceramide-1-Phosphate Transfer Protein | Protein Coding |
| 33 | TP53 | Tumor Protein P53 | Protein Coding |
| 34 | NEK7 | NIMA Related Kinase 7 | Protein Coding |
| 35 | CRTAC1 | Cartilage Acidic Protein 1 | Protein Coding |
| 36 | NFE2L2 | Nuclear Factor, Erythroid 2 Like 2 | Protein Coding |
| 37 | AGER | Advanced Glycosylation End-Product Specific Receptor | Protein Coding |
| 38 | TET2 | Tet Methylcytosine Dioxygenase 2 | Protein Coding |
| 39 | CTSV | Cathepsin V | Protein Coding |
| 40 | APOE | Apolipoprotein E | Protein Coding |
| 41 | SDHB | Succinate Dehydrogenase Complex Iron Sulfur Subunit B | Protein Coding |
| 42 | EEF2K | Eukaryotic Elongation Factor 2 Kinase | Protein Coding |
| 43 | P2RX7 | Purinergic Receptor P2X 7 | Protein Coding |
| 44 | CD274 | CD274 Molecule | Protein Coding |
| 45 | FGF21 | Fibroblast Growth Factor 21 | Protein Coding |
| 46 | TFAM | Transcription Factor A, Mitochondrial | Protein Coding |
| 47 | CEBPB | CCAAT Enhancer Binding Protein Beta | Protein Coding |
| 48 | MALT1 | MALT1 Paracaspase | Protein Coding |
| 49 | STK4 | Serine/Threonine Kinase 4 | Protein Coding |
| 50 | MST1 | Macrophage Stimulating 1 | Protein Coding |
| 51 | PRDM1 | PR/SET Domain 1 | Protein Coding |
| 52 | PRF1 | Perforin 1 | Protein Coding |
| 53 | ELAVL1 | ELAV Like RNA Binding Protein 1 | Protein Coding |
| 54 | TREM2 | Triggering Receptor Expressed On Myeloid Cells 2 | Protein Coding |
| 55 | HDAC6 | Histone Deacetylase 6 | Protein Coding |
| 56 | SQSTM1 | Sequestosome 1 | Protein Coding |
| 57 | IRF3 | Interferon Regulatory Factor 3 | Protein Coding |
| 58 | STING1 | Stimulator Of Interferon Response CGAMP Interactor 1 | Protein Coding |
| 59 | PTEN | Phosphatase And Tensin Homolog | Protein Coding |
| 60 | PECAM1 | Platelet And Endothelial Cell Adhesion Molecule 1 | Protein Coding |
| 61 | METTL3 | Methyltransferase Like 3 | Protein Coding |
| 62 | CAMP | Cathelicidin Antimicrobial Peptide | Protein Coding |
| 63 | MRE11 | MRE11 Homolog, Double Strand Break Repair Nuclease | Protein Coding |
| 64 | PARP1 | Poly (ADP-Ribose) Polymerase 1 | Protein Coding |
| 65 | GBP5 | Guanylate Binding Protein 5 | Protein Coding |
| 66 | NR1H2 | Nuclear Receptor Subfamily 1 Group H Member 2 | Protein Coding |
| 67 | CTSG | Cathepsin G | Protein Coding |
| 68 | MKI67 | Marker Of Proliferation Ki-67 | Protein Coding |
| 69 | IL36G | Interleukin 36 Gamma | Protein Coding |
| 70 | IL36B | Interleukin 36 Beta | Protein Coding |
| 71 | PRTN3 | Proteinase 3 | Protein Coding |
| 72 | SERPINB1 | Serpin Family B Member 1 | Protein Coding |
| 73 | BNIP3 | BCL2 Interacting Protein 3 | Protein Coding |
| 74 | ANO6 | Anoctamin 6 | Protein Coding |
| 75 | FADD | Fas Associated Via Death Domain | Protein Coding |
| 76 | NLRP7 | NLR Family Pyrin Domain Containing 7 | Protein Coding |
| 77 | SESN2 | Sestrin 2 | Protein Coding |
| 78 | TNF | Tumor Necrosis Factor | Protein Coding |
| 79 | VIM | Vimentin | Protein Coding |
| 80 | CAPN1 | Calpain 1 | Protein Coding |
| 81 | JUN | Jun Proto-Oncogene, AP-1 Transcription Factor Subunit | Protein Coding |
| 82 | MEFV | MEFV Innate Immuity Regulator, Pyrin | Protein Coding |
| 83 | APOL1 | Apolipoprotein L1 | Protein Coding |
| 84 | ALK | ALK Receptor Tyrosine Kinase | Protein Coding |
| 85 | SIRT1 | Sirtuin 1 | Protein Coding |
| 86 | BIRC3 | Baculoviral IAP Repeat Containing 3 | Protein Coding |
| 87 | BIRC2 | Baculoviral IAP Repeat Containing 2 | Protein Coding |
| 88 | UBE2D2 | Ubiquitin Conjugating Enzyme E2 D2 | Protein Coding |
| 89 | LY96 | Lymphocyte Antigen 96 | Protein Coding |
| 90 | RIPK3 | Receptor Interacting Serine/Threonine Kinase 3 | Protein Coding |
| 91 | GLMN | Glomulin, FKBP Associated Protein | Protein Coding |
| 92 | CARD8 | Caspase Recruitment Domain Family Member 8 | Protein Coding |
| 93 | IRGM | Immunity Related GTPase M | Protein Coding |
| 94 | NLRP13 | NLR Family Pyrin Domain Containing 13 | Protein Coding |
| 95 | TUBB6 | Tubulin Beta 6 Class V | Protein Coding |
| 96 | NOS2 | Nitric Oxide Synthase 2 | Protein Coding |
| 97 | NOS1 | Nitric Oxide Synthase 1 | Protein Coding |
| 98 | PYDC2 | Pyrin Domain Containing 2 | Protein Coding |
| 99 | ACE2 | Angiotensin Converting Enzyme 2 | Protein Coding |
| 100 | EGFR | Epidermal Growth Factor Receptor | Protein Coding |
| 101 | AKT1 | AKT Serine/Threonine Kinase 1 | Protein Coding |
| 102 | TP63 | Tumor Protein P63 | Protein Coding |
| 103 | CASP9 | Caspase 9 | Protein Coding |
| 104 | ATF6 | Activating Transcription Factor 6 | Protein Coding |
| 105 | IRF1 | Interferon Regulatory Factor 1 | Protein Coding |
| 106 | IRF2 | Interferon Regulatory Factor 2 | Protein Coding |
| 107 | IFI16 | Interferon Gamma Inducible Protein 16 | Protein Coding |
| 108 | POP1 | POP1 Homolog, Ribonuclease P/MRP Subunit | Protein Coding |
| 109 | ORMDL3 | ORMDL Sphingolipid Biosynthesis Regulator 3 | Protein Coding |
| 110 | BTK | Bruton Tyrosine Kinase | Protein Coding |
| 111 | MDM2 | MDM2 Proto-Oncogene | Protein Coding |
| 112 | STAT3 | Signal Transducer And Activator Of Transcription 3 | Protein Coding |
| 113 | NFKB1 | Nuclear Factor Kappa B Subunit 1 | Protein Coding |
| 114 | TLR2 | Toll Like Receptor 2 | Protein Coding |
| 115 | BCL2 | BCL2 Apoptosis Regulator | Protein Coding |
| 116 | ANXA2 | Annexin A2 | Protein Coding |
| 117 | IL1RN | Interleukin 1 Receptor Antagonist | Protein Coding |
| 118 | BECN1 | Beclin 1 | Protein Coding |
| 119 | CD14 | CD14 Molecule | Protein Coding |
| 120 | IL13 | Interleukin 13 | Protein Coding |
| 121 | CHI3L1 | Chitinase 3 Like 1 | Protein Coding |
| 122 | HUWE1 | HECT, UBA And WWE Domain Containing E3 Ubiquitin Protein Ligase 1 | Protein Coding |
| 123 | GSTO1 | Glutathione S-Transferase Omega 1 | Protein Coding |
| 124 | PANX1 | Pannexin 1 | Protein Coding |
| 125 | LRPPRC | Leucine Rich Pentatricopeptide Repeat Containing | Protein Coding |
| 126 | CXCL8 | C-X-C Motif Chemokine Ligand 8 | Protein Coding |
| 127 | IL13RA2 | Interleukin 13 Receptor Subunit Alpha 2 | Protein Coding |
| 128 | IL32 | Interleukin 32 | Protein Coding |
| 129 | BST2 | Bone Marrow Stromal Cell Antigen 2 | Protein Coding |
| 130 | LYST | Lysosomal Trafficking Regulator | Protein Coding |
| 131 | GPER1 | G Protein-Coupled Estrogen Receptor 1 | Protein Coding |
| 132 | NCR1 | Natural Cytotoxicity Triggering Receptor 1 | Protein Coding |
| 133 | CLEC5A | C-Type Lectin Domain Containing 5A | Protein Coding |
