# Supplementary material for: A Novel Defined Pyroptosis-Related Gene Signature for Predicting the Prognosis of Endometrial Cancer
Source: Dis Markers. 2022 Dec 16;2022:7570494. doi: 10.1155/2022/7570494 (PMC9806687; doi:10.1155/2022/7570494)
Supplement: Supplementary 2 — Table S2: 97 pyroptosis-related DEGs in EC. [file 7570494.f2.docx]

Table S2. 97 pyroptosis-related DEGs in EC

| id | baseMean | log2FoldChange | lfcSE | stat | pvalue | padj |
| --- | --- | --- | --- | --- | --- | --- |
| MKI67 | 3207.472 | 3.583894 | 0.172099 | 20.82455 | 2.59E-96 | 1.48E-93 |
| ANO6 | 1377.058 | -1.81602 | 0.115088 | -15.7794 | 4.32E-56 | 3.02E-54 |
| CASP6 | 1019.008 | 1.512754 | 0.098132 | 15.41547 | 1.29E-53 | 8.04E-52 |
| CTSV | 2769.8 | 4.528857 | 0.309581 | 14.62897 | 1.84E-48 | 8.77E-47 |
| TXNIP | 11649.7 | -2.15655 | 0.16923 | -12.7433 | 3.40E-37 | 8.65E-36 |
| CASP3 | 1071.392 | 0.942854 | 0.081608 | 11.55343 | 7.09E-31 | 1.24E-29 |
| GSDMC | 32.58969 | 3.136723 | 0.306004 | 10.2506 | 1.18E-24 | 1.43E-23 |
| PECAM1 | 2558.702 | -1.59685 | 0.157894 | -10.1134 | 4.82E-24 | 5.58E-23 |
| GSDME | 291.7911 | -1.81039 | 0.185842 | -9.74157 | 2.00E-22 | 2.02E-21 |
| CAMP | 23.64255 | 4.735902 | 0.495572 | 9.556434 | 1.22E-21 | 1.16E-20 |
| PARP1 | 6289.442 | 0.993296 | 0.105004 | 9.459566 | 3.09E-21 | 2.87E-20 |
| IL1RN | 355.4952 | 2.518575 | 0.270295 | 9.317881 | 1.19E-20 | 1.07E-19 |
| NLRP1 | 675.5214 | -1.79016 | 0.199075 | -8.99242 | 2.42E-19 | 1.98E-18 |
| JUN | 8536.841 | -1.45062 | 0.161523 | -8.98088 | 2.69E-19 | 2.18E-18 |
| BCL2 | 751.1414 | -1.84262 | 0.207023 | -8.90055 | 5.56E-19 | 4.44E-18 |
| CTSG | 12.83114 | -3.78629 | 0.425424 | -8.90004 | 5.58E-19 | 4.45E-18 |
| SIRT1 | 659.1219 | -0.97035 | 0.109867 | -8.83197 | 1.03E-18 | 8.08E-18 |
| ELAVL1 | 3531.325 | 0.451168 | 0.055081 | 8.191046 | 2.59E-16 | 1.68E-15 |
| POP1 | 305.8879 | 0.808886 | 0.099503 | 8.129247 | 4.32E-16 | 2.76E-15 |
| NLRP7 | 40.2318 | 3.435629 | 0.425402 | 8.076184 | 6.68E-16 | 4.20E-15 |
| P2RX7 | 92.1303 | -1.64591 | 0.206781 | -7.9597 | 1.72E-15 | 1.04E-14 |
| TUBB6 | 1451.705 | -1.54624 | 0.196752 | -7.85884 | 3.88E-15 | 2.28E-14 |
| IL36G | 5.726103 | 4.612835 | 0.58801 | 7.844821 | 4.34E-15 | 2.54E-14 |
| ANXA2 | 22377.48 | 1.183613 | 0.151585 | 7.808226 | 5.80E-15 | 3.37E-14 |
| IL32 | 3023.399 | 1.930258 | 0.252373 | 7.648442 | 2.03E-14 | 1.13E-13 |
| BNIP3 | 2121.601 | 1.300648 | 0.17061 | 7.623499 | 2.47E-14 | 1.36E-13 |
| NEK7 | 1051.786 | -1.0709 | 0.142195 | -7.53123 | 5.03E-14 | 2.69E-13 |
| TNF | 152.7409 | 2.289678 | 0.313439 | 7.30503 | 2.77E-13 | 1.39E-12 |
| GBP5 | 640.1491 | 2.250903 | 0.312124 | 7.211579 | 5.53E-13 | 2.71E-12 |
| CLEC5A | 66.69247 | 1.605482 | 0.224542 | 7.150041 | 8.68E-13 | 4.18E-12 |
| CARD8 | 713.0506 | -0.63706 | 0.092377 | -6.89626 | 5.34E-12 | 2.39E-11 |
| PTEN | 2362.42 | -0.8501 | 0.127605 | -6.66195 | 2.70E-11 | 1.13E-10 |
| CAPN1 | 9795.77 | 0.740975 | 0.113147 | 6.548762 | 5.80E-11 | 2.36E-10 |
| FGF21 | 3.432958 | 4.092179 | 0.627304 | 6.523434 | 6.87E-11 | 2.77E-10 |
| LYST | 510.3853 | -0.92123 | 0.141614 | -6.50519 | 7.76E-11 | 3.12E-10 |
| IL18 | 377.8645 | 1.458023 | 0.224875 | 6.483707 | 8.95E-11 | 3.57E-10 |
| ALK | 40.49329 | 2.452619 | 0.387485 | 6.329579 | 2.46E-10 | 9.43E-10 |
| NFE2L2 | 2932.186 | -0.61652 | 0.097587 | -6.31771 | 2.65E-10 | 1.01E-09 |
| AKT1 | 5402.148 | 0.585784 | 0.092951 | 6.302084 | 2.94E-10 | 1.12E-09 |
| NFKB1 | 1313.829 | -0.66275 | 0.106817 | -6.20457 | 5.48E-10 | 2.03E-09 |
| CASP5 | 6.062919 | 2.052773 | 0.332621 | 6.17151 | 6.76E-10 | 2.49E-09 |
| CASP8 | 904.4333 | 0.607878 | 0.101923 | 5.964077 | 2.46E-09 | 8.57E-09 |
| ZBP1 | 78.45377 | 1.754207 | 0.294323 | 5.960136 | 2.52E-09 | 8.77E-09 |
| AIM2 | 40.68881 | 1.865788 | 0.321283 | 5.807296 | 6.35E-09 | 2.13E-08 |
| UBE2D2 | 4446.026 | 0.632416 | 0.110917 | 5.701714 | 1.19E-08 | 3.89E-08 |
| TP53 | 4405.399 | 0.753516 | 0.133062 | 5.662893 | 1.49E-08 | 4.83E-08 |
| DDX3X | 7399.692 | -0.48335 | 0.085696 | -5.64029 | 1.70E-08 | 5.47E-08 |
| IRF3 | 2418.978 | 0.642296 | 0.117645 | 5.459588 | 4.77E-08 | 1.47E-07 |
| IL13RA2 | 39.30804 | 2.100249 | 0.38537 | 5.449954 | 5.04E-08 | 1.55E-07 |
| TP63 | 77.42731 | 1.620123 | 0.300689 | 5.388045 | 7.12E-08 | 2.16E-07 |
| IFI16 | 2083.681 | -1.04867 | 0.195237 | -5.37129 | 7.82E-08 | 2.36E-07 |
| APOL1 | 8758.269 | 1.360159 | 0.256315 | 5.306593 | 1.12E-07 | 3.31E-07 |
| GSDMB | 597.6421 | 1.211733 | 0.230079 | 5.266582 | 1.39E-07 | 4.09E-07 |
| CPTP | 1461.785 | 0.607744 | 0.118258 | 5.139142 | 2.76E-07 | 7.87E-07 |
| PYCARD | 839.1072 | 1.06317 | 0.209196 | 5.082164 | 3.73E-07 | 1.05E-06 |
| ACE2 | 69.12816 | 1.353094 | 0.266344 | 5.08025 | 3.77E-07 | 1.06E-06 |
| SDHB | 1869.361 | 0.428409 | 0.085243 | 5.025733 | 5.02E-07 | 1.39E-06 |
| PRDM1 | 576.7739 | -0.99541 | 0.199008 | -5.00187 | 5.68E-07 | 1.57E-06 |
| BST2 | 11044.37 | 1.44082 | 0.289484 | 4.977192 | 6.45E-07 | 1.77E-06 |
| FOXO3 | 1926.16 | -0.44847 | 0.091582 | -4.89693 | 9.73E-07 | 2.62E-06 |
| TLR2 | 317.5655 | 0.796762 | 0.162839 | 4.892952 | 9.93E-07 | 2.67E-06 |
| LY96 | 71.40736 | -1.06985 | 0.219364 | -4.87706 | 1.08E-06 | 2.89E-06 |
| MST1 | 519.7646 | 1.022702 | 0.220303 | 4.64225 | 3.45E-06 | 8.75E-06 |
| TREM2 | 237.3495 | 1.076476 | 0.239636 | 4.492134 | 7.05E-06 | 1.73E-05 |
| GSDMD | 3127.459 | 0.595033 | 0.132868 | 4.478374 | 7.52E-06 | 1.84E-05 |
| EGFR | 1439.82 | -0.79541 | 0.180492 | -4.40688 | 1.05E-05 | 2.53E-05 |
| MEFV | 18.3736 | 1.092665 | 0.248276 | 4.401016 | 1.08E-05 | 2.59E-05 |
| GZMB | 303.2786 | 1.397481 | 0.328558 | 4.253382 | 2.11E-05 | 4.91E-05 |
| CHI3L1 | 2719.898 | 1.349087 | 0.318104 | 4.241032 | 2.22E-05 | 5.16E-05 |
| SERPINB1 | 2751.668 | 0.68587 | 0.163497 | 4.194998 | 2.73E-05 | 6.27E-05 |
| RIPK3 | 247.4988 | 0.85172 | 0.203371 | 4.188012 | 2.81E-05 | 6.46E-05 |
| MRE11 | 714.2831 | -0.38113 | 0.091727 | -4.15502 | 3.25E-05 | 7.41E-05 |
| STING1 | 2158.819 | -0.67432 | 0.166949 | -4.03906 | 5.37E-05 | 0.000119 |
| ORMDL3 | 3354.98 | 0.623331 | 0.159778 | 3.901241 | 9.57E-05 | 0.000207 |
| APOE | 8850.937 | 0.959863 | 0.264161 | 3.633625 | 0.000279 | 0.000569 |
| CD14 | 2142.629 | 0.744382 | 0.206488 | 3.604957 | 0.000312 | 0.000632 |
| EEF2K | 1577.547 | -0.33211 | 0.09229 | -3.5986 | 0.00032 | 0.000647 |
| CRTAC1 | 233.9778 | -1.71468 | 0.484357 | -3.54011 | 0.0004 | 0.000799 |
| SESN2 | 817.0999 | 0.468947 | 0.132671 | 3.534657 | 0.000408 | 0.000815 |
| GPER1 | 211.8375 | -0.87458 | 0.249213 | -3.50939 | 0.000449 | 0.00089 |
| HDAC6 | 1980.028 | 0.352479 | 0.102693 | 3.432366 | 0.000598 | 0.001167 |
| IRF2 | 1092.778 | -0.30441 | 0.089311 | -3.40843 | 0.000653 | 0.001267 |
| NLRP3 | 54.66927 | -0.59497 | 0.17475 | -3.40472 | 0.000662 | 0.001283 |
| MDM2 | 2552.214 | 0.458144 | 0.13562 | 3.378157 | 0.00073 | 0.001406 |
| CXCL8 | 443.1677 | 0.949728 | 0.308867 | 3.07488 | 0.002106 | 0.003822 |
| IL36B | 1.324454 | 2.781761 | 0.9356 | 2.973236 | 0.002947 | 0.005232 |
| BIRC2 | 1260.098 | -0.26623 | 0.090234 | -2.95039 | 0.003174 | 0.005607 |
| METTL3 | 1103.469 | 0.250287 | 0.086098 | 2.907001 | 0.003649 | 0.006381 |
| NLRP9 | 3.180489 | -0.87088 | 0.33226 | -2.62108 | 0.008765 | 0.014487 |
| FADD | 295.4959 | 0.30107 | 0.116775 | 2.578208 | 0.009931 | 0.016293 |
| GLMN | 235.6505 | 0.318354 | 0.123493 | 2.577907 | 0.00994 | 0.016306 |
| GSTO1 | 2571.173 | 0.347626 | 0.135766 | 2.560471 | 0.010453 | 0.017107 |
| PANX1 | 447.3426 | 0.253047 | 0.103673 | 2.440826 | 0.014654 | 0.023411 |
| IRGM | 1.608793 | 0.958417 | 0.400413 | 2.393574 | 0.016685 | 0.026439 |
| GSDMA | 22.62965 | 0.765249 | 0.327259 | 2.338359 | 0.019369 | 0.030327 |
| TET2 | 761.2592 | -0.2792 | 0.119414 | -2.33804 | 0.019385 | 0.030351 |
| DHX9 | 5594.17 | 0.173158 | 0.078462 | 2.206909 | 0.02732 | 0.041718 |
