# Supplementary material for: A Novel Defined Pyroptosis-Related Gene Signature for Predicting the Prognosis of Endometrial Cancer
Source: Dis Markers. 2022 Dec 16;2022:7570494. doi: 10.1155/2022/7570494 (PMC9806687; doi:10.1155/2022/7570494)
Supplement: Supplementary 3 — Table S3: GO enrichment analysis. [file 7570494.f3.docx]

Table S3. GO enrichment analysis

| ONTOLOGY | | ID | Description | GeneRatio | BgRatio | pvalue | p.adjust | qvalue | geneID | Count |
| --- | --- | --- | --- | --- | --- | --- | --- | --- | --- | --- |
| BP | GO:0070269 | | pyroptosis | 13/97 | 21/18723 | 1.65E-25 | 5.47E-22 | 3.20E-22 | GSDME/NLRP1/NLRP9/DHX9/GSDMD/CASP8/GSDMA/TREM2/GSDMB/GZMB/ZBP1/AIM2/GSDMC | 13 |
| BP | GO:0032611 | | interleukin-1 beta production | 17/97 | 110/18723 | 8.40E-21 | 9.27E-18 | 5.42E-18 | NLRP1/P2RX7/IFI16/CARD8/NLRP3/PANX1/GSDMD/CPTP/CASP8/SERPINB1/PYCARD/TREM2/MEFV/AIM2/GBP5/TNF/NLRP7 | 17 |
| BP | GO:0032651 | | regulation of interleukin-1 beta production | 17/97 | 110/18723 | 8.40E-21 | 9.27E-18 | 5.42E-18 | NLRP1/P2RX7/IFI16/CARD8/NLRP3/PANX1/GSDMD/CPTP/CASP8/SERPINB1/PYCARD/TREM2/MEFV/AIM2/GBP5/TNF/NLRP7 | 17 |
| BP | GO:0009615 | | response to virus | 24/97 | 367/18723 | 4.61E-20 | 3.17E-17 | 1.85E-17 | BCL2/GSDME/NLRP1/IFI16/LYST/NLRP9/STING1/NFKB1/CARD8/DDX3X/IRF2/BIRC2/DHX9/FADD/IRF3/TLR2/RIPK3/IRGM/PYCARD/BNIP3/BST2/ZBP1/AIM2/TNF | 24 |
| BP | GO:0071216 | | cellular response to biotic stimulus | 21/97 | 246/18723 | 5.38E-20 | 3.17E-17 | 1.85E-17 | CTSG/TXNIP/LY96/NFKB1/CARD8/NLRP3/AKT1/IRF3/CD14/TP53/TLR2/CXCL8/IRGM/PYCARD/TREM2/IL18/TNF/IL36B/NLRP7/IL36G/CAMP | 21 |
| BP | GO:0001819 | | positive regulation of cytokine production | 26/97 | 467/18723 | 5.74E-20 | 3.17E-17 | 1.85E-17 | NLRP1/P2RX7/LY96/IFI16/SIRT1/NLRP9/STING1/CARD8/NLRP3/DDX3X/DHX9/PANX1/FADD/GLMN/GSDMD/CASP8/IRF3/CD14/TLR2/PYCARD/TREM2/CHI3L1/IL18/AIM2/GBP5/TNF | 26 |
| BP | GO:0032612 | | interleukin-1 production | 17/97 | 128/18723 | 1.24E-19 | 5.15E-17 | 3.01E-17 | NLRP1/P2RX7/IFI16/CARD8/NLRP3/PANX1/GSDMD/CPTP/CASP8/SERPINB1/PYCARD/TREM2/MEFV/AIM2/GBP5/TNF/NLRP7 | 17 |
| BP | GO:0032652 | | regulation of interleukin-1 production | 17/97 | 128/18723 | 1.24E-19 | 5.15E-17 | 3.01E-17 | NLRP1/P2RX7/IFI16/CARD8/NLRP3/PANX1/GSDMD/CPTP/CASP8/SERPINB1/PYCARD/TREM2/MEFV/AIM2/GBP5/TNF/NLRP7 | 17 |
| BP | GO:0034612 | | response to tumor necrosis factor | 20/97 | 253/18723 | 2.09E-18 | 7.69E-16 | 4.50E-16 | GSDME/SIRT1/GPER1/NFKB1/CARD8/NFE2L2/FOXO3/BIRC2/DHX9/AKT1/CASP8/CD14/TP53/CASP3/CXCL8/PYCARD/CHI3L1/AIM2/TNF/CAMP | 20 |
| BP | GO:0032496 | | response to lipopolysaccharide | 22/97 | 343/18723 | 3.08E-18 | 9.99E-16 | 5.84E-16 | CTSG/JUN/LY96/NFKB1/CARD8/NLRP3/AKT1/CASP8/IRF3/CD14/TLR2/CASP3/CXCL8/IRGM/PYCARD/TREM2/IL18/TNF/IL36B/NLRP7/IL36G/CAMP | 22 |
| BP | GO:0071219 | | cellular response to molecule of bacterial origin | 19/97 | 221/18723 | 3.32E-18 | 9.99E-16 | 5.84E-16 | CTSG/LY96/NFKB1/CARD8/NLRP3/AKT1/IRF3/CD14/TLR2/CXCL8/IRGM/PYCARD/TREM2/IL18/TNF/IL36B/NLRP7/IL36G/CAMP | 19 |
| BP | GO:0002237 | | response to molecule of bacterial origin | 22/97 | 363/18723 | 1.03E-17 | 2.85E-15 | 1.66E-15 | CTSG/JUN/LY96/NFKB1/CARD8/NLRP3/AKT1/CASP8/IRF3/CD14/TLR2/CASP3/CXCL8/IRGM/PYCARD/TREM2/IL18/TNF/IL36B/NLRP7/IL36G/CAMP | 22 |
| CC | GO:0061702 | | inflammasome complex | 9/97 | 17/19550 | 2.93E-17 | 6.65E-15 | 5.40E-15 | NLRP1/NLRP9/CARD8/NLRP3/DDX3X/GSDMD/PYCARD/AIM2/CASP5 | 9 |
| BP | GO:0071222 | | cellular response to lipopolysaccharide | 18/97 | 209/18723 | 2.68E-17 | 6.82E-15 | 3.99E-15 | CTSG/LY96/NFKB1/CARD8/NLRP3/AKT1/IRF3/CD14/TLR2/CXCL8/IRGM/PYCARD/IL18/TNF/IL36B/NLRP7/IL36G/CAMP | 18 |
| BP | GO:0051607 | | defense response to virus | 19/97 | 265/18723 | 1.00E-16 | 2.22E-14 | 1.30E-14 | BCL2/NLRP1/IFI16/LYST/NLRP9/STING1/CARD8/IRF2/BIRC2/DHX9/FADD/IRF3/TLR2/RIPK3/PYCARD/BNIP3/BST2/ZBP1/AIM2 | 19 |
| BP | GO:0140546 | | defense response to symbiont | 19/97 | 265/18723 | 1.00E-16 | 2.22E-14 | 1.30E-14 | BCL2/NLRP1/IFI16/LYST/NLRP9/STING1/CARD8/IRF2/BIRC2/DHX9/FADD/IRF3/TLR2/RIPK3/PYCARD/BNIP3/BST2/ZBP1/AIM2 | 19 |
| BP | GO:0071356 | | cellular response to tumor necrosis factor | 18/97 | 229/18723 | 1.37E-16 | 2.83E-14 | 1.66E-14 | GSDME/SIRT1/GPER1/NFKB1/CARD8/NFE2L2/FOXO3/BIRC2/DHX9/AKT1/CASP8/TP53/CXCL8/PYCARD/CHI3L1/AIM2/TNF/CAMP | 18 |
| BP | GO:0019221 | | cytokine-mediated signaling pathway | 23/97 | 472/18723 | 2.03E-16 | 3.96E-14 | 2.32E-14 | CTSG/SIRT1/CARD8/FOXO3/BIRC2/METTL3/FADD/AKT1/CASP8/IRF3/TP53/CXCL8/IRGM/PYCARD/TREM2/IL18/ZBP1/AIM2/IL13RA2/TNF/IL1RN/IL36B/IL36G | 23 |
| BP | GO:0032731 | | positive regulation of interleukin-1 beta production | 12/97 | 62/18723 | 3.23E-16 | 5.95E-14 | 3.48E-14 | NLRP1/P2RX7/IFI16/CARD8/NLRP3/PANX1/GSDMD/CASP8/PYCARD/AIM2/GBP5/TNF | 12 |
| BP | GO:0032732 | | positive regulation of interleukin-1 production | 12/97 | 73/18723 | 2.63E-15 | 4.58E-13 | 2.68E-13 | NLRP1/P2RX7/IFI16/CARD8/NLRP3/PANX1/GSDMD/CASP8/PYCARD/AIM2/GBP5/TNF | 12 |
| BP | GO:2000116 | | regulation of cysteine-type endopeptidase activity | 17/97 | 235/18723 | 4.13E-15 | 6.84E-13 | 4.00E-13 | NLRP1/IFI16/SIRT1/GPER1/CARD8/NLRP3/DDX3X/BIRC2/FADD/MDM2/AKT1/CASP8/PYCARD/MEFV/TP63/AIM2/TNF | 17 |
| BP | GO:0052548 | | regulation of endopeptidase activity | 21/97 | 432/18723 | 5.19E-15 | 8.19E-13 | 4.79E-13 | NLRP1/IFI16/SIRT1/GPER1/CARD8/NLRP3/DDX3X/BIRC2/FADD/MDM2/AKT1/CASP8/SERPINB1/PYCARD/MEFV/ANXA2/BST2/TP63/AIM2/TNF/NLRP7 | 21 |
| BP | GO:0052547 | | regulation of peptidase activity | 21/97 | 461/18723 | 1.87E-14 | 2.81E-12 | 1.65E-12 | NLRP1/IFI16/SIRT1/GPER1/CARD8/NLRP3/DDX3X/BIRC2/FADD/MDM2/AKT1/CASP8/SERPINB1/PYCARD/MEFV/ANXA2/BST2/TP63/AIM2/TNF/NLRP7 | 21 |
| BP | GO:0010506 | | regulation of autophagy | 18/97 | 317/18723 | 4.05E-14 | 5.84E-12 | 3.41E-12 | BCL2/IFI16/SIRT1/STING1/FOXO3/HDAC6/SESN2/AKT1/CPTP/ORMDL3/CAPN1/TP53/CASP3/IRGM/PYCARD/TREM2/MEFV/BNIP3 | 18 |
| BP | GO:0140632 | | inflammasome complex assembly | 8/97 | 20/18723 | 4.63E-14 | 6.32E-12 | 3.69E-12 | NLRP1/CARD8/DDX3X/CPTP/TREM2/MEFV/AIM2/GBP5 | 8 |
| BP | GO:0071496 | | cellular response to external stimulus | 18/97 | 320/18723 | 4.77E-14 | 6.32E-12 | 3.69E-12 | BCL2/JUN/IFI16/SIRT1/EGFR/NFKB1/NFE2L2/FOXO3/FADD/MDM2/SESN2/AKT1/GSDMD/CASP8/TP53/BNIP3/CASP5/CTSV | 18 |
| BP | GO:0045862 | | positive regulation of proteolysis | 19/97 | 372/18723 | 4.98E-14 | 6.35E-12 | 3.71E-12 | NLRP1/IFI16/SIRT1/GPER1/PTEN/CARD8/NFE2L2/NLRP3/DDX3X/FADD/MDM2/AKT1/CASP8/APOE/PYCARD/TREM2/MEFV/AIM2/TNF | 19 |
| BP | GO:0050727 | | regulation of inflammatory response | 19/97 | 386/18723 | 9.66E-14 | 1.19E-11 | 6.93E-12 | NLRP1/GPER1/EGFR/STING1/NFKB1/NLRP3/BIRC2/DHX9/IRF3/TLR2/APOE/PYCARD/TREM2/MEFV/ACE2/IL18/ZBP1/CASP5/TNF | 19 |
| BP | GO:0097193 | | intrinsic apoptotic signaling pathway | 17/97 | 288/18723 | 1.18E-13 | 1.40E-11 | 8.16E-12 | BCL2/GSDME/IFI16/SIRT1/NFE2L2/DDX3X/MDM2/AKT1/TP53/RIPK3/CASP3/PARP1/PYCARD/TREM2/BNIP3/TP63/TNF | 17 |
| BP | GO:0045088 | | regulation of innate immune response | 15/97 | 218/18723 | 4.03E-13 | 4.60E-11 | 2.69E-11 | IFI16/STING1/BIRC2/DHX9/METTL3/FADD/CASP8/IRF3/IRGM/APOE/PYCARD/TREM2/ZBP1/AIM2/GBP5 | 15 |
| BP | GO:0038061 | | NIK/NF-kappaB signaling | 13/97 | 143/18723 | 4.66E-13 | 5.15E-11 | 3.01E-11 | EGFR/NLRP3/DDX3X/BIRC2/AKT1/CD14/TLR2/RIPK3/TREM2/CHI3L1/IL18/TNF/ALK | 13 |
| BP | GO:0000302 | | response to reactive oxygen species | 15/97 | 222/18723 | 5.25E-13 | 5.61E-11 | 3.28E-11 | TXNIP/BCL2/JUN/SIRT1/EGFR/NFE2L2/FOXO3/HDAC6/MDM2/SESN2/AKT1/RIPK3/CASP3/APOE/BNIP3 | 15 |
| BP | GO:2001056 | | positive regulation of cysteine-type endopeptidase activity | 13/97 | 148/18723 | 7.27E-13 | 7.48E-11 | 4.38E-11 | NLRP1/IFI16/SIRT1/GPER1/CARD8/NLRP3/DDX3X/FADD/CASP8/PYCARD/MEFV/AIM2/TNF | 13 |
| BP | GO:0070265 | | necrotic cell death | 10/97 | 62/18723 | 7.45E-13 | 7.48E-11 | 4.38E-11 | GSDME/BIRC2/FADD/CASP8/IRF3/TP53/RIPK3/BNIP3/ZBP1/TNF | 10 |
| BP | GO:0002831 | | regulation of response to biotic stimulus | 17/97 | 327/18723 | 9.24E-13 | 9.00E-11 | 5.26E-11 | LY96/IFI16/STING1/CARD8/BIRC2/DHX9/METTL3/FADD/CASP8/IRF3/IRGM/APOE/PYCARD/TREM2/ZBP1/AIM2/GBP5 | 17 |
| BP | GO:0062197 | | cellular response to chemical stress | 17/97 | 337/18723 | 1.50E-12 | 1.42E-10 | 8.29E-11 | BCL2/JUN/SIRT1/EGFR/NFE2L2/DDX3X/FOXO3/HDAC6/MDM2/SESN2/AKT1/TP53/RIPK3/CASP3/PARP1/TREM2/BNIP3 | 17 |
| BP | GO:2001233 | | regulation of apoptotic signaling pathway | 17/97 | 356/18723 | 3.60E-12 | 3.32E-10 | 1.94E-10 | BCL2/GSDME/SIRT1/GPER1/PTEN/NFE2L2/DDX3X/FADD/MDM2/AKT1/TP53/RIPK3/PARP1/PYCARD/TREM2/TP63/TNF | 17 |
| BP | GO:0044546 | | NLRP3 inflammasome complex assembly | 7/97 | 19/18723 | 3.85E-12 | 3.45E-10 | 2.02E-10 | CARD8/DDX3X/CPTP/TREM2/MEFV/AIM2/GBP5 | 7 |
| BP | GO:0070997 | | neuron death | 17/97 | 361/18723 | 4.50E-12 | 3.92E-10 | 2.29E-10 | BCL2/NLRP1/JUN/SIRT1/FOXO3/FADD/AKT1/CASP8/TP53/CASP3/APOE/PARP1/TREM2/BNIP3/TP63/TNF/FGF21 | 17 |
| BP | GO:0031331 | | positive regulation of cellular catabolic process | 18/97 | 427/18723 | 6.28E-12 | 5.20E-10 | 3.04E-10 | P2RX7/SIRT1/PTEN/STING1/NFE2L2/FOXO3/METTL3/MDM2/SESN2/AKT1/ORMDL3/IRGM/APOE/TREM2/MEFV/BNIP3/TNF/FGF21 | 18 |
| BP | GO:0032103 | | positive regulation of response to external stimulus | 18/97 | 427/18723 | 6.28E-12 | 5.20E-10 | 3.04E-10 | ANO6/LY96/IFI16/EGFR/STING1/DHX9/FADD/IRF3/TLR2/CXCL8/IRGM/PYCARD/TREM2/IL18/ZBP1/AIM2/GBP5/TNF | 18 |
| BP | GO:0010950 | | positive regulation of endopeptidase activity | 13/97 | 179/18723 | 8.33E-12 | 6.73E-10 | 3.94E-10 | NLRP1/IFI16/SIRT1/GPER1/CARD8/NLRP3/DDX3X/FADD/CASP8/PYCARD/MEFV/AIM2/TNF | 13 |
| BP | GO:0006979 | | response to oxidative stress | 18/97 | 446/18723 | 1.29E-11 | 1.02E-09 | 5.94E-10 | TXNIP/BCL2/JUN/SIRT1/EGFR/NFE2L2/FOXO3/HDAC6/MDM2/SESN2/AKT1/TP53/RIPK3/CASP3/APOE/PARP1/TREM2/BNIP3 | 18 |
| BP | GO:0031349 | | positive regulation of defense response | 15/97 | 278/18723 | 1.34E-11 | 1.02E-09 | 5.94E-10 | IFI16/EGFR/STING1/DHX9/FADD/IRF3/TLR2/IRGM/PYCARD/TREM2/IL18/ZBP1/AIM2/GBP5/TNF | 15 |
| BP | GO:0071214 | | cellular response to abiotic stimulus | 16/97 | 331/18723 | 1.38E-11 | 1.02E-09 | 5.94E-10 | IFI16/SIRT1/PTEN/EGFR/NFKB1/DDX3X/METTL3/FADD/MDM2/AKT1/CASP8/TP53/CASP3/PARP1/BNIP3/CASP5 | 16 |
| BP | GO:0104004 | | cellular response to environmental stimulus | 16/97 | 331/18723 | 1.38E-11 | 1.02E-09 | 5.94E-10 | IFI16/SIRT1/PTEN/EGFR/NFKB1/DDX3X/METTL3/FADD/MDM2/AKT1/CASP8/TP53/CASP3/PARP1/BNIP3/CASP5 | 16 |
| BP | GO:0034599 | | cellular response to oxidative stress | 15/97 | 288/18723 | 2.22E-11 | 1.60E-09 | 9.34E-10 | BCL2/JUN/SIRT1/EGFR/NFE2L2/FOXO3/HDAC6/MDM2/SESN2/AKT1/TP53/RIPK3/PARP1/TREM2/BNIP3 | 15 |
| BP | GO:0010952 | | positive regulation of peptidase activity | 13/97 | 197/18723 | 2.80E-11 | 1.97E-09 | 1.15E-09 | NLRP1/IFI16/SIRT1/GPER1/CARD8/NLRP3/DDX3X/FADD/CASP8/PYCARD/MEFV/AIM2/TNF | 13 |
| BP | GO:2000377 | | regulation of reactive oxygen species metabolic process | 12/97 | 157/18723 | 3.05E-11 | 2.11E-09 | 1.23E-09 | BCL2/EGFR/NFE2L2/FOXO3/BIRC2/HDAC6/AKT1/TP53/RIPK3/BNIP3/ACE2/TNF | 12 |
| BP | GO:0060759 | | regulation of response to cytokine stimulus | 12/97 | 162/18723 | 4.41E-11 | 2.98E-09 | 1.74E-09 | CARD8/DHX9/METTL3/FADD/CASP8/IRF3/TLR2/IRGM/PYCARD/TREM2/ZBP1/IL1RN | 12 |
| BP | GO:2001242 | | regulation of intrinsic apoptotic signaling pathway | 12/97 | 164/18723 | 5.10E-11 | 3.38E-09 | 1.97E-09 | BCL2/GSDME/SIRT1/NFE2L2/DDX3X/MDM2/AKT1/TP53/RIPK3/PARP1/PYCARD/TREM2 | 12 |
| BP | GO:0043281 | | regulation of cysteine-type endopeptidase activity involved in apoptotic process | 13/97 | 209/18723 | 5.88E-11 | 3.82E-09 | 2.23E-09 | NLRP1/SIRT1/GPER1/CARD8/NLRP3/DDX3X/BIRC2/MDM2/AKT1/CASP8/PYCARD/TP63/TNF | 13 |
| BP | GO:0009896 | | positive regulation of catabolic process | 18/97 | 492/18723 | 6.48E-11 | 4.13E-09 | 2.41E-09 | P2RX7/SIRT1/PTEN/STING1/NFE2L2/FOXO3/METTL3/MDM2/SESN2/AKT1/ORMDL3/IRGM/APOE/TREM2/MEFV/BNIP3/TNF/FGF21 | 18 |
| BP | GO:0097300 | | programmed necrotic cell death | 8/97 | 47/18723 | 1.03E-10 | 6.44E-09 | 3.76E-09 | BIRC2/FADD/CASP8/IRF3/TP53/RIPK3/ZBP1/TNF | 8 |
| BP | GO:0048545 | | response to steroid hormone | 15/97 | 339/18723 | 2.19E-10 | 1.34E-08 | 7.85E-09 | TXNIP/BCL2/SIRT1/GPER1/EGFR/FOXO3/HDAC6/MDM2/TLR2/CASP3/PARP1/TP63/TNF/IL1RN/CTSV | 15 |
| BP | GO:0042542 | | response to hydrogen peroxide | 11/97 | 146/18723 | 2.48E-10 | 1.50E-08 | 8.75E-09 | TXNIP/BCL2/JUN/SIRT1/NFE2L2/FOXO3/HDAC6/MDM2/RIPK3/CASP3/BNIP3 | 11 |
| BP | GO:1900225 | | regulation of NLRP3 inflammasome complex assembly | 6/97 | 18/18723 | 2.92E-10 | 1.73E-08 | 1.01E-08 | CARD8/DDX3X/CPTP/TREM2/MEFV/GBP5 | 6 |
| BP | GO:0072593 | | reactive oxygen species metabolic process | 13/97 | 239/18723 | 3.11E-10 | 1.81E-08 | 1.06E-08 | BCL2/EGFR/NFE2L2/FOXO3/BIRC2/HDAC6/SESN2/AKT1/TP53/RIPK3/BNIP3/ACE2/TNF | 13 |
| BP | GO:0051402 | | neuron apoptotic process | 13/97 | 246/18723 | 4.44E-10 | 2.54E-08 | 1.48E-08 | BCL2/NLRP1/JUN/FOXO3/FADD/TP53/CASP3/APOE/PARP1/TREM2/BNIP3/TP63/TNF | 13 |
| BP | GO:0036294 | | cellular response to decreased oxygen levels | 11/97 | 161/18723 | 7.09E-10 | 3.98E-08 | 2.33E-08 | BCL2/SIRT1/PTEN/NFE2L2/FOXO3/EEF2K/MDM2/AKT1/TP53/TREM2/BNIP3 | 11 |
| BP | GO:0031663 | | lipopolysaccharide-mediated signaling pathway | 8/97 | 60/18723 | 7.93E-10 | 4.38E-08 | 2.56E-08 | LY96/CARD8/AKT1/IRF3/CD14/TLR2/IL18/TNF | 8 |
| BP | GO:2001235 | | positive regulation of apoptotic signaling pathway | 10/97 | 126/18723 | 1.02E-09 | 5.53E-08 | 3.23E-08 | GSDME/SIRT1/GPER1/PTEN/FADD/TP53/RIPK3/PYCARD/TP63/TNF | 10 |
| BP | GO:0002833 | | positive regulation of response to biotic stimulus | 11/97 | 168/18723 | 1.12E-09 | 5.96E-08 | 3.49E-08 | LY96/IFI16/STING1/DHX9/FADD/IRF3/IRGM/PYCARD/ZBP1/AIM2/GBP5 | 11 |
| BP | GO:0036293 | | response to decreased oxygen levels | 14/97 | 322/18723 | 1.16E-09 | 6.08E-08 | 3.56E-08 | BCL2/SIRT1/PTEN/NFE2L2/FOXO3/EEF2K/BIRC2/MDM2/AKT1/TP53/TLR2/CASP3/TREM2/BNIP3 | 14 |
| BP | GO:0070266 | | necroptotic process | 7/97 | 40/18723 | 1.30E-09 | 6.75E-08 | 3.95E-08 | BIRC2/FADD/CASP8/TP53/RIPK3/ZBP1/TNF | 7 |
| BP | GO:0045089 | | positive regulation of innate immune response | 10/97 | 131/18723 | 1.49E-09 | 7.61E-08 | 4.45E-08 | IFI16/STING1/DHX9/FADD/IRF3/IRGM/PYCARD/ZBP1/AIM2/GBP5 | 10 |
| CC | GO:0045121 | | membrane raft | 14/97 | 335/19550 | 1.11E-09 | 8.43E-08 | 6.84E-08 | PECAM1/EGFR/BIRC2/FADD/HDAC6/CASP8/CD14/TLR2/CASP3/TREM2/ANXA2/ACE2/BST2/TNF | 14 |
| CC | GO:0098857 | | membrane microdomain | 14/97 | 335/19550 | 1.11E-09 | 8.43E-08 | 6.84E-08 | PECAM1/EGFR/BIRC2/FADD/HDAC6/CASP8/CD14/TLR2/CASP3/TREM2/ANXA2/ACE2/BST2/TNF | 14 |
| BP | GO:0010939 | | regulation of necrotic cell death | 7/97 | 42/18723 | 1.87E-09 | 9.25E-08 | 5.41E-08 | BIRC2/FADD/CASP8/TP53/RIPK3/BNIP3/ZBP1 | 7 |
| BP | GO:0032691 | | negative regulation of interleukin-1 beta production | 7/97 | 42/18723 | 1.87E-09 | 9.25E-08 | 5.41E-08 | CARD8/NLRP3/CPTP/SERPINB1/TREM2/MEFV/NLRP7 | 7 |
| BP | GO:0071453 | | cellular response to oxygen levels | 11/97 | 177/18723 | 1.94E-09 | 9.46E-08 | 5.53E-08 | BCL2/SIRT1/PTEN/NFE2L2/FOXO3/EEF2K/MDM2/AKT1/TP53/TREM2/BNIP3 | 11 |
| BP | GO:0007249 | | I-kappaB kinase/NF-kappaB signaling | 13/97 | 281/18723 | 2.25E-09 | 1.08E-07 | 6.32E-08 | SIRT1/CARD8/BIRC2/FADD/AKT1/CASP8/IRF3/TLR2/RIPK3/PYCARD/TREM2/BST2/TNF | 13 |
| BP | GO:0031667 | | response to nutrient levels | 16/97 | 474/18723 | 2.67E-09 | 1.26E-07 | 7.39E-08 | BCL2/JUN/IFI16/SIRT1/PTEN/EGFR/NFE2L2/FOXO3/MDM2/SESN2/AKT1/TP53/APOE/BNIP3/FGF21/CTSV | 16 |
| BP | GO:0070482 | | response to oxygen levels | 14/97 | 347/18723 | 3.03E-09 | 1.41E-07 | 8.24E-08 | BCL2/SIRT1/PTEN/NFE2L2/FOXO3/EEF2K/BIRC2/MDM2/AKT1/TP53/TLR2/CASP3/TREM2/BNIP3 | 14 |
| BP | GO:0016241 | | regulation of macroautophagy | 10/97 | 141/18723 | 3.06E-09 | 1.41E-07 | 8.24E-08 | SIRT1/STING1/HDAC6/SESN2/AKT1/CAPN1/TP53/CASP3/IRGM/BNIP3 | 10 |
| BP | GO:0071887 | | leukocyte apoptotic process | 9/97 | 106/18723 | 4.00E-09 | 1.82E-07 | 1.06E-07 | SIRT1/PTEN/FADD/AKT1/ORMDL3/IRF3/TP53/RIPK3/CASP3 | 9 |
| BP | GO:0001959 | | regulation of cytokine-mediated signaling pathway | 10/97 | 150/18723 | 5.58E-09 | 2.50E-07 | 1.46E-07 | CARD8/METTL3/FADD/CASP8/IRF3/IRGM/PYCARD/TREM2/ZBP1/IL1RN | 10 |
| BP | GO:0032692 | | negative regulation of interleukin-1 production | 7/97 | 49/18723 | 5.78E-09 | 2.55E-07 | 1.49E-07 | CARD8/NLRP3/CPTP/SERPINB1/TREM2/MEFV/NLRP7 | 7 |
| BP | GO:0071456 | | cellular response to hypoxia | 10/97 | 151/18723 | 5.95E-09 | 2.60E-07 | 1.52E-07 | BCL2/SIRT1/PTEN/NFE2L2/FOXO3/MDM2/AKT1/TP53/TREM2/BNIP3 | 10 |
| BP | GO:1901222 | | regulation of NIK/NF-kappaB signaling | 9/97 | 112/18723 | 6.52E-09 | 2.77E-07 | 1.62E-07 | EGFR/NLRP3/DDX3X/BIRC2/CD14/TLR2/TREM2/IL18/TNF | 9 |
| BP | GO:0001666 | | response to hypoxia | 13/97 | 307/18723 | 6.53E-09 | 2.77E-07 | 1.62E-07 | BCL2/SIRT1/PTEN/NFE2L2/FOXO3/BIRC2/MDM2/AKT1/TP53/TLR2/CASP3/TREM2/BNIP3 | 13 |
| BP | GO:0062098 | | regulation of programmed necrotic cell death | 6/97 | 29/18723 | 7.13E-09 | 2.99E-07 | 1.75E-07 | BIRC2/FADD/CASP8/TP53/RIPK3/ZBP1 | 6 |
| BP | GO:0010038 | | response to metal ion | 14/97 | 373/18723 | 7.62E-09 | 3.13E-07 | 1.83E-07 | TXNIP/BCL2/JUN/PTEN/EGFR/NFE2L2/EEF2K/MDM2/AKT1/CASP8/CD14/CASP3/PARP1/BNIP3 | 14 |
| BP | GO:0034614 | | cellular response to reactive oxygen species | 10/97 | 155/18723 | 7.66E-09 | 3.13E-07 | 1.83E-07 | JUN/SIRT1/EGFR/NFE2L2/FOXO3/HDAC6/MDM2/AKT1/RIPK3/BNIP3 | 10 |
| BP | GO:0051091 | | positive regulation of DNA-binding transcription factor activity | 12/97 | 260/18723 | 1.00E-08 | 4.04E-07 | 2.36E-07 | PTEN/STING1/NLRP3/DHX9/AKT1/TLR2/RIPK3/PYCARD/IL18/AIM2/TNF/ALK | 12 |
| BP | GO:1901214 | | regulation of neuron death | 13/97 | 319/18723 | 1.03E-08 | 4.12E-07 | 2.41E-07 | BCL2/JUN/SIRT1/FOXO3/AKT1/CASP8/TP53/CASP3/APOE/PARP1/TREM2/TNF/FGF21 | 13 |
| BP | GO:0072331 | | signal transduction by p53 class mediator | 10/97 | 163/18723 | 1.24E-08 | 4.91E-07 | 2.87E-07 | BCL2/IFI16/SIRT1/FOXO3/MDM2/SESN2/AKT1/TP53/PYCARD/TP63 | 10 |
| BP | GO:0060760 | | positive regulation of response to cytokine stimulus | 7/97 | 57/18723 | 1.72E-08 | 6.71E-07 | 3.92E-07 | DHX9/FADD/IRF3/TLR2/IRGM/TREM2/ZBP1 | 7 |
| BP | GO:0097191 | | extrinsic apoptotic signaling pathway | 11/97 | 219/18723 | 1.80E-08 | 6.93E-07 | 4.05E-07 | BCL2/P2RX7/GPER1/PTEN/DDX3X/FOXO3/FADD/AKT1/CASP8/PYCARD/TNF | 11 |
| BP | GO:0002931 | | response to ischemia | 7/97 | 58/18723 | 1.95E-08 | 7.42E-07 | 4.34E-07 | BCL2/P2RX7/EEF2K/PANX1/AKT1/TP53/TREM2 | 7 |
| BP | GO:0001836 | | release of cytochrome c from mitochondria | 7/97 | 59/18723 | 2.20E-08 | 8.29E-07 | 4.85E-07 | BCL2/JUN/GPER1/AKT1/TP53/PYCARD/BNIP3 | 7 |
| BP | GO:0043280 | | positive regulation of cysteine-type endopeptidase activity involved in apoptotic process | 9/97 | 129/18723 | 2.26E-08 | 8.43E-07 | 4.93E-07 | NLRP1/SIRT1/GPER1/CARD8/NLRP3/DDX3X/CASP8/PYCARD/TNF | 9 |
| BP | GO:0016485 | | protein processing | 11/97 | 225/18723 | 2.38E-08 | 8.75E-07 | 5.11E-07 | CTSG/IFI16/CARD8/FADD/MDM2/CASP8/CASP3/PARP1/PYCARD/ACE2/NLRP7 | 11 |
| MF | GO:0002020 | | protease binding | 10/97 | 135/18368 | 2.40E-09 | 9.87E-07 | 7.68E-07 | BCL2/PTEN/PANX1/FADD/TP53/CASP3/PYCARD/ANXA2/TNF/NLRP7 | 10 |
| BP | GO:0016032 | | viral process | 14/97 | 415/18723 | 2.92E-08 | 1.06E-06 | 6.21E-07 | BCL2/ANO6/JUN/IFI16/EGFR/DDX3X/DHX9/TP53/CXCL8/APOE/ACE2/BST2/CLEC5A/TNF | 14 |
| BP | GO:0042742 | | defense response to bacterium | 13/97 | 350/18723 | 3.09E-08 | 1.11E-06 | 6.51E-07 | CTSG/NLRP1/LYST/GSDMD/GSDMA/TLR2/IRGM/PYCARD/TREM2/GSDMB/TNF/GSDMC/CAMP | 13 |
| BP | GO:0045861 | | negative regulation of proteolysis | 13/97 | 351/18723 | 3.20E-08 | 1.14E-06 | 6.66E-07 | IFI16/CARD8/DDX3X/BIRC2/HDAC6/MDM2/AKT1/SERPINB1/TP53/ANXA2/BST2/TNF/NLRP7 | 13 |
| BP | GO:0036473 | | cell death in response to oxidative stress | 8/97 | 95/18723 | 3.25E-08 | 1.15E-06 | 6.70E-07 | BCL2/SIRT1/NFE2L2/FOXO3/HDAC6/AKT1/PARP1/TREM2 | 8 |
| BP | GO:1902882 | | regulation of response to oxidative stress | 8/97 | 98/18723 | 4.15E-08 | 1.44E-06 | 8.40E-07 | SIRT1/NFE2L2/FOXO3/HDAC6/SESN2/AKT1/PARP1/TREM2 | 8 |
| BP | GO:1901653 | | cellular response to peptide | 13/97 | 359/18723 | 4.16E-08 | 1.44E-06 | 8.40E-07 | SIRT1/GPER1/PTEN/NFKB1/NFE2L2/FOXO3/EEF2K/MDM2/AKT1/TP53/PARP1/TREM2/FGF21 | 13 |
| BP | GO:0043254 | | regulation of protein-containing complex assembly | 14/97 | 428/18723 | 4.28E-08 | 1.46E-06 | 8.55E-07 | CARD8/DDX3X/BIRC2/HDAC6/CPTP/TP53/IRGM/APOE/PARP1/PYCARD/TREM2/MEFV/GBP5/TNF | 14 |
| BP | GO:0033209 | | tumor necrosis factor-mediated signaling pathway | 8/97 | 99/18723 | 4.50E-08 | 1.52E-06 | 8.89E-07 | CARD8/FOXO3/BIRC2/CASP8/TP53/PYCARD/AIM2/TNF | 8 |
| BP | GO:0045833 | | negative regulation of lipid metabolic process | 8/97 | 102/18723 | 5.69E-08 | 1.90E-06 | 1.11E-06 | SIRT1/GPER1/NFKB1/AKT1/ORMDL3/APOE/TNF/ALK | 8 |
| BP | GO:0031668 | | cellular response to extracellular stimulus | 11/97 | 246/18723 | 5.93E-08 | 1.96E-06 | 1.15E-06 | BCL2/JUN/IFI16/SIRT1/NFE2L2/FOXO3/MDM2/SESN2/GSDMD/TP53/CTSV | 11 |
| BP | GO:0051090 | | regulation of DNA-binding transcription factor activity | 14/97 | 440/18723 | 6.03E-08 | 1.98E-06 | 1.16E-06 | SIRT1/PTEN/STING1/CARD8/NLRP3/DHX9/AKT1/TLR2/RIPK3/PYCARD/IL18/AIM2/TNF/ALK | 14 |
| BP | GO:1901224 | | positive regulation of NIK/NF-kappaB signaling | 7/97 | 69/18723 | 6.68E-08 | 2.17E-06 | 1.27E-06 | EGFR/DDX3X/CD14/TLR2/TREM2/IL18/TNF | 7 |
| MF | GO:0097153 | | cysteine-type endopeptidase activity involved in apoptotic process | 5/97 | 15/18368 | 1.07E-08 | 2.19E-06 | 1.71E-06 | CASP8/CASP3/PYCARD/CASP6/CASP5 | 5 |
| BP | GO:0010951 | | negative regulation of endopeptidase activity | 11/97 | 252/18723 | 7.57E-08 | 2.44E-06 | 1.42E-06 | IFI16/CARD8/DDX3X/BIRC2/MDM2/AKT1/SERPINB1/ANXA2/BST2/TNF/NLRP7 | 11 |
| MF | GO:0001530 | | lipopolysaccharide binding | 6/97 | 33/18368 | 1.83E-08 | 2.51E-06 | 1.95E-06 | P2RX7/LY96/CD14/TLR2/TREM2/CAMP | 6 |
| BP | GO:0009411 | | response to UV | 9/97 | 149/18723 | 7.91E-08 | 2.52E-06 | 1.47E-06 | BCL2/SIRT1/EGFR/METTL3/MDM2/AKT1/TP53/CASP3/PARP1 | 9 |
| BP | GO:0008637 | | apoptotic mitochondrial changes | 8/97 | 107/18723 | 8.28E-08 | 2.60E-06 | 1.52E-06 | BCL2/JUN/GPER1/AKT1/TP53/PYCARD/BNIP3/GZMB | 8 |
| BP | GO:0030099 | | myeloid cell differentiation | 13/97 | 381/18723 | 8.33E-08 | 2.60E-06 | 1.52E-06 | JUN/IFI16/SIRT1/FOXO3/TET2/FADD/CASP8/TLR2/CASP3/PARP1/TREM2/ANXA2/TNF | 13 |
| BP | GO:0042771 | | intrinsic apoptotic signaling pathway in response to DNA damage by p53 class mediator | 6/97 | 43/18723 | 8.64E-08 | 2.67E-06 | 1.56E-06 | BCL2/IFI16/SIRT1/TP53/PYCARD/TP63 | 6 |
| BP | GO:0051092 | | positive regulation of NF-kappaB transcription factor activity | 9/97 | 152/18723 | 9.39E-08 | 2.88E-06 | 1.68E-06 | NLRP3/DHX9/TLR2/RIPK3/PYCARD/IL18/AIM2/TNF/ALK | 9 |
| BP | GO:1903201 | | regulation of oxidative stress-induced cell death | 7/97 | 74/18723 | 1.09E-07 | 3.31E-06 | 1.94E-06 | SIRT1/NFE2L2/FOXO3/HDAC6/AKT1/PARP1/TREM2 | 7 |
| BP | GO:0010466 | | negative regulation of peptidase activity | 11/97 | 262/18723 | 1.12E-07 | 3.38E-06 | 1.98E-06 | IFI16/CARD8/DDX3X/BIRC2/MDM2/AKT1/SERPINB1/ANXA2/BST2/TNF/NLRP7 | 11 |
| BP | GO:0008631 | | intrinsic apoptotic signaling pathway in response to oxidative stress | 6/97 | 45/18723 | 1.14E-07 | 3.41E-06 | 2.00E-06 | BCL2/SIRT1/NFE2L2/AKT1/PARP1/TREM2 | 6 |
| BP | GO:0009267 | | cellular response to starvation | 9/97 | 156/18723 | 1.17E-07 | 3.47E-06 | 2.03E-06 | BCL2/JUN/IFI16/SIRT1/NFE2L2/FOXO3/SESN2/TP53/CTSV | 9 |
| BP | GO:0002573 | | myeloid leukocyte differentiation | 10/97 | 208/18723 | 1.25E-07 | 3.67E-06 | 2.15E-06 | JUN/IFI16/SIRT1/FADD/CASP8/TLR2/PARP1/TREM2/ANXA2/TNF | 10 |
| BP | GO:0071347 | | cellular response to interleukin-1 | 8/97 | 113/18723 | 1.27E-07 | 3.68E-06 | 2.15E-06 | NFKB1/CXCL8/PYCARD/CHI3L1/ZBP1/IL1RN/NLRP7/CAMP | 8 |
| BP | GO:0072332 | | intrinsic apoptotic signaling pathway by p53 class mediator | 7/97 | 76/18723 | 1.31E-07 | 3.78E-06 | 2.21E-06 | BCL2/IFI16/SIRT1/MDM2/TP53/PYCARD/TP63 | 7 |
| BP | GO:0062012 | | regulation of small molecule metabolic process | 12/97 | 334/18723 | 1.56E-07 | 4.47E-06 | 2.61E-06 | P2RX7/SIRT1/GPER1/NFKB1/SESN2/AKT1/TP53/APOE/PARP1/MST1/TREM2/TNF | 12 |
| BP | GO:0042149 | | cellular response to glucose starvation | 6/97 | 48/18723 | 1.70E-07 | 4.78E-06 | 2.80E-06 | BCL2/IFI16/NFE2L2/FOXO3/SESN2/TP53 | 6 |
| BP | GO:0031669 | | cellular response to nutrient levels | 10/97 | 215/18723 | 1.70E-07 | 4.78E-06 | 2.80E-06 | BCL2/JUN/IFI16/SIRT1/NFE2L2/FOXO3/MDM2/SESN2/TP53/CTSV | 10 |
| BP | GO:0009612 | | response to mechanical stimulus | 10/97 | 216/18723 | 1.78E-07 | 4.95E-06 | 2.90E-06 | TXNIP/JUN/EGFR/NFKB1/FADD/AKT1/CASP8/BNIP3/CHI3L1/CASP5 | 10 |
| BP | GO:0060544 | | regulation of necroptotic process | 5/97 | 26/18723 | 2.03E-07 | 5.60E-06 | 3.27E-06 | BIRC2/FADD/CASP8/RIPK3/ZBP1 | 5 |
| BP | GO:0071260 | | cellular response to mechanical stimulus | 7/97 | 81/18723 | 2.05E-07 | 5.60E-06 | 3.27E-06 | EGFR/NFKB1/FADD/AKT1/CASP8/BNIP3/CASP5 | 7 |
| BP | GO:0042110 | | T cell activation | 14/97 | 487/18723 | 2.09E-07 | 5.66E-06 | 3.31E-06 | BCL2/PRDM1/NLRP3/METTL3/FADD/GLMN/AKT1/CASP8/TP53/RIPK3/CASP3/PYCARD/IL18/IL36B | 14 |
| BP | GO:0031960 | | response to corticosteroid | 9/97 | 167/18723 | 2.10E-07 | 5.66E-06 | 3.31E-06 | BCL2/GPER1/EGFR/FOXO3/CASP3/PARP1/TNF/IL1RN/CTSV | 9 |
| BP | GO:0010508 | | positive regulation of autophagy | 8/97 | 124/18723 | 2.60E-07 | 6.96E-06 | 4.07E-06 | SIRT1/STING1/FOXO3/SESN2/ORMDL3/IRGM/MEFV/BNIP3 | 8 |
| BP | GO:2000117 | | negative regulation of cysteine-type endopeptidase activity | 7/97 | 86/18723 | 3.10E-07 | 8.18E-06 | 4.78E-06 | IFI16/CARD8/DDX3X/BIRC2/MDM2/AKT1/TNF | 7 |
| BP | GO:0044403 | | biological process involved in symbiotic interaction | 11/97 | 290/18723 | 3.11E-07 | 8.18E-06 | 4.78E-06 | CTSG/ANO6/JUN/EGFR/CXCL8/IRGM/APOE/ACE2/APOL1/CLEC5A/CAMP | 11 |
| BP | GO:0032621 | | interleukin-18 production | 4/97 | 12/18723 | 3.25E-07 | 8.40E-06 | 4.91E-06 | NLRP9/DHX9/TLR2/GBP5 | 4 |
| BP | GO:0032661 | | regulation of interleukin-18 production | 4/97 | 12/18723 | 3.25E-07 | 8.40E-06 | 4.91E-06 | NLRP9/DHX9/TLR2/GBP5 | 4 |
| BP | GO:0071900 | | regulation of protein serine/threonine kinase activity | 12/97 | 359/18723 | 3.39E-07 | 8.72E-06 | 5.10E-06 | SIRT1/PTEN/EGFR/DDX3X/SESN2/AKT1/CASP3/IRGM/APOE/MST1/PYCARD/TNF | 12 |
| BP | GO:0051604 | | protein maturation | 11/97 | 294/18723 | 3.57E-07 | 9.09E-06 | 5.31E-06 | CTSG/IFI16/CARD8/FADD/MDM2/CASP8/CASP3/PARP1/PYCARD/ACE2/NLRP7 | 11 |
| BP | GO:1902175 | | regulation of oxidative stress-induced intrinsic apoptotic signaling pathway | 5/97 | 29/18723 | 3.62E-07 | 9.15E-06 | 5.35E-06 | SIRT1/NFE2L2/AKT1/PARP1/TREM2 | 5 |
| BP | GO:1900407 | | regulation of cellular response to oxidative stress | 7/97 | 89/18723 | 3.92E-07 | 9.84E-06 | 5.75E-06 | SIRT1/NFE2L2/FOXO3/HDAC6/AKT1/PARP1/TREM2 | 7 |
| BP | GO:0048660 | | regulation of smooth muscle cell proliferation | 9/97 | 180/18723 | 3.97E-07 | 9.89E-06 | 5.78E-06 | JUN/GPER1/PTEN/EGFR/MDM2/AKT1/APOE/IL18/TNF | 9 |
| BP | GO:0032608 | | interferon-beta production | 6/97 | 56/18723 | 4.36E-07 | 1.07E-05 | 6.25E-06 | STING1/DDX3X/DHX9/IRF3/TLR2/PYCARD | 6 |
| BP | GO:0032648 | | regulation of interferon-beta production | 6/97 | 56/18723 | 4.36E-07 | 1.07E-05 | 6.25E-06 | STING1/DDX3X/DHX9/IRF3/TLR2/PYCARD | 6 |
| BP | GO:0048659 | | smooth muscle cell proliferation | 9/97 | 184/18723 | 4.78E-07 | 1.16E-05 | 6.80E-06 | JUN/GPER1/PTEN/EGFR/MDM2/AKT1/APOE/IL18/TNF | 9 |
| BP | GO:0032481 | | positive regulation of type I interferon production | 6/97 | 58/18723 | 5.39E-07 | 1.30E-05 | 7.61E-06 | STING1/DDX3X/DHX9/IRF3/CD14/TLR2 | 6 |
| BP | GO:0001906 | | cell killing | 9/97 | 188/18723 | 5.72E-07 | 1.37E-05 | 8.03E-06 | CTSG/P2RX7/LYST/FADD/RIPK3/APOL1/GZMB/IL18/CAMP | 9 |
| BP | GO:2000351 | | regulation of endothelial cell apoptotic process | 6/97 | 59/18723 | 5.97E-07 | 1.42E-05 | 8.32E-06 | ANO6/GPER1/NFE2L2/FOXO3/TNF/FGF21 | 6 |
| BP | GO:0035456 | | response to interferon-beta | 5/97 | 32/18723 | 6.06E-07 | 1.43E-05 | 8.36E-06 | IFI16/STING1/IRGM/BST2/AIM2 | 5 |
| BP | GO:0032479 | | regulation of type I interferon production | 7/97 | 95/18723 | 6.13E-07 | 1.43E-05 | 8.36E-06 | STING1/DDX3X/DHX9/IRF3/CD14/TLR2/PYCARD | 7 |
| BP | GO:0032606 | | type I interferon production | 7/97 | 95/18723 | 6.13E-07 | 1.43E-05 | 8.36E-06 | STING1/DDX3X/DHX9/IRF3/CD14/TLR2/PYCARD | 7 |
| BP | GO:0043122 | | regulation of I-kappaB kinase/NF-kappaB signaling | 10/97 | 249/18723 | 6.59E-07 | 1.53E-05 | 8.93E-06 | SIRT1/CARD8/BIRC2/FADD/CASP8/IRF3/PYCARD/TREM2/BST2/TNF | 10 |
| BP | GO:1901216 | | positive regulation of neuron death | 7/97 | 97/18723 | 7.07E-07 | 1.63E-05 | 9.50E-06 | JUN/FOXO3/CASP8/TP53/CASP3/PARP1/TNF | 7 |
| BP | GO:0032642 | | regulation of chemokine production | 7/97 | 98/18723 | 7.58E-07 | 1.72E-05 | 1.01E-05 | DDX3X/TLR2/PYCARD/TREM2/MEFV/IL18/TNF | 7 |
| BP | GO:0070301 | | cellular response to hydrogen peroxide | 7/97 | 98/18723 | 7.58E-07 | 1.72E-05 | 1.01E-05 | SIRT1/NFE2L2/FOXO3/HDAC6/MDM2/RIPK3/BNIP3 | 7 |
| BP | GO:0070555 | | response to interleukin-1 | 8/97 | 143/18723 | 7.75E-07 | 1.75E-05 | 1.02E-05 | NFKB1/CXCL8/PYCARD/CHI3L1/ZBP1/IL1RN/NLRP7/CAMP | 8 |
| BP | GO:0032757 | | positive regulation of interleukin-8 production | 6/97 | 62/18723 | 8.04E-07 | 1.78E-05 | 1.04E-05 | FADD/CD14/TLR2/PYCARD/CHI3L1/TNF | 6 |
| BP | GO:0008630 | | intrinsic apoptotic signaling pathway in response to DNA damage | 7/97 | 99/18723 | 8.12E-07 | 1.78E-05 | 1.04E-05 | BCL2/IFI16/SIRT1/TP53/PYCARD/TP63/TNF | 7 |
| BP | GO:0032602 | | chemokine production | 7/97 | 99/18723 | 8.12E-07 | 1.78E-05 | 1.04E-05 | DDX3X/TLR2/PYCARD/TREM2/MEFV/IL18/TNF | 7 |
| BP | GO:0010821 | | regulation of mitochondrion organization | 8/97 | 144/18723 | 8.17E-07 | 1.78E-05 | 1.04E-05 | GPER1/HDAC6/AKT1/TP53/PYCARD/TREM2/BNIP3/GZMB | 8 |
| BP | GO:0014065 | | phosphatidylinositol 3-kinase signaling | 8/97 | 144/18723 | 8.17E-07 | 1.78E-05 | 1.04E-05 | SIRT1/GPER1/PTEN/EGFR/AKT1/TREM2/IL18/TNF | 8 |
| BP | GO:0009895 | | negative regulation of catabolic process | 11/97 | 320/18723 | 8.24E-07 | 1.78E-05 | 1.04E-05 | BCL2/EGFR/DHX9/ELAVL1/AKT1/CPTP/TP53/TREM2/ANXA2/TNF/ALK | 11 |
| BP | GO:0042594 | | response to starvation | 9/97 | 197/18723 | 8.47E-07 | 1.82E-05 | 1.07E-05 | BCL2/JUN/IFI16/SIRT1/NFE2L2/FOXO3/SESN2/TP53/CTSV | 9 |
| BP | GO:0010940 | | positive regulation of necrotic cell death | 4/97 | 15/18723 | 8.84E-07 | 1.89E-05 | 1.11E-05 | TP53/RIPK3/BNIP3/ZBP1 | 4 |
| BP | GO:0062014 | | negative regulation of small molecule metabolic process | 7/97 | 102/18723 | 9.95E-07 | 2.11E-05 | 1.23E-05 | SIRT1/NFKB1/AKT1/TP53/APOE/PARP1/MST1 | 7 |
| BP | GO:0072577 | | endothelial cell apoptotic process | 6/97 | 65/18723 | 1.07E-06 | 2.25E-05 | 1.32E-05 | ANO6/GPER1/NFE2L2/FOXO3/TNF/FGF21 | 6 |
| BP | GO:0050863 | | regulation of T cell activation | 11/97 | 329/18723 | 1.08E-06 | 2.27E-05 | 1.33E-05 | PRDM1/NLRP3/METTL3/FADD/GLMN/AKT1/RIPK3/CASP3/PYCARD/IL18/IL36B | 11 |
| BP | GO:2001236 | | regulation of extrinsic apoptotic signaling pathway | 8/97 | 151/18723 | 1.17E-06 | 2.43E-05 | 1.42E-05 | BCL2/GPER1/PTEN/DDX3X/FADD/AKT1/PYCARD/TNF | 8 |
| BP | GO:0097202 | | activation of cysteine-type endopeptidase activity | 4/97 | 16/18723 | 1.17E-06 | 2.43E-05 | 1.42E-05 | IFI16/FADD/CASP8/PYCARD | 4 |
| BP | GO:0043029 | | T cell homeostasis | 5/97 | 37/18723 | 1.29E-06 | 2.65E-05 | 1.55E-05 | BCL2/FADD/AKT1/RIPK3/CASP3 | 5 |
| BP | GO:0051341 | | regulation of oxidoreductase activity | 7/97 | 107/18723 | 1.38E-06 | 2.81E-05 | 1.64E-05 | EGFR/NFKB1/HDAC6/AKT1/RIPK3/APOE/TNF | 7 |
| BP | GO:0043491 | | protein kinase B signaling | 9/97 | 211/18723 | 1.50E-06 | 3.05E-05 | 1.78E-05 | SIRT1/GPER1/PTEN/EGFR/SESN2/AKT1/CHI3L1/IL18/TNF | 9 |
| BP | GO:0043434 | | response to peptide hormone | 12/97 | 414/18723 | 1.52E-06 | 3.08E-05 | 1.80E-05 | SIRT1/GPER1/PTEN/NFKB1/NFE2L2/EEF2K/MDM2/SESN2/AKT1/TLR2/PARP1/FGF21 | 12 |
| BP | GO:0009749 | | response to glucose | 9/97 | 212/18723 | 1.56E-06 | 3.10E-05 | 1.81E-05 | TXNIP/GPER1/PTEN/FOXO3/SESN2/CASP3/TREM2/FGF21/CTSV | 9 |
| BP | GO:0043523 | | regulation of neuron apoptotic process | 9/97 | 212/18723 | 1.56E-06 | 3.10E-05 | 1.81E-05 | BCL2/JUN/FOXO3/TP53/CASP3/APOE/PARP1/TREM2/TNF | 9 |
| BP | GO:1902107 | | positive regulation of leukocyte differentiation | 8/97 | 157/18723 | 1.57E-06 | 3.10E-05 | 1.81E-05 | JUN/NLRP3/FADD/CASP8/TREM2/IL18/TNF/IL36B | 8 |
| BP | GO:1903708 | | positive regulation of hemopoiesis | 8/97 | 157/18723 | 1.57E-06 | 3.10E-05 | 1.81E-05 | JUN/NLRP3/FADD/CASP8/TREM2/IL18/TNF/IL36B | 8 |
| BP | GO:0032728 | | positive regulation of interferon-beta production | 5/97 | 39/18723 | 1.68E-06 | 3.30E-05 | 1.93E-05 | STING1/DDX3X/DHX9/IRF3/TLR2 | 5 |
| BP | GO:0014066 | | regulation of phosphatidylinositol 3-kinase signaling | 7/97 | 111/18723 | 1.76E-06 | 3.43E-05 | 2.01E-05 | SIRT1/GPER1/PTEN/EGFR/TREM2/IL18/TNF | 7 |
| BP | GO:0032102 | | negative regulation of response to external stimulus | 12/97 | 420/18723 | 1.77E-06 | 3.43E-05 | 2.01E-05 | IFI16/GPER1/PTEN/NFKB1/CARD8/NLRP3/METTL3/APOE/TREM2/MEFV/ANXA2/TNF | 12 |
| BP | GO:1902105 | | regulation of leukocyte differentiation | 10/97 | 279/18723 | 1.85E-06 | 3.56E-05 | 2.08E-05 | JUN/PRDM1/NLRP3/METTL3/FADD/CASP8/TREM2/IL18/TNF/IL36B | 10 |
| BP | GO:0002821 | | positive regulation of adaptive immune response | 7/97 | 112/18723 | 1.87E-06 | 3.58E-05 | 2.09E-05 | SIRT1/NLRP3/FADD/PYCARD/TREM2/IL18/TNF | 7 |
| BP | GO:0070374 | | positive regulation of ERK1 and ERK2 cascade | 9/97 | 217/18723 | 1.89E-06 | 3.60E-05 | 2.10E-05 | GPER1/PTEN/EGFR/APOE/PYCARD/TREM2/CHI3L1/TNF/FGF21 | 9 |
| BP | GO:0002534 | | cytokine production involved in inflammatory response | 6/97 | 72/18723 | 1.96E-06 | 3.67E-05 | 2.15E-05 | PYCARD/TREM2/MEFV/GBP5/TNF/NLRP7 | 6 |
| BP | GO:0070227 | | lymphocyte apoptotic process | 6/97 | 72/18723 | 1.96E-06 | 3.67E-05 | 2.15E-05 | PTEN/FADD/AKT1/ORMDL3/TP53/RIPK3 | 6 |
| BP | GO:1900015 | | regulation of cytokine production involved in inflammatory response | 6/97 | 72/18723 | 1.96E-06 | 3.67E-05 | 2.15E-05 | PYCARD/TREM2/MEFV/GBP5/TNF/NLRP7 | 6 |
| BP | GO:0009746 | | response to hexose | 9/97 | 219/18723 | 2.04E-06 | 3.77E-05 | 2.21E-05 | TXNIP/GPER1/PTEN/FOXO3/SESN2/CASP3/TREM2/FGF21/CTSV | 9 |
| BP | GO:0034764 | | positive regulation of transmembrane transport | 9/97 | 219/18723 | 2.04E-06 | 3.77E-05 | 2.21E-05 | ANO6/P2RX7/GPER1/NFE2L2/GSTO1/AKT1/TREM2/ACE2/FGF21 | 9 |
| BP | GO:1903131 | | mononuclear cell differentiation | 12/97 | 426/18723 | 2.05E-06 | 3.78E-05 | 2.21E-05 | BCL2/JUN/IFI16/PRDM1/NLRP3/METTL3/FADD/TP53/RIPK3/TREM2/IL18/IL36B | 12 |
| BP | GO:0010822 | | positive regulation of mitochondrion organization | 6/97 | 74/18723 | 2.31E-06 | 4.22E-05 | 2.47E-05 | GPER1/TP53/PYCARD/TREM2/BNIP3/GZMB | 6 |
| BP | GO:0001818 | | negative regulation of cytokine production | 11/97 | 357/18723 | 2.39E-06 | 4.35E-05 | 2.54E-05 | NFKB1/CARD8/NLRP3/CPTP/SERPINB1/PYCARD/TREM2/MEFV/BST2/TNF/NLRP7 | 11 |
| BP | GO:0034284 | | response to monosaccharide | 9/97 | 225/18723 | 2.55E-06 | 4.61E-05 | 2.69E-05 | TXNIP/GPER1/PTEN/FOXO3/SESN2/CASP3/TREM2/FGF21/CTSV | 9 |
| BP | GO:0071375 | | cellular response to peptide hormone stimulus | 10/97 | 290/18723 | 2.61E-06 | 4.70E-05 | 2.75E-05 | SIRT1/GPER1/PTEN/NFKB1/NFE2L2/EEF2K/MDM2/AKT1/PARP1/FGF21 | 10 |
| BP | GO:0016236 | | macroautophagy | 10/97 | 291/18723 | 2.69E-06 | 4.81E-05 | 2.81E-05 | SIRT1/STING1/HDAC6/SESN2/AKT1/CAPN1/TP53/CASP3/IRGM/BNIP3 | 10 |
| BP | GO:2000379 | | positive regulation of reactive oxygen species metabolic process | 6/97 | 76/18723 | 2.70E-06 | 4.81E-05 | 2.81E-05 | EGFR/NFE2L2/FOXO3/TP53/RIPK3/ACE2 | 6 |
| BP | GO:0002221 | | pattern recognition receptor signaling pathway | 8/97 | 172/18723 | 3.11E-06 | 5.46E-05 | 3.19E-05 | LY96/DDX3X/BIRC2/IRF3/CD14/TLR2/IRGM/TREM2 | 8 |
| BP | GO:0046006 | | regulation of activated T cell proliferation | 5/97 | 44/18723 | 3.11E-06 | 5.46E-05 | 3.19E-05 | FADD/RIPK3/CASP3/PYCARD/IL18 | 5 |
| BP | GO:1903706 | | regulation of hemopoiesis | 11/97 | 367/18723 | 3.12E-06 | 5.46E-05 | 3.19E-05 | JUN/PRDM1/NLRP3/FOXO3/METTL3/FADD/CASP8/TREM2/IL18/TNF/IL36B | 11 |
| BP | GO:0002224 | | toll-like receptor signaling pathway | 7/97 | 121/18723 | 3.14E-06 | 5.47E-05 | 3.20E-05 | LY96/DDX3X/BIRC2/IRF3/CD14/TLR2/TREM2 | 7 |
| BP | GO:0050900 | | leukocyte migration | 11/97 | 369/18723 | 3.28E-06 | 5.70E-05 | 3.33E-05 | ANO6/PECAM1/LYST/NLRP3/FADD/AKT1/RIPK3/CXCL8/PYCARD/TREM2/TNF | 11 |
| BP | GO:2000106 | | regulation of leukocyte apoptotic process | 6/97 | 81/18723 | 3.92E-06 | 6.77E-05 | 3.96E-05 | SIRT1/PTEN/FADD/ORMDL3/TP53/RIPK3 | 6 |
| BP | GO:0006109 | | regulation of carbohydrate metabolic process | 8/97 | 178/18723 | 4.01E-06 | 6.85E-05 | 4.00E-05 | P2RX7/SIRT1/GPER1/NFKB1/SESN2/AKT1/TP53/MST1 | 8 |
| BP | GO:0048015 | | phosphatidylinositol-mediated signaling | 8/97 | 178/18723 | 4.01E-06 | 6.85E-05 | 4.00E-05 | SIRT1/GPER1/PTEN/EGFR/AKT1/TREM2/IL18/TNF | 8 |
| BP | GO:0008625 | | extrinsic apoptotic signaling pathway via death domain receptors | 6/97 | 82/18723 | 4.22E-06 | 7.13E-05 | 4.17E-05 | BCL2/PTEN/DDX3X/FADD/CASP8/TNF | 6 |
| BP | GO:0007346 | | regulation of mitotic cell cycle | 12/97 | 457/18723 | 4.22E-06 | 7.13E-05 | 4.17E-05 | BCL2/NEK7/SIRT1/PTEN/EGFR/DDX3X/MRE11/MDM2/AKT1/TP53/TNF/MKI67 | 12 |
| BP | GO:0051346 | | negative regulation of hydrolase activity | 11/97 | 379/18723 | 4.24E-06 | 7.13E-05 | 4.17E-05 | IFI16/CARD8/DDX3X/BIRC2/MDM2/AKT1/SERPINB1/ANXA2/BST2/TNF/NLRP7 | 11 |
| BP | GO:0050798 | | activated T cell proliferation | 5/97 | 47/18723 | 4.34E-06 | 7.27E-05 | 4.25E-05 | FADD/RIPK3/CASP3/PYCARD/IL18 | 5 |
| MF | GO:0001786 | | phosphatidylserine binding | 6/97 | 60/18368 | 7.38E-07 | 7.58E-05 | 5.90E-05 | GSDMD/GSDMA/TREM2/ANXA2/GSDMB/GSDMC | 6 |
| BP | GO:0071359 | | cellular response to dsRNA | 4/97 | 22/18723 | 4.61E-06 | 7.67E-05 | 4.49E-05 | STING1/NFKB1/DHX9/IRF3 | 4 |
| BP | GO:0009410 | | response to xenobiotic stimulus | 12/97 | 462/18723 | 4.72E-06 | 7.79E-05 | 4.55E-05 | TXNIP/BCL2/JUN/PTEN/EGFR/NFE2L2/FOXO3/GSTO1/MDM2/CASP3/AIM2/FGF21 | 12 |
| BP | GO:0048017 | | inositol lipid-mediated signaling | 8/97 | 182/18723 | 4.73E-06 | 7.79E-05 | 4.55E-05 | SIRT1/GPER1/PTEN/EGFR/AKT1/TREM2/IL18/TNF | 8 |
| BP | GO:0090199 | | regulation of release of cytochrome c from mitochondria | 5/97 | 48/18723 | 4.83E-06 | 7.88E-05 | 4.61E-05 | GPER1/AKT1/TP53/PYCARD/BNIP3 | 5 |
| BP | GO:2001238 | | positive regulation of extrinsic apoptotic signaling pathway | 5/97 | 48/18723 | 4.83E-06 | 7.88E-05 | 4.61E-05 | GPER1/PTEN/FADD/PYCARD/TNF | 5 |
| BP | GO:0098586 | | cellular response to virus | 6/97 | 84/18723 | 4.85E-06 | 7.88E-05 | 4.61E-05 | GSDME/NFKB1/DDX3X/BIRC2/IRF3/IRGM | 6 |
| BP | GO:0002819 | | regulation of adaptive immune response | 8/97 | 183/18723 | 4.92E-06 | 7.95E-05 | 4.65E-05 | SIRT1/NLRP3/FADD/RIPK3/PYCARD/TREM2/IL18/TNF | 8 |
| BP | GO:0045069 | | regulation of viral genome replication | 6/97 | 85/18723 | 5.20E-06 | 8.32E-05 | 4.87E-05 | BCL2/IFI16/DDX3X/CXCL8/BST2/TNF | 6 |
| BP | GO:0097194 | | execution phase of apoptosis | 6/97 | 85/18723 | 5.20E-06 | 8.32E-05 | 4.87E-05 | GPER1/AKT1/CASP8/TP53/CASP3/CASP6 | 6 |
| BP | GO:0033674 | | positive regulation of kinase activity | 12/97 | 467/18723 | 5.27E-06 | 8.39E-05 | 4.90E-05 | SIRT1/EGFR/DDX3X/MRE11/AKT1/RIPK3/IRGM/TREM2/CHI3L1/IL18/TNF/ALK | 12 |
| BP | GO:0030225 | | macrophage differentiation | 5/97 | 49/18723 | 5.35E-06 | 8.45E-05 | 4.94E-05 | SIRT1/FADD/CASP8/TLR2/PARP1 | 5 |
| BP | GO:0060964 | | regulation of gene silencing by miRNA | 5/97 | 49/18723 | 5.35E-06 | 8.45E-05 | 4.94E-05 | EGFR/DHX9/ELAVL1/TP53/TNF | 5 |
| BP | GO:0033002 | | muscle cell proliferation | 9/97 | 248/18723 | 5.63E-06 | 8.84E-05 | 5.17E-05 | JUN/GPER1/PTEN/EGFR/MDM2/AKT1/APOE/IL18/TNF | 9 |
| BP | GO:0019058 | | viral life cycle | 10/97 | 317/18723 | 5.75E-06 | 8.98E-05 | 5.25E-05 | BCL2/IFI16/EGFR/DDX3X/CXCL8/APOE/ACE2/BST2/CLEC5A/TNF | 10 |
| BP | GO:0070661 | | leukocyte proliferation | 10/97 | 318/18723 | 5.91E-06 | 9.17E-05 | 5.36E-05 | BCL2/FADD/GLMN/TP53/RIPK3/CASP3/PYCARD/TREM2/BST2/IL18 | 10 |
| BP | GO:0001961 | | positive regulation of cytokine-mediated signaling pathway | 5/97 | 50/18723 | 5.93E-06 | 9.17E-05 | 5.36E-05 | FADD/IRF3/IRGM/TREM2/ZBP1 | 5 |
| BP | GO:0050829 | | defense response to Gram-negative bacterium | 6/97 | 88/18723 | 6.36E-06 | 9.81E-05 | 5.73E-05 | CTSG/GSDMD/IRGM/PYCARD/TREM2/CAMP | 6 |
| BP | GO:0043124 | | negative regulation of I-kappaB kinase/NF-kappaB signaling | 5/97 | 51/18723 | 6.54E-06 | 0.0001 | 5.87E-05 | SIRT1/CARD8/CASP8/PYCARD/TREM2 | 5 |
| BP | GO:0009743 | | response to carbohydrate | 9/97 | 253/18723 | 6.62E-06 | 0.0001 | 5.87E-05 | TXNIP/GPER1/PTEN/FOXO3/SESN2/CASP3/TREM2/FGF21/CTSV | 9 |
| BP | GO:0097305 | | response to alcohol | 9/97 | 253/18723 | 6.62E-06 | 0.0001 | 5.87E-05 | PTEN/NLRP3/FOXO3/BIRC2/MDM2/AKT1/CASP8/CD14/PARP1 | 9 |
| BP | GO:0035458 | | cellular response to interferon-beta | 4/97 | 24/18723 | 6.64E-06 | 0.0001 | 5.87E-05 | IFI16/STING1/IRGM/AIM2 | 4 |
| BP | GO:0002218 | | activation of innate immune response | 5/97 | 52/18723 | 7.21E-06 | 0.000107 | 6.26E-05 | IFI16/STING1/PYCARD/ZBP1/AIM2 | 5 |
| BP | GO:0035196 | | production of miRNAs involved in gene silencing by miRNA | 5/97 | 52/18723 | 7.21E-06 | 0.000107 | 6.26E-05 | EGFR/DDX3X/METTL3/TP53/TNF | 5 |
| BP | GO:0060147 | | regulation of posttranscriptional gene silencing | 5/97 | 52/18723 | 7.21E-06 | 0.000107 | 6.26E-05 | EGFR/DHX9/ELAVL1/TP53/TNF | 5 |
| BP | GO:2000378 | | negative regulation of reactive oxygen species metabolic process | 5/97 | 52/18723 | 7.21E-06 | 0.000107 | 6.26E-05 | BCL2/HDAC6/AKT1/TP53/BNIP3 | 5 |
| BP | GO:0034976 | | response to endoplasmic reticulum stress | 9/97 | 256/18723 | 7.28E-06 | 0.000108 | 6.29E-05 | BCL2/JUN/SIRT1/NFE2L2/DDX3X/SESN2/TP53/CXCL8/FGF21 | 9 |
| BP | GO:0030217 | | T cell differentiation | 9/97 | 257/18723 | 7.51E-06 | 0.000111 | 6.46E-05 | BCL2/PRDM1/NLRP3/METTL3/FADD/TP53/RIPK3/IL18/IL36B | 9 |
| BP | GO:0060966 | | regulation of gene silencing by RNA | 5/97 | 53/18723 | 7.93E-06 | 0.000116 | 6.79E-05 | EGFR/DHX9/ELAVL1/TP53/TNF | 5 |
| BP | GO:0019216 | | regulation of lipid metabolic process | 10/97 | 331/18723 | 8.40E-06 | 0.000123 | 7.16E-05 | SIRT1/GPER1/NFKB1/AKT1/ORMDL3/APOE/TREM2/TNF/ALK/FGF21 | 10 |
| BP | GO:0031333 | | negative regulation of protein-containing complex assembly | 7/97 | 141/18723 | 8.64E-06 | 0.000125 | 7.30E-05 | CARD8/DDX3X/BIRC2/HDAC6/CPTP/TREM2/MEFV | 7 |
| BP | GO:0031050 | | dsRNA processing | 5/97 | 54/18723 | 8.70E-06 | 0.000125 | 7.30E-05 | EGFR/DDX3X/METTL3/TP53/TNF | 5 |
| BP | GO:0070228 | | regulation of lymphocyte apoptotic process | 5/97 | 54/18723 | 8.70E-06 | 0.000125 | 7.30E-05 | PTEN/FADD/ORMDL3/TP53/RIPK3 | 5 |
| BP | GO:0070918 | | production of small RNA involved in gene silencing by RNA | 5/97 | 54/18723 | 8.70E-06 | 0.000125 | 7.30E-05 | EGFR/DDX3X/METTL3/TP53/TNF | 5 |
| BP | GO:0050729 | | positive regulation of inflammatory response | 7/97 | 142/18723 | 9.05E-06 | 0.000129 | 7.52E-05 | EGFR/DHX9/TLR2/TREM2/IL18/ZBP1/TNF | 7 |
| BP | GO:2000045 | | regulation of G1/S transition of mitotic cell cycle | 7/97 | 142/18723 | 9.05E-06 | 0.000129 | 7.52E-05 | BCL2/PTEN/EGFR/DDX3X/MDM2/AKT1/TP53 | 7 |
| MF | GO:0005126 | | cytokine receptor binding | 10/97 | 271/18368 | 1.68E-06 | 0.000139 | 0.000108 | FADD/CASP8/CASP3/CXCL8/PYCARD/IL18/TNF/IL1RN/IL36B/IL36G | 10 |
| BP | GO:0002532 | | production of molecular mediator involved in inflammatory response | 6/97 | 95/18723 | 9.91E-06 | 0.000139 | 8.14E-05 | PYCARD/TREM2/MEFV/GBP5/TNF/NLRP7 | 6 |
| BP | GO:0050764 | | regulation of phagocytosis | 6/97 | 95/18723 | 9.91E-06 | 0.000139 | 8.14E-05 | ANO6/PTEN/TLR2/PYCARD/TREM2/TNF | 6 |
| BP | GO:0033135 | | regulation of peptidyl-serine phosphorylation | 7/97 | 144/18723 | 9.92E-06 | 0.000139 | 8.14E-05 | BCL2/PTEN/EGFR/HDAC6/AKT1/IRGM/TNF | 7 |
| BP | GO:0007568 | | aging | 10/97 | 339/18723 | 1.03E-05 | 0.000145 | 8.45E-05 | BCL2/JUN/SIRT1/PTEN/NFE2L2/FOXO3/AKT1/TP53/TP63/CTSV | 10 |
| BP | GO:1904035 | | regulation of epithelial cell apoptotic process | 6/97 | 96/18723 | 1.05E-05 | 0.000147 | 8.57E-05 | ANO6/GPER1/NFE2L2/FOXO3/TNF/FGF21 | 6 |
| BP | GO:0043255 | | regulation of carbohydrate biosynthetic process | 6/97 | 97/18723 | 1.12E-05 | 0.000155 | 9.06E-05 | SIRT1/GPER1/NFKB1/SESN2/AKT1/MST1 | 6 |
| BP | GO:2001243 | | negative regulation of intrinsic apoptotic signaling pathway | 6/97 | 98/18723 | 1.19E-05 | 0.000162 | 9.49E-05 | BCL2/SIRT1/NFE2L2/DDX3X/MDM2/AKT1 | 6 |
| BP | GO:0010212 | | response to ionizing radiation | 7/97 | 148/18723 | 1.19E-05 | 0.000162 | 9.49E-05 | BCL2/IFI16/SIRT1/MDM2/TP53/CASP3/PARP1 | 7 |
| BP | GO:0051384 | | response to glucocorticoid | 7/97 | 148/18723 | 1.19E-05 | 0.000162 | 9.49E-05 | BCL2/EGFR/FOXO3/CASP3/TNF/IL1RN/CTSV | 7 |
| BP | GO:0048608 | | reproductive structure development | 11/97 | 424/18723 | 1.22E-05 | 0.000167 | 9.74E-05 | BCL2/PRDM1/SIRT1/PTEN/FOXO3/BIRC2/AKT1/CASP8/CASP3/TP63/CTSV | 11 |
| BP | GO:0002763 | | positive regulation of myeloid leukocyte differentiation | 5/97 | 58/18723 | 1.24E-05 | 0.000168 | 9.80E-05 | JUN/FADD/CASP8/TREM2/TNF | 5 |
| BP | GO:0043525 | | positive regulation of neuron apoptotic process | 5/97 | 58/18723 | 1.24E-05 | 0.000168 | 9.80E-05 | JUN/FOXO3/TP53/CASP3/TNF | 5 |
| BP | GO:0090200 | | positive regulation of release of cytochrome c from mitochondria | 4/97 | 28/18723 | 1.26E-05 | 0.00017 | 9.92E-05 | GPER1/TP53/PYCARD/BNIP3 | 4 |
| BP | GO:0061458 | | reproductive system development | 11/97 | 427/18723 | 1.31E-05 | 0.000175 | 0.000102 | BCL2/PRDM1/SIRT1/PTEN/FOXO3/BIRC2/AKT1/CASP8/CASP3/TP63/CTSV | 11 |
| BP | GO:1903829 | | positive regulation of cellular protein localization | 9/97 | 276/18723 | 1.33E-05 | 0.000177 | 0.000104 | GPER1/EGFR/SESN2/AKT1/ORMDL3/PARP1/TREM2/GZMB/TNF | 9 |
| BP | GO:0045807 | | positive regulation of endocytosis | 6/97 | 100/18723 | 1.33E-05 | 0.000177 | 0.000104 | ANO6/EEF2K/CD14/APOE/TREM2/ANXA2 | 6 |
| BP | GO:0002685 | | regulation of leukocyte migration | 8/97 | 210/18723 | 1.35E-05 | 0.000177 | 0.000104 | ANO6/FADD/AKT1/RIPK3/CXCL8/PYCARD/TREM2/TNF | 8 |
| BP | GO:0032768 | | regulation of monooxygenase activity | 5/97 | 59/18723 | 1.35E-05 | 0.000177 | 0.000104 | EGFR/NFKB1/AKT1/APOE/TNF | 5 |
| BP | GO:0051055 | | negative regulation of lipid biosynthetic process | 5/97 | 59/18723 | 1.35E-05 | 0.000177 | 0.000104 | SIRT1/GPER1/NFKB1/ORMDL3/APOE | 5 |
| BP | GO:0050830 | | defense response to Gram-positive bacterium | 6/97 | 101/18723 | 1.41E-05 | 0.000185 | 0.000108 | CTSG/GSDMD/TLR2/PYCARD/TNF/CAMP | 6 |
| BP | GO:0033028 | | myeloid cell apoptotic process | 4/97 | 29/18723 | 1.46E-05 | 0.000189 | 0.000111 | BCL2/SIRT1/PTEN/IRF3 | 4 |
| BP | GO:1902253 | | regulation of intrinsic apoptotic signaling pathway by p53 class mediator | 4/97 | 29/18723 | 1.46E-05 | 0.000189 | 0.000111 | BCL2/SIRT1/MDM2/TP53 | 4 |
| BP | GO:0032677 | | regulation of interleukin-8 production | 6/97 | 102/18723 | 1.49E-05 | 0.000193 | 0.000113 | FADD/CD14/TLR2/PYCARD/CHI3L1/TNF | 6 |
| BP | GO:0002683 | | negative regulation of immune system process | 11/97 | 434/18723 | 1.52E-05 | 0.000196 | 0.000114 | IFI16/GPER1/METTL3/FADD/GLMN/AKT1/CASP3/TREM2/BST2/IL13RA2/TNF | 11 |
| BP | GO:0032025 | | response to cobalt ion | 3/97 | 10/18723 | 1.58E-05 | 0.000199 | 0.000116 | CASP8/CASP3/BNIP3 | 3 |
| BP | GO:0070391 | | response to lipoteichoic acid | 3/97 | 10/18723 | 1.58E-05 | 0.000199 | 0.000116 | CD14/TLR2/TREM2 | 3 |
| BP | GO:0071223 | | cellular response to lipoteichoic acid | 3/97 | 10/18723 | 1.58E-05 | 0.000199 | 0.000116 | CD14/TLR2/TREM2 | 3 |
| BP | GO:0032637 | | interleukin-8 production | 6/97 | 103/18723 | 1.58E-05 | 0.000199 | 0.000116 | FADD/CD14/TLR2/PYCARD/CHI3L1/TNF | 6 |
| BP | GO:0032760 | | positive regulation of tumor necrosis factor production | 6/97 | 103/18723 | 1.58E-05 | 0.000199 | 0.000116 | LY96/DHX9/FADD/CD14/TLR2/PYCARD | 6 |
| BP | GO:0045639 | | positive regulation of myeloid cell differentiation | 6/97 | 103/18723 | 1.58E-05 | 0.000199 | 0.000116 | JUN/FOXO3/FADD/CASP8/TREM2/TNF | 6 |
| BP | GO:0048661 | | positive regulation of smooth muscle cell proliferation | 6/97 | 104/18723 | 1.67E-05 | 0.000209 | 0.000122 | JUN/EGFR/MDM2/AKT1/IL18/TNF | 6 |
| BP | GO:0002260 | | lymphocyte homeostasis | 5/97 | 62/18723 | 1.72E-05 | 0.000214 | 0.000125 | BCL2/FADD/AKT1/RIPK3/CASP3 | 5 |
| BP | GO:1903078 | | positive regulation of protein localization to plasma membrane | 5/97 | 62/18723 | 1.72E-05 | 0.000214 | 0.000125 | GPER1/EGFR/AKT1/TREM2/TNF | 5 |
| BP | GO:0002443 | | leukocyte mediated immunity | 11/97 | 440/18723 | 1.72E-05 | 0.000214 | 0.000125 | CTSG/LYST/NLRP3/FADD/RIPK3/TREM2/GZMB/BST2/IL18/IL13RA2/TNF | 11 |
| MF | GO:0004197 | | cysteine-type endopeptidase activity | 7/97 | 119/18368 | 3.18E-06 | 0.000218 | 0.00017 | CASP8/CAPN1/CASP3/PYCARD/CASP6/CASP5/CTSV | 7 |
| MF | GO:0031625 | | ubiquitin protein ligase binding | 10/97 | 297/18368 | 3.82E-06 | 0.000224 | 0.000175 | TXNIP/BCL2/JUN/EGFR/STING1/GLMN/HDAC6/MDM2/CASP8/TP53 | 10 |
| BP | GO:0035821 | | modulation of process of other organism | 6/97 | 106/18723 | 1.86E-05 | 0.000229 | 0.000134 | ANO6/P2RX7/JUN/GSDMD/IRGM/APOE | 6 |
| BP | GO:1905477 | | positive regulation of protein localization to membrane | 6/97 | 106/18723 | 1.86E-05 | 0.000229 | 0.000134 | GPER1/EGFR/AKT1/TREM2/GZMB/TNF | 6 |
| BP | GO:0016239 | | positive regulation of macroautophagy | 5/97 | 63/18723 | 1.86E-05 | 0.000229 | 0.000134 | SIRT1/STING1/SESN2/IRGM/BNIP3 | 5 |
| BP | GO:1903557 | | positive regulation of tumor necrosis factor superfamily cytokine production | 6/97 | 107/18723 | 1.96E-05 | 0.00024 | 0.00014 | LY96/DHX9/FADD/CD14/TLR2/PYCARD | 6 |
| MF | GO:0005546 | | phosphatidylinositol-4,5-bisphosphate binding | 6/97 | 82/18368 | 4.70E-06 | 0.000241 | 0.000188 | GSDME/GSDMD/GSDMA/ANXA2/GSDMB/GSDMC | 6 |
| BP | GO:0010876 | | lipid localization | 11/97 | 448/18723 | 2.04E-05 | 0.000247 | 0.000145 | ANO6/P2RX7/SIRT1/NFKB1/AKT1/CPTP/APOE/TREM2/ANXA2/APOL1/TNF | 11 |
| BP | GO:0022407 | | regulation of cell-cell adhesion | 11/97 | 448/18723 | 2.04E-05 | 0.000247 | 0.000145 | NLRP3/METTL3/FADD/GLMN/AKT1/CASP3/PYCARD/IL18/TNF/IL1RN/IL36B | 11 |
| BP | GO:0033138 | | positive regulation of peptidyl-serine phosphorylation | 6/97 | 108/18723 | 2.07E-05 | 0.00025 | 0.000146 | BCL2/EGFR/HDAC6/AKT1/IRGM/TNF | 6 |
| BP | GO:0034250 | | positive regulation of cellular amide metabolic process | 7/97 | 162/18723 | 2.13E-05 | 0.000257 | 0.00015 | NFE2L2/DDX3X/DHX9/METTL3/ELAVL1/CASP3/TNF | 7 |
| BP | GO:0048102 | | autophagic cell death | 3/97 | 11/18723 | 2.16E-05 | 0.000259 | 0.000151 | TREM2/BNIP3/CTSV | 3 |
| BP | GO:0038034 | | signal transduction in absence of ligand | 5/97 | 65/18723 | 2.17E-05 | 0.000259 | 0.000151 | BCL2/FOXO3/FADD/AKT1/TNF | 5 |
| BP | GO:0097192 | | extrinsic apoptotic signaling pathway in absence of ligand | 5/97 | 65/18723 | 2.17E-05 | 0.000259 | 0.000151 | BCL2/FOXO3/FADD/AKT1/TNF | 5 |
| BP | GO:0010001 | | glial cell differentiation | 8/97 | 225/18723 | 2.22E-05 | 0.000263 | 0.000154 | PTEN/EGFR/METTL3/AKT1/TLR2/TREM2/BNIP3/TNF | 8 |
| BP | GO:0007159 | | leukocyte cell-cell adhesion | 10/97 | 371/18723 | 2.25E-05 | 0.000266 | 0.000156 | PECAM1/NLRP3/FADD/GLMN/AKT1/CASP3/PYCARD/IL18/TNF/IL36B | 10 |
| BP | GO:0002703 | | regulation of leukocyte mediated immunity | 8/97 | 226/18723 | 2.29E-05 | 0.00027 | 0.000158 | NLRP3/FADD/RIPK3/TREM2/BST2/IL18/IL13RA2/TNF | 8 |
| BP | GO:0050792 | | regulation of viral process | 7/97 | 164/18723 | 2.31E-05 | 0.000271 | 0.000159 | BCL2/IFI16/DDX3X/DHX9/CXCL8/BST2/TNF | 7 |
| BP | GO:0009314 | | response to radiation | 11/97 | 456/18723 | 2.40E-05 | 0.000281 | 0.000164 | BCL2/JUN/IFI16/SIRT1/EGFR/METTL3/MDM2/AKT1/TP53/CASP3/PARP1 | 11 |
| BP | GO:0031647 | | regulation of protein stability | 9/97 | 298/18723 | 2.44E-05 | 0.000283 | 0.000166 | BCL2/SIRT1/PTEN/HDAC6/MDM2/TP53/CASP3/IRGM/PYCARD | 9 |
| BP | GO:0048511 | | rhythmic process | 9/97 | 298/18723 | 2.44E-05 | 0.000283 | 0.000166 | JUN/SIRT1/PTEN/EGFR/FOXO3/DHX9/METTL3/TP53/CASP3 | 9 |
| BP | GO:0032609 | | interferon-gamma production | 6/97 | 112/18723 | 2.54E-05 | 0.000294 | 0.000172 | FADD/CD14/RIPK3/PYCARD/IL18/TNF | 6 |
| BP | GO:0032649 | | regulation of interferon-gamma production | 6/97 | 112/18723 | 2.54E-05 | 0.000294 | 0.000172 | FADD/CD14/RIPK3/PYCARD/IL18/TNF | 6 |
| CC | GO:0031983 | | vesicle lumen | 10/97 | 327/19550 | 5.19E-06 | 0.000294 | 0.000239 | CTSG/EGFR/NFKB1/DDX3X/GSDMD/SERPINB1/PYCARD/ANXA2/CHI3L1/CAMP | 10 |
| BP | GO:0055088 | | lipid homeostasis | 7/97 | 167/18723 | 2.59E-05 | 0.000298 | 0.000174 | SIRT1/DDX3X/SESN2/ORMDL3/APOE/TREM2/IL18 | 7 |
| MF | GO:0044389 | | ubiquitin-like protein ligase binding | 10/97 | 316/18368 | 6.60E-06 | 0.000301 | 0.000235 | TXNIP/BCL2/JUN/EGFR/STING1/GLMN/HDAC6/MDM2/CASP8/TP53 | 10 |
| BP | GO:1902806 | | regulation of cell cycle G1/S phase transition | 7/97 | 168/18723 | 2.69E-05 | 0.000309 | 0.000181 | BCL2/PTEN/EGFR/DDX3X/MDM2/AKT1/TP53 | 7 |
| BP | GO:0055094 | | response to lipoprotein particle | 4/97 | 34/18723 | 2.79E-05 | 0.000317 | 0.000185 | AKT1/APOE/TREM2/FGF21 | 4 |
| BP | GO:1990000 | | amyloid fibril formation | 4/97 | 34/18723 | 2.79E-05 | 0.000317 | 0.000185 | MDM2/RIPK3/APOE/TREM2 | 4 |
| BP | GO:0032490 | | detection of molecule of bacterial origin | 3/97 | 12/18723 | 2.87E-05 | 0.000325 | 0.00019 | LY96/TLR2/TREM2 | 3 |
| BP | GO:1904377 | | positive regulation of protein localization to cell periphery | 5/97 | 69/18723 | 2.91E-05 | 0.000329 | 0.000192 | GPER1/EGFR/AKT1/TREM2/TNF | 5 |
| BP | GO:0071675 | | regulation of mononuclear cell migration | 6/97 | 115/18723 | 2.95E-05 | 0.000333 | 0.000195 | ANO6/FADD/AKT1/RIPK3/PYCARD/TNF | 6 |
| BP | GO:0046890 | | regulation of lipid biosynthetic process | 7/97 | 171/18723 | 3.02E-05 | 0.000339 | 0.000198 | SIRT1/GPER1/NFKB1/AKT1/ORMDL3/APOE/TNF | 7 |
| MF | GO:0072341 | | modified amino acid binding | 6/97 | 93/18368 | 9.77E-06 | 0.000342 | 0.000266 | GSDMD/GSDMA/TREM2/ANXA2/GSDMB/GSDMC | 6 |
| MF | GO:0070851 | | growth factor receptor binding | 7/97 | 141/18368 | 9.77E-06 | 0.000342 | 0.000266 | PTEN/GLMN/PYCARD/IL1RN/IL36B/FGF21/IL36G | 7 |
| MF | GO:0038187 | | pattern recognition receptor activity | 4/97 | 26/18368 | 9.99E-06 | 0.000342 | 0.000266 | LY96/CARD8/CD14/TLR2 | 4 |
| BP | GO:0021782 | | glial cell development | 6/97 | 116/18723 | 3.10E-05 | 0.000347 | 0.000203 | PTEN/EGFR/AKT1/TLR2/TREM2/TNF | 6 |
| BP | GO:0006909 | | phagocytosis | 9/97 | 308/18723 | 3.16E-05 | 0.000352 | 0.000206 | ANO6/PECAM1/LYST/PTEN/CD14/TLR2/PYCARD/TREM2/TNF | 9 |
| BP | GO:0070372 | | regulation of ERK1 and ERK2 cascade | 9/97 | 309/18723 | 3.24E-05 | 0.00036 | 0.00021 | GPER1/PTEN/EGFR/APOE/PYCARD/TREM2/CHI3L1/TNF/FGF21 | 9 |
| BP | GO:0051259 | | protein complex oligomerization | 8/97 | 238/18723 | 3.31E-05 | 0.000367 | 0.000214 | NLRP1/STING1/ELAVL1/GSDMD/TP53/PYCARD/TP63/GBP5 | 8 |
| BP | GO:0032722 | | positive regulation of chemokine production | 5/97 | 71/18723 | 3.34E-05 | 0.000369 | 0.000216 | DDX3X/TLR2/PYCARD/IL18/TNF | 5 |
| BP | GO:0060338 | | regulation of type I interferon-mediated signaling pathway | 4/97 | 36/18723 | 3.51E-05 | 0.000384 | 0.000225 | METTL3/FADD/IRF3/ZBP1 | 4 |
| BP | GO:0071402 | | cellular response to lipoprotein particle stimulus | 4/97 | 36/18723 | 3.51E-05 | 0.000384 | 0.000225 | AKT1/APOE/TREM2/FGF21 | 4 |
| BP | GO:0042176 | | regulation of protein catabolic process | 10/97 | 391/18723 | 3.52E-05 | 0.000384 | 0.000225 | PTEN/EGFR/NFE2L2/GLMN/MDM2/AKT1/APOE/TREM2/ANXA2/TNF | 10 |
| BP | GO:0030330 | | DNA damage response, signal transduction by p53 class mediator | 5/97 | 72/18723 | 3.58E-05 | 0.000388 | 0.000227 | SIRT1/FOXO3/MDM2/SESN2/TP53 | 5 |
| BP | GO:0032729 | | positive regulation of interferon-gamma production | 5/97 | 72/18723 | 3.58E-05 | 0.000388 | 0.000227 | FADD/CD14/PYCARD/IL18/TNF | 5 |
| BP | GO:0032042 | | mitochondrial DNA metabolic process | 3/97 | 13/18723 | 3.71E-05 | 0.000399 | 0.000233 | SESN2/TP53/PARP1 | 3 |
| BP | GO:0036462 | | TRAIL-activated apoptotic signaling pathway | 3/97 | 13/18723 | 3.71E-05 | 0.000399 | 0.000233 | PTEN/FADD/CASP8 | 3 |
| BP | GO:0060340 | | positive regulation of type I interferon-mediated signaling pathway | 3/97 | 13/18723 | 3.71E-05 | 0.000399 | 0.000233 | FADD/IRF3/ZBP1 | 3 |
| BP | GO:0071901 | | negative regulation of protein serine/threonine kinase activity | 6/97 | 120/18723 | 3.76E-05 | 0.000403 | 0.000235 | SIRT1/PTEN/AKT1/CASP3/APOE/PYCARD | 6 |
| BP | GO:0043410 | | positive regulation of MAPK cascade | 11/97 | 480/18723 | 3.83E-05 | 0.000409 | 0.000239 | GSDME/GPER1/PTEN/EGFR/APOE/PYCARD/TREM2/CHI3L1/TNF/ALK/FGF21 | 11 |
| BP | GO:1900016 | | negative regulation of cytokine production involved in inflammatory response | 4/97 | 37/18723 | 3.92E-05 | 0.000418 | 0.000244 | PYCARD/TREM2/MEFV/NLRP7 | 4 |
| BP | GO:1904019 | | epithelial cell apoptotic process | 6/97 | 121/18723 | 3.93E-05 | 0.000418 | 0.000244 | ANO6/GPER1/NFE2L2/FOXO3/TNF/FGF21 | 6 |
| BP | GO:0070663 | | regulation of leukocyte proliferation | 8/97 | 245/18723 | 4.07E-05 | 0.000428 | 0.00025 | BCL2/FADD/GLMN/RIPK3/CASP3/PYCARD/BST2/IL18 | 8 |
| BP | GO:0006869 | | lipid transport | 10/97 | 398/18723 | 4.08E-05 | 0.000428 | 0.00025 | ANO6/P2RX7/SIRT1/NFKB1/AKT1/CPTP/APOE/TREM2/ANXA2/APOL1 | 10 |
| BP | GO:0002437 | | inflammatory response to antigenic stimulus | 5/97 | 74/18723 | 4.08E-05 | 0.000428 | 0.00025 | TREM2/TNF/IL1RN/IL36B/IL36G | 5 |
| BP | GO:0046323 | | glucose import | 5/97 | 74/18723 | 4.08E-05 | 0.000428 | 0.00025 | NFE2L2/SESN2/AKT1/TNF/FGF21 | 5 |
| BP | GO:0009416 | | response to light stimulus | 9/97 | 320/18723 | 4.25E-05 | 0.000444 | 0.00026 | BCL2/SIRT1/EGFR/METTL3/MDM2/AKT1/TP53/CASP3/PARP1 | 9 |
| BP | GO:0032640 | | tumor necrosis factor production | 7/97 | 181/18723 | 4.34E-05 | 0.000445 | 0.00026 | LY96/DHX9/FADD/CD14/TLR2/PYCARD/TREM2 | 7 |
| BP | GO:0032680 | | regulation of tumor necrosis factor production | 7/97 | 181/18723 | 4.34E-05 | 0.000445 | 0.00026 | LY96/DHX9/FADD/CD14/TLR2/PYCARD/TREM2 | 7 |
| BP | GO:1905952 | | regulation of lipid localization | 7/97 | 181/18723 | 4.34E-05 | 0.000445 | 0.00026 | SIRT1/NFKB1/AKT1/APOE/TREM2/ANXA2/TNF | 7 |
| BP | GO:0045913 | | positive regulation of carbohydrate metabolic process | 5/97 | 75/18723 | 4.36E-05 | 0.000445 | 0.00026 | P2RX7/SIRT1/GPER1/NFKB1/AKT1 | 5 |
| BP | GO:0009595 | | detection of biotic stimulus | 4/97 | 38/18723 | 4.37E-05 | 0.000445 | 0.00026 | LY96/NLRP3/TLR2/TREM2 | 4 |
| BP | GO:0032373 | | positive regulation of sterol transport | 4/97 | 38/18723 | 4.37E-05 | 0.000445 | 0.00026 | SIRT1/APOE/TREM2/ANXA2 | 4 |
| BP | GO:0032376 | | positive regulation of cholesterol transport | 4/97 | 38/18723 | 4.37E-05 | 0.000445 | 0.00026 | SIRT1/APOE/TREM2/ANXA2 | 4 |
| BP | GO:1901031 | | regulation of response to reactive oxygen species | 4/97 | 38/18723 | 4.37E-05 | 0.000445 | 0.00026 | NFE2L2/FOXO3/HDAC6/SESN2 | 4 |
| BP | GO:0001909 | | leukocyte mediated cytotoxicity | 6/97 | 124/18723 | 4.52E-05 | 0.000459 | 0.000268 | CTSG/LYST/FADD/RIPK3/GZMB/IL18 | 6 |
| MF | GO:0070273 | | phosphatidylinositol-4-phosphate binding | 4/97 | 29/18368 | 1.57E-05 | 0.00049 | 0.000381 | GSDMD/GSDMA/GSDMB/GSDMC | 4 |
| MF | GO:0097199 | | cysteine-type endopeptidase activity involved in apoptotic signaling pathway | 3/97 | 10/18368 | 1.67E-05 | 0.00049 | 0.000381 | CASP8/CASP3/CASP5 | 3 |
| BP | GO:0051896 | | regulation of protein kinase B signaling | 7/97 | 185/18723 | 4.99E-05 | 0.000505 | 0.000295 | SIRT1/GPER1/PTEN/EGFR/CHI3L1/IL18/TNF | 7 |
| MF | GO:1990841 | | promoter-specific chromatin binding | 5/97 | 62/18368 | 1.89E-05 | 0.000517 | 0.000403 | PRDM1/SIRT1/DHX9/IRF3/TP53 | 5 |
| BP | GO:0071706 | | tumor necrosis factor superfamily cytokine production | 7/97 | 186/18723 | 5.16E-05 | 0.000518 | 0.000303 | LY96/DHX9/FADD/CD14/TLR2/PYCARD/TREM2 | 7 |
| BP | GO:1903555 | | regulation of tumor necrosis factor superfamily cytokine production | 7/97 | 186/18723 | 5.16E-05 | 0.000518 | 0.000303 | LY96/DHX9/FADD/CD14/TLR2/PYCARD/TREM2 | 7 |
| BP | GO:0048565 | | digestive tract development | 6/97 | 127/18723 | 5.16E-05 | 0.000518 | 0.000303 | BCL2/PRDM1/EGFR/CXCL8/TP63/TNF | 6 |
| BP | GO:0006919 | | activation of cysteine-type endopeptidase activity involved in apoptotic process | 5/97 | 78/18723 | 5.27E-05 | 0.000522 | 0.000305 | NLRP1/CARD8/CASP8/PYCARD/TNF | 5 |
| BP | GO:0032371 | | regulation of sterol transport | 5/97 | 78/18723 | 5.27E-05 | 0.000522 | 0.000305 | SIRT1/NFKB1/APOE/TREM2/ANXA2 | 5 |
| BP | GO:0032374 | | regulation of cholesterol transport | 5/97 | 78/18723 | 5.27E-05 | 0.000522 | 0.000305 | SIRT1/NFKB1/APOE/TREM2/ANXA2 | 5 |
| BP | GO:0043154 | | negative regulation of cysteine-type endopeptidase activity involved in apoptotic process | 5/97 | 78/18723 | 5.27E-05 | 0.000522 | 0.000305 | DDX3X/BIRC2/MDM2/AKT1/TNF | 5 |
| BP | GO:1903362 | | regulation of cellular protein catabolic process | 8/97 | 255/18723 | 5.39E-05 | 0.000532 | 0.000311 | PTEN/NFE2L2/GLMN/MDM2/AKT1/APOE/TREM2/ANXA2 | 8 |
| BP | GO:0070371 | | ERK1 and ERK2 cascade | 9/97 | 330/18723 | 5.39E-05 | 0.000532 | 0.000311 | GPER1/PTEN/EGFR/APOE/PYCARD/TREM2/CHI3L1/TNF/FGF21 | 9 |
| BP | GO:0014068 | | positive regulation of phosphatidylinositol 3-kinase signaling | 5/97 | 79/18723 | 5.60E-05 | 0.00055 | 0.000322 | SIRT1/GPER1/TREM2/IL18/TNF | 5 |
| MF | GO:1902936 | | phosphatidylinositol bisphosphate binding | 6/97 | 107/18368 | 2.18E-05 | 0.000561 | 0.000436 | GSDME/GSDMD/GSDMA/ANXA2/GSDMB/GSDMC | 6 |
| BP | GO:0031348 | | negative regulation of defense response | 8/97 | 258/18723 | 5.85E-05 | 0.000574 | 0.000336 | IFI16/GPER1/NFKB1/NLRP3/METTL3/APOE/TREM2/MEFV | 8 |
| BP | GO:0050691 | | regulation of defense response to virus by host | 4/97 | 41/18723 | 5.92E-05 | 0.000578 | 0.000338 | STING1/DHX9/PYCARD/AIM2 | 4 |
| BP | GO:0019079 | | viral genome replication | 6/97 | 131/18723 | 6.14E-05 | 0.000596 | 0.000349 | BCL2/IFI16/DDX3X/CXCL8/BST2/TNF | 6 |
| BP | GO:0046683 | | response to organophosphorus | 6/97 | 131/18723 | 6.14E-05 | 0.000596 | 0.000349 | P2RX7/JUN/PTEN/EEF2K/BIRC2/PANX1 | 6 |
| BP | GO:1903037 | | regulation of leukocyte cell-cell adhesion | 9/97 | 336/18723 | 6.20E-05 | 0.0006 | 0.000351 | NLRP3/FADD/GLMN/AKT1/CASP3/PYCARD/IL18/TNF/IL36B | 9 |
| BP | GO:0060968 | | regulation of gene silencing | 5/97 | 81/18723 | 6.31E-05 | 0.00061 | 0.000356 | EGFR/DHX9/ELAVL1/TP53/TNF | 5 |
| BP | GO:0018209 | | peptidyl-serine modification | 9/97 | 338/18723 | 6.49E-05 | 0.000621 | 0.000363 | BCL2/PTEN/EGFR/HDAC6/AKT1/IRGM/PARP1/MST1/TNF | 9 |
| BP | GO:0055090 | | acylglycerol homeostasis | 4/97 | 42/18723 | 6.51E-05 | 0.000621 | 0.000363 | SIRT1/SESN2/APOE/IL18 | 4 |
| BP | GO:0070328 | | triglyceride homeostasis | 4/97 | 42/18723 | 6.51E-05 | 0.000621 | 0.000363 | SIRT1/SESN2/APOE/IL18 | 4 |
| BP | GO:1902895 | | positive regulation of pri-miRNA transcription by RNA polymerase II | 4/97 | 42/18723 | 6.51E-05 | 0.000621 | 0.000363 | JUN/FOXO3/TP53/TNF | 4 |
| BP | GO:0031330 | | negative regulation of cellular catabolic process | 8/97 | 262/18723 | 6.52E-05 | 0.000621 | 0.000363 | BCL2/DHX9/ELAVL1/AKT1/CPTP/TP53/TREM2/ANXA2 | 8 |
| BP | GO:0002697 | | regulation of immune effector process | 9/97 | 339/18723 | 6.63E-05 | 0.00063 | 0.000368 | SIRT1/NLRP3/FADD/RIPK3/TREM2/BST2/IL18/IL13RA2/TNF | 9 |
| BP | GO:1901654 | | response to ketone | 7/97 | 194/18723 | 6.73E-05 | 0.000637 | 0.000372 | TXNIP/SIRT1/EGFR/FOXO3/AKT1/TLR2/PARP1 | 7 |
| BP | GO:0061041 | | regulation of wound healing | 6/97 | 134/18723 | 6.96E-05 | 0.000657 | 0.000384 | ANO6/PTEN/NFE2L2/APOE/ANXA2/TNF | 6 |
| BP | GO:0030522 | | intracellular receptor signaling pathway | 8/97 | 265/18723 | 7.06E-05 | 0.000665 | 0.000389 | SIRT1/GPER1/BIRC2/HDAC6/IRF3/IRGM/PARP1/TP63 | 8 |
| BP | GO:0071674 | | mononuclear cell migration | 7/97 | 196/18723 | 7.18E-05 | 0.000674 | 0.000394 | ANO6/PECAM1/FADD/AKT1/RIPK3/PYCARD/TNF | 7 |
| CC | GO:0042581 | | specific granule | 7/97 | 160/19550 | 1.49E-05 | 0.000678 | 0.000551 | ANO6/NFKB1/GSDMD/ORMDL3/CHI3L1/CLEC5A/CAMP | 7 |
| BP | GO:0002687 | | positive regulation of leukocyte migration | 6/97 | 135/18723 | 7.26E-05 | 0.000679 | 0.000397 | ANO6/FADD/CXCL8/PYCARD/TREM2/TNF | 6 |
| BP | GO:0071248 | | cellular response to metal ion | 7/97 | 197/18723 | 7.41E-05 | 0.000692 | 0.000404 | JUN/EGFR/NFE2L2/EEF2K/AKT1/PARP1/BNIP3 | 7 |
| BP | GO:0006970 | | response to osmotic stress | 5/97 | 84/18723 | 7.51E-05 | 0.000695 | 0.000407 | EGFR/DDX3X/TP53/CASP3/TNF | 5 |
| BP | GO:0010660 | | regulation of muscle cell apoptotic process | 5/97 | 84/18723 | 7.51E-05 | 0.000695 | 0.000407 | SIRT1/PTEN/NFE2L2/TP53/BNIP3 | 5 |
| BP | GO:0097061 | | dendritic spine organization | 5/97 | 84/18723 | 7.51E-05 | 0.000695 | 0.000407 | PTEN/EEF2K/HDAC6/APOE/TREM2 | 5 |
| BP | GO:0051348 | | negative regulation of transferase activity | 8/97 | 268/18723 | 7.64E-05 | 0.000705 | 0.000412 | SIRT1/PTEN/AKT1/ORMDL3/TP53/CASP3/APOE/PYCARD | 8 |
| MF | GO:0061134 | | peptidase regulator activity | 8/97 | 230/18368 | 2.97E-05 | 0.000718 | 0.000559 | NLRP1/CARD8/BIRC2/SERPINB1/PYCARD/ANXA2/BST2/NLRP7 | 8 |
| BP | GO:0050999 | | regulation of nitric-oxide synthase activity | 4/97 | 44/18723 | 7.84E-05 | 0.000721 | 0.000422 | EGFR/AKT1/APOE/TNF | 4 |
| BP | GO:0055123 | | digestive system development | 6/97 | 137/18723 | 7.87E-05 | 0.000723 | 0.000422 | BCL2/PRDM1/EGFR/CXCL8/TP63/TNF | 6 |
| BP | GO:0042098 | | T cell proliferation | 7/97 | 199/18723 | 7.90E-05 | 0.000723 | 0.000423 | FADD/GLMN/TP53/RIPK3/CASP3/PYCARD/IL18 | 7 |
| BP | GO:0010507 | | negative regulation of autophagy | 5/97 | 85/18723 | 7.95E-05 | 0.000726 | 0.000424 | BCL2/AKT1/CPTP/TP53/TREM2 | 5 |
| BP | GO:0051054 | | positive regulation of DNA metabolic process | 7/97 | 201/18723 | 8.41E-05 | 0.000765 | 0.000447 | NEK7/SIRT1/EGFR/MRE11/DHX9/AKT1/PARP1 | 7 |
| BP | GO:0002449 | | lymphocyte mediated immunity | 9/97 | 350/18723 | 8.47E-05 | 0.000769 | 0.000449 | LYST/NLRP3/FADD/RIPK3/TREM2/GZMB/IL18/IL13RA2/TNF | 9 |
| BP | GO:0019722 | | calcium-mediated signaling | 7/97 | 202/18723 | 8.67E-05 | 0.000785 | 0.000459 | P2RX7/EGFR/GSTO1/CXCL8/IRGM/TREM2/TNF | 7 |
| BP | GO:0034349 | | glial cell apoptotic process | 3/97 | 17/18723 | 8.70E-05 | 0.000785 | 0.000459 | TP53/CASP3/TREM2 | 3 |
| BP | GO:0001776 | | leukocyte homeostasis | 5/97 | 87/18723 | 8.88E-05 | 0.000798 | 0.000466 | BCL2/FADD/AKT1/RIPK3/CASP3 | 5 |
| BP | GO:0019915 | | lipid storage | 5/97 | 87/18723 | 8.88E-05 | 0.000798 | 0.000466 | SIRT1/NFKB1/APOE/TREM2/TNF | 5 |
| BP | GO:0071383 | | cellular response to steroid hormone stimulus | 7/97 | 204/18723 | 9.22E-05 | 0.000822 | 0.000481 | SIRT1/GPER1/EGFR/FOXO3/HDAC6/PARP1/TP63 | 7 |
| BP | GO:0032355 | | response to estradiol | 6/97 | 141/18723 | 9.23E-05 | 0.000822 | 0.000481 | TXNIP/GPER1/PTEN/EGFR/CASP8/CASP3 | 6 |
| BP | GO:0034341 | | response to interferon-gamma | 6/97 | 141/18723 | 9.23E-05 | 0.000822 | 0.000481 | TP53/TLR2/IRGM/MEFV/BST2/GBP5 | 6 |
| BP | GO:0031648 | | protein destabilization | 4/97 | 46/18723 | 9.35E-05 | 0.000826 | 0.000483 | SIRT1/HDAC6/MDM2/IRGM | 4 |
| BP | GO:0035094 | | response to nicotine | 4/97 | 46/18723 | 9.35E-05 | 0.000826 | 0.000483 | BCL2/NFKB1/CASP3/TNF | 4 |
| BP | GO:1900087 | | positive regulation of G1/S transition of mitotic cell cycle | 4/97 | 46/18723 | 9.35E-05 | 0.000826 | 0.000483 | EGFR/DDX3X/MDM2/AKT1 | 4 |
| MF | GO:1901981 | | phosphatidylinositol phosphate binding | 7/97 | 173/18368 | 3.67E-05 | 0.000838 | 0.000652 | GSDME/AKT1/GSDMD/GSDMA/ANXA2/GSDMB/GSDMC | 7 |
| BP | GO:0010657 | | muscle cell apoptotic process | 5/97 | 89/18723 | 9.90E-05 | 0.000872 | 0.00051 | SIRT1/PTEN/NFE2L2/TP53/BNIP3 | 5 |
| BP | GO:0022411 | | cellular component disassembly | 10/97 | 443/18723 | 9.96E-05 | 0.000875 | 0.000511 | CTSG/GPER1/STING1/HDAC6/TP53/IRGM/TREM2/BNIP3/TNF/CTSV | 10 |
| BP | GO:0062013 | | positive regulation of small molecule metabolic process | 6/97 | 143/18723 | 9.98E-05 | 0.000875 | 0.000511 | P2RX7/SIRT1/GPER1/AKT1/TREM2/TNF | 6 |
| BP | GO:0043618 | | regulation of transcription from RNA polymerase II promoter in response to stress | 4/97 | 47/18723 | 0.000102 | 0.000887 | 0.000519 | JUN/NFE2L2/SESN2/TP53 | 4 |
| BP | GO:0046677 | | response to antibiotic | 4/97 | 47/18723 | 0.000102 | 0.000887 | 0.000519 | MDM2/CASP8/TP53/CASP3 | 4 |
| BP | GO:0010663 | | positive regulation of striated muscle cell apoptotic process | 3/97 | 18/18723 | 0.000104 | 0.000894 | 0.000522 | PTEN/TP53/BNIP3 | 3 |
| BP | GO:0010666 | | positive regulation of cardiac muscle cell apoptotic process | 3/97 | 18/18723 | 0.000104 | 0.000894 | 0.000522 | PTEN/TP53/BNIP3 | 3 |
| BP | GO:0060546 | | negative regulation of necroptotic process | 3/97 | 18/18723 | 0.000104 | 0.000894 | 0.000522 | BIRC2/FADD/CASP8 | 3 |
| BP | GO:0071360 | | cellular response to exogenous dsRNA | 3/97 | 18/18723 | 0.000104 | 0.000894 | 0.000522 | STING1/DHX9/IRF3 | 3 |
| BP | GO:0150078 | | positive regulation of neuroinflammatory response | 3/97 | 18/18723 | 0.000104 | 0.000894 | 0.000522 | TREM2/IL18/TNF | 3 |
| BP | GO:0034644 | | cellular response to UV | 5/97 | 90/18723 | 0.000104 | 0.000894 | 0.000522 | SIRT1/METTL3/MDM2/TP53/PARP1 | 5 |
| BP | GO:1904705 | | regulation of vascular associated smooth muscle cell proliferation | 5/97 | 90/18723 | 0.000104 | 0.000894 | 0.000522 | JUN/GPER1/PTEN/MDM2/TNF | 5 |
| BP | GO:1990874 | | vascular associated smooth muscle cell proliferation | 5/97 | 91/18723 | 0.00011 | 0.000939 | 0.000549 | JUN/GPER1/PTEN/MDM2/TNF | 5 |
| BP | GO:0010675 | | regulation of cellular carbohydrate metabolic process | 6/97 | 146/18723 | 0.000112 | 0.000953 | 0.000557 | SIRT1/GPER1/SESN2/AKT1/TP53/MST1 | 6 |
| BP | GO:0014074 | | response to purine-containing compound | 6/97 | 148/18723 | 0.000121 | 0.001022 | 0.000597 | P2RX7/JUN/PTEN/EEF2K/BIRC2/PANX1 | 6 |
| BP | GO:1903900 | | regulation of viral life cycle | 6/97 | 148/18723 | 0.000121 | 0.001022 | 0.000597 | BCL2/IFI16/DDX3X/CXCL8/BST2/TNF | 6 |
| BP | GO:0032088 | | negative regulation of NF-kappaB transcription factor activity | 5/97 | 93/18723 | 0.000122 | 0.001025 | 0.000599 | SIRT1/CARD8/NLRP3/PYCARD/AIM2 | 5 |
| BP | GO:0106027 | | neuron projection organization | 5/97 | 93/18723 | 0.000122 | 0.001025 | 0.000599 | PTEN/EEF2K/HDAC6/APOE/TREM2 | 5 |
| BP | GO:1901796 | | regulation of signal transduction by p53 class mediator | 5/97 | 93/18723 | 0.000122 | 0.001025 | 0.000599 | BCL2/SIRT1/MDM2/AKT1/TP53 | 5 |
| BP | GO:0062099 | | negative regulation of programmed necrotic cell death | 3/97 | 19/18723 | 0.000123 | 0.001029 | 0.000602 | BIRC2/FADD/CASP8 | 3 |
| BP | GO:1900221 | | regulation of amyloid-beta clearance | 3/97 | 19/18723 | 0.000123 | 0.001029 | 0.000602 | APOE/TREM2/TNF | 3 |
| BP | GO:0000082 | | G1/S transition of mitotic cell cycle | 7/97 | 214/18723 | 0.000124 | 0.001037 | 0.000606 | BCL2/PTEN/EGFR/DDX3X/MDM2/AKT1/TP53 | 7 |
| BP | GO:0032368 | | regulation of lipid transport | 6/97 | 149/18723 | 0.000125 | 0.001041 | 0.000609 | SIRT1/NFKB1/AKT1/APOE/TREM2/ANXA2 | 6 |
| BP | GO:0046651 | | lymphocyte proliferation | 8/97 | 288/18723 | 0.000126 | 0.001045 | 0.000611 | BCL2/FADD/GLMN/TP53/RIPK3/CASP3/PYCARD/IL18 | 8 |
| BP | GO:0051702 | | biological process involved in interaction with symbiont | 5/97 | 94/18723 | 0.000128 | 0.001062 | 0.000621 | CTSG/JUN/APOE/APOL1/CAMP | 5 |
| BP | GO:0010665 | | regulation of cardiac muscle cell apoptotic process | 4/97 | 50/18723 | 0.00013 | 0.001068 | 0.000624 | PTEN/NFE2L2/TP53/BNIP3 | 4 |
| BP | GO:0060337 | | type I interferon signaling pathway | 4/97 | 50/18723 | 0.00013 | 0.001068 | 0.000624 | METTL3/FADD/IRF3/ZBP1 | 4 |
| BP | GO:0070231 | | T cell apoptotic process | 4/97 | 50/18723 | 0.00013 | 0.001068 | 0.000624 | FADD/AKT1/TP53/RIPK3 | 4 |
| BP | GO:0034504 | | protein localization to nucleus | 8/97 | 290/18723 | 0.000132 | 0.001082 | 0.000633 | TXNIP/ELAVL1/MDM2/SESN2/AKT1/ORMDL3/TP53/PARP1 | 8 |
| BP | GO:0032943 | | mononuclear cell proliferation | 8/97 | 291/18723 | 0.000135 | 0.001105 | 0.000646 | BCL2/FADD/GLMN/TP53/RIPK3/CASP3/PYCARD/IL18 | 8 |
| CC | GO:0034774 | | secretory granule lumen | 9/97 | 322/19550 | 3.20E-05 | 0.001115 | 0.000905 | CTSG/NFKB1/DDX3X/GSDMD/SERPINB1/PYCARD/ANXA2/CHI3L1/CAMP | 9 |
| CC | GO:0060205 | | cytoplasmic vesicle lumen | 9/97 | 325/19550 | 3.44E-05 | 0.001115 | 0.000905 | CTSG/NFKB1/DDX3X/GSDMD/SERPINB1/PYCARD/ANXA2/CHI3L1/CAMP | 9 |
| BP | GO:0030098 | | lymphocyte differentiation | 9/97 | 374/18723 | 0.00014 | 0.001142 | 0.000667 | BCL2/PRDM1/NLRP3/METTL3/FADD/TP53/RIPK3/IL18/IL36B | 9 |
| BP | GO:0002718 | | regulation of cytokine production involved in immune response | 5/97 | 96/18723 | 0.000142 | 0.001153 | 0.000674 | SIRT1/NLRP3/BST2/IL18/TNF | 5 |
| BP | GO:2001020 | | regulation of response to DNA damage stimulus | 7/97 | 219/18723 | 0.000143 | 0.001162 | 0.000679 | BCL2/SIRT1/EGFR/DHX9/MDM2/TP53/PARP1 | 7 |
| BP | GO:0046931 | | pore complex assembly | 3/97 | 20/18723 | 0.000144 | 0.001162 | 0.000679 | ANO6/P2RX7/GSDMD | 3 |
| BP | GO:1902176 | | negative regulation of oxidative stress-induced intrinsic apoptotic signaling pathway | 3/97 | 20/18723 | 0.000144 | 0.001162 | 0.000679 | SIRT1/NFE2L2/AKT1 | 3 |
| BP | GO:2000479 | | regulation of cAMP-dependent protein kinase activity | 3/97 | 20/18723 | 0.000144 | 0.001162 | 0.000679 | SIRT1/SESN2/MST1 | 3 |
| BP | GO:0010662 | | regulation of striated muscle cell apoptotic process | 4/97 | 52/18723 | 0.000151 | 0.001212 | 0.000709 | PTEN/NFE2L2/TP53/BNIP3 | 4 |
| BP | GO:0071357 | | cellular response to type I interferon | 4/97 | 52/18723 | 0.000151 | 0.001212 | 0.000709 | METTL3/FADD/IRF3/ZBP1 | 4 |
| BP | GO:1903050 | | regulation of proteolysis involved in cellular protein catabolic process | 7/97 | 221/18723 | 0.000152 | 0.001212 | 0.000709 | PTEN/NFE2L2/GLMN/MDM2/AKT1/APOE/TREM2 | 7 |
| BP | GO:0050890 | | cognition | 8/97 | 296/18723 | 0.000152 | 0.001212 | 0.000709 | JUN/PTEN/EGFR/TLR2/CASP3/APOE/TREM2/TNF | 8 |
| BP | GO:1903364 | | positive regulation of cellular protein catabolic process | 6/97 | 155/18723 | 0.000155 | 0.001236 | 0.000723 | PTEN/NFE2L2/MDM2/AKT1/APOE/TREM2 | 6 |
| BP | GO:0002367 | | cytokine production involved in immune response | 5/97 | 98/18723 | 0.000156 | 0.00124 | 0.000725 | SIRT1/NLRP3/BST2/IL18/TNF | 5 |
| MF | GO:0043028 | | cysteine-type endopeptidase regulator activity involved in apoptotic process | 4/97 | 40/18368 | 5.77E-05 | 0.001249 | 0.000972 | NLRP1/CARD8/BIRC2/PYCARD | 4 |
| BP | GO:0002274 | | myeloid leukocyte activation | 7/97 | 223/18723 | 0.00016 | 0.001271 | 0.000743 | TLR2/CXCL8/PYCARD/TREM2/IL18/IL13RA2/TNF | 7 |
| BP | GO:0051051 | | negative regulation of transport | 10/97 | 470/18723 | 0.000161 | 0.001277 | 0.000746 | BCL2/PTEN/NFKB1/GSTO1/AKT1/TLR2/APOE/BST2/IL13RA2/TNF | 10 |
| BP | GO:1901990 | | regulation of mitotic cell cycle phase transition | 8/97 | 299/18723 | 0.000163 | 0.001278 | 0.000747 | BCL2/PTEN/EGFR/DDX3X/MRE11/MDM2/AKT1/TP53 | 8 |
| BP | GO:0034381 | | plasma lipoprotein particle clearance | 4/97 | 53/18723 | 0.000163 | 0.001278 | 0.000747 | APOE/TREM2/ANXA2/FGF21 | 4 |
| BP | GO:0043620 | | regulation of DNA-templated transcription in response to stress | 4/97 | 53/18723 | 0.000163 | 0.001278 | 0.000747 | JUN/NFE2L2/SESN2/TP53 | 4 |
| BP | GO:1990090 | | cellular response to nerve growth factor stimulus | 4/97 | 53/18723 | 0.000163 | 0.001278 | 0.000747 | PTEN/FOXO3/EEF2K/AKT1 | 4 |
| BP | GO:2001234 | | negative regulation of apoptotic signaling pathway | 7/97 | 224/18723 | 0.000165 | 0.001288 | 0.000753 | BCL2/SIRT1/NFE2L2/DDX3X/MDM2/AKT1/TNF | 7 |
| BP | GO:0000002 | | mitochondrial genome maintenance | 3/97 | 21/18723 | 0.000168 | 0.001297 | 0.000758 | SESN2/TP53/PARP1 | 3 |
| BP | GO:0007252 | | I-kappaB phosphorylation | 3/97 | 21/18723 | 0.000168 | 0.001297 | 0.000758 | AKT1/TLR2/TNF | 3 |
| BP | GO:0010988 | | regulation of low-density lipoprotein particle clearance | 3/97 | 21/18723 | 0.000168 | 0.001297 | 0.000758 | TREM2/ANXA2/FGF21 | 3 |
| BP | GO:1902254 | | negative regulation of intrinsic apoptotic signaling pathway by p53 class mediator | 3/97 | 21/18723 | 0.000168 | 0.001297 | 0.000758 | BCL2/SIRT1/MDM2 | 3 |
| BP | GO:0050670 | | regulation of lymphocyte proliferation | 7/97 | 225/18723 | 0.000169 | 0.001308 | 0.000765 | BCL2/FADD/GLMN/RIPK3/CASP3/PYCARD/IL18 | 7 |
| BP | GO:0042063 | | gliogenesis | 8/97 | 301/18723 | 0.00017 | 0.001312 | 0.000767 | PTEN/EGFR/METTL3/AKT1/TLR2/TREM2/BNIP3/TNF | 8 |
| BP | GO:0045786 | | negative regulation of cell cycle | 9/97 | 385/18723 | 0.000174 | 0.001335 | 0.00078 | BCL2/GPER1/PTEN/EGFR/MRE11/MDM2/TP53/CASP3/TNF | 9 |
| BP | GO:0071241 | | cellular response to inorganic substance | 7/97 | 226/18723 | 0.000174 | 0.001335 | 0.00078 | JUN/EGFR/NFE2L2/EEF2K/AKT1/PARP1/BNIP3 | 7 |
| BP | GO:0010659 | | cardiac muscle cell apoptotic process | 4/97 | 54/18723 | 0.000176 | 0.00134 | 0.000783 | PTEN/NFE2L2/TP53/BNIP3 | 4 |
| BP | GO:1902893 | | regulation of pri-miRNA transcription by RNA polymerase II | 4/97 | 54/18723 | 0.000176 | 0.00134 | 0.000783 | JUN/FOXO3/TP53/TNF | 4 |
| BP | GO:0045860 | | positive regulation of protein kinase activity | 9/97 | 386/18723 | 0.000177 | 0.00135 | 0.000789 | SIRT1/EGFR/DDX3X/AKT1/RIPK3/IRGM/CHI3L1/IL18/TNF | 9 |
| BP | GO:0032944 | | regulation of mononuclear cell proliferation | 7/97 | 227/18723 | 0.000179 | 0.001356 | 0.000793 | BCL2/FADD/GLMN/RIPK3/CASP3/PYCARD/IL18 | 7 |
| BP | GO:0046777 | | protein autophosphorylation | 7/97 | 227/18723 | 0.000179 | 0.001356 | 0.000793 | EGFR/DDX3X/MRE11/EEF2K/AKT1/RIPK3/ALK | 7 |
| MF | GO:0008234 | | cysteine-type peptidase activity | 7/97 | 190/18368 | 6.65E-05 | 0.001366 | 0.001064 | CASP8/CAPN1/CASP3/PYCARD/CASP6/CASP5/CTSV | 7 |
| BP | GO:0043331 | | response to dsRNA | 4/97 | 55/18723 | 0.000189 | 0.001423 | 0.000832 | STING1/NFKB1/DHX9/IRF3 | 4 |
| BP | GO:0061614 | | pri-miRNA transcription by RNA polymerase II | 4/97 | 55/18723 | 0.000189 | 0.001423 | 0.000832 | JUN/FOXO3/TP53/TNF | 4 |
| BP | GO:0032727 | | positive regulation of interferon-alpha production | 3/97 | 22/18723 | 0.000193 | 0.001449 | 0.000847 | DDX3X/DHX9/IRF3 | 3 |
| BP | GO:0060965 | | negative regulation of gene silencing by miRNA | 3/97 | 22/18723 | 0.000193 | 0.001449 | 0.000847 | ELAVL1/TP53/TNF | 3 |
| BP | GO:1901522 | | positive regulation of transcription from RNA polymerase II promoter involved in cellular response to chemical stimulus | 3/97 | 22/18723 | 0.000193 | 0.001449 | 0.000847 | NFE2L2/SESN2/TP53 | 3 |
| BP | GO:0016049 | | cell growth | 10/97 | 482/18723 | 0.000198 | 0.00148 | 0.000865 | BCL2/SIRT1/EGFR/DDX3X/HDAC6/SESN2/AKT1/TP53/APOE/BST2 | 10 |
| MF | GO:0061135 | | endopeptidase regulator activity | 7/97 | 194/18368 | 7.58E-05 | 0.001483 | 0.001155 | NLRP1/CARD8/BIRC2/SERPINB1/ANXA2/BST2/NLRP7 | 7 |
| BP | GO:0045732 | | positive regulation of protein catabolic process | 7/97 | 231/18723 | 0.000199 | 0.001485 | 0.000868 | PTEN/NFE2L2/MDM2/AKT1/APOE/TREM2/TNF | 7 |
| BP | GO:0010332 | | response to gamma radiation | 4/97 | 56/18723 | 0.000202 | 0.001499 | 0.000876 | BCL2/MDM2/TP53/PARP1 | 4 |
| BP | GO:0010658 | | striated muscle cell apoptotic process | 4/97 | 56/18723 | 0.000202 | 0.001499 | 0.000876 | PTEN/NFE2L2/TP53/BNIP3 | 4 |
| BP | GO:1990089 | | response to nerve growth factor | 4/97 | 56/18723 | 0.000202 | 0.001499 | 0.000876 | PTEN/FOXO3/EEF2K/AKT1 | 4 |
| BP | GO:1903076 | | regulation of protein localization to plasma membrane | 5/97 | 104/18723 | 0.000206 | 0.001526 | 0.000892 | GPER1/EGFR/AKT1/TREM2/TNF | 5 |
| BP | GO:0002700 | | regulation of production of molecular mediator of immune response | 6/97 | 164/18723 | 0.000211 | 0.001557 | 0.00091 | SIRT1/NLRP3/BST2/IL18/IL13RA2/TNF | 6 |
| MF | GO:0004175 | | endopeptidase activity | 10/97 | 430/18368 | 9.12E-05 | 0.001576 | 0.001227 | CTSG/CASP8/CAPN1/CASP3/PYCARD/ACE2/GZMB/CASP6/CASP5/CTSV | 10 |
| MF | GO:0005149 | | interleukin-1 receptor binding | 3/97 | 17/18368 | 9.20E-05 | 0.001576 | 0.001227 | IL1RN/IL36B/IL36G | 3 |
| MF | GO:0008656 | | cysteine-type endopeptidase activator activity involved in apoptotic process | 3/97 | 17/18368 | 9.20E-05 | 0.001576 | 0.001227 | NLRP1/CARD8/PYCARD | 3 |
| BP | GO:0040029 | | regulation of gene expression, epigenetic | 5/97 | 105/18723 | 0.000216 | 0.001588 | 0.000929 | IFI16/SIRT1/METTL3/GLMN/HDAC6 | 5 |
| BP | GO:0010883 | | regulation of lipid storage | 4/97 | 57/18723 | 0.000217 | 0.001592 | 0.00093 | SIRT1/NFKB1/TREM2/TNF | 4 |
| BP | GO:0019932 | | second-messenger-mediated signaling | 8/97 | 312/18723 | 0.000217 | 0.001593 | 0.000931 | P2RX7/EGFR/GSTO1/CXCL8/IRGM/APOE/TREM2/TNF | 8 |
| BP | GO:0002699 | | positive regulation of immune effector process | 7/97 | 235/18723 | 0.000221 | 0.001612 | 0.000943 | SIRT1/NLRP3/FADD/TREM2/IL18/IL13RA2/TNF | 7 |
| BP | GO:0045930 | | negative regulation of mitotic cell cycle | 7/97 | 235/18723 | 0.000221 | 0.001612 | 0.000943 | BCL2/PTEN/EGFR/MRE11/MDM2/TP53/TNF | 7 |
| BP | GO:0060547 | | negative regulation of necrotic cell death | 3/97 | 23/18723 | 0.000221 | 0.001612 | 0.000943 | BIRC2/FADD/CASP8 | 3 |
| BP | GO:0034340 | | response to type I interferon | 4/97 | 58/18723 | 0.000232 | 0.001672 | 0.000978 | METTL3/FADD/IRF3/ZBP1 | 4 |
| BP | GO:1902808 | | positive regulation of cell cycle G1/S phase transition | 4/97 | 58/18723 | 0.000232 | 0.001672 | 0.000978 | EGFR/DDX3X/MDM2/AKT1 | 4 |
| BP | GO:2001244 | | positive regulation of intrinsic apoptotic signaling pathway | 4/97 | 58/18723 | 0.000232 | 0.001672 | 0.000978 | GSDME/SIRT1/TP53/RIPK3 | 4 |
| BP | GO:0018105 | | peptidyl-serine phosphorylation | 8/97 | 315/18723 | 0.000232 | 0.001672 | 0.000978 | BCL2/PTEN/EGFR/HDAC6/AKT1/IRGM/MST1/TNF | 8 |
| BP | GO:0034767 | | positive regulation of ion transmembrane transport | 6/97 | 167/18723 | 0.000233 | 0.001672 | 0.000978 | ANO6/P2RX7/GPER1/GSTO1/TREM2/ACE2 | 6 |
| BP | GO:1903034 | | regulation of response to wounding | 6/97 | 167/18723 | 0.000233 | 0.001672 | 0.000978 | ANO6/PTEN/NFE2L2/APOE/ANXA2/TNF | 6 |
| BP | GO:0002824 | | positive regulation of adaptive immune response based on somatic recombination of immune receptors built from immunoglobulin superfamily domains | 5/97 | 107/18723 | 0.000236 | 0.00169 | 0.000988 | NLRP3/FADD/TREM2/IL18/TNF | 5 |
| BP | GO:0002706 | | regulation of lymphocyte mediated immunity | 6/97 | 168/18723 | 0.00024 | 0.001716 | 0.001003 | NLRP3/FADD/RIPK3/TREM2/IL18/TNF | 6 |
| BP | GO:0002822 | | regulation of adaptive immune response based on somatic recombination of immune receptors built from immunoglobulin superfamily domains | 6/97 | 168/18723 | 0.00024 | 0.001716 | 0.001003 | NLRP3/FADD/RIPK3/TREM2/IL18/TNF | 6 |
| BP | GO:1903039 | | positive regulation of leukocyte cell-cell adhesion | 7/97 | 239/18723 | 0.000245 | 0.001746 | 0.001021 | NLRP3/FADD/AKT1/PYCARD/IL18/TNF/IL36B | 7 |
| BP | GO:0046324 | | regulation of glucose import | 4/97 | 59/18723 | 0.000248 | 0.001757 | 0.001027 | NFE2L2/AKT1/TNF/FGF21 | 4 |
| BP | GO:0051353 | | positive regulation of oxidoreductase activity | 4/97 | 59/18723 | 0.000248 | 0.001757 | 0.001027 | AKT1/RIPK3/APOE/TNF | 4 |
| BP | GO:0033032 | | regulation of myeloid cell apoptotic process | 3/97 | 24/18723 | 0.000252 | 0.001766 | 0.001032 | BCL2/SIRT1/PTEN | 3 |
| BP | GO:0036003 | | positive regulation of transcription from RNA polymerase II promoter in response to stress | 3/97 | 24/18723 | 0.000252 | 0.001766 | 0.001032 | NFE2L2/SESN2/TP53 | 3 |
| BP | GO:0051000 | | positive regulation of nitric-oxide synthase activity | 3/97 | 24/18723 | 0.000252 | 0.001766 | 0.001032 | AKT1/APOE/TNF | 3 |
| BP | GO:0060149 | | negative regulation of posttranscriptional gene silencing | 3/97 | 24/18723 | 0.000252 | 0.001766 | 0.001032 | ELAVL1/TP53/TNF | 3 |
| BP | GO:0060967 | | negative regulation of gene silencing by RNA | 3/97 | 24/18723 | 0.000252 | 0.001766 | 0.001032 | ELAVL1/TP53/TNF | 3 |
| BP | GO:2000353 | | positive regulation of endothelial cell apoptotic process | 3/97 | 24/18723 | 0.000252 | 0.001766 | 0.001032 | ANO6/GPER1/FOXO3 | 3 |
| BP | GO:0044843 | | cell cycle G1/S phase transition | 7/97 | 241/18723 | 0.000258 | 0.001802 | 0.001053 | BCL2/PTEN/EGFR/DDX3X/MDM2/AKT1/TP53 | 7 |
| MF | GO:0042834 | | peptidoglycan binding | 3/97 | 18/18368 | 0.00011 | 0.001808 | 0.001408 | NLRP3/TLR2/TREM2 | 3 |
| BP | GO:0031638 | | zymogen activation | 4/97 | 60/18723 | 0.000264 | 0.00184 | 0.001076 | IFI16/FADD/CASP8/PYCARD | 4 |
| BP | GO:0042129 | | regulation of T cell proliferation | 6/97 | 171/18723 | 0.000264 | 0.00184 | 0.001076 | FADD/GLMN/RIPK3/CASP3/PYCARD/IL18 | 6 |
| BP | GO:0001503 | | ossification | 9/97 | 408/18723 | 0.000267 | 0.001853 | 0.001083 | BCL2/ANO6/P2RX7/EGFR/DHX9/AKT1/CLEC5A/TP63/TNF | 9 |
| BP | GO:1904659 | | glucose transmembrane transport | 5/97 | 110/18723 | 0.000268 | 0.001853 | 0.001083 | NFE2L2/SESN2/AKT1/TNF/FGF21 | 5 |
| BP | GO:1905954 | | positive regulation of lipid localization | 5/97 | 110/18723 | 0.000268 | 0.001853 | 0.001083 | SIRT1/NFKB1/APOE/TREM2/ANXA2 | 5 |
| BP | GO:0042770 | | signal transduction in response to DNA damage | 6/97 | 172/18723 | 0.000273 | 0.001883 | 0.001101 | SIRT1/FOXO3/MRE11/MDM2/SESN2/TP53 | 6 |
| BP | GO:0050994 | | regulation of lipid catabolic process | 4/97 | 61/18723 | 0.000282 | 0.001936 | 0.001132 | AKT1/TNF/ALK/FGF21 | 4 |
| BP | GO:2000401 | | regulation of lymphocyte migration | 4/97 | 61/18723 | 0.000282 | 0.001936 | 0.001132 | FADD/AKT1/RIPK3/PYCARD | 4 |
| BP | GO:0048143 | | astrocyte activation | 3/97 | 25/18723 | 0.000285 | 0.001946 | 0.001137 | EGFR/TREM2/TNF | 3 |
| BP | GO:0050995 | | negative regulation of lipid catabolic process | 3/97 | 25/18723 | 0.000285 | 0.001946 | 0.001137 | AKT1/TNF/ALK | 3 |
| BP | GO:0098581 | | detection of external biotic stimulus | 3/97 | 25/18723 | 0.000285 | 0.001946 | 0.001137 | LY96/TLR2/TREM2 | 3 |
| BP | GO:1903798 | | regulation of production of miRNAs involved in gene silencing by miRNA | 3/97 | 25/18723 | 0.000285 | 0.001946 | 0.001137 | EGFR/TP53/TNF | 3 |
| BP | GO:0001558 | | regulation of cell growth | 9/97 | 414/18723 | 0.000297 | 0.002024 | 0.001183 | BCL2/SIRT1/EGFR/DDX3X/SESN2/AKT1/TP53/APOE/BST2 | 9 |
| BP | GO:0051235 | | maintenance of location | 8/97 | 327/18723 | 0.000298 | 0.002024 | 0.001183 | SIRT1/GPER1/NFKB1/GSTO1/AKT1/APOE/TREM2/TNF | 8 |
| BP | GO:0048771 | | tissue remodeling | 6/97 | 175/18723 | 0.000299 | 0.002024 | 0.001183 | P2RX7/EGFR/MDM2/CAPN1/TP53/IL18 | 6 |
| BP | GO:1905475 | | regulation of protein localization to membrane | 6/97 | 175/18723 | 0.000299 | 0.002024 | 0.001183 | GPER1/EGFR/AKT1/TREM2/GZMB/TNF | 6 |
| MF | GO:0032813 | | tumor necrosis factor receptor superfamily binding | 4/97 | 49/18368 | 0.000129 | 0.00204 | 0.001588 | FADD/CASP8/CASP3/TNF | 4 |
| BP | GO:0002708 | | positive regulation of lymphocyte mediated immunity | 5/97 | 113/18723 | 0.000304 | 0.002048 | 0.001198 | NLRP3/FADD/TREM2/IL18/TNF | 5 |
| BP | GO:0050728 | | negative regulation of inflammatory response | 6/97 | 176/18723 | 0.000309 | 0.002078 | 0.001215 | GPER1/NFKB1/NLRP3/APOE/TREM2/MEFV | 6 |
| BP | GO:0045926 | | negative regulation of growth | 7/97 | 249/18723 | 0.000314 | 0.002112 | 0.001235 | BCL2/SIRT1/PTEN/DDX3X/SESN2/TP53/BST2 | 7 |
| BP | GO:0008645 | | hexose transmembrane transport | 5/97 | 114/18723 | 0.000316 | 0.002116 | 0.001237 | NFE2L2/SESN2/AKT1/TNF/FGF21 | 5 |
| BP | GO:0046660 | | female sex differentiation | 5/97 | 114/18723 | 0.000316 | 0.002116 | 0.001237 | BCL2/SIRT1/FOXO3/CASP3/TP63 | 5 |
| BP | GO:0046902 | | regulation of mitochondrial membrane permeability | 4/97 | 63/18723 | 0.000319 | 0.002131 | 0.001246 | BCL2/TP53/BNIP3/GZMB | 4 |
| BP | GO:0010875 | | positive regulation of cholesterol efflux | 3/97 | 26/18723 | 0.000321 | 0.002138 | 0.00125 | SIRT1/APOE/TREM2 | 3 |
| BP | GO:0070920 | | regulation of production of small RNA involved in gene silencing by RNA | 3/97 | 26/18723 | 0.000321 | 0.002138 | 0.00125 | EGFR/TP53/TNF | 3 |
| BP | GO:0015749 | | monosaccharide transmembrane transport | 5/97 | 116/18723 | 0.000343 | 0.002271 | 0.001327 | NFE2L2/SESN2/AKT1/TNF/FGF21 | 5 |
| BP | GO:0030518 | | intracellular steroid hormone receptor signaling pathway | 5/97 | 116/18723 | 0.000343 | 0.002271 | 0.001327 | SIRT1/GPER1/HDAC6/PARP1/TP63 | 5 |
| BP | GO:0043409 | | negative regulation of MAPK cascade | 6/97 | 180/18723 | 0.000348 | 0.002302 | 0.001346 | P2RX7/GPER1/PTEN/AKT1/APOE/TREM2 | 6 |
| MF | GO:0016505 | | peptidase activator activity involved in apoptotic process | 3/97 | 20/18368 | 0.000152 | 0.002321 | 0.001807 | NLRP1/CARD8/PYCARD | 3 |
| BP | GO:0030301 | | cholesterol transport | 5/97 | 117/18723 | 0.000357 | 0.002353 | 0.001376 | SIRT1/NFKB1/APOE/TREM2/ANXA2 | 5 |
| BP | GO:0071677 | | positive regulation of mononuclear cell migration | 4/97 | 65/18723 | 0.00036 | 0.002363 | 0.001382 | ANO6/FADD/PYCARD/TNF | 4 |
| BP | GO:0042104 | | positive regulation of activated T cell proliferation | 3/97 | 27/18723 | 0.00036 | 0.002363 | 0.001382 | FADD/PYCARD/IL18 | 3 |
| BP | GO:1903205 | | regulation of hydrogen peroxide-induced cell death | 3/97 | 27/18723 | 0.00036 | 0.002363 | 0.001382 | NFE2L2/FOXO3/HDAC6 | 3 |
| BP | GO:0007611 | | learning or memory | 7/97 | 255/18723 | 0.000363 | 0.002375 | 0.001389 | JUN/PTEN/EGFR/TLR2/CASP3/APOE/TREM2 | 7 |
| BP | GO:0034219 | | carbohydrate transmembrane transport | 5/97 | 118/18723 | 0.000371 | 0.002423 | 0.001416 | NFE2L2/SESN2/AKT1/TNF/FGF21 | 5 |
| BP | GO:0005996 | | monosaccharide metabolic process | 7/97 | 257/18723 | 0.00038 | 0.002473 | 0.001446 | SIRT1/GSTO1/SESN2/AKT1/TP53/MST1/TNF | 7 |
| BP | GO:0030193 | | regulation of blood coagulation | 4/97 | 66/18723 | 0.000381 | 0.002473 | 0.001446 | ANO6/NFE2L2/APOE/ANXA2 | 4 |
| BP | GO:0050766 | | positive regulation of phagocytosis | 4/97 | 66/18723 | 0.000381 | 0.002473 | 0.001446 | ANO6/PYCARD/TREM2/TNF | 4 |
| BP | GO:1905953 | | negative regulation of lipid localization | 4/97 | 66/18723 | 0.000381 | 0.002473 | 0.001446 | NFKB1/AKT1/TREM2/TNF | 4 |
| BP | GO:0010906 | | regulation of glucose metabolic process | 5/97 | 119/18723 | 0.000385 | 0.002494 | 0.001458 | SIRT1/SESN2/AKT1/TP53/MST1 | 5 |
| MF | GO:0005543 | | phospholipid binding | 10/97 | 466/18368 | 0.000176 | 0.002512 | 0.001955 | GSDME/AKT1/GSDMD/CPTP/GSDMA/APOE/TREM2/ANXA2/GSDMB/GSDMC | 10 |
| MF | GO:0005123 | | death receptor binding | 3/97 | 21/18368 | 0.000177 | 0.002512 | 0.001955 | FADD/CASP8/CASP3 | 3 |
| BP | GO:0042593 | | glucose homeostasis | 7/97 | 258/18723 | 0.000389 | 0.002513 | 0.001469 | SIRT1/GPER1/FOXO3/SESN2/AKT1/TREM2/FGF21 | 7 |
| BP | GO:0033500 | | carbohydrate homeostasis | 7/97 | 259/18723 | 0.000398 | 0.002567 | 0.001501 | SIRT1/GPER1/FOXO3/SESN2/AKT1/TREM2/FGF21 | 7 |
| BP | GO:0002761 | | regulation of myeloid leukocyte differentiation | 5/97 | 120/18723 | 0.000401 | 0.002571 | 0.001503 | JUN/FADD/CASP8/TREM2/TNF | 5 |
| BP | GO:0032607 | | interferon-alpha production | 3/97 | 28/18723 | 0.000402 | 0.002571 | 0.001503 | DDX3X/DHX9/IRF3 | 3 |
| BP | GO:0032647 | | regulation of interferon-alpha production | 3/97 | 28/18723 | 0.000402 | 0.002571 | 0.001503 | DDX3X/DHX9/IRF3 | 3 |
| BP | GO:0032801 | | receptor catabolic process | 3/97 | 28/18723 | 0.000402 | 0.002571 | 0.001503 | CAPN1/APOE/ANXA2 | 3 |
| BP | GO:0043123 | | positive regulation of I-kappaB kinase/NF-kappaB signaling | 6/97 | 186/18723 | 0.000414 | 0.002641 | 0.001544 | BIRC2/FADD/CASP8/IRF3/BST2/TNF | 6 |
| BP | GO:0071478 | | cellular response to radiation | 6/97 | 186/18723 | 0.000414 | 0.002641 | 0.001544 | IFI16/SIRT1/METTL3/MDM2/TP53/PARP1 | 6 |
| BP | GO:0030336 | | negative regulation of cell migration | 8/97 | 344/18723 | 0.000418 | 0.002659 | 0.001555 | BCL2/PTEN/NFE2L2/FOXO3/AKT1/APOE/BST2/TNF | 8 |
| BP | GO:0042391 | | regulation of membrane potential | 9/97 | 434/18723 | 0.00042 | 0.002663 | 0.001557 | BCL2/P2RX7/JUN/GPER1/PTEN/AKT1/PARP1/TREM2/BNIP3 | 9 |
| BP | GO:0061136 | | regulation of proteasomal protein catabolic process | 6/97 | 187/18723 | 0.000426 | 0.002683 | 0.001569 | NFE2L2/GLMN/MDM2/AKT1/APOE/TREM2 | 6 |
| BP | GO:0009636 | | response to toxic substance | 7/97 | 262/18723 | 0.000427 | 0.002683 | 0.001569 | BCL2/NFE2L2/GSTO1/MDM2/SESN2/TLR2/APOE | 7 |
| BP | GO:0031640 | | killing of cells of other organism | 4/97 | 68/18723 | 0.000428 | 0.002683 | 0.001569 | CTSG/P2RX7/APOL1/CAMP | 4 |
| BP | GO:0042698 | | ovulation cycle | 4/97 | 68/18723 | 0.000428 | 0.002683 | 0.001569 | SIRT1/EGFR/FOXO3/CASP3 | 4 |
| BP | GO:0061912 | | selective autophagy | 4/97 | 68/18723 | 0.000428 | 0.002683 | 0.001569 | STING1/HDAC6/SESN2/TP53 | 4 |
| BP | GO:1900046 | | regulation of hemostasis | 4/97 | 68/18723 | 0.000428 | 0.002683 | 0.001569 | ANO6/NFE2L2/APOE/ANXA2 | 4 |
| BP | GO:0048732 | | gland development | 9/97 | 436/18723 | 0.000434 | 0.002717 | 0.001588 | BCL2/PTEN/EGFR/FADD/AKT1/CAPN1/RIPK3/TP63/TNF | 9 |
| BP | GO:0030308 | | negative regulation of cell growth | 6/97 | 188/18723 | 0.000439 | 0.002742 | 0.001603 | BCL2/SIRT1/DDX3X/SESN2/TP53/BST2 | 6 |
| BP | GO:0032868 | | response to insulin | 7/97 | 264/18723 | 0.000447 | 0.002761 | 0.001614 | SIRT1/PTEN/EEF2K/SESN2/AKT1/TLR2/PARP1 | 7 |
| BP | GO:0010955 | | negative regulation of protein processing | 3/97 | 29/18723 | 0.000447 | 0.002761 | 0.001614 | CARD8/MDM2/NLRP7 | 3 |
| BP | GO:0051873 | | killing by host of symbiont cells | 3/97 | 29/18723 | 0.000447 | 0.002761 | 0.001614 | CTSG/APOL1/CAMP | 3 |
| BP | GO:0051883 | | killing of cells in other organism involved in symbiotic interaction | 3/97 | 29/18723 | 0.000447 | 0.002761 | 0.001614 | CTSG/APOL1/CAMP | 3 |
| BP | GO:0060969 | | negative regulation of gene silencing | 3/97 | 29/18723 | 0.000447 | 0.002761 | 0.001614 | ELAVL1/TP53/TNF | 3 |
| BP | GO:1903318 | | negative regulation of protein maturation | 3/97 | 29/18723 | 0.000447 | 0.002761 | 0.001614 | CARD8/MDM2/NLRP7 | 3 |
| BP | GO:0071482 | | cellular response to light stimulus | 5/97 | 123/18723 | 0.000449 | 0.002768 | 0.001618 | SIRT1/METTL3/MDM2/TP53/PARP1 | 5 |
| BP | GO:0050688 | | regulation of defense response to virus | 4/97 | 69/18723 | 0.000452 | 0.002779 | 0.001625 | STING1/DHX9/PYCARD/AIM2 | 4 |
| BP | GO:0050810 | | regulation of steroid biosynthetic process | 4/97 | 69/18723 | 0.000452 | 0.002779 | 0.001625 | SIRT1/NFKB1/APOE/TNF | 4 |
| BP | GO:0045936 | | negative regulation of phosphate metabolic process | 9/97 | 441/18723 | 0.000471 | 0.00289 | 0.00169 | SIRT1/PTEN/AKT1/TP53/CASP3/APOE/PARP1/PYCARD/TNF | 9 |
| BP | GO:0045123 | | cellular extravasation | 4/97 | 70/18723 | 0.000478 | 0.002925 | 0.00171 | PECAM1/FADD/RIPK3/TNF | 4 |
| BP | GO:0010563 | | negative regulation of phosphorus metabolic process | 9/97 | 442/18723 | 0.000479 | 0.002927 | 0.001711 | SIRT1/PTEN/AKT1/TP53/CASP3/APOE/PARP1/PYCARD/TNF | 9 |
| BP | GO:1904375 | | regulation of protein localization to cell periphery | 5/97 | 125/18723 | 0.000483 | 0.002947 | 0.001723 | GPER1/EGFR/AKT1/TREM2/TNF | 5 |
| BP | GO:0051960 | | regulation of nervous system development | 9/97 | 443/18723 | 0.000487 | 0.002964 | 0.001733 | GPER1/PTEN/EGFR/EEF2K/AKT1/TP53/TLR2/TREM2/TNF | 9 |
| BP | GO:0045948 | | positive regulation of translational initiation | 3/97 | 30/18723 | 0.000494 | 0.002995 | 0.001751 | DDX3X/METTL3/TNF | 3 |
| BP | GO:0060218 | | hematopoietic stem cell differentiation | 3/97 | 30/18723 | 0.000494 | 0.002995 | 0.001751 | NFE2L2/METTL3/TP53 | 3 |
| BP | GO:2000637 | | positive regulation of gene silencing by miRNA | 3/97 | 30/18723 | 0.000494 | 0.002995 | 0.001751 | EGFR/DHX9/TP53 | 3 |
| BP | GO:0050818 | | regulation of coagulation | 4/97 | 71/18723 | 0.000504 | 0.003048 | 0.001782 | ANO6/NFE2L2/APOE/ANXA2 | 4 |
| BP | GO:0071479 | | cellular response to ionizing radiation | 4/97 | 72/18723 | 0.000532 | 0.003209 | 0.001876 | IFI16/SIRT1/MDM2/TP53 | 4 |
| BP | GO:1904062 | | regulation of cation transmembrane transport | 8/97 | 357/18723 | 0.000534 | 0.003218 | 0.001881 | ANO6/GPER1/PTEN/GSTO1/AKT1/CAPN1/TREM2/ACE2 | 8 |
| BP | GO:0002230 | | positive regulation of defense response to virus by host | 3/97 | 31/18723 | 0.000545 | 0.003261 | 0.001907 | STING1/PYCARD/AIM2 | 3 |
| BP | GO:0036474 | | cell death in response to hydrogen peroxide | 3/97 | 31/18723 | 0.000545 | 0.003261 | 0.001907 | NFE2L2/FOXO3/HDAC6 | 3 |
| BP | GO:0060148 | | positive regulation of posttranscriptional gene silencing | 3/97 | 31/18723 | 0.000545 | 0.003261 | 0.001907 | EGFR/DHX9/TP53 | 3 |
| BP | GO:1901797 | | negative regulation of signal transduction by p53 class mediator | 3/97 | 31/18723 | 0.000545 | 0.003261 | 0.001907 | BCL2/SIRT1/MDM2 | 3 |
| BP | GO:0006006 | | glucose metabolic process | 6/97 | 196/18723 | 0.000547 | 0.003264 | 0.001908 | SIRT1/SESN2/AKT1/TP53/MST1/TNF | 6 |
| BP | GO:0051052 | | regulation of DNA metabolic process | 8/97 | 359/18723 | 0.000554 | 0.003297 | 0.001927 | NEK7/SIRT1/EGFR/MRE11/DHX9/AKT1/TP53/PARP1 | 8 |
| BP | GO:2000146 | | negative regulation of cell motility | 8/97 | 359/18723 | 0.000554 | 0.003297 | 0.001927 | BCL2/PTEN/NFE2L2/FOXO3/AKT1/APOE/BST2/TNF | 8 |
| BP | GO:0045739 | | positive regulation of DNA repair | 4/97 | 73/18723 | 0.00056 | 0.003327 | 0.001945 | SIRT1/EGFR/DHX9/PARP1 | 4 |
| BP | GO:0043270 | | positive regulation of ion transport | 7/97 | 275/18723 | 0.000569 | 0.003375 | 0.001973 | ANO6/P2RX7/GPER1/GSTO1/AKT1/TREM2/ACE2 | 7 |
| MF | GO:0005125 | | cytokine activity | 7/97 | 235/18368 | 0.000248 | 0.0034 | 0.002647 | CXCL8/IL18/IL32/TNF/IL1RN/IL36B/IL36G | 7 |
| BP | GO:0015918 | | sterol transport | 5/97 | 130/18723 | 0.000578 | 0.003418 | 0.001998 | SIRT1/NFKB1/APOE/TREM2/ANXA2 | 5 |
| BP | GO:0033143 | | regulation of intracellular steroid hormone receptor signaling pathway | 4/97 | 74/18723 | 0.00059 | 0.003479 | 0.002034 | SIRT1/HDAC6/PARP1/TP63 | 4 |
| BP | GO:0051881 | | regulation of mitochondrial membrane potential | 4/97 | 74/18723 | 0.00059 | 0.003479 | 0.002034 | BCL2/AKT1/PARP1/BNIP3 | 4 |
| BP | GO:0051098 | | regulation of binding | 8/97 | 363/18723 | 0.000596 | 0.003507 | 0.00205 | JUN/IFI16/STING1/DHX9/AKT1/APOE/PARP1/ANXA2 | 8 |
| BP | GO:0019835 | | cytolysis | 3/97 | 32/18723 | 0.0006 | 0.003515 | 0.002055 | GSDMB/APOL1/GZMB | 3 |
| BP | GO:1901889 | | negative regulation of cell junction assembly | 3/97 | 32/18723 | 0.0006 | 0.003515 | 0.002055 | PTEN/TLR2/TNF | 3 |
| BP | GO:0050767 | | regulation of neurogenesis | 8/97 | 364/18723 | 0.000607 | 0.003552 | 0.002077 | GPER1/PTEN/EGFR/EEF2K/TP53/TLR2/TREM2/TNF | 8 |
| BP | GO:0071902 | | positive regulation of protein serine/threonine kinase activity | 6/97 | 200/18723 | 0.000608 | 0.003553 | 0.002077 | SIRT1/EGFR/DDX3X/AKT1/IRGM/TNF | 6 |
| BP | GO:0007569 | | cell aging | 5/97 | 132/18723 | 0.000619 | 0.003609 | 0.00211 | BCL2/SIRT1/PTEN/TP53/TP63 | 5 |
| BP | GO:0033077 | | T cell differentiation in thymus | 4/97 | 75/18723 | 0.000621 | 0.003609 | 0.00211 | BCL2/FADD/TP53/RIPK3 | 4 |
| BP | GO:0034121 | | regulation of toll-like receptor signaling pathway | 4/97 | 75/18723 | 0.000621 | 0.003609 | 0.00211 | DDX3X/BIRC2/TLR2/TREM2 | 4 |
| BP | GO:0051271 | | negative regulation of cellular component movement | 8/97 | 367/18723 | 0.00064 | 0.003705 | 0.002166 | BCL2/PTEN/NFE2L2/FOXO3/AKT1/APOE/BST2/TNF | 8 |
| BP | GO:0016051 | | carbohydrate biosynthetic process | 6/97 | 202/18723 | 0.000641 | 0.003705 | 0.002166 | SIRT1/GPER1/NFKB1/SESN2/AKT1/MST1 | 6 |
| BP | GO:1903052 | | positive regulation of proteolysis involved in cellular protein catabolic process | 5/97 | 133/18723 | 0.000641 | 0.003705 | 0.002166 | PTEN/NFE2L2/MDM2/AKT1/TREM2 | 5 |
| BP | GO:0010259 | | multicellular organism aging | 3/97 | 33/18723 | 0.000657 | 0.003749 | 0.002192 | TP53/TP63/CTSV | 3 |
| BP | GO:0010984 | | regulation of lipoprotein particle clearance | 3/97 | 33/18723 | 0.000657 | 0.003749 | 0.002192 | TREM2/ANXA2/FGF21 | 3 |
| BP | GO:0033198 | | response to ATP | 3/97 | 33/18723 | 0.000657 | 0.003749 | 0.002192 | P2RX7/PTEN/PANX1 | 3 |
| BP | GO:0046685 | | response to arsenic-containing substance | 3/97 | 33/18723 | 0.000657 | 0.003749 | 0.002192 | PTEN/DDX3X/GSTO1 | 3 |
| BP | GO:1901099 | | negative regulation of signal transduction in absence of ligand | 3/97 | 33/18723 | 0.000657 | 0.003749 | 0.002192 | BCL2/AKT1/TNF | 3 |
| BP | GO:1903146 | | regulation of autophagy of mitochondrion | 3/97 | 33/18723 | 0.000657 | 0.003749 | 0.002192 | HDAC6/TP53/BNIP3 | 3 |
| BP | GO:2001240 | | negative regulation of extrinsic apoptotic signaling pathway in absence of ligand | 3/97 | 33/18723 | 0.000657 | 0.003749 | 0.002192 | BCL2/AKT1/TNF | 3 |
| BP | GO:0051701 | | biological process involved in interaction with host | 6/97 | 203/18723 | 0.000657 | 0.003749 | 0.002192 | ANO6/EGFR/CXCL8/IRGM/ACE2/CLEC5A | 6 |
| BP | GO:0002705 | | positive regulation of leukocyte mediated immunity | 5/97 | 134/18723 | 0.000663 | 0.003774 | 0.002206 | NLRP3/FADD/TREM2/IL18/TNF | 5 |
| BP | GO:0044262 | | cellular carbohydrate metabolic process | 7/97 | 283/18723 | 0.000675 | 0.003833 | 0.002241 | SIRT1/GPER1/PTEN/SESN2/AKT1/TP53/MST1 | 7 |
| BP | GO:0010827 | | regulation of glucose transmembrane transport | 4/97 | 77/18723 | 0.000686 | 0.003891 | 0.002275 | NFE2L2/AKT1/TNF/FGF21 | 4 |
| BP | GO:0022409 | | positive regulation of cell-cell adhesion | 7/97 | 284/18723 | 0.000689 | 0.003901 | 0.00228 | NLRP3/FADD/AKT1/PYCARD/IL18/TNF/IL36B | 7 |
| BP | GO:0043401 | | steroid hormone mediated signaling pathway | 5/97 | 136/18723 | 0.000709 | 0.003986 | 0.00233 | SIRT1/GPER1/HDAC6/PARP1/TP63 | 5 |
| BP | GO:0045727 | | positive regulation of translation | 5/97 | 136/18723 | 0.000709 | 0.003986 | 0.00233 | DDX3X/DHX9/METTL3/ELAVL1/TNF | 5 |
| BP | GO:0002495 | | antigen processing and presentation of peptide antigen via MHC class II | 3/97 | 34/18723 | 0.000718 | 0.003986 | 0.00233 | PYCARD/TREM2/CTSV | 3 |
| BP | GO:0010661 | | positive regulation of muscle cell apoptotic process | 3/97 | 34/18723 | 0.000718 | 0.003986 | 0.00233 | PTEN/TP53/BNIP3 | 3 |
| BP | GO:0032770 | | positive regulation of monooxygenase activity | 3/97 | 34/18723 | 0.000718 | 0.003986 | 0.00233 | AKT1/APOE/TNF | 3 |
| BP | GO:0034383 | | low-density lipoprotein particle clearance | 3/97 | 34/18723 | 0.000718 | 0.003986 | 0.00233 | TREM2/ANXA2/FGF21 | 3 |
| BP | GO:0035590 | | purinergic nucleotide receptor signaling pathway | 3/97 | 34/18723 | 0.000718 | 0.003986 | 0.00233 | CTSG/ANO6/P2RX7 | 3 |
| BP | GO:0043516 | | regulation of DNA damage response, signal transduction by p53 class mediator | 3/97 | 34/18723 | 0.000718 | 0.003986 | 0.00233 | SIRT1/MDM2/TP53 | 3 |
| BP | GO:0048536 | | spleen development | 3/97 | 34/18723 | 0.000718 | 0.003986 | 0.00233 | BCL2/FADD/RIPK3 | 3 |
| BP | GO:0070232 | | regulation of T cell apoptotic process | 3/97 | 34/18723 | 0.000718 | 0.003986 | 0.00233 | FADD/TP53/RIPK3 | 3 |
| BP | GO:0061045 | | negative regulation of wound healing | 4/97 | 78/18723 | 0.00072 | 0.003986 | 0.00233 | PTEN/APOE/ANXA2/TNF | 4 |
| BP | GO:0090559 | | regulation of membrane permeability | 4/97 | 78/18723 | 0.00072 | 0.003986 | 0.00233 | BCL2/TP53/BNIP3/GZMB | 4 |
| BP | GO:0002764 | | immune response-regulating signaling pathway | 9/97 | 468/18723 | 0.000721 | 0.003986 | 0.00233 | BCL2/LY96/DDX3X/BIRC2/IRF3/CD14/TLR2/IRGM/TREM2 | 9 |
| BP | GO:0006417 | | regulation of translation | 9/97 | 468/18723 | 0.000721 | 0.003986 | 0.00233 | DDX3X/FOXO3/DHX9/METTL3/ELAVL1/SESN2/AKT1/GZMB/TNF | 9 |
| BP | GO:0045471 | | response to ethanol | 5/97 | 137/18723 | 0.000733 | 0.004046 | 0.002366 | PTEN/NLRP3/BIRC2/CASP8/CD14 | 5 |
| BP | GO:0001893 | | maternal placenta development | 3/97 | 35/18723 | 0.000782 | 0.00429 | 0.002508 | PRDM1/AKT1/CTSV | 3 |
| BP | GO:0043243 | | positive regulation of protein-containing complex disassembly | 3/97 | 35/18723 | 0.000782 | 0.00429 | 0.002508 | IRGM/BNIP3/TNF | 3 |
| BP | GO:0045598 | | regulation of fat cell differentiation | 5/97 | 139/18723 | 0.000782 | 0.00429 | 0.002508 | SIRT1/GPER1/HDAC6/AKT1/TNF | 5 |
| BP | GO:0007623 | | circadian rhythm | 6/97 | 210/18723 | 0.000785 | 0.00429 | 0.002508 | JUN/SIRT1/PTEN/EGFR/METTL3/TP53 | 6 |
| BP | GO:0045637 | | regulation of myeloid cell differentiation | 6/97 | 210/18723 | 0.000785 | 0.00429 | 0.002508 | JUN/FOXO3/FADD/CASP8/TREM2/TNF | 6 |
| BP | GO:0050866 | | negative regulation of cell activation | 6/97 | 210/18723 | 0.000785 | 0.00429 | 0.002508 | GPER1/GLMN/CASP3/APOE/TREM2/IL13RA2 | 6 |
| BP | GO:0030100 | | regulation of endocytosis | 6/97 | 211/18723 | 0.000804 | 0.004383 | 0.002562 | ANO6/EEF2K/CD14/APOE/TREM2/ANXA2 | 6 |
| BP | GO:0050807 | | regulation of synapse organization | 6/97 | 211/18723 | 0.000804 | 0.004383 | 0.002562 | PTEN/EEF2K/TLR2/APOE/TREM2/TNF | 6 |
| BP | GO:0007292 | | female gamete generation | 5/97 | 140/18723 | 0.000808 | 0.004396 | 0.00257 | BCL2/SIRT1/FOXO3/METTL3/PANX1 | 5 |
| BP | GO:0010632 | | regulation of epithelial cell migration | 7/97 | 292/18723 | 0.000811 | 0.004403 | 0.002574 | SIRT1/PTEN/NFE2L2/HDAC6/AKT1/APOE/TNF | 7 |
| BP | GO:0006469 | | negative regulation of protein kinase activity | 6/97 | 212/18723 | 0.000824 | 0.00447 | 0.002613 | SIRT1/PTEN/AKT1/CASP3/APOE/PYCARD | 6 |
| BP | GO:0001942 | | hair follicle development | 4/97 | 81/18723 | 0.00083 | 0.004471 | 0.002614 | BCL2/EGFR/TP63/TNF | 4 |
| BP | GO:0001960 | | negative regulation of cytokine-mediated signaling pathway | 4/97 | 81/18723 | 0.00083 | 0.004471 | 0.002614 | CARD8/METTL3/TREM2/IL1RN | 4 |
| BP | GO:0051149 | | positive regulation of muscle cell differentiation | 4/97 | 81/18723 | 0.00083 | 0.004471 | 0.002614 | BCL2/SIRT1/GPER1/MDM2 | 4 |
| BP | GO:0051817 | | modulation of process of other organism involved in symbiotic interaction | 4/97 | 81/18723 | 0.00083 | 0.004471 | 0.002614 | ANO6/JUN/IRGM/APOE | 4 |
| BP | GO:0035296 | | regulation of tube diameter | 5/97 | 141/18723 | 0.000834 | 0.00448 | 0.002619 | GPER1/EGFR/AKT1/APOE/ACE2 | 5 |
| BP | GO:0097746 | | blood vessel diameter maintenance | 5/97 | 141/18723 | 0.000834 | 0.00448 | 0.002619 | GPER1/EGFR/AKT1/APOE/ACE2 | 5 |
| CC | GO:0030667 | | secretory granule membrane | 8/97 | 311/19550 | 0.000159 | 0.004507 | 0.003658 | ANO6/PECAM1/STING1/ORMDL3/CD14/TLR2/BST2/CLEC5A | 8 |
| BP | GO:0002504 | | antigen processing and presentation of peptide or polysaccharide antigen via MHC class II | 3/97 | 36/18723 | 0.00085 | 0.004543 | 0.002656 | PYCARD/TREM2/CTSV | 3 |
| BP | GO:0051385 | | response to mineralocorticoid | 3/97 | 36/18723 | 0.00085 | 0.004543 | 0.002656 | GPER1/FOXO3/PARP1 | 3 |
| BP | GO:0090322 | | regulation of superoxide metabolic process | 3/97 | 36/18723 | 0.00085 | 0.004543 | 0.002656 | EGFR/NFE2L2/AKT1 | 3 |
| BP | GO:0035150 | | regulation of tube size | 5/97 | 142/18723 | 0.000861 | 0.004595 | 0.002686 | GPER1/EGFR/AKT1/APOE/ACE2 | 5 |
| BP | GO:0050870 | | positive regulation of T cell activation | 6/97 | 216/18723 | 0.000908 | 0.004829 | 0.002823 | NLRP3/FADD/AKT1/PYCARD/IL18/IL36B | 6 |
| BP | GO:0048678 | | response to axon injury | 4/97 | 83/18723 | 0.00091 | 0.004829 | 0.002823 | BCL2/PTEN/TREM2/BNIP3 | 4 |
| BP | GO:0051899 | | membrane depolarization | 4/97 | 83/18723 | 0.00091 | 0.004829 | 0.002823 | BCL2/P2RX7/JUN/PARP1 | 4 |
| BP | GO:0001890 | | placenta development | 5/97 | 144/18723 | 0.000917 | 0.004862 | 0.002842 | PRDM1/BIRC2/AKT1/CASP8/CTSV | 5 |
| BP | GO:0051961 | | negative regulation of nervous system development | 5/97 | 145/18723 | 0.000946 | 0.004994 | 0.00292 | PTEN/TP53/TLR2/TREM2/TNF | 5 |
| BP | GO:1901987 | | regulation of cell cycle phase transition | 8/97 | 390/18723 | 0.000949 | 0.004994 | 0.00292 | BCL2/PTEN/EGFR/DDX3X/MRE11/MDM2/AKT1/TP53 | 8 |
| BP | GO:0022404 | | molting cycle process | 4/97 | 84/18723 | 0.000951 | 0.004994 | 0.00292 | BCL2/EGFR/TP63/TNF | 4 |
| BP | GO:0022405 | | hair cycle process | 4/97 | 84/18723 | 0.000951 | 0.004994 | 0.00292 | BCL2/EGFR/TP63/TNF | 4 |
| BP | GO:0032370 | | positive regulation of lipid transport | 4/97 | 84/18723 | 0.000951 | 0.004994 | 0.00292 | SIRT1/APOE/TREM2/ANXA2 | 4 |
| BP | GO:2000134 | | negative regulation of G1/S transition of mitotic cell cycle | 4/97 | 84/18723 | 0.000951 | 0.004994 | 0.00292 | BCL2/PTEN/MDM2/TP53 | 4 |
| BP | GO:0050803 | | regulation of synapse structure or activity | 6/97 | 218/18723 | 0.000953 | 0.004994 | 0.00292 | PTEN/EEF2K/TLR2/APOE/TREM2/TNF | 6 |
| BP | GO:0040013 | | negative regulation of locomotion | 8/97 | 391/18723 | 0.000965 | 0.005052 | 0.002953 | BCL2/PTEN/NFE2L2/FOXO3/AKT1/APOE/BST2/TNF | 8 |
| BP | GO:0045580 | | regulation of T cell differentiation | 5/97 | 146/18723 | 0.000976 | 0.005098 | 0.002981 | PRDM1/NLRP3/METTL3/IL18/IL36B | 5 |
| BP | GO:0002709 | | regulation of T cell mediated immunity | 4/97 | 85/18723 | 0.000994 | 0.005171 | 0.003023 | NLRP3/FADD/RIPK3/IL18 | 4 |
| BP | GO:0097006 | | regulation of plasma lipoprotein particle levels | 4/97 | 85/18723 | 0.000994 | 0.005171 | 0.003023 | APOE/TREM2/ANXA2/FGF21 | 4 |
| BP | GO:0098773 | | skin epidermis development | 4/97 | 85/18723 | 0.000994 | 0.005171 | 0.003023 | BCL2/EGFR/TP63/TNF | 4 |
| BP | GO:0097242 | | amyloid-beta clearance | 3/97 | 38/18723 | 0.000997 | 0.005177 | 0.003027 | APOE/TREM2/TNF | 3 |
| BP | GO:0051222 | | positive regulation of protein transport | 7/97 | 303/18723 | 0.001006 | 0.005214 | 0.003048 | GPER1/EGFR/MDM2/TLR2/TREM2/GZMB/TNF | 7 |
| BP | GO:0034765 | | regulation of ion transmembrane transport | 9/97 | 491/18723 | 0.001011 | 0.005231 | 0.003058 | ANO6/P2RX7/GPER1/PTEN/GSTO1/AKT1/CAPN1/TREM2/ACE2 | 9 |
| BP | GO:0034103 | | regulation of tissue remodeling | 4/97 | 86/18723 | 0.001039 | 0.00536 | 0.003134 | P2RX7/EGFR/TP53/IL18 | 4 |
| BP | GO:0060761 | | negative regulation of response to cytokine stimulus | 4/97 | 86/18723 | 0.001039 | 0.00536 | 0.003134 | CARD8/METTL3/TREM2/IL1RN | 4 |
| MF | GO:0002039 | | p53 binding | 4/97 | 66/18368 | 0.00041 | 0.005434 | 0.004231 | SIRT1/MDM2/TP53/TP63 | 4 |
| BP | GO:0002701 | | negative regulation of production of molecular mediator of immune response | 3/97 | 39/18723 | 0.001076 | 0.005536 | 0.003236 | BST2/IL13RA2/TNF | 3 |
| BP | GO:0046326 | | positive regulation of glucose import | 3/97 | 39/18723 | 0.001076 | 0.005536 | 0.003236 | NFE2L2/AKT1/FGF21 | 3 |
| BP | GO:1900182 | | positive regulation of protein localization to nucleus | 4/97 | 87/18723 | 0.001084 | 0.005562 | 0.003252 | SESN2/AKT1/ORMDL3/PARP1 | 4 |
| BP | GO:1903578 | | regulation of ATP metabolic process | 4/97 | 87/18723 | 0.001084 | 0.005562 | 0.003252 | P2RX7/TP53/PARP1/TREM2 | 4 |
| BP | GO:0070665 | | positive regulation of leukocyte proliferation | 5/97 | 150/18723 | 0.001101 | 0.005637 | 0.003296 | BCL2/FADD/PYCARD/BST2/IL18 | 5 |
| BP | GO:0043535 | | regulation of blood vessel endothelial cell migration | 5/97 | 151/18723 | 0.001134 | 0.005789 | 0.003384 | SIRT1/NFE2L2/AKT1/APOE/TNF | 5 |
| BP | GO:1904064 | | positive regulation of cation transmembrane transport | 5/97 | 151/18723 | 0.001134 | 0.005789 | 0.003384 | ANO6/GPER1/GSTO1/TREM2/ACE2 | 5 |
| BP | GO:0045740 | | positive regulation of DNA replication | 3/97 | 40/18723 | 0.001159 | 0.005858 | 0.003425 | JUN/EGFR/DHX9 | 3 |
| BP | GO:0071276 | | cellular response to cadmium ion | 3/97 | 40/18723 | 0.001159 | 0.005858 | 0.003425 | JUN/EGFR/AKT1 | 3 |
| BP | GO:0150077 | | regulation of neuroinflammatory response | 3/97 | 40/18723 | 0.001159 | 0.005858 | 0.003425 | TREM2/IL18/TNF | 3 |
| BP | GO:0002002 | | regulation of angiotensin levels in blood | 2/97 | 10/18723 | 0.001164 | 0.005858 | 0.003425 | CTSG/ACE2 | 2 |
| BP | GO:0002003 | | angiotensin maturation | 2/97 | 10/18723 | 0.001164 | 0.005858 | 0.003425 | CTSG/ACE2 | 2 |
| BP | GO:0032070 | | regulation of deoxyribonuclease activity | 2/97 | 10/18723 | 0.001164 | 0.005858 | 0.003425 | SIRT1/AKT1 | 2 |
| BP | GO:0042368 | | vitamin D biosynthetic process | 2/97 | 10/18723 | 0.001164 | 0.005858 | 0.003425 | NFKB1/TNF | 2 |
| BP | GO:0045792 | | negative regulation of cell size | 2/97 | 10/18723 | 0.001164 | 0.005858 | 0.003425 | PTEN/AKT1 | 2 |
| BP | GO:2000659 | | regulation of interleukin-1-mediated signaling pathway | 2/97 | 10/18723 | 0.001164 | 0.005858 | 0.003425 | ZBP1/IL1RN | 2 |
| BP | GO:0008643 | | carbohydrate transport | 5/97 | 152/18723 | 0.001168 | 0.005871 | 0.003432 | NFE2L2/SESN2/AKT1/TNF/FGF21 | 5 |
| BP | GO:0070588 | | calcium ion transmembrane transport | 7/97 | 312/18723 | 0.001191 | 0.00598 | 0.003496 | ANO6/P2RX7/GPER1/PANX1/GSTO1/AKT1/ANXA2 | 7 |
| MF | GO:0033612 | | receptor serine/threonine kinase binding | 3/97 | 29/18368 | 0.000472 | 0.006065 | 0.004722 | FADD/MDM2/PYCARD | 3 |
| BP | GO:0045444 | | fat cell differentiation | 6/97 | 229/18723 | 0.001227 | 0.006145 | 0.003592 | SIRT1/GPER1/HDAC6/AKT1/BNIP3/TNF | 6 |
| BP | GO:0010959 | | regulation of metal ion transport | 8/97 | 406/18723 | 0.001228 | 0.006145 | 0.003592 | BCL2/ANO6/P2RX7/GPER1/PTEN/GSTO1/AKT1/TREM2 | 8 |
| BP | GO:0048477 | | oogenesis | 4/97 | 90/18723 | 0.00123 | 0.006148 | 0.003594 | BCL2/FOXO3/METTL3/PANX1 | 4 |
| BP | GO:0032733 | | positive regulation of interleukin-10 production | 3/97 | 41/18723 | 0.001246 | 0.006216 | 0.003634 | TLR2/PYCARD/TREM2 | 3 |
| BP | GO:0002696 | | positive regulation of leukocyte activation | 8/97 | 409/18723 | 0.001287 | 0.00641 | 0.003747 | BCL2/NLRP3/FADD/AKT1/PYCARD/TREM2/IL18/IL36B | 8 |
| CC | GO:0035580 | | specific granule lumen | 4/97 | 62/19550 | 0.000255 | 0.006427 | 0.005215 | NFKB1/GSDMD/CHI3L1/CAMP | 4 |
| BP | GO:0071312 | | cellular response to alkaloid | 3/97 | 42/18723 | 0.001337 | 0.006629 | 0.003875 | MDM2/CASP3/CASP6 | 3 |
| BP | GO:1904037 | | positive regulation of epithelial cell apoptotic process | 3/97 | 42/18723 | 0.001337 | 0.006629 | 0.003875 | ANO6/GPER1/FOXO3 | 3 |
| BP | GO:2000404 | | regulation of T cell migration | 3/97 | 42/18723 | 0.001337 | 0.006629 | 0.003875 | FADD/RIPK3/PYCARD | 3 |
| BP | GO:1904951 | | positive regulation of establishment of protein localization | 7/97 | 319/18723 | 0.001353 | 0.006702 | 0.003918 | GPER1/EGFR/MDM2/TLR2/TREM2/GZMB/TNF | 7 |
| BP | GO:0032755 | | positive regulation of interleukin-6 production | 4/97 | 93/18723 | 0.001389 | 0.006839 | 0.003998 | DHX9/TLR2/PYCARD/TNF | 4 |
| BP | GO:0097306 | | cellular response to alcohol | 4/97 | 93/18723 | 0.001389 | 0.006839 | 0.003998 | PTEN/FOXO3/MDM2/AKT1 | 4 |
| BP | GO:1901992 | | positive regulation of mitotic cell cycle phase transition | 4/97 | 93/18723 | 0.001389 | 0.006839 | 0.003998 | EGFR/DDX3X/MDM2/AKT1 | 4 |
| BP | GO:1902807 | | negative regulation of cell cycle G1/S phase transition | 4/97 | 93/18723 | 0.001389 | 0.006839 | 0.003998 | BCL2/PTEN/MDM2/TP53 | 4 |
| BP | GO:0010986 | | positive regulation of lipoprotein particle clearance | 2/97 | 11/18723 | 0.001417 | 0.006875 | 0.004019 | TREM2/ANXA2 | 2 |
| BP | GO:0030656 | | regulation of vitamin metabolic process | 2/97 | 11/18723 | 0.001417 | 0.006875 | 0.004019 | NFKB1/TNF | 2 |
| BP | GO:0032493 | | response to bacterial lipoprotein | 2/97 | 11/18723 | 0.001417 | 0.006875 | 0.004019 | CD14/TLR2 | 2 |
| BP | GO:0036490 | | regulation of translation in response to endoplasmic reticulum stress | 2/97 | 11/18723 | 0.001417 | 0.006875 | 0.004019 | DDX3X/SESN2 | 2 |
| BP | GO:0043619 | | regulation of transcription from RNA polymerase II promoter in response to oxidative stress | 2/97 | 11/18723 | 0.001417 | 0.006875 | 0.004019 | NFE2L2/SESN2 | 2 |
| BP | GO:0044068 | | modulation by symbiont of host cellular process | 2/97 | 11/18723 | 0.001417 | 0.006875 | 0.004019 | ANO6/IRGM | 2 |
| BP | GO:0045628 | | regulation of T-helper 2 cell differentiation | 2/97 | 11/18723 | 0.001417 | 0.006875 | 0.004019 | NLRP3/IL18 | 2 |
| BP | GO:0071888 | | macrophage apoptotic process | 2/97 | 11/18723 | 0.001417 | 0.006875 | 0.004019 | SIRT1/IRF3 | 2 |
| BP | GO:1900227 | | positive regulation of NLRP3 inflammasome complex assembly | 2/97 | 11/18723 | 0.001417 | 0.006875 | 0.004019 | DDX3X/GBP5 | 2 |
| BP | GO:2000644 | | regulation of receptor catabolic process | 2/97 | 11/18723 | 0.001417 | 0.006875 | 0.004019 | APOE/ANXA2 | 2 |
| BP | GO:0000209 | | protein polyubiquitination | 6/97 | 236/18723 | 0.001431 | 0.006883 | 0.004024 | BCL2/DDX3X/BIRC2/HDAC6/MDM2/UBE2D2 | 6 |
| BP | GO:0014002 | | astrocyte development | 3/97 | 43/18723 | 0.001431 | 0.006883 | 0.004024 | EGFR/TREM2/TNF | 3 |
| BP | GO:0031952 | | regulation of protein autophosphorylation | 3/97 | 43/18723 | 0.001431 | 0.006883 | 0.004024 | DDX3X/MRE11/EEF2K | 3 |
| BP | GO:0034142 | | toll-like receptor 4 signaling pathway | 3/97 | 43/18723 | 0.001431 | 0.006883 | 0.004024 | LY96/CD14/TREM2 | 3 |
| BP | GO:0035794 | | positive regulation of mitochondrial membrane permeability | 3/97 | 43/18723 | 0.001431 | 0.006883 | 0.004024 | TP53/BNIP3/GZMB | 3 |
| BP | GO:2001239 | | regulation of extrinsic apoptotic signaling pathway in absence of ligand | 3/97 | 43/18723 | 0.001431 | 0.006883 | 0.004024 | BCL2/AKT1/TNF | 3 |
| BP | GO:0000079 | | regulation of cyclin-dependent protein serine/threonine kinase activity | 4/97 | 94/18723 | 0.001445 | 0.00693 | 0.004051 | PTEN/EGFR/AKT1/CASP3 | 4 |
| BP | GO:1903035 | | negative regulation of response to wounding | 4/97 | 94/18723 | 0.001445 | 0.00693 | 0.004051 | PTEN/APOE/ANXA2/TNF | 4 |
| BP | GO:0019318 | | hexose metabolic process | 6/97 | 237/18723 | 0.001462 | 0.006991 | 0.004087 | SIRT1/SESN2/AKT1/TP53/MST1/TNF | 6 |
| BP | GO:0033673 | | negative regulation of kinase activity | 6/97 | 237/18723 | 0.001462 | 0.006991 | 0.004087 | SIRT1/PTEN/AKT1/CASP3/APOE/PYCARD | 6 |
| MF | GO:0005164 | | tumor necrosis factor receptor binding | 3/97 | 31/18368 | 0.000576 | 0.007062 | 0.005499 | FADD/CASP8/TNF | 3 |
| MF | GO:0035091 | | phosphatidylinositol binding | 7/97 | 271/18368 | 0.000584 | 0.007062 | 0.005499 | GSDME/AKT1/GSDMD/GSDMA/ANXA2/GSDMB/GSDMC | 7 |
| BP | GO:0008585 | | female gonad development | 4/97 | 95/18723 | 0.001503 | 0.007175 | 0.004195 | BCL2/SIRT1/FOXO3/CASP3 | 4 |
| MF | GO:0051721 | | protein phosphatase 2A binding | 3/97 | 32/18368 | 0.000634 | 0.007234 | 0.005633 | BCL2/AKT1/TP53 | 3 |
| MF | GO:0003725 | | double-stranded RNA binding | 4/97 | 74/18368 | 0.000634 | 0.007234 | 0.005633 | NLRP1/DHX9/ELAVL1/ZBP1 | 4 |
| BP | GO:0050867 | | positive regulation of cell activation | 8/97 | 420/18723 | 0.001522 | 0.007254 | 0.004241 | BCL2/NLRP3/FADD/AKT1/PYCARD/TREM2/IL18/IL36B | 8 |
| BP | GO:0051403 | | stress-activated MAPK cascade | 6/97 | 239/18723 | 0.001526 | 0.007265 | 0.004247 | EGFR/NFKB1/AKT1/PYCARD/TREM2/TNF | 6 |
| BP | GO:0150076 | | neuroinflammatory response | 3/97 | 44/18723 | 0.00153 | 0.007275 | 0.004253 | TREM2/IL18/TNF | 3 |
| BP | GO:0015914 | | phospholipid transport | 4/97 | 96/18723 | 0.001562 | 0.007404 | 0.004329 | ANO6/P2RX7/CPTP/APOE | 4 |
| BP | GO:1901655 | | cellular response to ketone | 4/97 | 96/18723 | 0.001562 | 0.007404 | 0.004329 | SIRT1/EGFR/FOXO3/AKT1 | 4 |
| BP | GO:0006816 | | calcium ion transport | 8/97 | 422/18723 | 0.001568 | 0.00741 | 0.004332 | BCL2/ANO6/P2RX7/GPER1/PANX1/GSTO1/AKT1/ANXA2 | 8 |
| BP | GO:0042060 | | wound healing | 8/97 | 422/18723 | 0.001568 | 0.00741 | 0.004332 | ANO6/PTEN/EGFR/NFE2L2/CASP3/APOE/ANXA2/TNF | 8 |
| BP | GO:0044772 | | mitotic cell cycle phase transition | 8/97 | 424/18723 | 0.001615 | 0.007623 | 0.004456 | BCL2/PTEN/EGFR/DDX3X/MRE11/MDM2/AKT1/TP53 | 8 |
| BP | GO:2001237 | | negative regulation of extrinsic apoptotic signaling pathway | 4/97 | 97/18723 | 0.001623 | 0.007627 | 0.004459 | BCL2/DDX3X/AKT1/TNF | 4 |
| BP | GO:0022602 | | ovulation cycle process | 3/97 | 45/18723 | 0.001634 | 0.007627 | 0.004459 | SIRT1/FOXO3/CASP3 | 3 |
| BP | GO:0045933 | | positive regulation of muscle contraction | 3/97 | 45/18723 | 0.001634 | 0.007627 | 0.004459 | GPER1/GSTO1/ACE2 | 3 |
| BP | GO:0048538 | | thymus development | 3/97 | 45/18723 | 0.001634 | 0.007627 | 0.004459 | BCL2/FADD/RIPK3 | 3 |
| BP | GO:1902108 | | regulation of mitochondrial membrane permeability involved in apoptotic process | 3/97 | 45/18723 | 0.001634 | 0.007627 | 0.004459 | TP53/BNIP3/GZMB | 3 |
| BP | GO:1904646 | | cellular response to amyloid-beta | 3/97 | 45/18723 | 0.001634 | 0.007627 | 0.004459 | FOXO3/PARP1/TREM2 | 3 |
| BP | GO:0050806 | | positive regulation of synaptic transmission | 5/97 | 164/18723 | 0.001635 | 0.007627 | 0.004459 | GPER1/PTEN/EGFR/APOE/TNF | 5 |
| BP | GO:2000058 | | regulation of ubiquitin-dependent protein catabolic process | 5/97 | 164/18723 | 0.001635 | 0.007627 | 0.004459 | PTEN/NFE2L2/GLMN/MDM2/AKT1 | 5 |
| BP | GO:0032635 | | interleukin-6 production | 5/97 | 165/18723 | 0.001679 | 0.00781 | 0.004566 | DHX9/TLR2/PYCARD/TREM2/TNF | 5 |
| BP | GO:0032675 | | regulation of interleukin-6 production | 5/97 | 165/18723 | 0.001679 | 0.00781 | 0.004566 | DHX9/TLR2/PYCARD/TREM2/TNF | 5 |
| BP | GO:1904029 | | regulation of cyclin-dependent protein kinase activity | 4/97 | 98/18723 | 0.001685 | 0.00781 | 0.004566 | PTEN/EGFR/AKT1/CASP3 | 4 |
| BP | GO:0000012 | | single strand break repair | 2/97 | 12/18723 | 0.001695 | 0.00781 | 0.004566 | SIRT1/PARP1 | 2 |
| BP | GO:0051095 | | regulation of helicase activity | 2/97 | 12/18723 | 0.001695 | 0.00781 | 0.004566 | SIRT1/TP53 | 2 |
| BP | GO:0072683 | | T cell extravasation | 2/97 | 12/18723 | 0.001695 | 0.00781 | 0.004566 | FADD/RIPK3 | 2 |
| BP | GO:0140052 | | cellular response to oxidised low-density lipoprotein particle stimulus | 2/97 | 12/18723 | 0.001695 | 0.00781 | 0.004566 | AKT1/TREM2 | 2 |
| BP | GO:1901298 | | regulation of hydrogen peroxide-mediated programmed cell death | 2/97 | 12/18723 | 0.001695 | 0.00781 | 0.004566 | FOXO3/HDAC6 | 2 |
| BP | GO:1903800 | | positive regulation of production of miRNAs involved in gene silencing by miRNA | 2/97 | 12/18723 | 0.001695 | 0.00781 | 0.004566 | EGFR/TP53 | 2 |
| BP | GO:0010828 | | positive regulation of glucose transmembrane transport | 3/97 | 46/18723 | 0.001741 | 0.008012 | 0.004684 | NFE2L2/AKT1/FGF21 | 3 |
| BP | GO:0060996 | | dendritic spine development | 4/97 | 99/18723 | 0.001749 | 0.008039 | 0.0047 | PTEN/EEF2K/HDAC6/APOE | 4 |
| BP | GO:0007254 | | JNK cascade | 5/97 | 167/18723 | 0.00177 | 0.008122 | 0.004748 | EGFR/NFKB1/AKT1/PYCARD/TNF | 5 |
| BP | GO:0031098 | | stress-activated protein kinase signaling cascade | 6/97 | 247/18723 | 0.001803 | 0.008261 | 0.00483 | EGFR/NFKB1/AKT1/PYCARD/TREM2/TNF | 6 |
| BP | GO:0019218 | | regulation of steroid metabolic process | 4/97 | 100/18723 | 0.001815 | 0.008291 | 0.004847 | SIRT1/NFKB1/APOE/TNF | 4 |
| BP | GO:0046545 | | development of primary female sexual characteristics | 4/97 | 100/18723 | 0.001815 | 0.008291 | 0.004847 | BCL2/SIRT1/FOXO3/CASP3 | 4 |
| BP | GO:0099173 | | postsynapse organization | 5/97 | 168/18723 | 0.001817 | 0.008291 | 0.004847 | PTEN/EEF2K/HDAC6/APOE/TREM2 | 5 |
| BP | GO:0001774 | | microglial cell activation | 3/97 | 47/18723 | 0.001853 | 0.008421 | 0.004923 | TLR2/TREM2/TNF | 3 |
| BP | GO:0010803 | | regulation of tumor necrosis factor-mediated signaling pathway | 3/97 | 47/18723 | 0.001853 | 0.008421 | 0.004923 | CARD8/CASP8/PYCARD | 3 |
| BP | GO:0042311 | | vasodilation | 3/97 | 47/18723 | 0.001853 | 0.008421 | 0.004923 | GPER1/EGFR/APOE | 3 |
| BP | GO:0098869 | | cellular oxidant detoxification | 4/97 | 101/18723 | 0.001883 | 0.008544 | 0.004995 | NFE2L2/GSTO1/SESN2/APOE | 4 |
| BP | GO:0050673 | | epithelial cell proliferation | 8/97 | 437/18723 | 0.001951 | 0.008834 | 0.005164 | SIRT1/PTEN/EGFR/AKT1/SERPINB1/APOE/TP63/TNF | 8 |
| BP | GO:0010522 | | regulation of calcium ion transport into cytosol | 4/97 | 102/18723 | 0.001952 | 0.008834 | 0.005164 | BCL2/P2RX7/GPER1/GSTO1 | 4 |
| BP | GO:0006111 | | regulation of gluconeogenesis | 3/97 | 48/18723 | 0.001969 | 0.008866 | 0.005183 | SIRT1/SESN2/MST1 | 3 |
| BP | GO:1902003 | | regulation of amyloid-beta formation | 3/97 | 48/18723 | 0.001969 | 0.008866 | 0.005183 | CASP3/APOE/TNF | 3 |
| BP | GO:1905710 | | positive regulation of membrane permeability | 3/97 | 48/18723 | 0.001969 | 0.008866 | 0.005183 | TP53/BNIP3/GZMB | 3 |
| BP | GO:0090257 | | regulation of muscle system process | 6/97 | 252/18723 | 0.001994 | 0.008866 | 0.005183 | GPER1/FOXO3/GSTO1/ORMDL3/PARP1/ACE2 | 6 |
| BP | GO:0031053 | | primary miRNA processing | 2/97 | 13/18723 | 0.001996 | 0.008866 | 0.005183 | DDX3X/METTL3 | 2 |
| BP | GO:0032494 | | response to peptidoglycan | 2/97 | 13/18723 | 0.001996 | 0.008866 | 0.005183 | TREM2/CAMP | 2 |
| BP | GO:0036005 | | response to macrophage colony-stimulating factor | 2/97 | 13/18723 | 0.001996 | 0.008866 | 0.005183 | TLR2/TREM2 | 2 |
| BP | GO:0036006 | | cellular response to macrophage colony-stimulating factor stimulus | 2/97 | 13/18723 | 0.001996 | 0.008866 | 0.005183 | TLR2/TREM2 | 2 |
| BP | GO:0042362 | | fat-soluble vitamin biosynthetic process | 2/97 | 13/18723 | 0.001996 | 0.008866 | 0.005183 | NFKB1/TNF | 2 |
| BP | GO:0060100 | | positive regulation of phagocytosis, engulfment | 2/97 | 13/18723 | 0.001996 | 0.008866 | 0.005183 | ANO6/TREM2 | 2 |
| BP | GO:0061418 | | regulation of transcription from RNA polymerase II promoter in response to hypoxia | 2/97 | 13/18723 | 0.001996 | 0.008866 | 0.005183 | NFE2L2/TP53 | 2 |
| BP | GO:1900044 | | regulation of protein K63-linked ubiquitination | 2/97 | 13/18723 | 0.001996 | 0.008866 | 0.005183 | DDX3X/BIRC2 | 2 |
| BP | GO:1905155 | | positive regulation of membrane invagination | 2/97 | 13/18723 | 0.001996 | 0.008866 | 0.005183 | ANO6/TREM2 | 2 |
| BP | GO:1905907 | | negative regulation of amyloid fibril formation | 2/97 | 13/18723 | 0.001996 | 0.008866 | 0.005183 | APOE/TREM2 | 2 |
| BP | GO:0001678 | | cellular glucose homeostasis | 5/97 | 172/18723 | 0.002014 | 0.008931 | 0.005221 | SIRT1/GPER1/FOXO3/TREM2/FGF21 | 5 |
| BP | GO:0014013 | | regulation of gliogenesis | 4/97 | 103/18723 | 0.002023 | 0.008959 | 0.005237 | EGFR/TLR2/TREM2/TNF | 4 |
| CC | GO:0030666 | | endocytic vesicle membrane | 6/97 | 193/19550 | 0.000402 | 0.009123 | 0.007403 | EGFR/MDM2/TLR2/IRGM/APOE/ACE2 | 6 |
| BP | GO:0010469 | | regulation of signaling receptor activity | 5/97 | 173/18723 | 0.002065 | 0.009123 | 0.005333 | PTEN/HDAC6/CAPN1/ACE2/TNF | 5 |
| BP | GO:0051099 | | positive regulation of binding | 5/97 | 173/18723 | 0.002065 | 0.009123 | 0.005333 | STING1/DHX9/APOE/PARP1/ANXA2 | 5 |
| BP | GO:0010823 | | negative regulation of mitochondrion organization | 3/97 | 49/18723 | 0.00209 | 0.009182 | 0.005368 | AKT1/TP53/BNIP3 | 3 |
| BP | GO:0043330 | | response to exogenous dsRNA | 3/97 | 49/18723 | 0.00209 | 0.009182 | 0.005368 | STING1/DHX9/IRF3 | 3 |
| BP | GO:0070741 | | response to interleukin-6 | 3/97 | 49/18723 | 0.00209 | 0.009182 | 0.005368 | NFKB1/CHI3L1/CAMP | 3 |
| BP | GO:1904707 | | positive regulation of vascular associated smooth muscle cell proliferation | 3/97 | 49/18723 | 0.00209 | 0.009182 | 0.005368 | JUN/MDM2/TNF | 3 |
| BP | GO:0045619 | | regulation of lymphocyte differentiation | 5/97 | 174/18723 | 0.002118 | 0.009293 | 0.005433 | PRDM1/NLRP3/METTL3/IL18/IL36B | 5 |
| BP | GO:0062207 | | regulation of pattern recognition receptor signaling pathway | 4/97 | 105/18723 | 0.00217 | 0.009496 | 0.005551 | DDX3X/BIRC2/TLR2/TREM2 | 4 |
| BP | GO:2001022 | | positive regulation of response to DNA damage stimulus | 4/97 | 105/18723 | 0.00217 | 0.009496 | 0.005551 | SIRT1/EGFR/DHX9/PARP1 | 4 |
| BP | GO:1902930 | | regulation of alcohol biosynthetic process | 3/97 | 50/18723 | 0.002215 | 0.00968 | 0.005659 | GPER1/NFKB1/APOE | 3 |
| BP | GO:0043534 | | blood vessel endothelial cell migration | 5/97 | 176/18723 | 0.002226 | 0.009715 | 0.00568 | SIRT1/NFE2L2/AKT1/APOE/TNF | 5 |
| BP | GO:0071466 | | cellular response to xenobiotic stimulus | 5/97 | 177/18723 | 0.002281 | 0.009944 | 0.005813 | EGFR/NFE2L2/GSTO1/AIM2/FGF21 | 5 |
| BP | GO:0010421 | | hydrogen peroxide-mediated programmed cell death | 2/97 | 14/18723 | 0.002321 | 0.009987 | 0.005838 | FOXO3/HDAC6 | 2 |
| BP | GO:0045989 | | positive regulation of striated muscle contraction | 2/97 | 14/18723 | 0.002321 | 0.009987 | 0.005838 | GSTO1/ACE2 | 2 |
| BP | GO:0070141 | | response to UV-A | 2/97 | 14/18723 | 0.002321 | 0.009987 | 0.005838 | EGFR/AKT1 | 2 |
| BP | GO:0071236 | | cellular response to antibiotic | 2/97 | 14/18723 | 0.002321 | 0.009987 | 0.005838 | MDM2/TP53 | 2 |
| BP | GO:1902036 | | regulation of hematopoietic stem cell differentiation | 2/97 | 14/18723 | 0.002321 | 0.009987 | 0.005838 | NFE2L2/METTL3 | 2 |
| BP | GO:1902166 | | negative regulation of intrinsic apoptotic signaling pathway in response to DNA damage by p53 class mediator | 2/97 | 14/18723 | 0.002321 | 0.009987 | 0.005838 | BCL2/SIRT1 | 2 |
| BP | GO:1902916 | | positive regulation of protein polyubiquitination | 2/97 | 14/18723 | 0.002321 | 0.009987 | 0.005838 | DDX3X/BIRC2 | 2 |
| BP | GO:0006275 | | regulation of DNA replication | 4/97 | 107/18723 | 0.002324 | 0.009987 | 0.005838 | JUN/EGFR/DHX9/TP53 | 4 |
| BP | GO:0042303 | | molting cycle | 4/97 | 107/18723 | 0.002324 | 0.009987 | 0.005838 | BCL2/EGFR/TP63/TNF | 4 |
| BP | GO:0042633 | | hair cycle | 4/97 | 107/18723 | 0.002324 | 0.009987 | 0.005838 | BCL2/EGFR/TP63/TNF | 4 |
| BP | GO:2000060 | | positive regulation of ubiquitin-dependent protein catabolic process | 4/97 | 107/18723 | 0.002324 | 0.009987 | 0.005838 | PTEN/NFE2L2/MDM2/AKT1 | 4 |
| BP | GO:0010874 | | regulation of cholesterol efflux | 3/97 | 51/18723 | 0.002344 | 0.010048 | 0.005874 | SIRT1/APOE/TREM2 | 3 |
| BP | GO:0014009 | | glial cell proliferation | 3/97 | 51/18723 | 0.002344 | 0.010048 | 0.005874 | EGFR/TREM2/TNF | 3 |
| BP | GO:1901991 | | negative regulation of mitotic cell cycle phase transition | 5/97 | 179/18723 | 0.002395 | 0.010252 | 0.005993 | BCL2/PTEN/MRE11/MDM2/TP53 | 5 |
| BP | GO:0043588 | | skin development | 6/97 | 263/18723 | 0.002469 | 0.010554 | 0.00617 | TXNIP/BCL2/EGFR/CASP3/TP63/TNF | 6 |
| BP | GO:0002456 | | T cell mediated immunity | 4/97 | 109/18723 | 0.002486 | 0.010613 | 0.006204 | NLRP3/FADD/RIPK3/IL18 | 4 |
| BP | GO:0008361 | | regulation of cell size | 5/97 | 181/18723 | 0.002513 | 0.010679 | 0.006243 | ANO6/P2RX7/PTEN/AKT1/APOE | 5 |
| BP | GO:0045766 | | positive regulation of angiogenesis | 5/97 | 181/18723 | 0.002513 | 0.010679 | 0.006243 | SIRT1/NFE2L2/CXCL8/CHI3L1/CAMP | 5 |
| BP | GO:1904018 | | positive regulation of vasculature development | 5/97 | 181/18723 | 0.002513 | 0.010679 | 0.006243 | SIRT1/NFE2L2/CXCL8/CHI3L1/CAMP | 5 |
| BP | GO:0050730 | | regulation of peptidyl-tyrosine phosphorylation | 6/97 | 264/18723 | 0.002516 | 0.010679 | 0.006243 | PECAM1/EGFR/TP53/TREM2/IL18/TNF | 6 |
| BP | GO:0002460 | | adaptive immune response based on somatic recombination of immune receptors built from immunoglobulin superfamily domains | 7/97 | 356/18723 | 0.002517 | 0.010679 | 0.006243 | NLRP3/FADD/RIPK3/TREM2/IL18/IL13RA2/TNF | 7 |
| BP | GO:0010631 | | epithelial cell migration | 7/97 | 357/18723 | 0.002557 | 0.010834 | 0.006333 | SIRT1/PTEN/NFE2L2/HDAC6/AKT1/APOE/TNF | 7 |
| BP | GO:0051260 | | protein homooligomerization | 5/97 | 182/18723 | 0.002574 | 0.010876 | 0.006358 | NLRP1/ELAVL1/GSDMD/PYCARD/GBP5 | 5 |
| BP | GO:0060401 | | cytosolic calcium ion transport | 5/97 | 182/18723 | 0.002574 | 0.010876 | 0.006358 | BCL2/P2RX7/GPER1/GSTO1/AKT1 | 5 |
| BP | GO:1903202 | | negative regulation of oxidative stress-induced cell death | 3/97 | 53/18723 | 0.002618 | 0.011047 | 0.006458 | SIRT1/NFE2L2/AKT1 | 3 |
| BP | GO:0009306 | | protein secretion | 7/97 | 359/18723 | 0.002638 | 0.011119 | 0.0065 | GPER1/EGFR/TLR2/APOE/TREM2/TNF/IL1RN | 7 |
| BP | GO:0034384 | | high-density lipoprotein particle clearance | 2/97 | 15/18723 | 0.002669 | 0.011152 | 0.00652 | APOE/TREM2 | 2 |
| BP | GO:0043518 | | negative regulation of DNA damage response, signal transduction by p53 class mediator | 2/97 | 15/18723 | 0.002669 | 0.011152 | 0.00652 | SIRT1/MDM2 | 2 |
| BP | GO:0045064 | | T-helper 2 cell differentiation | 2/97 | 15/18723 | 0.002669 | 0.011152 | 0.00652 | NLRP3/IL18 | 2 |
| BP | GO:0060099 | | regulation of phagocytosis, engulfment | 2/97 | 15/18723 | 0.002669 | 0.011152 | 0.00652 | ANO6/TREM2 | 2 |
| BP | GO:0097468 | | programmed cell death in response to reactive oxygen species | 2/97 | 15/18723 | 0.002669 | 0.011152 | 0.00652 | FOXO3/HDAC6 | 2 |
| BP | GO:0140374 | | antiviral innate immune response | 2/97 | 15/18723 | 0.002669 | 0.011152 | 0.00652 | NLRP1/CARD8 | 2 |
| BP | GO:1903799 | | negative regulation of production of miRNAs involved in gene silencing by miRNA | 2/97 | 15/18723 | 0.002669 | 0.011152 | 0.00652 | TP53/TNF | 2 |
| BP | GO:0035592 | | establishment of protein localization to extracellular region | 7/97 | 360/18723 | 0.002679 | 0.011164 | 0.006527 | GPER1/EGFR/TLR2/APOE/TREM2/TNF/IL1RN | 7 |
| BP | GO:0090132 | | epithelium migration | 7/97 | 360/18723 | 0.002679 | 0.011164 | 0.006527 | SIRT1/PTEN/NFE2L2/HDAC6/AKT1/APOE/TNF | 7 |
| BP | GO:0050708 | | regulation of protein secretion | 6/97 | 268/18723 | 0.002711 | 0.011282 | 0.006596 | GPER1/EGFR/TLR2/APOE/TREM2/TNF | 6 |
| BP | GO:0046632 | | alpha-beta T cell differentiation | 4/97 | 112/18723 | 0.002743 | 0.011401 | 0.006665 | BCL2/PRDM1/NLRP3/IL18 | 4 |
| BP | GO:0010524 | | positive regulation of calcium ion transport into cytosol | 3/97 | 54/18723 | 0.002761 | 0.011441 | 0.006689 | P2RX7/GPER1/GSTO1 | 3 |
| BP | GO:0043433 | | negative regulation of DNA-binding transcription factor activity | 5/97 | 185/18723 | 0.002762 | 0.011441 | 0.006689 | SIRT1/CARD8/NLRP3/PYCARD/AIM2 | 5 |
| BP | GO:0051251 | | positive regulation of lymphocyte activation | 7/97 | 362/18723 | 0.002763 | 0.011441 | 0.006689 | BCL2/NLRP3/FADD/AKT1/PYCARD/IL18/IL36B | 7 |
| BP | GO:0090130 | | tissue migration | 7/97 | 365/18723 | 0.002892 | 0.011962 | 0.006993 | SIRT1/PTEN/NFE2L2/HDAC6/AKT1/APOE/TNF | 7 |
| BP | GO:0010676 | | positive regulation of cellular carbohydrate metabolic process | 3/97 | 55/18723 | 0.00291 | 0.011988 | 0.007008 | SIRT1/GPER1/AKT1 | 3 |
| BP | GO:0051898 | | negative regulation of protein kinase B signaling | 3/97 | 55/18723 | 0.00291 | 0.011988 | 0.007008 | SIRT1/GPER1/PTEN | 3 |
| BP | GO:1902991 | | regulation of amyloid precursor protein catabolic process | 3/97 | 55/18723 | 0.00291 | 0.011988 | 0.007008 | CASP3/APOE/TNF | 3 |
| BP | GO:0048872 | | homeostasis of number of cells | 6/97 | 272/18723 | 0.002917 | 0.011988 | 0.007008 | BCL2/FOXO3/FADD/AKT1/RIPK3/CASP3 | 6 |
| BP | GO:0002244 | | hematopoietic progenitor cell differentiation | 4/97 | 114/18723 | 0.002924 | 0.011988 | 0.007008 | BCL2/NFE2L2/METTL3/TP53 | 4 |
| BP | GO:1901800 | | positive regulation of proteasomal protein catabolic process | 4/97 | 114/18723 | 0.002924 | 0.011988 | 0.007008 | NFE2L2/MDM2/AKT1/TREM2 | 4 |
| BP | GO:1903008 | | organelle disassembly | 4/97 | 114/18723 | 0.002924 | 0.011988 | 0.007008 | STING1/HDAC6/TP53/BNIP3 | 4 |
| BP | GO:0034249 | | negative regulation of cellular amide metabolic process | 6/97 | 273/18723 | 0.00297 | 0.012163 | 0.007111 | DDX3X/METTL3/SESN2/ORMDL3/APOE/GZMB | 6 |
| BP | GO:0043279 | | response to alkaloid | 4/97 | 115/18723 | 0.003017 | 0.012254 | 0.007164 | FADD/MDM2/CASP3/CASP6 | 4 |
| BP | GO:1901989 | | positive regulation of cell cycle phase transition | 4/97 | 115/18723 | 0.003017 | 0.012254 | 0.007164 | EGFR/DDX3X/MDM2/AKT1 | 4 |
| BP | GO:0071692 | | protein localization to extracellular region | 7/97 | 368/18723 | 0.003026 | 0.012254 | 0.007164 | GPER1/EGFR/TLR2/APOE/TREM2/TNF/IL1RN | 7 |
| BP | GO:0001991 | | regulation of systemic arterial blood pressure by circulatory renin-angiotensin | 2/97 | 16/18723 | 0.00304 | 0.012254 | 0.007164 | CTSG/ACE2 | 2 |
| BP | GO:0002830 | | positive regulation of type 2 immune response | 2/97 | 16/18723 | 0.00304 | 0.012254 | 0.007164 | NLRP3/IL18 | 2 |
| BP | GO:0010225 | | response to UV-C | 2/97 | 16/18723 | 0.00304 | 0.012254 | 0.007164 | MDM2/TP53 | 2 |
| BP | GO:0045651 | | positive regulation of macrophage differentiation | 2/97 | 16/18723 | 0.00304 | 0.012254 | 0.007164 | FADD/CASP8 | 2 |
| BP | GO:0051044 | | positive regulation of membrane protein ectodomain proteolysis | 2/97 | 16/18723 | 0.00304 | 0.012254 | 0.007164 | APOE/TNF | 2 |
| BP | GO:0055089 | | fatty acid homeostasis | 2/97 | 16/18723 | 0.00304 | 0.012254 | 0.007164 | SIRT1/APOE | 2 |
| BP | GO:1902165 | | regulation of intrinsic apoptotic signaling pathway in response to DNA damage by p53 class mediator | 2/97 | 16/18723 | 0.00304 | 0.012254 | 0.007164 | BCL2/SIRT1 | 2 |
| BP | GO:1902931 | | negative regulation of alcohol biosynthetic process | 2/97 | 16/18723 | 0.00304 | 0.012254 | 0.007164 | NFKB1/APOE | 2 |
| BP | GO:1905153 | | regulation of membrane invagination | 2/97 | 16/18723 | 0.00304 | 0.012254 | 0.007164 | ANO6/TREM2 | 2 |
| BP | GO:1905906 | | regulation of amyloid fibril formation | 2/97 | 16/18723 | 0.00304 | 0.012254 | 0.007164 | APOE/TREM2 | 2 |
| BP | GO:0002711 | | positive regulation of T cell mediated immunity | 3/97 | 56/18723 | 0.003063 | 0.012285 | 0.007182 | NLRP3/FADD/IL18 | 3 |
| BP | GO:0045071 | | negative regulation of viral genome replication | 3/97 | 56/18723 | 0.003063 | 0.012285 | 0.007182 | IFI16/BST2/TNF | 3 |
| BP | GO:0045599 | | negative regulation of fat cell differentiation | 3/97 | 56/18723 | 0.003063 | 0.012285 | 0.007182 | SIRT1/GPER1/TNF | 3 |
| BP | GO:1904645 | | response to amyloid-beta | 3/97 | 56/18723 | 0.003063 | 0.012285 | 0.007182 | FOXO3/PARP1/TREM2 | 3 |
| BP | GO:0002366 | | leukocyte activation involved in immune response | 6/97 | 275/18723 | 0.003079 | 0.012334 | 0.007211 | NLRP3/TP53/PYCARD/TREM2/IL18/IL13RA2 | 6 |
| BP | GO:0009755 | | hormone-mediated signaling pathway | 5/97 | 190/18723 | 0.003097 | 0.012392 | 0.007244 | SIRT1/GPER1/HDAC6/PARP1/TP63 | 5 |
| BP | GO:0007006 | | mitochondrial membrane organization | 4/97 | 116/18723 | 0.003113 | 0.012394 | 0.007246 | BCL2/TP53/BNIP3/GZMB | 4 |
| BP | GO:0018107 | | peptidyl-threonine phosphorylation | 4/97 | 116/18723 | 0.003113 | 0.012394 | 0.007246 | BCL2/AKT1/IRGM/CHI3L1 | 4 |
| BP | GO:0043200 | | response to amino acid | 4/97 | 116/18723 | 0.003113 | 0.012394 | 0.007246 | SESN2/CASP3/TNF/FGF21 | 4 |
| BP | GO:1990748 | | cellular detoxification | 4/97 | 116/18723 | 0.003113 | 0.012394 | 0.007246 | NFE2L2/GSTO1/SESN2/APOE | 4 |
| BP | GO:0007548 | | sex differentiation | 6/97 | 276/18723 | 0.003134 | 0.012466 | 0.007288 | BCL2/SIRT1/FOXO3/CASP3/TP63/CTSV | 6 |
| MF | GO:0140297 | | DNA-binding transcription factor binding | 8/97 | 394/18368 | 0.001145 | 0.012717 | 0.009902 | BCL2/JUN/SIRT1/NFE2L2/NLRP3/DHX9/TP53/PARP1 | 8 |
| BP | GO:0072676 | | lymphocyte migration | 4/97 | 117/18723 | 0.00321 | 0.012752 | 0.007455 | FADD/AKT1/RIPK3/PYCARD | 4 |
| BP | GO:0034205 | | amyloid-beta formation | 3/97 | 57/18723 | 0.003221 | 0.01278 | 0.007471 | CASP3/APOE/TNF | 3 |
| BP | GO:0032872 | | regulation of stress-activated MAPK cascade | 5/97 | 192/18723 | 0.003239 | 0.012836 | 0.007504 | EGFR/AKT1/PYCARD/TREM2/TNF | 5 |
| BP | GO:0007281 | | germ cell development | 6/97 | 278/18723 | 0.003248 | 0.012839 | 0.007506 | BCL2/PRDM1/FOXO3/METTL3/PANX1/AKT1 | 6 |
| BP | GO:0022898 | | regulation of transmembrane transporter activity | 6/97 | 278/18723 | 0.003248 | 0.012839 | 0.007506 | BCL2/PTEN/GSTO1/CAPN1/TREM2/ACE2 | 6 |
| BP | GO:0002263 | | cell activation involved in immune response | 6/97 | 279/18723 | 0.003305 | 0.013015 | 0.007609 | NLRP3/TP53/PYCARD/TREM2/IL18/IL13RA2 | 6 |
| BP | GO:0043542 | | endothelial cell migration | 6/97 | 279/18723 | 0.003305 | 0.013015 | 0.007609 | SIRT1/PTEN/NFE2L2/AKT1/APOE/TNF | 6 |
| BP | GO:0022612 | | gland morphogenesis | 4/97 | 118/18723 | 0.00331 | 0.013015 | 0.007609 | BCL2/CAPN1/TP63/TNF | 4 |
| BP | GO:0071346 | | cellular response to interferon-gamma | 4/97 | 118/18723 | 0.00331 | 0.013015 | 0.007609 | TP53/TLR2/IRGM/GBP5 | 4 |
| BP | GO:0050731 | | positive regulation of peptidyl-tyrosine phosphorylation | 5/97 | 193/18723 | 0.003312 | 0.013015 | 0.007609 | PECAM1/TP53/TREM2/IL18/TNF | 5 |
| MF | GO:0071723 | | lipopeptide binding | 2/97 | 10/18368 | 0.001208 | 0.013069 | 0.010175 | CD14/TLR2 | 2 |
| BP | GO:0002253 | | activation of immune response | 7/97 | 375/18723 | 0.003357 | 0.013162 | 0.007695 | BCL2/IFI16/STING1/PYCARD/TREM2/ZBP1/AIM2 | 7 |
| BP | GO:0018108 | | peptidyl-tyrosine phosphorylation | 7/97 | 375/18723 | 0.003357 | 0.013162 | 0.007695 | PECAM1/EGFR/TP53/TREM2/IL18/TNF/ALK | 7 |
| BP | GO:0070302 | | regulation of stress-activated protein kinase signaling cascade | 5/97 | 195/18723 | 0.003461 | 0.013552 | 0.007923 | EGFR/AKT1/PYCARD/TREM2/TNF | 5 |
| BP | GO:0018212 | | peptidyl-tyrosine modification | 7/97 | 378/18723 | 0.003507 | 0.013718 | 0.00802 | PECAM1/EGFR/TP53/TREM2/IL18/TNF/ALK | 7 |
| BP | GO:0051897 | | positive regulation of protein kinase B signaling | 4/97 | 120/18723 | 0.003515 | 0.013734 | 0.008029 | EGFR/CHI3L1/IL18/TNF | 4 |
| BP | GO:0022408 | | negative regulation of cell-cell adhesion | 5/97 | 196/18723 | 0.003537 | 0.013802 | 0.008069 | METTL3/GLMN/AKT1/CASP3/IL1RN | 5 |
| BP | GO:0072659 | | protein localization to plasma membrane | 6/97 | 284/18723 | 0.003606 | 0.014053 | 0.008216 | GPER1/EGFR/AKT1/TREM2/ANXA2/TNF | 6 |
| BP | GO:0043244 | | regulation of protein-containing complex disassembly | 4/97 | 121/18723 | 0.003621 | 0.014082 | 0.008232 | HDAC6/IRGM/BNIP3/TNF | 4 |
| BP | GO:0045931 | | positive regulation of mitotic cell cycle | 4/97 | 121/18723 | 0.003621 | 0.014082 | 0.008232 | EGFR/DDX3X/MDM2/AKT1 | 4 |
| BP | GO:0050678 | | regulation of epithelial cell proliferation | 7/97 | 381/18723 | 0.003662 | 0.014224 | 0.008316 | SIRT1/PTEN/EGFR/AKT1/APOE/TP63/TNF | 7 |
| MF | GO:0051400 | | BH domain binding | 2/97 | 11/18368 | 0.001472 | 0.014402 | 0.011213 | BCL2/IRGM | 2 |
| MF | GO:0070513 | | death domain binding | 2/97 | 11/18368 | 0.001472 | 0.014402 | 0.011213 | BCL2/IRGM | 2 |
| MF | GO:0097371 | | MDM2/MDM4 family protein binding | 2/97 | 11/18368 | 0.001472 | 0.014402 | 0.011213 | TP53/TP63 | 2 |
| MF | GO:1901612 | | cardiolipin binding | 2/97 | 11/18368 | 0.001472 | 0.014402 | 0.011213 | GSDME/GSDMD | 2 |
| MF | GO:0016504 | | peptidase activator activity | 3/97 | 43/18368 | 0.001512 | 0.014449 | 0.01125 | NLRP1/CARD8/PYCARD | 3 |
| BP | GO:0002753 | | cytoplasmic pattern recognition receptor signaling pathway | 3/97 | 60/18723 | 0.003725 | 0.014451 | 0.008449 | BIRC2/IRF3/IRGM | 3 |
| BP | GO:0042692 | | muscle cell differentiation | 7/97 | 384/18723 | 0.003823 | 0.014782 | 0.008642 | BCL2/SIRT1/GPER1/GLMN/MDM2/AKT1/CASP3 | 7 |
| BP | GO:0002923 | | regulation of humoral immune response mediated by circulating immunoglobulin | 2/97 | 18/18723 | 0.003851 | 0.014782 | 0.008642 | TREM2/TNF | 2 |
| BP | GO:0030728 | | ovulation | 2/97 | 18/18723 | 0.003851 | 0.014782 | 0.008642 | SIRT1/FOXO3 | 2 |
| BP | GO:0030730 | | sequestering of triglyceride | 2/97 | 18/18723 | 0.003851 | 0.014782 | 0.008642 | TREM2/TNF | 2 |
| BP | GO:0034134 | | toll-like receptor 2 signaling pathway | 2/97 | 18/18723 | 0.003851 | 0.014782 | 0.008642 | TLR2/TREM2 | 2 |
| BP | GO:0035743 | | CD4-positive, alpha-beta T cell cytokine production | 2/97 | 18/18723 | 0.003851 | 0.014782 | 0.008642 | NLRP3/IL18 | 2 |
| BP | GO:0043217 | | myelin maintenance | 2/97 | 18/18723 | 0.003851 | 0.014782 | 0.008642 | PTEN/AKT1 | 2 |
| BP | GO:0048535 | | lymph node development | 2/97 | 18/18723 | 0.003851 | 0.014782 | 0.008642 | FADD/RIPK3 | 2 |
| BP | GO:1903209 | | positive regulation of oxidative stress-induced cell death | 2/97 | 18/18723 | 0.003851 | 0.014782 | 0.008642 | FOXO3/HDAC6 | 2 |
| BP | GO:0060997 | | dendritic spine morphogenesis | 3/97 | 61/18723 | 0.003903 | 0.014915 | 0.00872 | PTEN/EEF2K/HDAC6 | 3 |
| BP | GO:0060998 | | regulation of dendritic spine development | 3/97 | 61/18723 | 0.003903 | 0.014915 | 0.00872 | PTEN/EEF2K/APOE | 3 |
| BP | GO:0071384 | | cellular response to corticosteroid stimulus | 3/97 | 61/18723 | 0.003903 | 0.014915 | 0.00872 | GPER1/EGFR/FOXO3 | 3 |
| BP | GO:0090342 | | regulation of cell aging | 3/97 | 61/18723 | 0.003903 | 0.014915 | 0.00872 | SIRT1/PTEN/TP53 | 3 |
| BP | GO:0010498 | | proteasomal protein catabolic process | 8/97 | 490/18723 | 0.003929 | 0.014996 | 0.008767 | SIRT1/NFE2L2/BIRC2/GLMN/MDM2/AKT1/APOE/TREM2 | 8 |
| BP | GO:0097237 | | cellular response to toxic substance | 4/97 | 124/18723 | 0.003952 | 0.015067 | 0.008809 | NFE2L2/GSTO1/SESN2/APOE | 4 |
| BP | GO:0071103 | | DNA conformation change | 6/97 | 290/18723 | 0.003992 | 0.015203 | 0.008888 | SIRT1/GPER1/DDX3X/MRE11/DHX9/TP53 | 6 |
| BP | GO:0018210 | | peptidyl-threonine modification | 4/97 | 125/18723 | 0.004067 | 0.015437 | 0.009025 | BCL2/AKT1/IRGM/CHI3L1 | 4 |
| BP | GO:0032613 | | interleukin-10 production | 3/97 | 62/18723 | 0.004087 | 0.015437 | 0.009025 | TLR2/PYCARD/TREM2 | 3 |
| BP | GO:0032615 | | interleukin-12 production | 3/97 | 62/18723 | 0.004087 | 0.015437 | 0.009025 | NFKB1/TLR2/MEFV | 3 |
| BP | GO:0032653 | | regulation of interleukin-10 production | 3/97 | 62/18723 | 0.004087 | 0.015437 | 0.009025 | TLR2/PYCARD/TREM2 | 3 |
| BP | GO:0032655 | | regulation of interleukin-12 production | 3/97 | 62/18723 | 0.004087 | 0.015437 | 0.009025 | NFKB1/TLR2/MEFV | 3 |
| BP | GO:0048002 | | antigen processing and presentation of peptide antigen | 3/97 | 62/18723 | 0.004087 | 0.015437 | 0.009025 | PYCARD/TREM2/CTSV | 3 |
| BP | GO:0060135 | | maternal process involved in female pregnancy | 3/97 | 62/18723 | 0.004087 | 0.015437 | 0.009025 | PRDM1/AKT1/CTSV | 3 |
| BP | GO:0032869 | | cellular response to insulin stimulus | 5/97 | 203/18723 | 0.004105 | 0.01549 | 0.009056 | SIRT1/PTEN/EEF2K/AKT1/PARP1 | 5 |
| BP | GO:1901361 | | organic cyclic compound catabolic process | 8/97 | 495/18723 | 0.004175 | 0.015737 | 0.0092 | NFE2L2/TET2/DHX9/METTL3/ELAVL1/AKT1/POP1/APOE | 8 |
| BP | GO:0031929 | | TOR signaling | 4/97 | 126/18723 | 0.004184 | 0.015751 | 0.009208 | SIRT1/SESN2/AKT1/TREM2 | 4 |
| BP | GO:1901888 | | regulation of cell junction assembly | 5/97 | 204/18723 | 0.004191 | 0.015762 | 0.009215 | PTEN/EEF2K/TLR2/ACE2/TNF | 5 |
| BP | GO:0010948 | | negative regulation of cell cycle process | 6/97 | 294/18723 | 0.004267 | 0.01593 | 0.009313 | BCL2/GPER1/PTEN/MRE11/MDM2/TP53 | 6 |
| BP | GO:0070059 | | intrinsic apoptotic signaling pathway in response to endoplasmic reticulum stress | 3/97 | 63/18723 | 0.004275 | 0.01593 | 0.009313 | BCL2/SIRT1/TP53 | 3 |
| BP | GO:0002902 | | regulation of B cell apoptotic process | 2/97 | 19/18723 | 0.004289 | 0.01593 | 0.009313 | PTEN/ORMDL3 | 2 |
| BP | GO:0006977 | | DNA damage response, signal transduction by p53 class mediator resulting in cell cycle arrest | 2/97 | 19/18723 | 0.004289 | 0.01593 | 0.009313 | MDM2/TP53 | 2 |
| BP | GO:0010744 | | positive regulation of macrophage derived foam cell differentiation | 2/97 | 19/18723 | 0.004289 | 0.01593 | 0.009313 | NFKB1/IL18 | 2 |
| BP | GO:0014067 | | negative regulation of phosphatidylinositol 3-kinase signaling | 2/97 | 19/18723 | 0.004289 | 0.01593 | 0.009313 | PTEN/TREM2 | 2 |
| BP | GO:0032026 | | response to magnesium ion | 2/97 | 19/18723 | 0.004289 | 0.01593 | 0.009313 | MDM2/CD14 | 2 |
| BP | GO:0032695 | | negative regulation of interleukin-12 production | 2/97 | 19/18723 | 0.004289 | 0.01593 | 0.009313 | NFKB1/MEFV | 2 |
| BP | GO:0044003 | | modulation by symbiont of host process | 2/97 | 19/18723 | 0.004289 | 0.01593 | 0.009313 | ANO6/IRGM | 2 |
| BP | GO:0044320 | | cellular response to leptin stimulus | 2/97 | 19/18723 | 0.004289 | 0.01593 | 0.009313 | SIRT1/PTEN | 2 |
| BP | GO:2000269 | | regulation of fibroblast apoptotic process | 2/97 | 19/18723 | 0.004289 | 0.01593 | 0.009313 | TP53/TP63 | 2 |
| MF | GO:0035325 | | Toll-like receptor binding | 2/97 | 12/18368 | 0.00176 | 0.016075 | 0.012516 | LY96/TLR2 | 2 |
| MF | GO:0089720 | | caspase binding | 2/97 | 12/18368 | 0.00176 | 0.016075 | 0.012516 | FADD/NLRP7 | 2 |
| BP | GO:0017038 | | protein import | 5/97 | 206/18723 | 0.004368 | 0.016204 | 0.009473 | TXNIP/ELAVL1/AKT1/TP53/APOE | 5 |
| BP | GO:1901215 | | negative regulation of neuron death | 5/97 | 208/18723 | 0.004549 | 0.016858 | 0.009856 | BCL2/SIRT1/AKT1/APOE/FGF21 | 5 |
| BP | GO:0010720 | | positive regulation of cell development | 6/97 | 298/18723 | 0.004555 | 0.01686 | 0.009857 | BCL2/GPER1/EGFR/EEF2K/TLR2/TNF | 6 |
| BP | GO:0002720 | | positive regulation of cytokine production involved in immune response | 3/97 | 65/18723 | 0.004667 | 0.017225 | 0.01007 | SIRT1/NLRP3/IL18 | 3 |
| BP | GO:0070613 | | regulation of protein processing | 3/97 | 65/18723 | 0.004667 | 0.017225 | 0.01007 | CARD8/MDM2/NLRP7 | 3 |
| BP | GO:0006282 | | regulation of DNA repair | 4/97 | 130/18723 | 0.004674 | 0.017225 | 0.01007 | SIRT1/EGFR/DHX9/PARP1 | 4 |
| BP | GO:0043467 | | regulation of generation of precursor metabolites and energy | 4/97 | 130/18723 | 0.004674 | 0.017225 | 0.01007 | P2RX7/AKT1/TP53/BNIP3 | 4 |
| BP | GO:0031396 | | regulation of protein ubiquitination | 5/97 | 210/18723 | 0.004736 | 0.017254 | 0.010087 | PTEN/DDX3X/BIRC2/GLMN/AKT1 | 5 |
| BP | GO:0002577 | | regulation of antigen processing and presentation | 2/97 | 20/18723 | 0.00475 | 0.017254 | 0.010087 | PYCARD/TREM2 | 2 |
| BP | GO:0009110 | | vitamin biosynthetic process | 2/97 | 20/18723 | 0.00475 | 0.017254 | 0.010087 | NFKB1/TNF | 2 |
| BP | GO:0010523 | | negative regulation of calcium ion transport into cytosol | 2/97 | 20/18723 | 0.00475 | 0.017254 | 0.010087 | BCL2/GSTO1 | 2 |
| BP | GO:0017121 | | plasma membrane phospholipid scrambling | 2/97 | 20/18723 | 0.00475 | 0.017254 | 0.010087 | ANO6/P2RX7 | 2 |
| BP | GO:0042359 | | vitamin D metabolic process | 2/97 | 20/18723 | 0.00475 | 0.017254 | 0.010087 | NFKB1/TNF | 2 |
| BP | GO:0043555 | | regulation of translation in response to stress | 2/97 | 20/18723 | 0.00475 | 0.017254 | 0.010087 | DDX3X/SESN2 | 2 |
| BP | GO:0051900 | | regulation of mitochondrial depolarization | 2/97 | 20/18723 | 0.00475 | 0.017254 | 0.010087 | BCL2/PARP1 | 2 |
| BP | GO:0060252 | | positive regulation of glial cell proliferation | 2/97 | 20/18723 | 0.00475 | 0.017254 | 0.010087 | EGFR/TNF | 2 |
| BP | GO:0070932 | | histone H3 deacetylation | 2/97 | 20/18723 | 0.00475 | 0.017254 | 0.010087 | SIRT1/HDAC6 | 2 |
| BP | GO:0071243 | | cellular response to arsenic-containing substance | 2/97 | 20/18723 | 0.00475 | 0.017254 | 0.010087 | DDX3X/GSTO1 | 2 |
| BP | GO:1902004 | | positive regulation of amyloid-beta formation | 2/97 | 20/18723 | 0.00475 | 0.017254 | 0.010087 | CASP3/TNF | 2 |
| BP | GO:2000010 | | positive regulation of protein localization to cell surface | 2/97 | 20/18723 | 0.00475 | 0.017254 | 0.010087 | AKT1/TNF | 2 |
| BP | GO:0006913 | | nucleocytoplasmic transport | 6/97 | 301/18723 | 0.00478 | 0.017326 | 0.010129 | TXNIP/DHX9/ELAVL1/MDM2/AKT1/TP53 | 6 |
| BP | GO:0051169 | | nuclear transport | 6/97 | 301/18723 | 0.00478 | 0.017326 | 0.010129 | TXNIP/DHX9/ELAVL1/MDM2/AKT1/TP53 | 6 |
| BP | GO:0019827 | | stem cell population maintenance | 4/97 | 131/18723 | 0.004803 | 0.017389 | 0.010166 | FOXO3/METTL3/ELAVL1/TP63 | 4 |
| BP | GO:0014015 | | positive regulation of gliogenesis | 3/97 | 66/18723 | 0.004871 | 0.017561 | 0.010267 | EGFR/TLR2/TNF | 3 |
| BP | GO:0019229 | | regulation of vasoconstriction | 3/97 | 66/18723 | 0.004871 | 0.017561 | 0.010267 | EGFR/AKT1/ACE2 | 3 |
| BP | GO:0072678 | | T cell migration | 3/97 | 66/18723 | 0.004871 | 0.017561 | 0.010267 | FADD/RIPK3/PYCARD | 3 |
| BP | GO:2000272 | | negative regulation of signaling receptor activity | 3/97 | 66/18723 | 0.004871 | 0.017561 | 0.010267 | PTEN/ACE2/TNF | 3 |
| BP | GO:0007162 | | negative regulation of cell adhesion | 6/97 | 303/18723 | 0.004935 | 0.017751 | 0.010377 | PTEN/METTL3/GLMN/AKT1/CASP3/IL1RN | 6 |
| BP | GO:0060070 | | canonical Wnt signaling pathway | 6/97 | 303/18723 | 0.004935 | 0.017751 | 0.010377 | PTEN/EGFR/NFKB1/DDX3X/FOXO3/APOE | 6 |
| BP | GO:0046328 | | regulation of JNK cascade | 4/97 | 133/18723 | 0.005066 | 0.018205 | 0.010643 | EGFR/AKT1/PYCARD/TNF | 4 |
| BP | GO:0050435 | | amyloid-beta metabolic process | 3/97 | 67/18723 | 0.005081 | 0.018217 | 0.01065 | CASP3/APOE/TNF | 3 |
| BP | GO:1903317 | | regulation of protein maturation | 3/97 | 67/18723 | 0.005081 | 0.018217 | 0.01065 | CARD8/MDM2/NLRP7 | 3 |
| MF | GO:0061133 | | endopeptidase activator activity | 2/97 | 13/18368 | 0.002073 | 0.01852 | 0.01442 | NLRP1/CARD8 | 2 |
| BP | GO:0032434 | | regulation of proteasomal ubiquitin-dependent protein catabolic process | 4/97 | 134/18723 | 0.005202 | 0.018591 | 0.010869 | NFE2L2/GLMN/MDM2/AKT1 | 4 |
| BP | GO:0042552 | | myelination | 4/97 | 134/18723 | 0.005202 | 0.018591 | 0.010869 | PTEN/AKT1/ORMDL3/TLR2 | 4 |
| BP | GO:0098727 | | maintenance of cell number | 4/97 | 134/18723 | 0.005202 | 0.018591 | 0.010869 | FOXO3/METTL3/ELAVL1/TP63 | 4 |
| BP | GO:0006925 | | inflammatory cell apoptotic process | 2/97 | 21/18723 | 0.005232 | 0.018598 | 0.010873 | SIRT1/IRF3 | 2 |
| BP | GO:0022010 | | central nervous system myelination | 2/97 | 21/18723 | 0.005232 | 0.018598 | 0.010873 | PTEN/TLR2 | 2 |
| BP | GO:0032291 | | axon ensheathment in central nervous system | 2/97 | 21/18723 | 0.005232 | 0.018598 | 0.010873 | PTEN/TLR2 | 2 |
| BP | GO:0051152 | | positive regulation of smooth muscle cell differentiation | 2/97 | 21/18723 | 0.005232 | 0.018598 | 0.010873 | SIRT1/GPER1 | 2 |
| BP | GO:0097062 | | dendritic spine maintenance | 2/97 | 21/18723 | 0.005232 | 0.018598 | 0.010873 | APOE/TREM2 | 2 |
| BP | GO:0042267 | | natural killer cell mediated cytotoxicity | 3/97 | 68/18723 | 0.005296 | 0.018724 | 0.010946 | LYST/GZMB/IL18 | 3 |
| BP | GO:0042987 | | amyloid precursor protein catabolic process | 3/97 | 68/18723 | 0.005296 | 0.018724 | 0.010946 | CASP3/APOE/TNF | 3 |
| BP | GO:0046637 | | regulation of alpha-beta T cell differentiation | 3/97 | 68/18723 | 0.005296 | 0.018724 | 0.010946 | PRDM1/NLRP3/IL18 | 3 |
| BP | GO:0046686 | | response to cadmium ion | 3/97 | 68/18723 | 0.005296 | 0.018724 | 0.010946 | JUN/EGFR/AKT1 | 3 |
| BP | GO:0051926 | | negative regulation of calcium ion transport | 3/97 | 68/18723 | 0.005296 | 0.018724 | 0.010946 | BCL2/GSTO1/AKT1 | 3 |
| BP | GO:0002440 | | production of molecular mediator of immune response | 6/97 | 308/18723 | 0.005337 | 0.01884 | 0.011014 | SIRT1/NLRP3/BST2/IL18/IL13RA2/TNF | 6 |
| BP | GO:0001101 | | response to acid chemical | 4/97 | 135/18723 | 0.00534 | 0.01884 | 0.011014 | SESN2/CASP3/TNF/FGF21 | 4 |
| BP | GO:0007272 | | ensheathment of neurons | 4/97 | 136/18723 | 0.00548 | 0.019273 | 0.011268 | PTEN/AKT1/ORMDL3/TLR2 | 4 |
| BP | GO:0008366 | | axon ensheathment | 4/97 | 136/18723 | 0.00548 | 0.019273 | 0.011268 | PTEN/AKT1/ORMDL3/TLR2 | 4 |
| BP | GO:1900180 | | regulation of protein localization to nucleus | 4/97 | 136/18723 | 0.00548 | 0.019273 | 0.011268 | SESN2/AKT1/ORMDL3/PARP1 | 4 |
| BP | GO:0032409 | | regulation of transporter activity | 6/97 | 310/18723 | 0.005505 | 0.019341 | 0.011307 | BCL2/PTEN/GSTO1/CAPN1/TREM2/ACE2 | 6 |
| BP | GO:0033344 | | cholesterol efflux | 3/97 | 69/18723 | 0.005516 | 0.019357 | 0.011317 | SIRT1/APOE/TREM2 | 3 |
| BP | GO:0050671 | | positive regulation of lymphocyte proliferation | 4/97 | 137/18723 | 0.005623 | 0.019692 | 0.011512 | BCL2/FADD/PYCARD/IL18 | 4 |
| BP | GO:0050714 | | positive regulation of protein secretion | 4/97 | 137/18723 | 0.005623 | 0.019692 | 0.011512 | GPER1/EGFR/TLR2/TREM2 | 4 |
| CC | GO:0031968 | | organelle outer membrane | 6/97 | 230/19550 | 0.001007 | 0.019918 | 0.016163 | BCL2/STING1/FOXO3/CPTP/CASP8/BNIP3 | 6 |
| CC | GO:0019867 | | outer membrane | 6/97 | 232/19550 | 0.001053 | 0.019918 | 0.016163 | BCL2/STING1/FOXO3/CPTP/CASP8/BNIP3 | 6 |
| BP | GO:0007413 | | axonal fasciculation | 2/97 | 22/18723 | 0.005736 | 0.019982 | 0.011682 | CRTAC1/CASP3 | 2 |
| BP | GO:0032069 | | regulation of nuclease activity | 2/97 | 22/18723 | 0.005736 | 0.019982 | 0.011682 | SIRT1/AKT1 | 2 |
| BP | GO:0044346 | | fibroblast apoptotic process | 2/97 | 22/18723 | 0.005736 | 0.019982 | 0.011682 | TP53/TP63 | 2 |
| BP | GO:0045624 | | positive regulation of T-helper cell differentiation | 2/97 | 22/18723 | 0.005736 | 0.019982 | 0.011682 | NLRP3/IL18 | 2 |
| BP | GO:0106030 | | neuron projection fasciculation | 2/97 | 22/18723 | 0.005736 | 0.019982 | 0.011682 | CRTAC1/CASP3 | 2 |
| BP | GO:0002064 | | epithelial cell development | 5/97 | 220/18723 | 0.005753 | 0.02002 | 0.011704 | PECAM1/PRDM1/AKT1/TP63/TNF | 5 |
| BP | GO:0032946 | | positive regulation of mononuclear cell proliferation | 4/97 | 138/18723 | 0.005768 | 0.020052 | 0.011723 | BCL2/FADD/PYCARD/IL18 | 4 |
| MF | GO:1990782 | | protein tyrosine kinase binding | 4/97 | 105/18368 | 0.002324 | 0.020327 | 0.015826 | PTEN/TP53/MST1/TREM2 | 4 |
| BP | GO:0008406 | | gonad development | 5/97 | 221/18723 | 0.005862 | 0.020358 | 0.011902 | BCL2/SIRT1/FOXO3/CASP3/CTSV | 5 |
| MF | GO:0140666 | | annealing activity | 2/97 | 14/18368 | 0.00241 | 0.020636 | 0.016067 | DDX3X/TP53 | 2 |
| BP | GO:0002228 | | natural killer cell mediated immunity | 3/97 | 71/18723 | 0.005972 | 0.020697 | 0.0121 | LYST/GZMB/IL18 | 3 |
| BP | GO:0033555 | | multicellular organismal response to stress | 3/97 | 71/18723 | 0.005972 | 0.020697 | 0.0121 | BCL2/PTEN/APOE | 3 |
| BP | GO:0015748 | | organophosphate ester transport | 4/97 | 140/18723 | 0.006066 | 0.020956 | 0.012251 | ANO6/P2RX7/CPTP/APOE | 4 |
| BP | GO:0030177 | | positive regulation of Wnt signaling pathway | 4/97 | 140/18723 | 0.006066 | 0.020956 | 0.012251 | EGFR/NFKB1/DDX3X/TLR2 | 4 |
| BP | GO:0050768 | | negative regulation of neurogenesis | 4/97 | 140/18723 | 0.006066 | 0.020956 | 0.012251 | PTEN/TP53/TREM2/TNF | 4 |
| BP | GO:0006959 | | humoral immune response | 6/97 | 317/18723 | 0.006123 | 0.02113 | 0.012353 | CTSG/BCL2/CXCL8/TREM2/TNF/CAMP | 6 |
| BP | GO:0034329 | | cell junction assembly | 7/97 | 420/18723 | 0.006191 | 0.021344 | 0.012478 | BCL2/PECAM1/PTEN/EEF2K/TLR2/ACE2/TNF | 7 |
| BP | GO:0003081 | | regulation of systemic arterial blood pressure by renin-angiotensin | 2/97 | 23/18723 | 0.006261 | 0.021407 | 0.012515 | CTSG/ACE2 | 2 |
| BP | GO:0010888 | | negative regulation of lipid storage | 2/97 | 23/18723 | 0.006261 | 0.021407 | 0.012515 | TREM2/TNF | 2 |
| BP | GO:0030194 | | positive regulation of blood coagulation | 2/97 | 23/18723 | 0.006261 | 0.021407 | 0.012515 | ANO6/NFE2L2 | 2 |
| BP | GO:0045662 | | negative regulation of myoblast differentiation | 2/97 | 23/18723 | 0.006261 | 0.021407 | 0.012515 | IL18/TNF | 2 |
| BP | GO:0050765 | | negative regulation of phagocytosis | 2/97 | 23/18723 | 0.006261 | 0.021407 | 0.012515 | PTEN/TLR2 | 2 |
| BP | GO:0051882 | | mitochondrial depolarization | 2/97 | 23/18723 | 0.006261 | 0.021407 | 0.012515 | BCL2/PARP1 | 2 |
| BP | GO:1900048 | | positive regulation of hemostasis | 2/97 | 23/18723 | 0.006261 | 0.021407 | 0.012515 | ANO6/NFE2L2 | 2 |
| BP | GO:2001169 | | regulation of ATP biosynthetic process | 2/97 | 23/18723 | 0.006261 | 0.021407 | 0.012515 | PARP1/TREM2 | 2 |
| MF | GO:0001228 | | DNA-binding transcription activator activity, RNA polymerase II-specific | 8/97 | 450/18368 | 0.002631 | 0.02149 | 0.016732 | JUN/NFKB1/NFE2L2/FOXO3/IRF2/IRF3/TP53/TP63 | 8 |
| MF | GO:0004866 | | endopeptidase inhibitor activity | 5/97 | 180/18368 | 0.002663 | 0.02149 | 0.016732 | BIRC2/SERPINB1/ANXA2/BST2/NLRP7 | 5 |
| MF | GO:0047485 | | protein N-terminus binding | 4/97 | 110/18368 | 0.002752 | 0.02149 | 0.016732 | BIRC2/MDM2/TP53/PARP1 | 4 |
| MF | GO:0017136 | | NAD-dependent histone deacetylase activity | 2/97 | 15/18368 | 0.002771 | 0.02149 | 0.016732 | SIRT1/HDAC6 | 2 |
| MF | GO:1901611 | | phosphatidylglycerol binding | 2/97 | 15/18368 | 0.002771 | 0.02149 | 0.016732 | GSDME/GSDMD | 2 |
| BP | GO:0050769 | | positive regulation of neurogenesis | 5/97 | 225/18723 | 0.006315 | 0.021569 | 0.012609 | GPER1/EGFR/EEF2K/TLR2/TNF | 5 |
| MF | GO:0001216 | | DNA-binding transcription activator activity | 8/97 | 456/18368 | 0.002853 | 0.021713 | 0.016906 | JUN/NFKB1/NFE2L2/FOXO3/IRF2/IRF3/TP53/TP63 | 8 |
| BP | GO:0007009 | | plasma membrane organization | 4/97 | 142/18723 | 0.006374 | 0.021748 | 0.012714 | ANO6/P2RX7/PTEN/AKT1 | 4 |
| BP | GO:0016042 | | lipid catabolic process | 6/97 | 320/18723 | 0.006402 | 0.021822 | 0.012758 | SESN2/AKT1/APOE/TNF/ALK/FGF21 | 6 |
| BP | GO:0034763 | | negative regulation of transmembrane transport | 4/97 | 143/18723 | 0.006532 | 0.022241 | 0.013002 | PTEN/GSTO1/AKT1/TNF | 4 |
| BP | GO:0045137 | | development of primary sexual characteristics | 5/97 | 227/18723 | 0.00655 | 0.022281 | 0.013026 | BCL2/SIRT1/FOXO3/CASP3/CTSV | 5 |
| BP | GO:0050808 | | synapse organization | 7/97 | 426/18723 | 0.006674 | 0.022678 | 0.013258 | PTEN/EEF2K/HDAC6/TLR2/APOE/TREM2/TNF | 7 |
| CC | GO:0043220 | | Schmidt-Lanterman incisure | 2/97 | 11/19550 | 0.001302 | 0.022727 | 0.018443 | PTEN/ANXA2 | 2 |
| BP | GO:0006801 | | superoxide metabolic process | 3/97 | 74/18723 | 0.006699 | 0.022738 | 0.013293 | EGFR/NFE2L2/AKT1 | 3 |
| MF | GO:0030414 | | peptidase inhibitor activity | 5/97 | 187/18368 | 0.003138 | 0.022758 | 0.017719 | BIRC2/SERPINB1/ANXA2/BST2/NLRP7 | 5 |
| MF | GO:0034979 | | NAD-dependent protein deacetylase activity | 2/97 | 16/18368 | 0.003156 | 0.022758 | 0.017719 | SIRT1/HDAC6 | 2 |
| MF | GO:0050700 | | CARD domain binding | 2/97 | 16/18368 | 0.003156 | 0.022758 | 0.017719 | CARD8/IRGM | 2 |
| BP | GO:0008544 | | epidermis development | 6/97 | 324/18723 | 0.006789 | 0.022827 | 0.013345 | TXNIP/BCL2/EGFR/CASP3/TP63/TNF | 6 |
| BP | GO:0001649 | | osteoblast differentiation | 5/97 | 229/18723 | 0.006792 | 0.022827 | 0.013345 | DHX9/AKT1/CLEC5A/TP63/TNF | 5 |
| BP | GO:0001783 | | B cell apoptotic process | 2/97 | 24/18723 | 0.006807 | 0.022827 | 0.013345 | PTEN/ORMDL3 | 2 |
| BP | GO:0002726 | | positive regulation of T cell cytokine production | 2/97 | 24/18723 | 0.006807 | 0.022827 | 0.013345 | NLRP3/IL18 | 2 |
| BP | GO:0002922 | | positive regulation of humoral immune response | 2/97 | 24/18723 | 0.006807 | 0.022827 | 0.013345 | TREM2/TNF | 2 |
| BP | GO:0016486 | | peptide hormone processing | 2/97 | 24/18723 | 0.006807 | 0.022827 | 0.013345 | CTSG/ACE2 | 2 |
| BP | GO:0032928 | | regulation of superoxide anion generation | 2/97 | 24/18723 | 0.006807 | 0.022827 | 0.013345 | EGFR/AKT1 | 2 |
| BP | GO:0044321 | | response to leptin | 2/97 | 24/18723 | 0.006807 | 0.022827 | 0.013345 | SIRT1/PTEN | 2 |
| BP | GO:0045649 | | regulation of macrophage differentiation | 2/97 | 24/18723 | 0.006807 | 0.022827 | 0.013345 | FADD/CASP8 | 2 |
| BP | GO:0050820 | | positive regulation of coagulation | 2/97 | 24/18723 | 0.006807 | 0.022827 | 0.013345 | ANO6/NFE2L2 | 2 |
| BP | GO:0051043 | | regulation of membrane protein ectodomain proteolysis | 2/97 | 24/18723 | 0.006807 | 0.022827 | 0.013345 | APOE/TNF | 2 |
| BP | GO:0090343 | | positive regulation of cell aging | 2/97 | 24/18723 | 0.006807 | 0.022827 | 0.013345 | SIRT1/TP53 | 2 |
| BP | GO:0006094 | | gluconeogenesis | 3/97 | 75/18723 | 0.006952 | 0.023264 | 0.0136 | SIRT1/SESN2/MST1 | 3 |
| BP | GO:0048662 | | negative regulation of smooth muscle cell proliferation | 3/97 | 75/18723 | 0.006952 | 0.023264 | 0.0136 | GPER1/PTEN/APOE | 3 |
| BP | GO:0010594 | | regulation of endothelial cell migration | 5/97 | 232/18723 | 0.007166 | 0.023955 | 0.014005 | SIRT1/NFE2L2/AKT1/APOE/TNF | 5 |
| BP | GO:0030111 | | regulation of Wnt signaling pathway | 6/97 | 328/18723 | 0.007193 | 0.024024 | 0.014045 | EGFR/NFKB1/DDX3X/FOXO3/TLR2/APOE | 6 |
| BP | GO:0043536 | | positive regulation of blood vessel endothelial cell migration | 3/97 | 76/18723 | 0.007211 | 0.024057 | 0.014064 | SIRT1/NFE2L2/AKT1 | 3 |
| BP | GO:0031664 | | regulation of lipopolysaccharide-mediated signaling pathway | 2/97 | 25/18723 | 0.007374 | 0.024432 | 0.014283 | LY96/CARD8 | 2 |
| BP | GO:0035902 | | response to immobilization stress | 2/97 | 25/18723 | 0.007374 | 0.024432 | 0.014283 | FOXO3/MDM2 | 2 |
| BP | GO:0060706 | | cell differentiation involved in embryonic placenta development | 2/97 | 25/18723 | 0.007374 | 0.024432 | 0.014283 | PRDM1/CASP8 | 2 |
| BP | GO:0070423 | | nucleotide-binding oligomerization domain containing signaling pathway | 2/97 | 25/18723 | 0.007374 | 0.024432 | 0.014283 | BIRC2/IRGM | 2 |
| BP | GO:0140448 | | signaling receptor ligand precursor processing | 2/97 | 25/18723 | 0.007374 | 0.024432 | 0.014283 | CTSG/ACE2 | 2 |
| BP | GO:1902993 | | positive regulation of amyloid precursor protein catabolic process | 2/97 | 25/18723 | 0.007374 | 0.024432 | 0.014283 | CASP3/TNF | 2 |
| BP | GO:1904385 | | cellular response to angiotensin | 2/97 | 25/18723 | 0.007374 | 0.024432 | 0.014283 | NFKB1/NFE2L2 | 2 |
| BP | GO:0045834 | | positive regulation of lipid metabolic process | 4/97 | 149/18723 | 0.007534 | 0.024934 | 0.014577 | AKT1/APOE/TNF/FGF21 | 4 |
| BP | GO:0045785 | | positive regulation of cell adhesion | 7/97 | 437/18723 | 0.007631 | 0.025231 | 0.014751 | NLRP3/FADD/AKT1/PYCARD/IL18/TNF/IL36B | 7 |
| BP | GO:1990778 | | protein localization to cell periphery | 6/97 | 333/18723 | 0.007722 | 0.025508 | 0.014912 | GPER1/EGFR/AKT1/TREM2/ANXA2/TNF | 6 |
| BP | GO:0019319 | | hexose biosynthetic process | 3/97 | 78/18723 | 0.007745 | 0.025558 | 0.014942 | SIRT1/SESN2/MST1 | 3 |
| BP | GO:0031334 | | positive regulation of protein-containing complex assembly | 5/97 | 237/18723 | 0.00782 | 0.025754 | 0.015056 | DDX3X/TP53/PYCARD/GBP5/TNF | 5 |
| BP | GO:1901617 | | organic hydroxy compound biosynthetic process | 5/97 | 237/18723 | 0.00782 | 0.025754 | 0.015056 | SIRT1/GPER1/NFKB1/APOE/TNF | 5 |
| BP | GO:0044409 | | entry into host | 4/97 | 151/18723 | 0.007889 | 0.025929 | 0.015158 | EGFR/CXCL8/ACE2/CLEC5A | 4 |
| BP | GO:0071333 | | cellular response to glucose stimulus | 4/97 | 151/18723 | 0.007889 | 0.025929 | 0.015158 | GPER1/FOXO3/TREM2/FGF21 | 4 |
| BP | GO:0034114 | | regulation of heterotypic cell-cell adhesion | 2/97 | 26/18723 | 0.007962 | 0.02604 | 0.015224 | TNF/IL1RN | 2 |
| BP | GO:0035872 | | nucleotide-binding domain, leucine rich repeat containing receptor signaling pathway | 2/97 | 26/18723 | 0.007962 | 0.02604 | 0.015224 | BIRC2/IRGM | 2 |
| BP | GO:0045672 | | positive regulation of osteoclast differentiation | 2/97 | 26/18723 | 0.007962 | 0.02604 | 0.015224 | TREM2/TNF | 2 |
| BP | GO:1900017 | | positive regulation of cytokine production involved in inflammatory response | 2/97 | 26/18723 | 0.007962 | 0.02604 | 0.015224 | GBP5/TNF | 2 |
| BP | GO:2000108 | | positive regulation of leukocyte apoptotic process | 2/97 | 26/18723 | 0.007962 | 0.02604 | 0.015224 | SIRT1/TP53 | 2 |
| BP | GO:0006446 | | regulation of translational initiation | 3/97 | 79/18723 | 0.008021 | 0.026208 | 0.015321 | DDX3X/METTL3/TNF | 3 |
| BP | GO:0098754 | | detoxification | 4/97 | 152/18723 | 0.008071 | 0.026343 | 0.0154 | NFE2L2/GSTO1/SESN2/APOE | 4 |
| CC | GO:0016605 | | PML body | 4/97 | 103/19550 | 0.00173 | 0.026362 | 0.021393 | SIRT1/PTEN/MRE11/TP53 | 4 |
| CC | GO:0043209 | | myelin sheath | 3/97 | 48/19550 | 0.001742 | 0.026362 | 0.021393 | BCL2/PTEN/ANXA2 | 3 |
| BP | GO:0071331 | | cellular response to hexose stimulus | 4/97 | 153/18723 | 0.008255 | 0.026918 | 0.015737 | GPER1/FOXO3/TREM2/FGF21 | 4 |
| BP | GO:0001655 | | urogenital system development | 6/97 | 338/18723 | 0.008279 | 0.026942 | 0.015751 | BCL2/PECAM1/PRDM1/PTEN/FADD/TP63 | 6 |
| BP | GO:0016055 | | Wnt signaling pathway | 7/97 | 444/18723 | 0.008291 | 0.026942 | 0.015751 | PTEN/EGFR/NFKB1/DDX3X/FOXO3/TLR2/APOE | 7 |
| BP | GO:0021766 | | hippocampus development | 3/97 | 80/18723 | 0.008303 | 0.026942 | 0.015751 | PTEN/CASP3/ALK | 3 |
| BP | GO:0032204 | | regulation of telomere maintenance | 3/97 | 80/18723 | 0.008303 | 0.026942 | 0.015751 | NEK7/MRE11/PARP1 | 3 |
| BP | GO:0048145 | | regulation of fibroblast proliferation | 3/97 | 80/18723 | 0.008303 | 0.026942 | 0.015751 | DHX9/TP53/ANXA2 | 3 |
| BP | GO:0010975 | | regulation of neuron projection development | 7/97 | 445/18723 | 0.008389 | 0.027166 | 0.015882 | PTEN/NFE2L2/EEF2K/MDM2/AKT1/APOE/ALK | 7 |
| BP | GO:0046700 | | heterocycle catabolic process | 7/97 | 445/18723 | 0.008389 | 0.027166 | 0.015882 | NFE2L2/TET2/DHX9/METTL3/ELAVL1/AKT1/POP1 | 7 |
| BP | GO:0071326 | | cellular response to monosaccharide stimulus | 4/97 | 154/18723 | 0.008442 | 0.027313 | 0.015968 | GPER1/FOXO3/TREM2/FGF21 | 4 |
| BP | GO:0198738 | | cell-cell signaling by wnt | 7/97 | 446/18723 | 0.008487 | 0.027313 | 0.015968 | PTEN/EGFR/NFKB1/DDX3X/FOXO3/TLR2/APOE | 7 |
| BP | GO:1903320 | | regulation of protein modification by small protein conjugation or removal | 5/97 | 242/18723 | 0.008515 | 0.027313 | 0.015968 | PTEN/DDX3X/BIRC2/GLMN/AKT1 | 5 |
| BP | GO:0002719 | | negative regulation of cytokine production involved in immune response | 2/97 | 27/18723 | 0.00857 | 0.027313 | 0.015968 | BST2/TNF | 2 |
| BP | GO:0007263 | | nitric oxide mediated signal transduction | 2/97 | 27/18723 | 0.00857 | 0.027313 | 0.015968 | EGFR/APOE | 2 |
| BP | GO:0009651 | | response to salt stress | 2/97 | 27/18723 | 0.00857 | 0.027313 | 0.015968 | TP53/TNF | 2 |
| BP | GO:0010894 | | negative regulation of steroid biosynthetic process | 2/97 | 27/18723 | 0.00857 | 0.027313 | 0.015968 | NFKB1/APOE | 2 |
| BP | GO:0031954 | | positive regulation of protein autophosphorylation | 2/97 | 27/18723 | 0.00857 | 0.027313 | 0.015968 | DDX3X/MRE11 | 2 |
| BP | GO:0060333 | | interferon-gamma-mediated signaling pathway | 2/97 | 27/18723 | 0.00857 | 0.027313 | 0.015968 | TP53/IRGM | 2 |
| BP | GO:1902914 | | regulation of protein polyubiquitination | 2/97 | 27/18723 | 0.00857 | 0.027313 | 0.015968 | DDX3X/BIRC2 | 2 |
| BP | GO:1903203 | | regulation of oxidative stress-induced neuron death | 2/97 | 27/18723 | 0.00857 | 0.027313 | 0.015968 | PARP1/TREM2 | 2 |
| BP | GO:0000422 | | autophagy of mitochondrion | 3/97 | 81/18723 | 0.00859 | 0.027313 | 0.015968 | HDAC6/TP53/BNIP3 | 3 |
| BP | GO:0010833 | | telomere maintenance via telomere lengthening | 3/97 | 81/18723 | 0.00859 | 0.027313 | 0.015968 | NEK7/MRE11/PARP1 | 3 |
| BP | GO:0044773 | | mitotic DNA damage checkpoint signaling | 3/97 | 81/18723 | 0.00859 | 0.027313 | 0.015968 | MRE11/MDM2/TP53 | 3 |
| BP | GO:0046209 | | nitric oxide metabolic process | 3/97 | 81/18723 | 0.00859 | 0.027313 | 0.015968 | AKT1/TLR2/TNF | 3 |
| BP | GO:0048144 | | fibroblast proliferation | 3/97 | 81/18723 | 0.00859 | 0.027313 | 0.015968 | DHX9/TP53/ANXA2 | 3 |
| BP | GO:0048708 | | astrocyte differentiation | 3/97 | 81/18723 | 0.00859 | 0.027313 | 0.015968 | EGFR/TREM2/TNF | 3 |
| BP | GO:0061726 | | mitochondrion disassembly | 3/97 | 81/18723 | 0.00859 | 0.027313 | 0.015968 | HDAC6/TP53/BNIP3 | 3 |
| BP | GO:2001021 | | negative regulation of response to DNA damage stimulus | 3/97 | 81/18723 | 0.00859 | 0.027313 | 0.015968 | BCL2/SIRT1/MDM2 | 3 |
| BP | GO:0006606 | | protein import into nucleus | 4/97 | 155/18723 | 0.008632 | 0.027392 | 0.016014 | TXNIP/ELAVL1/AKT1/TP53 | 4 |
| BP | GO:0051147 | | regulation of muscle cell differentiation | 4/97 | 155/18723 | 0.008632 | 0.027392 | 0.016014 | BCL2/SIRT1/GPER1/MDM2 | 4 |
| BP | GO:0016358 | | dendrite development | 5/97 | 243/18723 | 0.008659 | 0.027453 | 0.01605 | PTEN/EEF2K/HDAC6/APOE/ALK | 5 |
| BP | GO:0001933 | | negative regulation of protein phosphorylation | 6/97 | 342/18723 | 0.008745 | 0.027671 | 0.016177 | SIRT1/PTEN/AKT1/CASP3/APOE/PYCARD | 6 |
| BP | GO:0045765 | | regulation of angiogenesis | 6/97 | 342/18723 | 0.008745 | 0.027671 | 0.016177 | SIRT1/NFE2L2/CXCL8/CHI3L1/TNF/CAMP | 6 |
| BP | GO:0046631 | | alpha-beta T cell activation | 4/97 | 156/18723 | 0.008825 | 0.027897 | 0.016309 | BCL2/PRDM1/NLRP3/IL18 | 4 |
| BP | GO:0001892 | | embryonic placenta development | 3/97 | 82/18723 | 0.008884 | 0.02795 | 0.01634 | PRDM1/AKT1/CASP8 | 3 |
| BP | GO:0016575 | | histone deacetylation | 3/97 | 82/18723 | 0.008884 | 0.02795 | 0.01634 | SIRT1/HDAC6/TP53 | 3 |
| BP | GO:0046364 | | monosaccharide biosynthetic process | 3/97 | 82/18723 | 0.008884 | 0.02795 | 0.01634 | SIRT1/SESN2/MST1 | 3 |
| BP | GO:0046889 | | positive regulation of lipid biosynthetic process | 3/97 | 82/18723 | 0.008884 | 0.02795 | 0.01634 | AKT1/APOE/TNF | 3 |
| BP | GO:2001057 | | reactive nitrogen species metabolic process | 3/97 | 82/18723 | 0.008884 | 0.02795 | 0.01634 | AKT1/TLR2/TNF | 3 |
| BP | GO:0000423 | | mitophagy | 2/97 | 28/18723 | 0.009199 | 0.028805 | 0.01684 | HDAC6/TP53 | 2 |
| BP | GO:0034123 | | positive regulation of toll-like receptor signaling pathway | 2/97 | 28/18723 | 0.009199 | 0.028805 | 0.01684 | DDX3X/TLR2 | 2 |
| BP | GO:0060765 | | regulation of androgen receptor signaling pathway | 2/97 | 28/18723 | 0.009199 | 0.028805 | 0.01684 | SIRT1/HDAC6 | 2 |
| BP | GO:0090344 | | negative regulation of cell aging | 2/97 | 28/18723 | 0.009199 | 0.028805 | 0.01684 | SIRT1/PTEN | 2 |
| BP | GO:1990776 | | response to angiotensin | 2/97 | 28/18723 | 0.009199 | 0.028805 | 0.01684 | NFKB1/NFE2L2 | 2 |
| BP | GO:0043488 | | regulation of mRNA stability | 4/97 | 158/18723 | 0.009218 | 0.028839 | 0.01686 | DHX9/METTL3/ELAVL1/AKT1 | 4 |
| BP | GO:0051170 | | import into nucleus | 4/97 | 159/18723 | 0.009419 | 0.02944 | 0.017211 | TXNIP/ELAVL1/AKT1/TP53 | 4 |
| BP | GO:1901342 | | regulation of vasculature development | 6/97 | 348/18723 | 0.009478 | 0.029541 | 0.01727 | SIRT1/NFE2L2/CXCL8/CHI3L1/TNF/CAMP | 6 |
| BP | GO:0032508 | | DNA duplex unwinding | 3/97 | 84/18723 | 0.009487 | 0.029541 | 0.01727 | DDX3X/MRE11/DHX9 | 3 |
| BP | GO:0042310 | | vasoconstriction | 3/97 | 84/18723 | 0.009487 | 0.029541 | 0.01727 | EGFR/AKT1/ACE2 | 3 |
| BP | GO:1900542 | | regulation of purine nucleotide metabolic process | 3/97 | 84/18723 | 0.009487 | 0.029541 | 0.01727 | P2RX7/PARP1/TREM2 | 3 |
| BP | GO:1901988 | | negative regulation of cell cycle phase transition | 5/97 | 249/18723 | 0.009559 | 0.029737 | 0.017385 | BCL2/PTEN/MRE11/MDM2/TP53 | 5 |
| BP | GO:0043271 | | negative regulation of ion transport | 4/97 | 160/18723 | 0.009623 | 0.02988 | 0.017469 | BCL2/PTEN/GSTO1/AKT1 | 4 |
| BP | GO:0060402 | | calcium ion transport into cytosol | 4/97 | 160/18723 | 0.009623 | 0.02988 | 0.017469 | BCL2/P2RX7/GPER1/GSTO1 | 4 |
| MF | GO:0048018 | | receptor ligand activity | 8/97 | 487/18368 | 0.004245 | 0.030079 | 0.023419 | CXCL8/IL18/IL32/TNF/IL1RN/IL36B/FGF21/IL36G | 8 |
| CC | GO:0043218 | | compact myelin | 2/97 | 14/19550 | 0.002133 | 0.030258 | 0.024554 | PTEN/ANXA2 | 2 |
| BP | GO:0032092 | | positive regulation of protein binding | 3/97 | 85/18723 | 0.009798 | 0.030347 | 0.017742 | STING1/APOE/ANXA2 | 3 |
| BP | GO:0044774 | | mitotic DNA integrity checkpoint signaling | 3/97 | 85/18723 | 0.009798 | 0.030347 | 0.017742 | MRE11/MDM2/TP53 | 3 |
| BP | GO:0002360 | | T cell lineage commitment | 2/97 | 29/18723 | 0.009847 | 0.030347 | 0.017742 | BCL2/TP53 | 2 |
| BP | GO:0031069 | | hair follicle morphogenesis | 2/97 | 29/18723 | 0.009847 | 0.030347 | 0.017742 | BCL2/TP63 | 2 |
| BP | GO:0045737 | | positive regulation of cyclin-dependent protein serine/threonine kinase activity | 2/97 | 29/18723 | 0.009847 | 0.030347 | 0.017742 | EGFR/AKT1 | 2 |
| BP | GO:0048753 | | pigment granule organization | 2/97 | 29/18723 | 0.009847 | 0.030347 | 0.017742 | BCL2/LYST | 2 |
| BP | GO:1902230 | | negative regulation of intrinsic apoptotic signaling pathway in response to DNA damage | 2/97 | 29/18723 | 0.009847 | 0.030347 | 0.017742 | BCL2/SIRT1 | 2 |
| BP | GO:2000406 | | positive regulation of T cell migration | 2/97 | 29/18723 | 0.009847 | 0.030347 | 0.017742 | FADD/PYCARD | 2 |
| CC | GO:0005635 | | nuclear envelope | 8/97 | 468/19550 | 0.002286 | 0.030523 | 0.024769 | BCL2/P2RX7/SIRT1/GPER1/EGFR/CPTP/PARP1/BNIP3 | 8 |
| MF | GO:0035613 | | RNA stem-loop binding | 2/97 | 19/18368 | 0.004452 | 0.03101 | 0.024144 | DDX3X/DHX9 | 2 |
| BP | GO:0006140 | | regulation of nucleotide metabolic process | 3/97 | 86/18723 | 0.010115 | 0.031143 | 0.018207 | P2RX7/PARP1/TREM2 | 3 |
| BP | GO:0060828 | | regulation of canonical Wnt signaling pathway | 5/97 | 253/18723 | 0.010194 | 0.031357 | 0.018332 | EGFR/NFKB1/DDX3X/FOXO3/APOE | 5 |
| MF | GO:0042826 | | histone deacetylase binding | 4/97 | 127/18368 | 0.004602 | 0.031367 | 0.024422 | PRDM1/HDAC6/TP53/PARP1 | 4 |
| MF | GO:0030546 | | signaling receptor activator activity | 8/97 | 495/18368 | 0.004677 | 0.031367 | 0.024422 | CXCL8/IL18/IL32/TNF/IL1RN/IL36B/FGF21/IL36G | 8 |
| MF | GO:0140678 | | molecular function inhibitor activity | 4/97 | 128/18368 | 0.004732 | 0.031367 | 0.024422 | TXNIP/BCL2/GLMN/ANXA2 | 4 |
| BP | GO:0010976 | | positive regulation of neuron projection development | 4/97 | 163/18723 | 0.010252 | 0.031479 | 0.018403 | NFE2L2/EEF2K/APOE/ALK | 4 |
| BP | GO:0071322 | | cellular response to carbohydrate stimulus | 4/97 | 163/18723 | 0.010252 | 0.031479 | 0.018403 | GPER1/FOXO3/TREM2/FGF21 | 4 |
| BP | GO:0006839 | | mitochondrial transport | 5/97 | 254/18723 | 0.010357 | 0.03177 | 0.018573 | BCL2/AKT1/TP53/BNIP3/GZMB | 5 |
| BP | GO:0051262 | | protein tetramerization | 3/97 | 87/18723 | 0.010437 | 0.03198 | 0.018696 | TP53/TP63/GBP5 | 3 |
| BP | GO:0006884 | | cell volume homeostasis | 2/97 | 30/18723 | 0.010515 | 0.03198 | 0.018696 | ANO6/P2RX7 | 2 |
| BP | GO:0010800 | | positive regulation of peptidyl-threonine phosphorylation | 2/97 | 30/18723 | 0.010515 | 0.03198 | 0.018696 | IRGM/CHI3L1 | 2 |
| BP | GO:0031571 | | mitotic G1 DNA damage checkpoint signaling | 2/97 | 30/18723 | 0.010515 | 0.03198 | 0.018696 | MDM2/TP53 | 2 |
| BP | GO:0045939 | | negative regulation of steroid metabolic process | 2/97 | 30/18723 | 0.010515 | 0.03198 | 0.018696 | NFKB1/APOE | 2 |
| BP | GO:0045940 | | positive regulation of steroid metabolic process | 2/97 | 30/18723 | 0.010515 | 0.03198 | 0.018696 | APOE/TNF | 2 |
| BP | GO:0070229 | | negative regulation of lymphocyte apoptotic process | 2/97 | 30/18723 | 0.010515 | 0.03198 | 0.018696 | FADD/ORMDL3 | 2 |
| BP | GO:0070498 | | interleukin-1-mediated signaling pathway | 2/97 | 30/18723 | 0.010515 | 0.03198 | 0.018696 | ZBP1/IL1RN | 2 |
| BP | GO:1903579 | | negative regulation of ATP metabolic process | 2/97 | 30/18723 | 0.010515 | 0.03198 | 0.018696 | TP53/PARP1 | 2 |
| BP | GO:0051924 | | regulation of calcium ion transport | 5/97 | 255/18723 | 0.010521 | 0.03198 | 0.018696 | BCL2/P2RX7/GPER1/GSTO1/AKT1 | 5 |
| BP | GO:1903522 | | regulation of blood circulation | 5/97 | 256/18723 | 0.010688 | 0.032456 | 0.018974 | EGFR/GSTO1/MDM2/AKT1/ACE2 | 5 |
| BP | GO:0030101 | | natural killer cell activation | 3/97 | 88/18723 | 0.010766 | 0.032662 | 0.019095 | PRDM1/CASP8/IL18 | 3 |
| BP | GO:0030307 | | positive regulation of cell growth | 4/97 | 166/18723 | 0.010907 | 0.033001 | 0.019293 | BCL2/EGFR/DDX3X/AKT1 | 4 |
| BP | GO:0043112 | | receptor metabolic process | 4/97 | 166/18723 | 0.010907 | 0.033001 | 0.019293 | CAPN1/CXCL8/APOE/ANXA2 | 4 |
| BP | GO:0061013 | | regulation of mRNA catabolic process | 4/97 | 166/18723 | 0.010907 | 0.033001 | 0.019293 | DHX9/METTL3/ELAVL1/AKT1 | 4 |
| MF | GO:0061629 | | RNA polymerase II-specific DNA-binding transcription factor binding | 6/97 | 299/18368 | 0.005073 | 0.033093 | 0.025766 | JUN/SIRT1/NFE2L2/DHX9/TP53/PARP1 | 6 |
| BP | GO:0014910 | | regulation of smooth muscle cell migration | 3/97 | 89/18723 | 0.0111 | 0.033553 | 0.019616 | BCL2/NFE2L2/MDM2 | 3 |
| BP | GO:0001782 | | B cell homeostasis | 2/97 | 31/18723 | 0.011203 | 0.033618 | 0.019654 | BCL2/CASP3 | 2 |
| BP | GO:0002828 | | regulation of type 2 immune response | 2/97 | 31/18723 | 0.011203 | 0.033618 | 0.019654 | NLRP3/IL18 | 2 |
| BP | GO:0030212 | | hyaluronan metabolic process | 2/97 | 31/18723 | 0.011203 | 0.033618 | 0.019654 | NFKB1/AKT1 | 2 |
| BP | GO:0036475 | | neuron death in response to oxidative stress | 2/97 | 31/18723 | 0.011203 | 0.033618 | 0.019654 | PARP1/TREM2 | 2 |
| BP | GO:0044819 | | mitotic G1/S transition checkpoint signaling | 2/97 | 31/18723 | 0.011203 | 0.033618 | 0.019654 | MDM2/TP53 | 2 |
| BP | GO:0048566 | | embryonic digestive tract development | 2/97 | 31/18723 | 0.011203 | 0.033618 | 0.019654 | CXCL8/TNF | 2 |
| BP | GO:0071480 | | cellular response to gamma radiation | 2/97 | 31/18723 | 0.011203 | 0.033618 | 0.019654 | MDM2/TP53 | 2 |
| BP | GO:2000144 | | positive regulation of DNA-templated transcription, initiation | 2/97 | 31/18723 | 0.011203 | 0.033618 | 0.019654 | JUN/TP53 | 2 |
| BP | GO:0006260 | | DNA replication | 5/97 | 260/18723 | 0.011373 | 0.034097 | 0.019934 | JUN/EGFR/MRE11/DHX9/TP53 | 5 |
| BP | GO:0032392 | | DNA geometric change | 3/97 | 90/18723 | 0.01144 | 0.034208 | 0.019998 | DDX3X/MRE11/DHX9 | 3 |
| BP | GO:0032436 | | positive regulation of proteasomal ubiquitin-dependent protein catabolic process | 3/97 | 90/18723 | 0.01144 | 0.034208 | 0.019998 | NFE2L2/MDM2/AKT1 | 3 |
| BP | GO:0099175 | | regulation of postsynapse organization | 3/97 | 90/18723 | 0.01144 | 0.034208 | 0.019998 | PTEN/EEF2K/APOE | 3 |
| MF | GO:0097110 | | scaffold protein binding | 3/97 | 67/18368 | 0.005356 | 0.034331 | 0.02673 | PANX1/CASP8/TREM2 | 3 |
| MF | GO:0004407 | | histone deacetylase activity | 2/97 | 21/18368 | 0.005429 | 0.034331 | 0.02673 | SIRT1/HDAC6 | 2 |
| BP | GO:0006937 | | regulation of muscle contraction | 4/97 | 169/18723 | 0.011589 | 0.03459 | 0.020222 | GPER1/GSTO1/ORMDL3/ACE2 | 4 |
| BP | GO:0021543 | | pallium development | 4/97 | 169/18723 | 0.011589 | 0.03459 | 0.020222 | PTEN/CASP3/BNIP3/ALK | 4 |
| BP | GO:0001667 | | ameboidal-type cell migration | 7/97 | 475/18723 | 0.01173 | 0.034978 | 0.020449 | SIRT1/PTEN/NFE2L2/HDAC6/AKT1/APOE/TNF | 7 |
| BP | GO:0002065 | | columnar/cuboidal epithelial cell differentiation | 3/97 | 91/18723 | 0.011787 | 0.035053 | 0.020493 | PRDM1/AKT1/TP63 | 3 |
| BP | GO:0002275 | | myeloid cell activation involved in immune response | 3/97 | 91/18723 | 0.011787 | 0.035053 | 0.020493 | PYCARD/TREM2/IL13RA2 | 3 |
| BP | GO:0045582 | | positive regulation of T cell differentiation | 3/97 | 91/18723 | 0.011787 | 0.035053 | 0.020493 | NLRP3/IL18/IL36B | 3 |
| BP | GO:0043487 | | regulation of RNA stability | 4/97 | 170/18723 | 0.011822 | 0.035127 | 0.020536 | DHX9/METTL3/ELAVL1/AKT1 | 4 |
| BP | GO:0003018 | | vascular process in circulatory system | 5/97 | 263/18723 | 0.011905 | 0.035135 | 0.02054 | GPER1/EGFR/AKT1/APOE/ACE2 | 5 |
| BP | GO:0010039 | | response to iron ion | 2/97 | 32/18723 | 0.01191 | 0.035135 | 0.02054 | BCL2/MDM2 | 2 |
| BP | GO:0010743 | | regulation of macrophage derived foam cell differentiation | 2/97 | 32/18723 | 0.01191 | 0.035135 | 0.02054 | NFKB1/IL18 | 2 |
| BP | GO:0043372 | | positive regulation of CD4-positive, alpha-beta T cell differentiation | 2/97 | 32/18723 | 0.01191 | 0.035135 | 0.02054 | NLRP3/IL18 | 2 |
| BP | GO:0045736 | | negative regulation of cyclin-dependent protein serine/threonine kinase activity | 2/97 | 32/18723 | 0.01191 | 0.035135 | 0.02054 | PTEN/CASP3 | 2 |
| BP | GO:0050850 | | positive regulation of calcium-mediated signaling | 2/97 | 32/18723 | 0.01191 | 0.035135 | 0.02054 | TREM2/TNF | 2 |
| BP | GO:0097345 | | mitochondrial outer membrane permeabilization | 2/97 | 32/18723 | 0.01191 | 0.035135 | 0.02054 | BNIP3/GZMB | 2 |
| BP | GO:1901532 | | regulation of hematopoietic progenitor cell differentiation | 2/97 | 32/18723 | 0.01191 | 0.035135 | 0.02054 | NFE2L2/METTL3 | 2 |
| BP | GO:0048525 | | negative regulation of viral process | 3/97 | 92/18723 | 0.012139 | 0.03578 | 0.020918 | IFI16/BST2/TNF | 3 |
| MF | GO:0033558 | | protein deacetylase activity | 2/97 | 22/18368 | 0.005952 | 0.036575 | 0.028477 | SIRT1/HDAC6 | 2 |
| MF | GO:0001227 | | DNA-binding transcription repressor activity, RNA polymerase II-specific | 6/97 | 310/18368 | 0.006028 | 0.036575 | 0.028477 | JUN/IFI16/PRDM1/NFKB1/FOXO3/IRF3 | 6 |
| MF | GO:0004857 | | enzyme inhibitor activity | 3/97 | 70/18368 | 0.006051 | 0.036575 | 0.028477 | TXNIP/GLMN/ANXA2 | 3 |
| BP | GO:0051591 | | response to cAMP | 3/97 | 93/18723 | 0.012497 | 0.036803 | 0.021516 | JUN/EEF2K/BIRC2 | 3 |
| BP | GO:0006694 | | steroid biosynthetic process | 4/97 | 173/18723 | 0.01254 | 0.036895 | 0.02157 | SIRT1/NFKB1/APOE/TNF | 4 |
| BP | GO:0010165 | | response to X-ray | 2/97 | 33/18723 | 0.012635 | 0.03698 | 0.021619 | TP53/CASP3 | 2 |
| BP | GO:0048730 | | epidermis morphogenesis | 2/97 | 33/18723 | 0.012635 | 0.03698 | 0.021619 | BCL2/TP63 | 2 |
| BP | GO:1903715 | | regulation of aerobic respiration | 2/97 | 33/18723 | 0.012635 | 0.03698 | 0.021619 | AKT1/BNIP3 | 2 |
| BP | GO:1904030 | | negative regulation of cyclin-dependent protein kinase activity | 2/97 | 33/18723 | 0.012635 | 0.03698 | 0.021619 | PTEN/CASP3 | 2 |
| BP | GO:1904031 | | positive regulation of cyclin-dependent protein kinase activity | 2/97 | 33/18723 | 0.012635 | 0.03698 | 0.021619 | EGFR/AKT1 | 2 |
| BP | GO:2000036 | | regulation of stem cell population maintenance | 2/97 | 33/18723 | 0.012635 | 0.03698 | 0.021619 | ELAVL1/TP63 | 2 |
| MF | GO:0001217 | | DNA-binding transcription repressor activity | 6/97 | 313/18368 | 0.00631 | 0.037584 | 0.029262 | JUN/IFI16/PRDM1/NFKB1/FOXO3/IRF3 | 6 |
| BP | GO:0030316 | | osteoclast differentiation | 3/97 | 94/18723 | 0.012862 | 0.037609 | 0.021987 | TREM2/ANXA2/TNF | 3 |
| MF | GO:0070412 | | R-SMAD binding | 2/97 | 23/18368 | 0.006497 | 0.037877 | 0.029491 | JUN/PARP1 | 2 |
| MF | GO:0003678 | | DNA helicase activity | 3/97 | 72/18368 | 0.006543 | 0.037877 | 0.029491 | DDX3X/MRE11/DHX9 | 3 |
| BP | GO:0052126 | | movement in host environment | 4/97 | 175/18723 | 0.013033 | 0.038077 | 0.02226 | EGFR/CXCL8/ACE2/CLEC5A | 4 |
| BP | GO:0008593 | | regulation of Notch signaling pathway | 3/97 | 95/18723 | 0.013232 | 0.038522 | 0.022521 | METTL3/AKT1/TP63 | 3 |
| BP | GO:0042982 | | amyloid precursor protein metabolic process | 3/97 | 95/18723 | 0.013232 | 0.038522 | 0.022521 | CASP3/APOE/TNF | 3 |
| BP | GO:0048709 | | oligodendrocyte differentiation | 3/97 | 95/18723 | 0.013232 | 0.038522 | 0.022521 | PTEN/TLR2/BNIP3 | 3 |
| BP | GO:1901379 | | regulation of potassium ion transmembrane transport | 3/97 | 95/18723 | 0.013232 | 0.038522 | 0.022521 | ANO6/PTEN/TREM2 | 3 |
| BP | GO:0010634 | | positive regulation of epithelial cell migration | 4/97 | 176/18723 | 0.013285 | 0.038641 | 0.02259 | SIRT1/NFE2L2/HDAC6/AKT1 | 4 |
| BP | GO:0001662 | | behavioral fear response | 2/97 | 34/18723 | 0.01338 | 0.038648 | 0.022594 | BCL2/APOE | 2 |
| BP | GO:0014072 | | response to isoquinoline alkaloid | 2/97 | 34/18723 | 0.01338 | 0.038648 | 0.022594 | FADD/MDM2 | 2 |
| BP | GO:0016242 | | negative regulation of macroautophagy | 2/97 | 34/18723 | 0.01338 | 0.038648 | 0.022594 | AKT1/TP53 | 2 |
| BP | GO:0039528 | | cytoplasmic pattern recognition receptor signaling pathway in response to virus | 2/97 | 34/18723 | 0.01338 | 0.038648 | 0.022594 | BIRC2/IRF3 | 2 |
| BP | GO:0043276 | | anoikis | 2/97 | 34/18723 | 0.01338 | 0.038648 | 0.022594 | BCL2/AKT1 | 2 |
| BP | GO:0043278 | | response to morphine | 2/97 | 34/18723 | 0.01338 | 0.038648 | 0.022594 | FADD/MDM2 | 2 |
| BP | GO:0048854 | | brain morphogenesis | 2/97 | 34/18723 | 0.01338 | 0.038648 | 0.022594 | PTEN/FOXO3 | 2 |
| BP | GO:2000352 | | negative regulation of endothelial cell apoptotic process | 2/97 | 34/18723 | 0.01338 | 0.038648 | 0.022594 | NFE2L2/FGF21 | 2 |
| BP | GO:0051962 | | positive regulation of nervous system development | 5/97 | 272/18723 | 0.013605 | 0.039239 | 0.02294 | GPER1/EGFR/EEF2K/TLR2/TNF | 5 |
| BP | GO:0042632 | | cholesterol homeostasis | 3/97 | 96/18723 | 0.013609 | 0.039239 | 0.02294 | SIRT1/APOE/IL18 | 3 |
| BP | GO:0009266 | | response to temperature stimulus | 4/97 | 178/18723 | 0.013796 | 0.039746 | 0.023236 | SIRT1/AKT1/CASP8/CD14 | 4 |
| BP | GO:0050878 | | regulation of body fluid levels | 6/97 | 379/18723 | 0.013982 | 0.040202 | 0.023503 | ANO6/EGFR/NFE2L2/APOE/ANXA2/TP63 | 6 |
| BP | GO:0014909 | | smooth muscle cell migration | 3/97 | 97/18723 | 0.013991 | 0.040202 | 0.023503 | BCL2/NFE2L2/MDM2 | 3 |
| BP | GO:0055092 | | sterol homeostasis | 3/97 | 97/18723 | 0.013991 | 0.040202 | 0.023503 | SIRT1/APOE/IL18 | 3 |
| BP | GO:0001936 | | regulation of endothelial cell proliferation | 4/97 | 179/18723 | 0.014057 | 0.040291 | 0.023555 | SIRT1/AKT1/APOE/TNF | 4 |
| BP | GO:0002209 | | behavioral defense response | 2/97 | 35/18723 | 0.014144 | 0.040291 | 0.023555 | BCL2/APOE | 2 |
| BP | GO:0032814 | | regulation of natural killer cell activation | 2/97 | 35/18723 | 0.014144 | 0.040291 | 0.023555 | PRDM1/IL18 | 2 |
| BP | GO:0033146 | | regulation of intracellular estrogen receptor signaling pathway | 2/97 | 35/18723 | 0.014144 | 0.040291 | 0.023555 | PARP1/TP63 | 2 |
| BP | GO:0034405 | | response to fluid shear stress | 2/97 | 35/18723 | 0.014144 | 0.040291 | 0.023555 | NFE2L2/AKT1 | 2 |
| BP | GO:0045907 | | positive regulation of vasoconstriction | 2/97 | 35/18723 | 0.014144 | 0.040291 | 0.023555 | EGFR/AKT1 | 2 |
| BP | GO:0045922 | | negative regulation of fatty acid metabolic process | 2/97 | 35/18723 | 0.014144 | 0.040291 | 0.023555 | SIRT1/AKT1 | 2 |
| BP | GO:1905207 | | regulation of cardiocyte differentiation | 2/97 | 35/18723 | 0.014144 | 0.040291 | 0.023555 | GPER1/EGFR | 2 |
| BP | GO:1905898 | | positive regulation of response to endoplasmic reticulum stress | 2/97 | 35/18723 | 0.014144 | 0.040291 | 0.023555 | SIRT1/NFE2L2 | 2 |
| BP | GO:2000403 | | positive regulation of lymphocyte migration | 2/97 | 35/18723 | 0.014144 | 0.040291 | 0.023555 | FADD/PYCARD | 2 |
| BP | GO:0007033 | | vacuole organization | 4/97 | 180/18723 | 0.014321 | 0.04069 | 0.023788 | LYST/STING1/IRGM/ANXA2 | 4 |
| BP | GO:0010721 | | negative regulation of cell development | 4/97 | 180/18723 | 0.014321 | 0.04069 | 0.023788 | PTEN/TP53/TREM2/TNF | 4 |
| BP | GO:0071897 | | DNA biosynthetic process | 4/97 | 180/18723 | 0.014321 | 0.04069 | 0.023788 | NEK7/SIRT1/MRE11/TP53 | 4 |
| BP | GO:0032535 | | regulation of cellular component size | 6/97 | 383/18723 | 0.014656 | 0.041606 | 0.024324 | ANO6/P2RX7/PTEN/AKT1/APOE/PYCARD | 6 |
| BP | GO:0031341 | | regulation of cell killing | 3/97 | 99/18723 | 0.014774 | 0.041907 | 0.0245 | P2RX7/FADD/RIPK3 | 3 |
| BP | GO:0006401 | | RNA catabolic process | 5/97 | 278/18723 | 0.014824 | 0.041977 | 0.024541 | DHX9/METTL3/ELAVL1/AKT1/POP1 | 5 |
| BP | GO:0022412 | | cellular process involved in reproduction in multicellular organism | 6/97 | 384/18723 | 0.014828 | 0.041977 | 0.024541 | BCL2/PRDM1/FOXO3/METTL3/PANX1/AKT1 | 6 |
| BP | GO:0002691 | | regulation of cellular extravasation | 2/97 | 36/18723 | 0.014926 | 0.041977 | 0.024541 | FADD/RIPK3 | 2 |
| BP | GO:0030224 | | monocyte differentiation | 2/97 | 36/18723 | 0.014926 | 0.041977 | 0.024541 | JUN/IFI16 | 2 |
| BP | GO:0042092 | | type 2 immune response | 2/97 | 36/18723 | 0.014926 | 0.041977 | 0.024541 | NLRP3/IL18 | 2 |
| BP | GO:0042119 | | neutrophil activation | 2/97 | 36/18723 | 0.014926 | 0.041977 | 0.024541 | CXCL8/IL18 | 2 |
| BP | GO:0045746 | | negative regulation of Notch signaling pathway | 2/97 | 36/18723 | 0.014926 | 0.041977 | 0.024541 | METTL3/AKT1 | 2 |
| BP | GO:0051354 | | negative regulation of oxidoreductase activity | 2/97 | 36/18723 | 0.014926 | 0.041977 | 0.024541 | NFKB1/HDAC6 | 2 |
| BP | GO:0060251 | | regulation of glial cell proliferation | 2/97 | 36/18723 | 0.014926 | 0.041977 | 0.024541 | EGFR/TNF | 2 |
| BP | GO:1902229 | | regulation of intrinsic apoptotic signaling pathway in response to DNA damage | 2/97 | 36/18723 | 0.014926 | 0.041977 | 0.024541 | BCL2/SIRT1 | 2 |
| BP | GO:0042326 | | negative regulation of phosphorylation | 6/97 | 385/18723 | 0.015001 | 0.042153 | 0.024643 | SIRT1/PTEN/AKT1/CASP3/APOE/PYCARD | 6 |
| BP | GO:0006641 | | triglyceride metabolic process | 3/97 | 100/18723 | 0.015175 | 0.04257 | 0.024887 | SIRT1/APOE/FGF21 | 3 |
| BP | GO:0060079 | | excitatory postsynaptic potential | 3/97 | 100/18723 | 0.015175 | 0.04257 | 0.024887 | P2RX7/PTEN/AKT1 | 3 |
| MF | GO:0005539 | | glycosaminoglycan binding | 5/97 | 230/18368 | 0.007477 | 0.042682 | 0.033232 | CTSG/NLRP3/TLR2/APOE/TREM2 | 5 |
| MF | GO:0001618 | | virus receptor activity | 3/97 | 76/18368 | 0.007597 | 0.042772 | 0.033301 | EGFR/ACE2/CLEC5A | 3 |
| BP | GO:0006476 | | protein deacetylation | 3/97 | 101/18723 | 0.015582 | 0.043638 | 0.025512 | SIRT1/HDAC6/TP53 | 3 |
| BP | GO:0042102 | | positive regulation of T cell proliferation | 3/97 | 101/18723 | 0.015582 | 0.043638 | 0.025512 | FADD/PYCARD/IL18 | 3 |
| MF | GO:0140272 | | exogenous protein binding | 3/97 | 77/18368 | 0.007875 | 0.043738 | 0.034054 | EGFR/ACE2/CLEC5A | 3 |
| BP | GO:0001990 | | regulation of systemic arterial blood pressure by hormone | 2/97 | 37/18723 | 0.015726 | 0.043856 | 0.025639 | CTSG/ACE2 | 2 |
| BP | GO:0002369 | | T cell cytokine production | 2/97 | 37/18723 | 0.015726 | 0.043856 | 0.025639 | NLRP3/IL18 | 2 |
| BP | GO:0002724 | | regulation of T cell cytokine production | 2/97 | 37/18723 | 0.015726 | 0.043856 | 0.025639 | NLRP3/IL18 | 2 |
| BP | GO:0032570 | | response to progesterone | 2/97 | 37/18723 | 0.015726 | 0.043856 | 0.025639 | TXNIP/TLR2 | 2 |
| BP | GO:0032885 | | regulation of polysaccharide biosynthetic process | 2/97 | 37/18723 | 0.015726 | 0.043856 | 0.025639 | NFKB1/AKT1 | 2 |
| MF | GO:0001221 | | transcription coregulator binding | 3/97 | 78/18368 | 0.008159 | 0.044519 | 0.034662 | NFE2L2/FOXO3/HDAC6 | 3 |
| MF | GO:0019903 | | protein phosphatase binding | 4/97 | 150/18368 | 0.008232 | 0.044519 | 0.034662 | BCL2/EGFR/AKT1/TP53 | 4 |
| BP | GO:1904063 | | negative regulation of cation transmembrane transport | 3/97 | 102/18723 | 0.015995 | 0.044569 | 0.026056 | PTEN/GSTO1/AKT1 | 3 |
| BP | GO:0015850 | | organic hydroxy compound transport | 5/97 | 284/18723 | 0.016116 | 0.044867 | 0.02623 | SIRT1/NFKB1/APOE/TREM2/ANXA2 | 5 |
| CC | GO:0005741 | | mitochondrial outer membrane | 5/97 | 205/19550 | 0.003567 | 0.044987 | 0.036507 | BCL2/STING1/FOXO3/CASP8/BNIP3 | 5 |
| BP | GO:0002695 | | negative regulation of leukocyte activation | 4/97 | 187/18723 | 0.016255 | 0.045216 | 0.026434 | GPER1/GLMN/CASP3/IL13RA2 | 4 |
| BP | GO:0003279 | | cardiac septum development | 3/97 | 103/18723 | 0.016414 | 0.045621 | 0.026671 | PRDM1/MDM2/TP53 | 3 |
| BP | GO:0010742 | | macrophage derived foam cell differentiation | 2/97 | 38/18723 | 0.016544 | 0.045714 | 0.026725 | NFKB1/IL18 | 2 |
| BP | GO:0033144 | | negative regulation of intracellular steroid hormone receptor signaling pathway | 2/97 | 38/18723 | 0.016544 | 0.045714 | 0.026725 | SIRT1/TP63 | 2 |
| BP | GO:0042596 | | fear response | 2/97 | 38/18723 | 0.016544 | 0.045714 | 0.026725 | BCL2/APOE | 2 |
| BP | GO:0071392 | | cellular response to estradiol stimulus | 2/97 | 38/18723 | 0.016544 | 0.045714 | 0.026725 | GPER1/EGFR | 2 |
| BP | GO:0090077 | | foam cell differentiation | 2/97 | 38/18723 | 0.016544 | 0.045714 | 0.026725 | NFKB1/IL18 | 2 |
| BP | GO:1902110 | | positive regulation of mitochondrial membrane permeability involved in apoptotic process | 2/97 | 38/18723 | 0.016544 | 0.045714 | 0.026725 | BNIP3/GZMB | 2 |
| BP | GO:1903580 | | positive regulation of ATP metabolic process | 2/97 | 38/18723 | 0.016544 | 0.045714 | 0.026725 | P2RX7/TREM2 | 2 |
| BP | GO:0032006 | | regulation of TOR signaling | 3/97 | 104/18723 | 0.01684 | 0.046414 | 0.027135 | SIRT1/SESN2/TREM2 | 3 |
| BP | GO:0045621 | | positive regulation of lymphocyte differentiation | 3/97 | 104/18723 | 0.01684 | 0.046414 | 0.027135 | NLRP3/IL18/IL36B | 3 |
| BP | GO:0046634 | | regulation of alpha-beta T cell activation | 3/97 | 104/18723 | 0.01684 | 0.046414 | 0.027135 | PRDM1/NLRP3/IL18 | 3 |
| CC | GO:0005771 | | multivesicular body | 3/97 | 64/19550 | 0.003963 | 0.047211 | 0.038312 | EGFR/HDAC6/BST2 | 3 |
| CC | GO:0034361 | | very-low-density lipoprotein particle | 2/97 | 20/19550 | 0.004368 | 0.047211 | 0.038312 | APOE/APOL1 | 2 |
| CC | GO:0034385 | | triglyceride-rich plasma lipoprotein particle | 2/97 | 20/19550 | 0.004368 | 0.047211 | 0.038312 | APOE/APOL1 | 2 |
| BP | GO:0001914 | | regulation of T cell mediated cytotoxicity | 2/97 | 39/18723 | 0.01738 | 0.047667 | 0.027867 | FADD/RIPK3 | 2 |
| BP | GO:0016233 | | telomere capping | 2/97 | 39/18723 | 0.01738 | 0.047667 | 0.027867 | NEK7/MRE11 | 2 |
| BP | GO:0045622 | | regulation of T-helper cell differentiation | 2/97 | 39/18723 | 0.01738 | 0.047667 | 0.027867 | NLRP3/IL18 | 2 |
| BP | GO:0071548 | | response to dexamethasone | 2/97 | 39/18723 | 0.01738 | 0.047667 | 0.027867 | EGFR/FOXO3 | 2 |
| BP | GO:0090207 | | regulation of triglyceride metabolic process | 2/97 | 39/18723 | 0.01738 | 0.047667 | 0.027867 | APOE/FGF21 | 2 |
| BP | GO:2000516 | | positive regulation of CD4-positive, alpha-beta T cell activation | 2/97 | 39/18723 | 0.01738 | 0.047667 | 0.027867 | NLRP3/IL18 | 2 |
| BP | GO:0006766 | | vitamin metabolic process | 3/97 | 106/18723 | 0.017709 | 0.048367 | 0.028276 | NFKB1/GSTO1/TNF | 3 |
| BP | GO:0019882 | | antigen processing and presentation | 3/97 | 106/18723 | 0.017709 | 0.048367 | 0.028276 | PYCARD/TREM2/CTSV | 3 |
| BP | GO:0042116 | | macrophage activation | 3/97 | 106/18723 | 0.017709 | 0.048367 | 0.028276 | TLR2/TREM2/TNF | 3 |
| BP | GO:0090263 | | positive regulation of canonical Wnt signaling pathway | 3/97 | 106/18723 | 0.017709 | 0.048367 | 0.028276 | EGFR/NFKB1/DDX3X | 3 |
| BP | GO:0099565 | | chemical synaptic transmission, postsynaptic | 3/97 | 106/18723 | 0.017709 | 0.048367 | 0.028276 | P2RX7/PTEN/AKT1 | 3 |
| BP | GO:0001935 | | endothelial cell proliferation | 4/97 | 193/18723 | 0.018037 | 0.049184 | 0.028754 | SIRT1/AKT1/APOE/TNF | 4 |
| BP | GO:0007565 | | female pregnancy | 4/97 | 193/18723 | 0.018037 | 0.049184 | 0.028754 | BCL2/PRDM1/AKT1/CTSV | 4 |
| BP | GO:0021761 | | limbic system development | 3/97 | 107/18723 | 0.018153 | 0.049355 | 0.028854 | PTEN/CASP3/ALK | 3 |
| BP | GO:0002714 | | positive regulation of B cell mediated immunity | 2/97 | 40/18723 | 0.018234 | 0.049355 | 0.028854 | TREM2/TNF | 2 |
| BP | GO:0002891 | | positive regulation of immunoglobulin mediated immune response | 2/97 | 40/18723 | 0.018234 | 0.049355 | 0.028854 | TREM2/TNF | 2 |
| BP | GO:0030501 | | positive regulation of bone mineralization | 2/97 | 40/18723 | 0.018234 | 0.049355 | 0.028854 | ANO6/P2RX7 | 2 |
| BP | GO:0045429 | | positive regulation of nitric oxide biosynthetic process | 2/97 | 40/18723 | 0.018234 | 0.049355 | 0.028854 | AKT1/TNF | 2 |
| BP | GO:0051281 | | positive regulation of release of sequestered calcium ion into cytosol | 2/97 | 40/18723 | 0.018234 | 0.049355 | 0.028854 | GPER1/GSTO1 | 2 |
| BP | GO:1901223 | | negative regulation of NIK/NF-kappaB signaling | 2/97 | 40/18723 | 0.018234 | 0.049355 | 0.028854 | NLRP3/DDX3X | 2 |
| BP | GO:1902686 | | mitochondrial outer membrane permeabilization involved in programmed cell death | 2/97 | 40/18723 | 0.018234 | 0.049355 | 0.028854 | BNIP3/GZMB | 2 |
| BP | GO:2000142 | | regulation of DNA-templated transcription, initiation | 2/97 | 40/18723 | 0.018234 | 0.049355 | 0.028854 | JUN/TP53 | 2 |
| BP | GO:0050777 | | negative regulation of immune response | 4/97 | 194/18723 | 0.018346 | 0.049616 | 0.029007 | IFI16/METTL3/TREM2/IL13RA2 | 4 |
