# Supplementary material for: A Novel Defined Pyroptosis-Related Gene Signature for Predicting the Prognosis of Endometrial Cancer
Source: Dis Markers. 2022 Dec 16;2022:7570494. doi: 10.1155/2022/7570494 (PMC9806687; doi:10.1155/2022/7570494)
Supplement: Supplementary 4 — Table S4: KEGG pathway analysis. [file 7570494.f4.docx]

Tabel S4 KEGG pathway analysis

| ID | Description | GeneRatio | BgRatio | p value | p.adjust | q value | Count |
| --- | --- | --- | --- | --- | --- | --- | --- |
| hsa04621 | NOD-like receptor signaling pathway | 28/78 | 184/8115 | 5.16E-27 | 1.01E-24 | 5.26E-25 | 28 |
| hsa05132 | Salmonella infection | 21/78 | 249/8115 | 8.35E-15 | 8.18E-13 | 4.26E-13 | 21 |
| hsa05131 | Shigellosis | 20/78 | 247/8115 | 8.89E-14 | 5.81E-12 | 3.03E-12 | 20 |
| hsa05417 | Lipid and atherosclerosis | 18/78 | 215/8115 | 1.06E-12 | 5.20E-11 | 2.71E-11 | 18 |
| hsa04210 | Apoptosis | 15/78 | 136/8115 | 1.88E-12 | 7.39E-11 | 3.85E-11 | 15 |
| hsa05134 | Legionellosis | 10/78 | 57/8115 | 1.12E-10 | 3.65E-09 | 1.90E-09 | 10 |
| hsa05133 | Pertussis | 10/78 | 76/8115 | 2.13E-09 | 5.97E-08 | 3.11E-08 | 10 |
| hsa04620 | Toll-like receptor signaling pathway | 11/78 | 104/8115 | 3.41E-09 | 7.71E-08 | 4.02E-08 | 11 |
| hsa05161 | Hepatitis B | 13/78 | 162/8115 | 3.54E-09 | 7.71E-08 | 4.02E-08 | 13 |
| hsa04936 | Alcoholic liver disease | 12/78 | 142/8115 | 8.35E-09 | 1.64E-07 | 8.53E-08 | 12 |
| hsa01524 | Platinum drug resistance | 9/78 | 73/8115 | 2.58E-08 | 4.60E-07 | 2.40E-07 | 9 |
| hsa05145 | Toxoplasmosis | 10/78 | 112/8115 | 9.59E-08 | 1.57E-06 | 8.16E-07 | 10 |
| hsa04623 | Cytosolic DNA-sensing pathway | 8/78 | 63/8115 | 1.28E-07 | 1.93E-06 | 1.00E-06 | 8 |
| hsa04217 | Necroptosis | 11/78 | 159/8115 | 2.90E-07 | 3.86E-06 | 2.01E-06 | 11 |
| hsa04622 | RIG-I-like receptor signaling pathway | 8/78 | 70/8115 | 2.95E-07 | 3.86E-06 | 2.01E-06 | 8 |
| hsa05169 | Epstein-Barr virus infection | 12/78 | 202/8115 | 4.20E-07 | 5.14E-06 | 2.68E-06 | 12 |
| hsa05164 | Influenza A | 11/78 | 171/8115 | 6.04E-07 | 6.96E-06 | 3.63E-06 | 11 |
| hsa05135 | Yersinia infection | 10/78 | 137/8115 | 6.41E-07 | 6.98E-06 | 3.64E-06 | 10 |
| hsa05170 | Human immunodeficiency virus 1 infection | 12/78 | 212/8115 | 7.05E-07 | 7.19E-06 | 3.74E-06 | 12 |
| hsa05162 | Measles | 10/78 | 139/8115 | 7.33E-07 | 7.19E-06 | 3.74E-06 | 10 |
| hsa05152 | Tuberculosis | 11/78 | 180/8115 | 1.01E-06 | 9.40E-06 | 4.90E-06 | 11 |
| hsa04668 | TNF signaling pathway | 9/78 | 112/8115 | 1.08E-06 | 9.64E-06 | 5.02E-06 | 9 |
| hsa05163 | Human cytomegalovirus infection | 12/78 | 225/8115 | 1.33E-06 | 1.13E-05 | 5.90E-06 | 12 |
| hsa04218 | Cellular senescence | 10/78 | 156/8115 | 2.12E-06 | 1.73E-05 | 9.00E-06 | 10 |
| hsa05130 | Pathogenic Escherichia coli infection | 11/78 | 197/8115 | 2.45E-06 | 1.92E-05 | 1.00E-05 | 11 |
| hsa04657 | IL-17 signaling pathway | 8/78 | 94/8115 | 2.89E-06 | 2.18E-05 | 1.14E-05 | 8 |
| hsa05142 | Chagas disease | 8/78 | 102/8115 | 5.36E-06 | 3.89E-05 | 2.03E-05 | 8 |
| hsa04115 | p53 signaling pathway | 7/78 | 73/8115 | 5.65E-06 | 3.96E-05 | 2.06E-05 | 7 |
| hsa04064 | NF-kappa B signaling pathway | 8/78 | 104/8115 | 6.20E-06 | 4.05E-05 | 2.11E-05 | 8 |
| hsa04625 | C-type lectin receptor signaling pathway | 8/78 | 104/8115 | 6.20E-06 | 4.05E-05 | 2.11E-05 | 8 |
| hsa05418 | Fluid shear stress and atherosclerosis | 9/78 | 139/8115 | 6.55E-06 | 4.14E-05 | 2.16E-05 | 9 |
| hsa04215 | Apoptosis - multiple species | 5/78 | 32/8115 | 1.18E-05 | 7.26E-05 | 3.78E-05 | 5 |
| hsa05160 | Hepatitis C | 9/78 | 157/8115 | 1.76E-05 | 0.000105 | 5.45E-05 | 9 |
| hsa05235 | PD-L1 expression and PD-1 checkpoint pathway in cancer | 7/78 | 89/8115 | 2.11E-05 | 0.000122 | 6.35E-05 | 7 |
| hsa05222 | Small cell lung cancer | 7/78 | 92/8115 | 2.63E-05 | 0.000147 | 7.66E-05 | 7 |
| hsa05215 | Prostate cancer | 7/78 | 97/8115 | 3.71E-05 | 0.000202 | 0.000105 | 7 |
| hsa01522 | Endocrine resistance | 7/78 | 98/8115 | 3.96E-05 | 0.00021 | 0.000109 | 7 |
| hsa04933 | AGE-RAGE signaling pathway in diabetic complications | 7/78 | 100/8115 | 4.51E-05 | 0.000233 | 0.000121 | 7 |
| hsa05146 | Amoebiasis | 7/78 | 102/8115 | 5.13E-05 | 0.000258 | 0.000134 | 7 |
| hsa05218 | Melanoma | 6/78 | 72/8115 | 6.13E-05 | 0.0003 | 0.000156 | 6 |
| hsa05171 | Coronavirus disease - COVID-19 | 10/78 | 232/8115 | 6.87E-05 | 0.000329 | 0.000171 | 10 |
| hsa05167 | Kaposi sarcoma-associated herpesvirus infection | 9/78 | 194/8115 | 9.30E-05 | 0.000434 | 0.000226 | 9 |
| hsa05144 | Malaria | 5/78 | 50/8115 | 0.000109 | 0.000496 | 0.000259 | 5 |
| hsa04932 | Non-alcoholic fatty liver disease | 8/78 | 155/8115 | 0.000112 | 0.000498 | 0.000259 | 8 |
| hsa05203 | Viral carcinogenesis | 9/78 | 204/8115 | 0.000137 | 0.000595 | 0.00031 | 9 |
| hsa05210 | Colorectal cancer | 6/78 | 86/8115 | 0.000166 | 0.000706 | 0.000368 | 6 |
| hsa04211 | Longevity regulating pathway | 6/78 | 89/8115 | 0.0002 | 0.000835 | 0.000435 | 6 |
| hsa05213 | Endometrial cancer | 5/78 | 58/8115 | 0.000222 | 0.000906 | 0.000472 | 5 |
| hsa04068 | FoxO signaling pathway | 7/78 | 131/8115 | 0.000248 | 0.000992 | 0.000517 | 7 |
| hsa05208 | Chemical carcinogenesis - reactive oxygen species | 9/78 | 223/8115 | 0.000267 | 0.001046 | 0.000545 | 9 |
| hsa05165 | Human papillomavirus infection | 11/78 | 331/8115 | 0.000296 | 0.001136 | 0.000592 | 11 |
| hsa05321 | Inflammatory bowel disease | 5/78 | 65/8115 | 0.00038 | 0.001431 | 0.000745 | 5 |
| hsa05120 | Epithelial cell signaling in Helicobacter pylori infection | 5/78 | 70/8115 | 0.000536 | 0.001981 | 0.001032 | 5 |
| hsa05223 | Non-small cell lung cancer | 5/78 | 72/8115 | 0.00061 | 0.002176 | 0.001134 | 5 |
| hsa05219 | Bladder cancer | 4/78 | 41/8115 | 0.000611 | 0.002176 | 0.001134 | 4 |
| hsa05214 | Glioma | 5/78 | 75/8115 | 0.000736 | 0.002576 | 0.001342 | 5 |
| hsa05168 | Herpes simplex virus 1 infection | 13/78 | 495/8115 | 0.000787 | 0.002707 | 0.00141 | 13 |
| hsa01521 | EGFR tyrosine kinase inhibitor resistance | 5/78 | 79/8115 | 0.000933 | 0.003111 | 0.001621 | 5 |
| hsa04071 | Sphingolipid signaling pathway | 6/78 | 119/8115 | 0.000952 | 0.003111 | 0.001621 | 6 |
| hsa04722 | Neurotrophin signaling pathway | 6/78 | 119/8115 | 0.000952 | 0.003111 | 0.001621 | 6 |
| hsa04010 | MAPK signaling pathway | 9/78 | 294/8115 | 0.001931 | 0.0061 | 0.003178 | 9 |
| hsa05323 | Rheumatoid arthritis | 5/78 | 93/8115 | 0.001938 | 0.0061 | 0.003178 | 5 |
| hsa04151 | PI3K-Akt signaling pathway | 10/78 | 354/8115 | 0.001961 | 0.0061 | 0.003178 | 10 |
| hsa04613 | Neutrophil extracellular trap formation | 7/78 | 190/8115 | 0.002244 | 0.006871 | 0.003579 | 7 |
| hsa05202 | Transcriptional misregulation in cancer | 7/78 | 193/8115 | 0.002451 | 0.007391 | 0.00385 | 7 |
| hsa05206 | MicroRNAs in cancer | 9/78 | 310/8115 | 0.002768 | 0.00821 | 0.004277 | 9 |
| hsa05224 | Breast cancer | 6/78 | 147/8115 | 0.002806 | 0.00821 | 0.004277 | 6 |
| hsa05205 | Proteoglycans in cancer | 7/78 | 205/8115 | 0.003434 | 0.009897 | 0.005156 | 7 |
| hsa05010 | Alzheimer disease | 10/78 | 384/8115 | 0.003542 | 0.010062 | 0.005242 | 10 |
| hsa05230 | Central carbon metabolism in cancer | 4/78 | 70/8115 | 0.004482 | 0.012549 | 0.006538 | 4 |
| hsa04137 | Mitophagy - animal | 4/78 | 72/8115 | 0.004957 | 0.013684 | 0.007128 | 4 |
| hsa05143 | African trypanosomiasis | 3/78 | 37/8115 | 0.005248 | 0.014287 | 0.007443 | 3 |
| hsa05225 | Hepatocellular carcinoma | 6/78 | 168/8115 | 0.005406 | 0.014516 | 0.007562 | 6 |
| hsa04152 | AMPK signaling pathway | 5/78 | 120/8115 | 0.005815 | 0.015402 | 0.008024 | 5 |
| hsa05212 | Pancreatic cancer | 4/78 | 76/8115 | 0.006006 | 0.015489 | 0.008069 | 4 |
| hsa05220 | Chronic myeloid leukemia | 4/78 | 76/8115 | 0.006006 | 0.015489 | 0.008069 | 4 |
| hsa05140 | Leishmaniasis | 4/78 | 77/8115 | 0.006289 | 0.016009 | 0.00834 | 4 |
| hsa04060 | Cytokine-cytokine receptor interaction | 8/78 | 295/8115 | 0.00715 | 0.017967 | 0.00936 | 8 |
| hsa04380 | Osteoclast differentiation | 5/78 | 128/8115 | 0.007608 | 0.018876 | 0.009833 | 5 |
| hsa04915 | Estrogen signaling pathway | 5/78 | 138/8115 | 0.010352 | 0.025362 | 0.013212 | 5 |
| hsa04510 | Focal adhesion | 6/78 | 201/8115 | 0.012555 | 0.030381 | 0.015827 | 6 |
| hsa05226 | Gastric cancer | 5/78 | 149/8115 | 0.014082 | 0.03366 | 0.017535 | 5 |
| hsa05207 | Chemical carcinogenesis - receptor activation | 6/78 | 212/8115 | 0.015977 | 0.03773 | 0.019655 | 6 |
| hsa04660 | T cell receptor signaling pathway | 4/78 | 104/8115 | 0.017604 | 0.041077 | 0.021399 | 4 |
| hsa05166 | Human T-cell leukemia virus 1 infection | 6/78 | 222/8115 | 0.019609 | 0.045216 | 0.023555 | 6 |
| hsa04931 | Insulin resistance | 4/78 | 108/8115 | 0.019939 | 0.045443 | 0.023673 | 4 |
| hsa04614 | Renin-angiotensin system | 2/78 | 23/8115 | 0.020251 | 0.045623 | 0.023767 | 2 |
| hsa04066 | HIF-1 signaling pathway | 4/78 | 109/8115 | 0.020552 | 0.045774 | 0.023846 | 4 |
| hsa04213 | Longevity regulating pathway - multiple species | 3/78 | 62/8115 | 0.021534 | 0.047422 | 0.024704 | 3 |
