# Supplementary material for: A Novel Defined Pyroptosis-Related Gene Signature for Predicting the Prognosis of Endometrial Cancer
Source: Dis Markers. 2022 Dec 16;2022:7570494. doi: 10.1155/2022/7570494 (PMC9806687; doi:10.1155/2022/7570494)
Supplement: Supplementary 5 — Table S5: univariate Cox regression analysis. [file 7570494.f5.docx]

Table S5. Univariate Cox regression analysis

| id | beta | HR (95% CI for HR) | wald.test | p.value |
| --- | --- | --- | --- | --- |
| age | 0.04032 | 1.041 (1.019-1.064) | 12.96 | 0.000318 |
| weight | -0.00321 | 0.9968 (0.988-1.006) | 0.5 | 0.4774 |
| histology | 1.009 | 2.742 (1.761-4.271) | 19.92 | 8.07E-06 |
| grade | 0.02729 | 1.028 (0.7776-1.358) | 0.04 | 0.8479 |
| stage | 1.496 | 4.464 (2.856-6.978) | 43.1 | 5.20E-11 |
| riskScore | 0.3061 | 1.358 (1.248-1.478) | 49.96 | 1.57E-12 |
