# Supplementary material for: A Novel Defined Pyroptosis-Related Gene Signature for Predicting the Prognosis of Endometrial Cancer
Source: Dis Markers. 2022 Dec 16;2022:7570494. doi: 10.1155/2022/7570494 (PMC9806687; doi:10.1155/2022/7570494)
Supplement: Supplementary 6 — Table S6: multivariate Cox regression analysis. [file 7570494.f6.docx]

Table S6. Multivariate Cox regression analysis

| id | coef | HR | HR.95L | HR.95H | pValue |
| --- | --- | --- | --- | --- | --- |
| age | 0.048481 | 1.049675 | 1.022465 | 1.07761 | 0.000297 |
| weight | 0.010293 | 1.010346 | 0.999905 | 1.020895 | 0.052124 |
| histology | 0.04701 | 1.048133 | 0.600303 | 1.830047 | 0.868691 |
| grade | 0.006665 | 1.006687 | 0.753611 | 1.344752 | 0.964014 |
| stage | 1.482988 | 4.406093 | 2.672939 | 7.263037 | 6.05E-09 |
| riskScore | 0.219299 | 1.245204 | 1.120345 | 1.383979 | 4.75E-05 |
