# Supplementary material for: A Novel Defined Pyroptosis-Related Gene Signature for Predicting the Prognosis of Endometrial Cancer
Source: Dis Markers. 2022 Dec 16;2022:7570494. doi: 10.1155/2022/7570494 (PMC9806687; doi:10.1155/2022/7570494)
Supplement: Supplementary 7 — Table S7: clinicopathological features as well as risk score. [file 7570494.f7.docx]

Tables S7. Clinicopathological features as well as riskScore

| submitter_id | age | weight | histology | grade | stage | riskScore |
| --- | --- | --- | --- | --- | --- | --- |
| TCGA-D1-A17C | 78 | 81 | Endometrioid | G1 | Stage I-II | 0.786177 |
| TCGA-D1-A1O7 | 60 | 96 | Endometrioid | G3 | Stage I-II | 0.515455 |
| TCGA-AX-A3G8 | 76 | 62 | Endometrioid | G2 | Stage I-II | 0.609521 |
| TCGA-D1-A17F | 68 | 79 | Endometrioid | G1 | Stage I-II | 0.844664 |
| TCGA-AX-A3G3 | 58 | 55 | Serous | G3 | Stage III-IV | 0.776337 |
| TCGA-D1-A1NS | 53 | 126 | Endometrioid | G3 | Stage I-II | 0.626411 |
| TCGA-D1-A17N | 46 | 134 | Endometrioid | G3 | Stage I-II | 0.793904 |
| TCGA-D1-A17A | 59 | 102 | Endometrioid | G1 | Stage I-II | 0.777911 |
| TCGA-AJ-A3EJ | 74 | 73 | Serous | G1 | Stage III-IV | 1.42431 |
| TCGA-D1-A101 | 60 | 82 | Endometrioid | G1 | Stage I-II | 0.487727 |
| TCGA-H5-A2HR | 71 | 95 | Endometrioid | G3 | Stage I-II | 1.192969 |
| TCGA-D1-A17Q | 54 | 68 | Endometrioid | G3 | Stage I-II | 0.975171 |
| TCGA-BG-A221 | 84 | 62 | Endometrioid | G1 | Stage I-II | 1.308519 |
| TCGA-D1-A15Z | 72 | 125 | Endometrioid | G3 | Stage I-II | 1.569299 |
| TCGA-D1-A1O0 | 77 | 117 | Endometrioid | G3 | Stage I-II | 8.167739 |
| TCGA-D1-A17T | 67 | 83 | Endometrioid | G1 | Stage I-II | 0.530492 |
| TCGA-E6-A2P8 | 53 | 76 | Serous | G3 | Stage III-IV | 0.510223 |
| TCGA-D1-A16F | 60 | 90 | Endometrioid | G2 | Stage I-II | 1.515055 |
| TCGA-AJ-A8CT | 58 | 115 | Endometrioid | G2 | Stage I-II | 0.834737 |
| TCGA-AX-A2HK | 77 | 73 | Endometrioid | G2 | Stage III-IV | 10.14153 |
| TCGA-D1-A16X | 54 | 101 | Endometrioid | G3 | Stage I-II | 1.38585 |
| TCGA-D1-A160 | 70 | 122 | Endometrioid | G3 | Stage I-II | 0.641648 |
| TCGA-D1-A1O8 | 70 | 85 | Endometrioid | G3 | Stage III-IV | 0.576321 |
| TCGA-D1-A16V | 78 | 105 | Endometrioid | G2 | Stage III-IV | 0.715474 |
| TCGA-BG-A0W1 | 89 | 56 | Endometrioid | G2 | Stage I-II | 0.750157 |
| TCGA-D1-A3DG | 81 | 86 | Endometrioid | G3 | Stage III-IV | 5.281878 |
| TCGA-D1-A102 | 49 | 122 | Endometrioid | G1 | Stage I-II | 0.429728 |
| TCGA-QS-A8F1 | 85 | 75 | Serous | G3 | Stage III-IV | 3.743649 |
| TCGA-AX-A2H2 | 86 | 65 | Serous | G1 | Stage I-II | 2.699605 |
| TCGA-BS-A0TE | 35 | 91 | Endometrioid | G2 | Stage III-IV | 1.255452 |
| TCGA-D1-A0ZS | 54 | 62 | Endometrioid | G2 | Stage I-II | 0.303469 |
| TCGA-EY-A72D | 87 | 106 | Endometrioid | G3 | Stage I-II | 1.908021 |
| TCGA-EY-A1G7 | 86 | 85 | Endometrioid | G3 | Stage I-II | 2.445389 |
| TCGA-D1-A175 | 48 | 64 | Endometrioid | G1 | Stage I-II | 1.676563 |
| TCGA-AP-A1DV | 59 | 46 | Endometrioid | G3 | Stage I-II | 0.861708 |
| TCGA-D1-A17B | 69 | 110 | Endometrioid | G3 | Stage I-II | 0.803747 |
| TCGA-EC-A1QX | 71 | 90 | Endometrioid | G3 | Stage I-II | 0.727219 |
| TCGA-FI-A2D4 | 44 | 112 | Endometrioid | G3 | Stage III-IV | 1.775267 |
| TCGA-D1-A2G6 | 53 | 80 | Endometrioid | G3 | Stage III-IV | 1.445218 |
| TCGA-FI-A2EX | 58 | 89 | Serous | G3 | Stage III-IV | 2.05598 |
| TCGA-DI-A1NO | 68 | 122 | Endometrioid | G3 | Stage III-IV | 3.024043 |
| TCGA-KP-A3W1 | 76 | 66 | Serous | G3 | Stage I-II | 1.175633 |
| TCGA-BG-A222 | 49 | 132 | Endometrioid | G2 | Stage I-II | 1.361625 |
| TCGA-BK-A6W4 | 62 | 107 | Endometrioid | G1 | Stage I-II | 0.876601 |
| TCGA-BK-A6W3 | 34 | 89 | Endometrioid | G3 | Stage I-II | 0.445747 |
| TCGA-EY-A212 | 83 | 81 | Serous | G3 | Stage III-IV | 4.700559 |
| TCGA-D1-A17M | 56 | 142 | Endometrioid | G2 | Stage I-II | 1.34889 |
| TCGA-AJ-A23O | 69 | 60 | Endometrioid | G3 | Stage I-II | 2.287676 |
| TCGA-5S-A9Q8 | 51 | 86 | Endometrioid | G2 | Stage III-IV | 0.862721 |
| TCGA-D1-A16Q | 54 | 117 | Endometrioid | G3 | Stage I-II | 0.630479 |
| TCGA-BG-A0MA | 60 | 111 | Endometrioid | G3 | Stage I-II | 0.564486 |
| TCGA-EO-A1Y5 | 63 | 79 | Serous | G3 | Stage III-IV | 1.520562 |
| TCGA-EY-A1GW | 73 | 81 | Endometrioid | G3 | Stage III-IV | 0.399441 |
| TCGA-D1-A17R | 58 | 126 | Endometrioid | G1 | Stage I-II | 2.416445 |
| TCGA-A5-A7WJ | 64 | 64 | Endometrioid | G2 | Stage I-II | 1.66869 |
| TCGA-BG-A3EW | 62 | 126 | Endometrioid | G2 | Stage III-IV | 0.860174 |
| TCGA-EY-A4KR | 56 | 114 | Serous | G3 | Stage III-IV | 0.994215 |
| TCGA-D1-A16I | 62 | 84 | Serous | G2 | Stage I-II | 4.788752 |
| TCGA-B5-A1MY | 62 | 67 | Serous | G2 | Stage III-IV | 4.023463 |
| TCGA-AX-A06D | 82 | 91 | Endometrioid | G1 | Stage III-IV | 2.230458 |
| TCGA-BG-A0YV | 67 | 54 | Serous | G3 | Stage III-IV | 4.915784 |
| TCGA-EY-A210 | 82 | 63 | Serous | G3 | Stage III-IV | 0.317006 |
| TCGA-AP-A05O | 68 | 103 | Endometrioid | G1 | Stage I-II | 0.433886 |
| TCGA-AJ-A5DW | 56 | 109 | Endometrioid | G1 | Stage I-II | 0.492425 |
| TCGA-BK-A56F | 75 | 84 | Endometrioid | G3 | Stage I-II | 0.647157 |
| TCGA-D1-A1NU | 74 | 93 | Serous | G3 | Stage I-II | 1.555993 |
| TCGA-AX-A1C4 | 52 | 115 | Endometrioid | G1 | Stage I-II | 1.003432 |
| TCGA-KP-A3W0 | 72 | 94 | Serous | G3 | Stage I-II | 2.091733 |
| TCGA-B5-A0K9 | 88 | 65 | Endometrioid | G3 | Stage I-II | 1.476157 |
| TCGA-AX-A05S | 81 | 48 | Endometrioid | G3 | Stage III-IV | 1.02805 |
| TCGA-BG-A186 | 61 | 75 | Endometrioid | G3 | Stage III-IV | 1.159979 |
| TCGA-BG-A18A | 74 | 84 | Endometrioid | G3 | Stage I-II | 1.174845 |
| TCGA-K6-A3WQ | 60 | 99 | Serous | G3 | Stage III-IV | 1.87965 |
| TCGA-EY-A548 | 83 | 67 | Endometrioid | G3 | Stage I-II | 0.928679 |
| TCGA-BG-A220 | 69 | 127 | Endometrioid | G2 | Stage I-II | 0.574734 |
| TCGA-EY-A214 | 66 | 94 | Endometrioid | G3 | Stage III-IV | 1.130896 |
| TCGA-AX-A3GB | 73 | 87 | Endometrioid | G3 | Stage I-II | 0.472611 |
| TCGA-AJ-A3EK | 53 | 60 | Endometrioid | G2 | Stage I-II | 0.454441 |
| TCGA-B5-A1MW | 55 | 62 | Endometrioid | G2 | Stage I-II | 2.248493 |
| TCGA-EY-A54A | 67 | 70 | Endometrioid | G3 | Stage III-IV | 1.632708 |
| TCGA-BG-A187 | 65 | 61 | Endometrioid | G3 | Stage I-II | 0.696453 |
| TCGA-D1-A179 | 76 | 95 | Serous | G3 | Stage III-IV | 1.25008 |
| TCGA-AJ-A3OL | 55 | 64 | Endometrioid | G1 | Stage III-IV | 2.731753 |
| TCGA-BG-A0YU | 37 | 107 | Endometrioid | G2 | Stage I-II | 0.847601 |
| TCGA-A5-A3LP | 74 | 46 | Serous | G1 | Stage I-II | 1.672307 |
| TCGA-EY-A1G8 | 83 | 49 | Endometrioid | G3 | Stage I-II | 1.782304 |
| TCGA-D1-A1O5 | 61 | 80 | Endometrioid | G3 | Stage I-II | 0.934874 |
| TCGA-AJ-A3OJ | 54 | 104 | Endometrioid | G1 | Stage I-II | 1.057881 |
| TCGA-BG-A0RY | 68 | 118 | Endometrioid | G1 | Stage I-II | 2.29914 |
| TCGA-BG-A18B | 53 | 116 | Endometrioid | G1 | Stage I-II | 0.440036 |
| TCGA-BG-A18C | 72 | 96 | Endometrioid | G2 | Stage I-II | 1.3697 |
| TCGA-AJ-A3NG | 83 | 93 | Serous | G1 | Stage I-II | 1.986279 |
| TCGA-D1-A167 | 70 | 72 | Endometrioid | G1 | Stage I-II | 1.228333 |
| TCGA-EY-A1GV | 75 | 79 | Serous | G3 | Stage III-IV | 1.623381 |
| TCGA-D1-A3DA | 77 | 57 | Endometrioid | G3 | Stage III-IV | 0.454743 |
| TCGA-A5-A0VQ | 62 | 89 | Endometrioid | G1 | Stage I-II | 1.059198 |
| TCGA-AJ-A3OK | 73 | 75 | Serous | G3 | Stage I-II | 0.408703 |
| TCGA-EC-A1NJ | 73 | 62 | Endometrioid | G3 | Stage I-II | 0.349377 |
| TCGA-EY-A1GT | 68 | 70 | Endometrioid | G3 | Stage III-IV | 1.261759 |
| TCGA-DI-A0WH | 64 | 107 | Endometrioid | G3 | Stage I-II | 1.052148 |
| TCGA-EO-A3KW | 72 | 62 | Serous | G3 | Stage I-II | 1.962582 |
| TCGA-B5-A121 | 57 | 80 | Endometrioid | G3 | Stage III-IV | 0.986485 |
| TCGA-BK-A4ZD | 42 | 142 | Endometrioid | G2 | Stage I-II | 0.576079 |
| TCGA-B5-A11U | 74 | 61 | Endometrioid | G2 | Stage III-IV | 0.940393 |
| TCGA-EC-A24G | 57 | 93 | Endometrioid | G3 | Stage I-II | 0.723959 |
| TCGA-A5-AB3J | 52 | 59 | Endometrioid | G1 | Stage I-II | 0.587626 |
| TCGA-D1-A0ZN | 60 | 82 | Endometrioid | G2 | Stage I-II | 1.32574 |
| TCGA-AJ-A3QS | 57 | 71 | Serous | G2 | Stage III-IV | 1.466778 |
| TCGA-D1-A1NY | 67 | 97 | Endometrioid | G3 | Stage I-II | 0.464858 |
| TCGA-AJ-A3TW | 78 | 55 | Serous | G1 | Stage I-II | 0.661738 |
| TCGA-D1-A3JP | 61 | 59 | Serous | G3 | Stage I-II | 0.966 |
| TCGA-EY-A5W2 | 72 | 83 | Endometrioid | G3 | Stage I-II | 0.574951 |
| TCGA-AJ-A3I9 | 52 | 72 | Endometrioid | G2 | Stage I-II | 0.545499 |
| TCGA-D1-A3JQ | 61 | 121 | Serous | G3 | Stage III-IV | 0.763815 |
| TCGA-B5-A11Z | 61 | 84 | Endometrioid | G3 | Stage I-II | 0.476172 |
| TCGA-D1-A16Y | 56 | 87 | Endometrioid | G2 | Stage I-II | 3.384441 |
| TCGA-A5-A0R7 | 55 | 136 | Endometrioid | G2 | Stage I-II | 0.678472 |
| TCGA-EY-A1GR | 67 | 93 | Endometrioid | G2 | Stage I-II | 2.102108 |
| TCGA-AX-A2HH | 60 | 65 | Endometrioid | G3 | Stage I-II | 1.029387 |
| TCGA-JU-AAVI | 61 | 121 | Serous | G3 | Stage III-IV | 0.627422 |
| TCGA-A5-A0GA | 67 | 90 | Endometrioid | G2 | Stage III-IV | 1.004033 |
| TCGA-EY-A1GQ | 76 | 81 | Endometrioid | G3 | Stage I-II | 1.186429 |
| TCGA-EY-A547 | 75 | 108 | Endometrioid | G3 | Stage I-II | 0.584284 |
| TCGA-D1-A1NZ | 60 | 90 | Endometrioid | G3 | Stage I-II | 0.613613 |
| TCGA-D1-A177 | 70 | 53 | Endometrioid | G1 | Stage I-II | 0.898204 |
| TCGA-AJ-A3BG | 65 | 66 | Serous | G3 | Stage I-II | 3.041515 |
| TCGA-AJ-A3BF | 65 | 113 | Serous | G2 | Stage III-IV | 1.91017 |
| TCGA-KP-A3W4 | 63 | 61 | Serous | G3 | Stage III-IV | 1.110656 |
| TCGA-D1-A1NX | 66 | 87 | Serous | G3 | Stage III-IV | 1.318242 |
| TCGA-SJ-A6ZJ | 61 | 132 | Endometrioid | G3 | Stage I-II | 0.581953 |
| TCGA-AX-A3G9 | 63 | 60 | Endometrioid | G2 | Stage III-IV | 0.636496 |
| TCGA-B5-A11Y | 59 | 63 | Endometrioid | G3 | Stage I-II | 0.738346 |
| TCGA-A5-A7WK | 71 | 97 | Serous | G2 | Stage I-II | 6.215924 |
| TCGA-BG-A0LW | 47 | 141 | Endometrioid | G2 | Stage I-II | 1.840693 |
| TCGA-D1-A16S | 70 | 59 | Serous | G2 | Stage III-IV | 3.735671 |
| TCGA-EY-A1GP | 54 | 72 | Endometrioid | G3 | Stage I-II | 0.738454 |
| TCGA-D1-A3DH | 71 | 76 | Endometrioid | G3 | Stage I-II | 2.599709 |
| TCGA-EY-A215 | 60 | 49 | Endometrioid | G3 | Stage I-II | 1.507527 |
| TCGA-AJ-A3BI | 67 | 100 | Endometrioid | G1 | Stage I-II | 0.359413 |
| TCGA-EY-A1H0 | 57 | 90 | Endometrioid | G3 | Stage III-IV | 0.961264 |
| TCGA-BG-A0M0 | 66 | 117 | Endometrioid | G2 | Stage I-II | 0.817438 |
| TCGA-EO-A2CH | 73 | 66 | Serous | G3 | Stage III-IV | 10.72721 |
| TCGA-B5-A11W | 61 | 102 | Endometrioid | G1 | Stage I-II | 0.298659 |
| TCGA-D1-A174 | 51 | 65 | Endometrioid | G2 | Stage I-II | 1.081664 |
| TCGA-B5-A11V | 64 | 71 | Endometrioid | G3 | Stage I-II | 0.849749 |
| TCGA-A5-A0R8 | 81 | 62 | Endometrioid | G2 | Stage I-II | 0.881988 |
| TCGA-AJ-A2QL | 60 | 112 | Endometrioid | G3 | Stage III-IV | 0.446497 |
| TCGA-D1-A0ZO | 75 | 110 | Endometrioid | G3 | Stage I-II | 0.588695 |
| TCGA-EY-A549 | 78 | 74 | Endometrioid | G3 | Stage I-II | 0.878673 |
| TCGA-BG-A2AD | 63 | 80 | Endometrioid | G3 | Stage III-IV | 1.025555 |
| TCGA-EY-A2ON | 61 | 100 | Serous | G3 | Stage III-IV | 3.996618 |
| TCGA-BG-A0LX | 57 | 96 | Endometrioid | G2 | Stage I-II | 0.352261 |
| TCGA-AJ-A23M | 61 | 141 | Serous | G2 | Stage I-II | 1.716452 |
| TCGA-BG-A0MU | 78 | 68 | Endometrioid | G3 | Stage III-IV | 0.583439 |
| TCGA-AX-A3G1 | 84 | 61 | Endometrioid | G3 | Stage I-II | 2.693374 |
| TCGA-AJ-A3NE | 46 | 80 | Endometrioid | G3 | Stage I-II | 0.691464 |
| TCGA-SJ-A6ZI | 64 | 93 | Endometrioid | G3 | Stage I-II | 0.545932 |
| TCGA-AJ-A3IA | 77 | 58 | Serous | G3 | Stage I-II | 2.033078 |
| TCGA-AP-A05P | 54 | 74 | Endometrioid | G1 | Stage I-II | 0.653708 |
| TCGA-BG-A0M2 | 62 | 76 | Endometrioid | G3 | Stage I-II | 0.309391 |
| TCGA-AJ-A2QK | 65 | 119 | Endometrioid | G3 | Stage I-II | 0.24132 |
| TCGA-BG-A0MT | 64 | 73 | Endometrioid | G3 | Stage I-II | 0.928253 |
| TCGA-AJ-A3NF | 60 | 117 | Serous | G3 | Stage III-IV | 1.197688 |
| TCGA-AP-A3K1 | 56 | 60 | Serous | G3 | Stage III-IV | 1.44966 |
| TCGA-AP-A052 | 59 | 126 | Serous | G2 | Stage III-IV | 6.301112 |
| TCGA-D1-A103 | 87 | 60 | Endometrioid | G2 | Stage I-II | 0.461137 |
| TCGA-EY-A1GS | 71 | 62 | Serous | G3 | Stage III-IV | 3.413801 |
| TCGA-BG-A0M6 | 73 | 55 | Serous | G1 | Stage III-IV | 2.687858 |
| TCGA-EO-A3KU | 68 | 65 | Serous | G3 | Stage I-II | 1.652344 |
| TCGA-AJ-A3EM | 69 | 82 | Endometrioid | G1 | Stage I-II | 2.724722 |
| TCGA-AJ-A5DV | 65 | 147 | Endometrioid | G2 | Stage I-II | 0.682567 |
| TCGA-AJ-A3EL | 47 | 57 | Endometrioid | G3 | Stage I-II | 0.488545 |
| TCGA-D1-A0ZZ | 80 | 51 | Serous | G3 | Stage I-II | 2.060509 |
| TCGA-QF-A5YS | 57 | 79 | Serous | G3 | Stage I-II | 0.991471 |
| TCGA-EY-A1GK | 74 | 105 | Endometrioid | G3 | Stage I-II | 0.188141 |
| TCGA-EY-A2OM | 55 | 110 | Endometrioid | G3 | Stage I-II | 0.744331 |
| TCGA-D1-A17U | 53 | 87 | Endometrioid | G3 | Stage III-IV | 0.783366 |
| TCGA-AX-A1CR | 70 | 97 | Serous | G1 | Stage I-II | 0.677437 |
| TCGA-A5-A0GW | 46 | 49 | Endometrioid | G2 | Stage I-II | 0.684825 |
| TCGA-PG-A5BC | 72 | 87 | Serous | G3 | Stage I-II | 1.141941 |
| TCGA-AP-A054 | 64 | 75 | Endometrioid | G2 | Stage III-IV | 2.213315 |
| TCGA-EY-A1GI | 52 | 69 | Endometrioid | G1 | Stage I-II | 0.39482 |
| TCGA-EY-A1GH | 70 | 117 | Endometrioid | G3 | Stage I-II | 0.375943 |
| TCGA-BG-A0MI | 83 | 101 | Endometrioid | G1 | Stage I-II | 1.915909 |
| TCGA-QS-A5YR | 61 | 100 | Endometrioid | G3 | Stage III-IV | 0.468962 |
| TCGA-BG-A2AE | 57 | 135 | Serous | G3 | Stage I-II | 0.991337 |
| TCGA-KP-A3VZ | 69 | 55 | Serous | G3 | Stage III-IV | 2.979851 |
| TCGA-AP-A05N | 58 | 123 | Endometrioid | G3 | Stage I-II | 1.478693 |
| TCGA-D1-A0ZV | 58 | 77 | Endometrioid | G1 | Stage I-II | 0.932222 |
| TCGA-BK-A26L | 71 | 75 | Serous | G3 | Stage III-IV | 1.183127 |
| TCGA-D1-A17S | 59 | 109 | Endometrioid | G3 | Stage I-II | 1.177992 |
| TCGA-BS-A0TA | 58 | 90 | Endometrioid | G3 | Stage III-IV | 0.253288 |
| TCGA-EY-A1GE | 67 | 103 | Endometrioid | G3 | Stage I-II | 1.86307 |
| TCGA-A5-A0R9 | 51 | 145 | Endometrioid | G3 | Stage I-II | 0.752871 |
| TCGA-A5-A2K5 | 76 | 127 | Endometrioid | G2 | Stage I-II | 0.646657 |
| TCGA-QS-A5YQ | 55 | 83 | Endometrioid | G2 | Stage III-IV | 0.77471 |
| TCGA-BK-A13C | 47 | 93 | Endometrioid | G2 | Stage I-II | 0.812933 |
| TCGA-AP-A0LV | 39 | 76 | Endometrioid | G3 | Stage I-II | 0.65576 |
| TCGA-FI-A2D6 | 74 | 95 | Endometrioid | G3 | Stage I-II | 1.594381 |
| TCGA-AX-A2H7 | 87 | 60 | Endometrioid | G3 | Stage I-II | 0.748648 |
| TCGA-A5-A3LO | 64 | 103 | Endometrioid | G3 | Stage I-II | 2.521461 |
| TCGA-AX-A1C8 | 77 | 66 | Serous | G2 | Stage III-IV | 1.249951 |
| TCGA-DF-A2L0 | 60 | 95 | Endometrioid | G3 | Stage III-IV | 2.895148 |
| TCGA-AX-A1CC | 65 | 99 | Serous | G3 | Stage I-II | 1.392489 |
| TCGA-KP-A3W3 | 72 | 126 | Serous | G3 | Stage I-II | 1.496235 |
| TCGA-AX-A1C9 | 73 | 73 | Endometrioid | G2 | Stage I-II | 0.912346 |
| TCGA-D1-A17L | 81 | 123 | Endometrioid | G1 | Stage I-II | 0.298661 |
| TCGA-EO-A3B1 | 63 | 93 | Serous | G3 | Stage I-II | 1.068559 |
| TCGA-AP-A0LM | 33 | 100 | Endometrioid | G2 | Stage III-IV | 0.524361 |
| TCGA-EY-A1GF | 75 | 71 | Endometrioid | G3 | Stage I-II | 0.568337 |
| TCGA-EO-A3AZ | 80 | 78 | Serous | G3 | Stage I-II | 1.852755 |
| TCGA-B5-A0K3 | 62 | 88 | Endometrioid | G1 | Stage I-II | 2.420921 |
| TCGA-EY-A3L3 | 82 | 92 | Serous | G3 | Stage I-II | 2.931544 |
| TCGA-AX-A06H | 60 | 98 | Endometrioid | G3 | Stage III-IV | 0.332703 |
| TCGA-D1-A16G | 74 | 75 | Serous | G1 | Stage III-IV | 3.372642 |
| TCGA-B5-A3FH | 74 | 85 | Endometrioid | G3 | Stage I-II | 0.336005 |
| TCGA-AP-A1E3 | 45 | 130 | Endometrioid | G3 | Stage III-IV | 0.571274 |
| TCGA-B5-A0K8 | 71 | 61 | Serous | G1 | Stage I-II | 0.775999 |
| TCGA-AX-A2HF | 67 | 70 | Serous | G2 | Stage I-II | 1.371532 |
| TCGA-D1-A17H | 61 | 96 | Endometrioid | G2 | Stage I-II | 1.147439 |
| TCGA-D1-A17K | 74 | 97 | Endometrioid | G2 | Stage I-II | 11.40049 |
| TCGA-A5-A0GU | 58 | 95 | Endometrioid | G2 | Stage I-II | 0.271103 |
| TCGA-A5-A2K4 | 69 | 51 | Serous | G3 | Stage III-IV | 1.568567 |
| TCGA-D1-A0ZU | 34 | 94 | Endometrioid | G2 | Stage I-II | 0.794225 |
| TCGA-A5-A0VO | 64 | 86 | Endometrioid | G2 | Stage I-II | 0.605331 |
| TCGA-FI-A2EY | 63 | 96 | Serous | G3 | Stage III-IV | 5.444079 |
| TCGA-A5-A0RA | 68 | 113 | Endometrioid | G3 | Stage I-II | 0.372207 |
| TCGA-EY-A2OQ | 61 | 79 | Endometrioid | G3 | Stage I-II | 1.487182 |
| TCGA-D1-A161 | 78 | 71 | Endometrioid | G1 | Stage I-II | 0.436175 |
| TCGA-AJ-A2QM | 67 | 74 | Serous | G3 | Stage I-II | 3.728928 |
| TCGA-EO-A1Y8 | 87 | 58 | Serous | G3 | Stage I-II | 1.812527 |
| TCGA-B5-A3S1 | 71 | 68 | Serous | G3 | Stage III-IV | 0.940957 |
| TCGA-AX-A05U | 56 | 62 | Endometrioid | G1 | Stage I-II | 0.783438 |
| TCGA-D1-A15X | 45 | 72 | Serous | G3 | Stage I-II | 0.448216 |
| TCGA-D1-A16J | 61 | 130 | Endometrioid | G3 | Stage I-II | 4.148964 |
| TCGA-D1-A0ZP | 59 | 58 | Serous | G3 | Stage III-IV | 2.241965 |
| TCGA-AX-A06J | 71 | 119 | Endometrioid | G3 | Stage I-II | 0.514979 |
| TCGA-D1-A15W | 58 | 126 | Endometrioid | G1 | Stage III-IV | 0.548896 |
| TCGA-AX-A2H4 | 66 | 84 | Serous | G2 | Stage III-IV | 1.113376 |
| TCGA-A5-A1OK | 63 | 87 | Endometrioid | G3 | Stage III-IV | 0.921971 |
| TCGA-D1-A176 | 67 | 94 | Endometrioid | G1 | Stage I-II | 0.902313 |
| TCGA-D1-A17D | 58 | 68 | Endometrioid | G3 | Stage I-II | 0.88368 |
| TCGA-FI-A2F8 | 64 | 99 | Serous | G3 | Stage I-II | 1.722598 |
| TCGA-AX-A2HJ | 35 | 56 | Endometrioid | G3 | Stage I-II | 0.634656 |
| TCGA-BS-A0U7 | 63 | 66 | Endometrioid | G1 | Stage I-II | 1.794108 |
| TCGA-BK-A139 | 74 | 59 | Endometrioid | G3 | Stage I-II | 0.465748 |
| TCGA-EY-A2OP | 63 | 57 | Endometrioid | G3 | Stage I-II | 1.679379 |
| TCGA-D1-A16N | 51 | 89 | Endometrioid | G1 | Stage III-IV | 4.862905 |
| TCGA-DI-A2QU | 67 | 93 | Serous | G3 | Stage III-IV | 0.640053 |
| TCGA-AX-A2IN | 63 | 100 | Endometrioid | G2 | Stage I-II | 1.077742 |
| TCGA-AP-A1DO | 62 | 80 | Endometrioid | G2 | Stage I-II | 0.696578 |
| TCGA-B5-A1N2 | 70 | 114 | Serous | G3 | Stage III-IV | 3.14019 |
| TCGA-BK-A13B | 58 | 131 | Endometrioid | G1 | Stage I-II | 0.57585 |
| TCGA-A5-A1OF | 47 | 70 | Serous | G3 | Stage I-II | 0.922277 |
| TCGA-A5-A1OG | 65 | 64 | Serous | G1 | Stage III-IV | 3.11638 |
| TCGA-AX-A1CA | 70 | 77 | Serous | G1 | Stage I-II | 2.200363 |
| TCGA-B5-A11Q | 64 | 88 | Endometrioid | G2 | Stage III-IV | 0.620092 |
| TCGA-EY-A3QX | 64 | 63 | Serous | G3 | Stage III-IV | 1.668914 |
| TCGA-D1-A163 | 50 | 109 | Endometrioid | G1 | Stage III-IV | 0.88493 |
| TCGA-EY-A1GM | 60 | 71 | Serous | G3 | Stage III-IV | 4.229466 |
| TCGA-A5-A0GB | 65 | 122 | Endometrioid | G3 | Stage I-II | 0.763079 |
| TCGA-EY-A1GU | 66 | 90 | Endometrioid | G3 | Stage I-II | 0.926525 |
| TCGA-AX-A1C5 | 47 | 107 | Endometrioid | G3 | Stage III-IV | 0.650835 |
| TCGA-A5-A1OJ | 31 | 94 | Endometrioid | G2 | Stage I-II | 0.663319 |
| TCGA-AX-A05T | 82 | 61 | Endometrioid | G1 | Stage I-II | 0.582792 |
| TCGA-E6-A8L9 | 61 | 78 | Serous | G3 | Stage I-II | 0.606385 |
| TCGA-EY-A1GL | 51 | 73 | Endometrioid | G3 | Stage I-II | 0.942773 |
| TCGA-AP-A0LO | 45 | 67 | Endometrioid | G3 | Stage I-II | 1.129039 |
| TCGA-D1-A16O | 44 | 102 | Endometrioid | G3 | Stage I-II | 0.980552 |
| TCGA-AJ-A3BK | 68 | 92 | Endometrioid | G3 | Stage I-II | 0.815781 |
| TCGA-AX-A1CE | 60 | 58 | Endometrioid | G3 | Stage I-II | 0.476259 |
| TCGA-AJ-A3BD | 57 | 85 | Serous | G3 | Stage III-IV | 0.969357 |
| TCGA-BG-A0VX | 58 | 157 | Endometrioid | G3 | Stage I-II | 0.985473 |
| TCGA-AX-A2HC | 53 | 118 | Endometrioid | G3 | Stage III-IV | 1.807253 |
| TCGA-AJ-A2QO | 85 | 67 | Endometrioid | G3 | Stage I-II | 0.607709 |
| TCGA-EO-A3AS | 86 | 58 | Endometrioid | G3 | Stage I-II | 1.932193 |
| TCGA-AX-A1C7 | 77 | 60 | Serous | G3 | Stage I-II | 2.182142 |
| TCGA-A5-A2K7 | 41 | 72 | Endometrioid | G1 | Stage I-II | 1.359182 |
| TCGA-BK-A0CC | 69 | 72 | Serous | G3 | Stage III-IV | 0.922132 |
| TCGA-AJ-A8CV | 58 | 86 | Endometrioid | G3 | Stage I-II | 0.365466 |
| TCGA-EO-A3AV | 51 | 45 | Endometrioid | G3 | Stage III-IV | 0.981332 |
| TCGA-AJ-A2QN | 60 | 132 | Endometrioid | G2 | Stage I-II | 0.998702 |
| TCGA-BG-A0M3 | 74 | 75 | Endometrioid | G1 | Stage I-II | 0.730504 |
| TCGA-AX-A060 | 77 | 87 | Endometrioid | G3 | Stage I-II | 1.543524 |
| TCGA-AP-A1DP | 70 | 83 | Endometrioid | G3 | Stage I-II | 0.912483 |
| TCGA-AX-A0IU | 79 | 76 | Serous | G2 | Stage III-IV | 1.59852 |
| TCGA-BK-A0C9 | 57 | 127 | Endometrioid | G2 | Stage I-II | 0.502194 |
| TCGA-BK-A0CB | 60 | 111 | Endometrioid | G1 | Stage III-IV | 1.092992 |
| TCGA-KJ-A3U4 | 55 | 96 | Serous | G3 | Stage I-II | 1.408816 |
| TCGA-D1-A168 | 67 | 76 | Endometrioid | G3 | Stage I-II | 1.635126 |
| TCGA-AP-A5FX | 68 | 80 | Serous | G1 | Stage III-IV | 3.11864 |
| TCGA-A5-A1OH | 86 | 59 | Serous | G2 | Stage III-IV | 3.63183 |
| TCGA-AP-A0LH | 60 | 83 | Serous | G1 | Stage I-II | 1.089121 |
| TCGA-D1-A16B | 64 | 86 | Endometrioid | G2 | Stage I-II | 0.453517 |
| TCGA-AX-A1CP | 84 | 55 | Serous | G2 | Stage I-II | 2.477495 |
| TCGA-B5-A0K1 | 69 | 117 | Endometrioid | G1 | Stage I-II | 0.654757 |
| TCGA-DI-A1BY | 63 | 125 | Serous | G3 | Stage I-II | 0.45383 |
| TCGA-D1-A16E | 73 | 60 | Endometrioid | G1 | Stage I-II | 0.600882 |
| TCGA-A5-A0GR | 69 | 88 | Endometrioid | G2 | Stage I-II | 0.627374 |
| TCGA-AP-A05J | 66 | 79 | Serous | G3 | Stage I-II | 2.02942 |
| TCGA-AJ-A3NC | 63 | 110 | Endometrioid | G1 | Stage I-II | 0.611616 |
| TCGA-EY-A1GX | 76 | 69 | Endometrioid | G3 | Stage III-IV | 0.327804 |
| TCGA-D1-A169 | 63 | 102 | Endometrioid | G1 | Stage III-IV | 0.638653 |
| TCGA-D1-A16D | 49 | 151 | Endometrioid | G3 | Stage I-II | 1.164061 |
| TCGA-AX-A1CN | 54 | 146 | Endometrioid | G2 | Stage I-II | 1.318663 |
| TCGA-D1-A162 | 69 | 100 | Endometrioid | G1 | Stage I-II | 0.94303 |
| TCGA-AX-A0IW | 67 | 61 | Serous | G3 | Stage III-IV | 4.502216 |
| TCGA-A5-A0R6 | 64 | 68 | Serous | G3 | Stage III-IV | 1.532291 |
| TCGA-EO-A3AY | 58 | 76 | Endometrioid | G3 | Stage I-II | 0.365807 |
| TCGA-AX-A2HG | 56 | 115 | Endometrioid | G1 | Stage I-II | 1.168321 |
| TCGA-EO-A3KX | 80 | 44 | Endometrioid | G3 | Stage I-II | 0.506031 |
| TCGA-PG-A7D5 | 62 | 79 | Serous | G3 | Stage I-II | 1.037843 |
| TCGA-DI-A1BU | 55 | 93 | Serous | G3 | Stage I-II | 0.730987 |
| TCGA-B5-A3FD | 70 | 115 | Endometrioid | G1 | Stage I-II | 1.481489 |
| TCGA-D1-A15V | 68 | 69 | Serous | G1 | Stage I-II | 2.700523 |
| TCGA-5B-A90C | 69 | 98 | Endometrioid | G3 | Stage I-II | 2.808341 |
| TCGA-QF-A5YT | 57 | 146 | Endometrioid | G3 | Stage I-II | 0.744098 |
| TCGA-AX-A0J0 | 47 | 53 | Endometrioid | G3 | Stage I-II | 0.877117 |
| TCGA-2E-A9G8 | 59 | 71 | Endometrioid | G1 | Stage III-IV | 2.395716 |
| TCGA-FI-A3PV | 68 | 105 | Serous | G3 | Stage I-II | 2.138229 |
| TCGA-BG-A0MC | 74 | 135 | Endometrioid | G3 | Stage I-II | 0.841625 |
| TCGA-A5-A0VP | 74 | 58 | Endometrioid | G3 | Stage I-II | 0.774703 |
| TCGA-BG-A0MO | 63 | 83 | Endometrioid | G1 | Stage I-II | 0.671318 |
| TCGA-AX-A0IZ | 53 | 60 | Endometrioid | G3 | Stage I-II | 0.879677 |
| TCGA-AX-A3FX | 69 | 85 | Endometrioid | G2 | Stage I-II | 0.367557 |
| TCGA-B5-A11M | 43 | 139 | Endometrioid | G2 | Stage I-II | 0.474367 |
| TCGA-AP-A05H | 75 | 89 | Serous | G3 | Stage I-II | 6.378665 |
| TCGA-E6-A2P9 | 65 | 125 | Endometrioid | G3 | Stage I-II | 0.950967 |
| TCGA-EY-A2OO | 56 | 79 | Serous | G3 | Stage I-II | 2.279314 |
| TCGA-AP-A1E1 | 74 | 73 | Endometrioid | G3 | Stage I-II | 0.776391 |
| TCGA-AX-A062 | 53 | 122 | Endometrioid | G2 | Stage I-II | 0.786311 |
| TCGA-EO-A22U | 83 | 59 | Endometrioid | G3 | Stage I-II | 1.039858 |
| TCGA-EO-A3B0 | 43 | 53 | Endometrioid | G2 | Stage III-IV | 0.710466 |
| TCGA-AX-A2HD | 69 | 51 | Endometrioid | G2 | Stage III-IV | 0.933002 |
| TCGA-A5-A0GP | 58 | 60 | Endometrioid | G2 | Stage I-II | 1.111706 |
| TCGA-AP-A0LJ | 42 | 80 | Endometrioid | G2 | Stage I-II | 1.047293 |
| TCGA-AP-A1DQ | 76 | 60 | Serous | G3 | Stage III-IV | 2.967296 |
| TCGA-BS-A0T9 | 39 | 109 | Endometrioid | G3 | Stage III-IV | 1.247023 |
| TCGA-A5-A0G3 | 61 | 64 | Serous | G3 | Stage III-IV | 8.169367 |
| TCGA-AP-A059 | 69 | 99 | Endometrioid | G3 | Stage I-II | 0.643629 |
| TCGA-B5-A1MX | 47 | 58 | Endometrioid | G3 | Stage I-II | 0.828657 |
| TCGA-BG-A0MG | 73 | 61 | Endometrioid | G3 | Stage I-II | 0.354084 |
| TCGA-B5-A0K0 | 48 | 140 | Endometrioid | G3 | Stage I-II | 1.227946 |
| TCGA-D1-A2G0 | 70 | 73 | Serous | G3 | Stage I-II | 2.759633 |
| TCGA-AP-A0L8 | 70 | 61 | Serous | G1 | Stage III-IV | 3.614647 |
| TCGA-AP-A1DR | 59 | 109 | Endometrioid | G2 | Stage III-IV | 0.488819 |
| TCGA-EO-A1Y7 | 65 | 95 | Endometrioid | G3 | Stage I-II | 0.481268 |
| TCGA-AP-A0LT | 57 | 91 | Endometrioid | G2 | Stage I-II | 1.577445 |
| TCGA-AX-A064 | 81 | 48 | Endometrioid | G2 | Stage I-II | 0.749436 |
| TCGA-EO-A3AU | 72 | 78 | Endometrioid | G3 | Stage I-II | 0.461104 |
| TCGA-B5-A1MU | 79 | 44 | Serous | G2 | Stage III-IV | 1.521529 |
| TCGA-BG-A0VV | 53 | 147 | Endometrioid | G1 | Stage I-II | 0.835361 |
| TCGA-EO-A3L0 | 76 | 110 | Endometrioid | G3 | Stage I-II | 0.569864 |
| TCGA-AP-A1E4 | 54 | 54 | Endometrioid | G1 | Stage I-II | 2.209418 |
| TCGA-EO-A2CG | 69 | 68 | Serous | G3 | Stage I-II | 1.177798 |
| TCGA-B5-A1MV | 84 | 72 | Endometrioid | G2 | Stage I-II | 1.109054 |
| TCGA-BG-A0VT | 56 | 144 | Endometrioid | G3 | Stage III-IV | 0.83024 |
| TCGA-BG-A0VW | 77 | 93 | Endometrioid | G1 | Stage I-II | 0.386657 |
| TCGA-B5-A1MZ | 54 | 114 | Endometrioid | G3 | Stage I-II | 0.350373 |
| TCGA-BG-A0VZ | 58 | 109 | Endometrioid | G1 | Stage III-IV | 0.301452 |
| TCGA-A5-A0GV | 67 | 63 | Endometrioid | G3 | Stage I-II | 0.803973 |
| TCGA-EY-A1GC | 62 | 96 | Endometrioid | G3 | Stage I-II | 0.581922 |
| TCGA-B5-A11J | 64 | 134 | Endometrioid | G3 | Stage I-II | 0.305851 |
| TCGA-B5-A0JT | 63 | 119 | Endometrioid | G2 | Stage III-IV | 1.091313 |
| TCGA-AP-A1DM | 60 | 92 | Endometrioid | G2 | Stage I-II | 1.707223 |
| TCGA-BG-A0W2 | 57 | 121 | Endometrioid | G3 | Stage I-II | 0.429288 |
| TCGA-B5-A0JR | 73 | 105 | Endometrioid | G1 | Stage I-II | 0.672096 |
| TCGA-AX-A06F | 59 | 84 | Endometrioid | G1 | Stage III-IV | 0.897791 |
| TCGA-EO-A22X | 36 | 56 | Endometrioid | G3 | Stage III-IV | 0.594376 |
| TCGA-B5-A0K4 | 51 | 75 | Endometrioid | G1 | Stage I-II | 1.038914 |
| TCGA-AP-A05D | 67 | 98 | Serous | G3 | Stage III-IV | 2.016948 |
| TCGA-B5-A0JX | 62 | 113 | Endometrioid | G3 | Stage I-II | 0.674743 |
| TCGA-E6-A1M0 | 56 | 114 | Endometrioid | G3 | Stage III-IV | 0.764621 |
| TCGA-FI-A2EW | 71 | 83 | Serous | G3 | Stage I-II | 0.970255 |
| TCGA-BS-A0V4 | 56 | 96 | Endometrioid | G3 | Stage I-II | 1.324114 |
| TCGA-BG-A0MQ | 71 | 101 | Endometrioid | G2 | Stage I-II | 1.044967 |
| TCGA-B5-A11E | 53 | 52 | Endometrioid | G1 | Stage I-II | 0.991151 |
| TCGA-AP-A1E0 | 40 | 59 | Endometrioid | G3 | Stage III-IV | 0.595706 |
| TCGA-A5-A0GM | 53 | 97 | Endometrioid | G2 | Stage I-II | 0.767695 |
| TCGA-A5-A0GN | 65 | 68 | Endometrioid | G2 | Stage I-II | 0.870176 |
| TCGA-AX-A06L | 63 | 112 | Endometrioid | G2 | Stage I-II | 0.452078 |
| TCGA-AX-A0IS | 52 | 132 | Endometrioid | G1 | Stage I-II | 1.092644 |
| TCGA-AX-A2H8 | 64 | 113 | Endometrioid | G1 | Stage I-II | 2.510649 |
| TCGA-EO-A22S | 58 | 84 | Endometrioid | G3 | Stage I-II | 1.439573 |
| TCGA-AP-A0LP | 76 | 60 | Endometrioid | G3 | Stage I-II | 0.897848 |
| TCGA-B5-A11N | 69 | 90 | Endometrioid | G3 | Stage I-II | 0.662814 |
| TCGA-EO-A22R | 56 | 88 | Endometrioid | G3 | Stage I-II | 0.857014 |
| TCGA-BG-A0MS | 53 | 65 | Endometrioid | G2 | Stage III-IV | 1.022216 |
| TCGA-BS-A0TI | 64 | 59 | Endometrioid | G1 | Stage I-II | 1.561254 |
| TCGA-BG-A0MH | 61 | 136 | Endometrioid | G2 | Stage I-II | 0.449635 |
| TCGA-B5-A0K7 | 64 | 61 | Endometrioid | G2 | Stage I-II | 1.197354 |
| TCGA-BS-A0V7 | 48 | 102 | Endometrioid | G3 | Stage I-II | 0.31737 |
| TCGA-BG-A0M7 | 60 | 102 | Endometrioid | G1 | Stage III-IV | 0.509283 |
| TCGA-B5-A0JN | 84 | 58 | Serous | G3 | Stage III-IV | 3.917255 |
| TCGA-B5-A0K6 | 58 | 132 | Endometrioid | G3 | Stage I-II | 0.964102 |
| TCGA-AX-A2IO | 83 | 64 | Serous | G3 | Stage I-II | 1.165751 |
| TCGA-A5-A0GJ | 44 | 115 | Endometrioid | G2 | Stage I-II | 2.68895 |
| TCGA-A5-A2K3 | 68 | 80 | Serous | G2 | Stage I-II | 0.257334 |
| TCGA-AP-A0LG | 54 | 76 | Endometrioid | G2 | Stage I-II | 1.031156 |
| TCGA-BG-A0M8 | 50 | 209 | Endometrioid | G2 | Stage I-II | 0.920933 |
| TCGA-BS-A0WQ | 55 | 93 | Endometrioid | G3 | Stage I-II | 1.712466 |
| TCGA-FI-A2CX | 82 | 52 | Endometrioid | G3 | Stage I-II | 0.58698 |
| TCGA-EO-A22T | 56 | 53 | Endometrioid | G3 | Stage I-II | 0.754144 |
| TCGA-AX-A06B | 72 | 93 | Endometrioid | G3 | Stage I-II | 0.782482 |
| TCGA-BS-A0TJ | 59 | 88 | Endometrioid | G3 | Stage I-II | 0.882766 |
| TCGA-FI-A2CY | 60 | 100 | Serous | G3 | Stage I-II | 1.451415 |
| TCGA-A5-A0GX | 53 | 108 | Endometrioid | G1 | Stage I-II | 0.89804 |
| TCGA-BG-A0M4 | 60 | 97 | Endometrioid | G3 | Stage I-II | 1.506347 |
| TCGA-AX-A2H5 | 67 | 87 | Serous | G3 | Stage III-IV | 0.679161 |
| TCGA-B5-A0JZ | 60 | 87 | Endometrioid | G3 | Stage III-IV | 0.449623 |
| TCGA-AX-A05Z | 37 | 66 | Endometrioid | G3 | Stage III-IV | 0.656857 |
| TCGA-AP-A1DH | 62 | 127 | Endometrioid | G3 | Stage I-II | 1.093665 |
| TCGA-B5-A3FC | 53 | 76 | Endometrioid | G3 | Stage I-II | 0.131276 |
| TCGA-B5-A0K2 | 54 | 84 | Endometrioid | G3 | Stage III-IV | 0.362099 |
| TCGA-BS-A0UT | 62 | 65 | Endometrioid | G3 | Stage I-II | 1.074297 |
| TCGA-BS-A0UV | 55 | 112 | Endometrioid | G3 | Stage III-IV | 0.508705 |
| TCGA-B5-A11S | 63 | 118 | Endometrioid | G2 | Stage I-II | 0.599958 |
| TCGA-FI-A2F4 | 64 | 74 | Endometrioid | G3 | Stage I-II | 0.344891 |
| TCGA-AX-A1CJ | 59 | 115 | Endometrioid | G2 | Stage I-II | 0.619622 |
| TCGA-A5-A0GQ | 76 | 57 | Endometrioid | G2 | Stage I-II | 0.559687 |
| TCGA-A5-A0GH | 57 | 55 | Endometrioid | G3 | Stage I-II | 1.925376 |
| TCGA-BG-A0M9 | 73 | 93 | Endometrioid | G3 | Stage I-II | 1.625978 |
| TCGA-AX-A1CF | 69 | 79 | Endometrioid | G3 | Stage III-IV | 1.359422 |
| TCGA-B5-A11R | 51 | 89 | Endometrioid | G2 | Stage I-II | 0.252034 |
| TCGA-AX-A0J1 | 80 | 57 | Endometrioid | G3 | Stage I-II | 0.367405 |
| TCGA-BS-A0TG | 60 | 64 | Endometrioid | G1 | Stage III-IV | 0.391846 |
| TCGA-BS-A0TD | 65 | 85 | Endometrioid | G2 | Stage I-II | 0.647083 |
| TCGA-FI-A3PX | 57 | 90 | Serous | G3 | Stage III-IV | 1.84246 |
| TCGA-E6-A1LZ | 76 | 65 | Serous | G3 | Stage I-II | 3.218483 |
| TCGA-AX-A063 | 63 | 88 | Endometrioid | G3 | Stage I-II | 0.397692 |
| TCGA-AX-A1CK | 58 | 72 | Endometrioid | G1 | Stage I-II | 0.571574 |
| TCGA-AX-A05W | 60 | 74 | Endometrioid | G3 | Stage I-II | 0.94951 |
| TCGA-AX-A2HA | 35 | 60 | Endometrioid | G3 | Stage I-II | 0.430428 |
| TCGA-BS-A0UL | 54 | 114 | Endometrioid | G3 | Stage I-II | 0.441008 |
| TCGA-BS-A0UJ | 68 | 52 | Endometrioid | G3 | Stage I-II | 1.114215 |
| TCGA-AP-A0LN | 56 | 119 | Endometrioid | G1 | Stage I-II | 0.817153 |
| TCGA-A5-A0G9 | 79 | 82 | Endometrioid | G2 | Stage I-II | 0.741868 |
| TCGA-BS-A0V8 | 68 | 85 | Endometrioid | G2 | Stage I-II | 0.313658 |
| TCGA-AP-A0LL | 56 | 72 | Endometrioid | G3 | Stage I-II | 0.821428 |
| TCGA-AX-A1CI | 61 | 74 | Endometrioid | G3 | Stage I-II | 0.734811 |
| TCGA-BS-A0TC | 69 | 68 | Endometrioid | G1 | Stage I-II | 1.043965 |
| TCGA-B5-A11O | 62 | 114 | Endometrioid | G2 | Stage I-II | 0.405732 |
| TCGA-BS-A0UF | 65 | 57 | Endometrioid | G3 | Stage I-II | 0.768406 |
| TCGA-B5-A0JV | 63 | 94 | Endometrioid | G3 | Stage I-II | 0.892674 |
| TCGA-AP-A056 | 64 | 56 | Endometrioid | G3 | Stage I-II | 0.363031 |
| TCGA-B5-A0JS | 54 | 110 | Endometrioid | G2 | Stage I-II | 0.25276 |
| TCGA-BS-A0UM | 64 | 90 | Endometrioid | G3 | Stage I-II | 1.198102 |
| TCGA-BS-A0VI | 58 | 91 | Endometrioid | G2 | Stage I-II | 0.739015 |
| TCGA-A5-A0GI | 63 | 66 | Endometrioid | G1 | Stage I-II | 0.832457 |
| TCGA-AX-A05Y | 57 | 92 | Endometrioid | G3 | Stage I-II | 1.279425 |
| TCGA-B5-A11H | 67 | 101 | Endometrioid | G3 | Stage III-IV | 0.459784 |
| TCGA-BS-A0V6 | 55 | 83 | Endometrioid | G3 | Stage I-II | 0.429924 |
| TCGA-AP-A1DK | 53 | 56 | Endometrioid | G3 | Stage I-II | 1.653404 |
| TCGA-B5-A3FB | 73 | 115 | Endometrioid | G1 | Stage I-II | 0.609463 |
| TCGA-AP-A0LF | 82 | 72 | Endometrioid | G1 | Stage III-IV | 0.434423 |
| TCGA-AP-A0LS | 63 | 81 | Endometrioid | G1 | Stage I-II | 0.212299 |
| TCGA-B5-A3FA | 73 | 74 | Endometrioid | G3 | Stage I-II | 0.493032 |
| TCGA-BS-A0U5 | 76 | 59 | Endometrioid | G2 | Stage I-II | 2.025038 |
| TCGA-FI-A2EU | 68 | 93 | Serous | G3 | Stage I-II | 2.858849 |
| TCGA-BS-A0U8 | 55 | 82 | Endometrioid | G1 | Stage III-IV | 0.387488 |
| TCGA-AP-A0LI | 67 | 86 | Serous | G2 | Stage III-IV | 1.4426 |
| TCGA-A5-A0GE | 38 | 57 | Endometrioid | G1 | Stage I-II | 0.273248 |
| TCGA-A5-A0G1 | 67 | 59 | Serous | G3 | Stage I-II | 0.55353 |
| TCGA-DF-A2KS | 69 | 66 | Endometrioid | G3 | Stage I-II | 1.214305 |
| TCGA-DI-A2QY | 64 | 85 | Serous | G3 | Stage I-II | 2.767187 |
| TCGA-AP-A0LE | 57 | 75 | Endometrioid | G2 | Stage I-II | 1.050713 |
| TCGA-A5-A0GD | 75 | 74 | Endometrioid | G1 | Stage I-II | 0.538802 |
| TCGA-B5-A11G | 71 | 77 | Endometrioid | G3 | Stage I-II | 0.591263 |
| TCGA-BS-A0UA | 68 | 64 | Endometrioid | G2 | Stage I-II | 0.81843 |
| TCGA-AP-A0LD | 63 | 68 | Endometrioid | G3 | Stage I-II | 1.76182 |
| TCGA-A5-A2K2 | 77 | 50 | Serous | G3 | Stage I-II | 1.713838 |
| TCGA-A5-A0GG | 76 | 64 | Endometrioid | G1 | Stage I-II | 1.02301 |
| TCGA-AP-A0L9 | 71 | 60 | Serous | G3 | Stage I-II | 1.90464 |
| TCGA-AP-A051 | 69 | 97 | Endometrioid | G1 | Stage I-II | 0.918944 |
| TCGA-B5-A3F9 | 56 | 155 | Endometrioid | G3 | Stage I-II | 0.833094 |
| TCGA-A5-A0G2 | 57 | 60 | Serous | G2 | Stage III-IV | 0.853701 |
| TCGA-B5-A1MR | 65 | 58 | Endometrioid | G2 | Stage III-IV | 0.685234 |
